# Charge Transfer from Quantum-Confined 0D, 1D, and 2D Nanocrystals

**DOI:** 10.1021/acs.chemrev.3c00742

**Published:** 2024-04-17

**Authors:** Qiuyang Li, Kaifeng Wu, Haiming Zhu, Ye Yang, Sheng He, Tianquan Lian

**Affiliations:** † Department of Physics, University of Michigan, 450 Church St, Ann Arbor, Michigan 48109, United States; ‡ State Key Laboratory of Molecular Reaction Dynamics and Collaborative Innovation Center of Chemistry for Energy Materials (iChEM), Dalian Institute of Chemical Physics, Chinese Academy of Sciences, Dalian, Liaoning 116023, China; § University of Chinese Academy of Sciences, Beijing 100049, China; ∥ Department of Chemistry, Zhejiang University, Hangzhou, Zhejiang 310027, China; ⊥ The State Key Laboratory of Physical Chemistry of Solid Surfaces, iChEM (Collaborative Innovation Center of Chemistry for Energy Materials), College of Chemistry & Chemical Engineering, Xiamen University, Xiamen, Fujian 361005, China; # Department of Chemistry, Emory University, Atlanta, Georgia 30322, United States

## Abstract

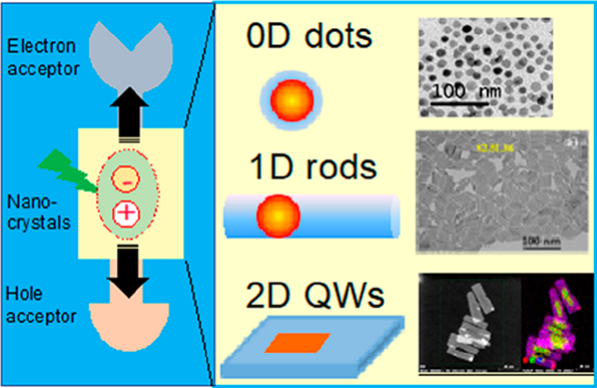

The properties of colloidal quantum-confined semiconductor nanocrystals (NCs), including zero-dimensional (0D) quantum dots, 1D nanorods, 2D nanoplatelets, and their heterostructures, can be tuned through their size, dimensionality, and material composition. In their photovoltaic and photocatalytic applications, a key step is to generate spatially separated and long-lived electrons and holes by interfacial charge transfer. These charge transfer properties have been extensively studied recently, which is the subject of this Review. The Review starts with a summary of the electronic structure and optical properties of 0D–2D nanocrystals, followed by the advances in wave function engineering, a novel way to control the spatial distribution of electrons and holes, through their size, dimension, and composition. It discusses the dependence of NC charge transfer on various parameters and the development of the Auger-assisted charge transfer model. Recent advances in understanding multiple exciton generation, decay, and dissociation are also discussed, with an emphasis on multiple carrier transfer. Finally, the applications of nanocrystal-based systems for photocatalysis are reviewed, focusing on the photodriven charge separation and recombination processes that dictate the function and performance of these materials. The Review ends with a summary and outlook of key remaining challenges and promising future directions in the field.

## 1 Introduction

**Figure 1:**
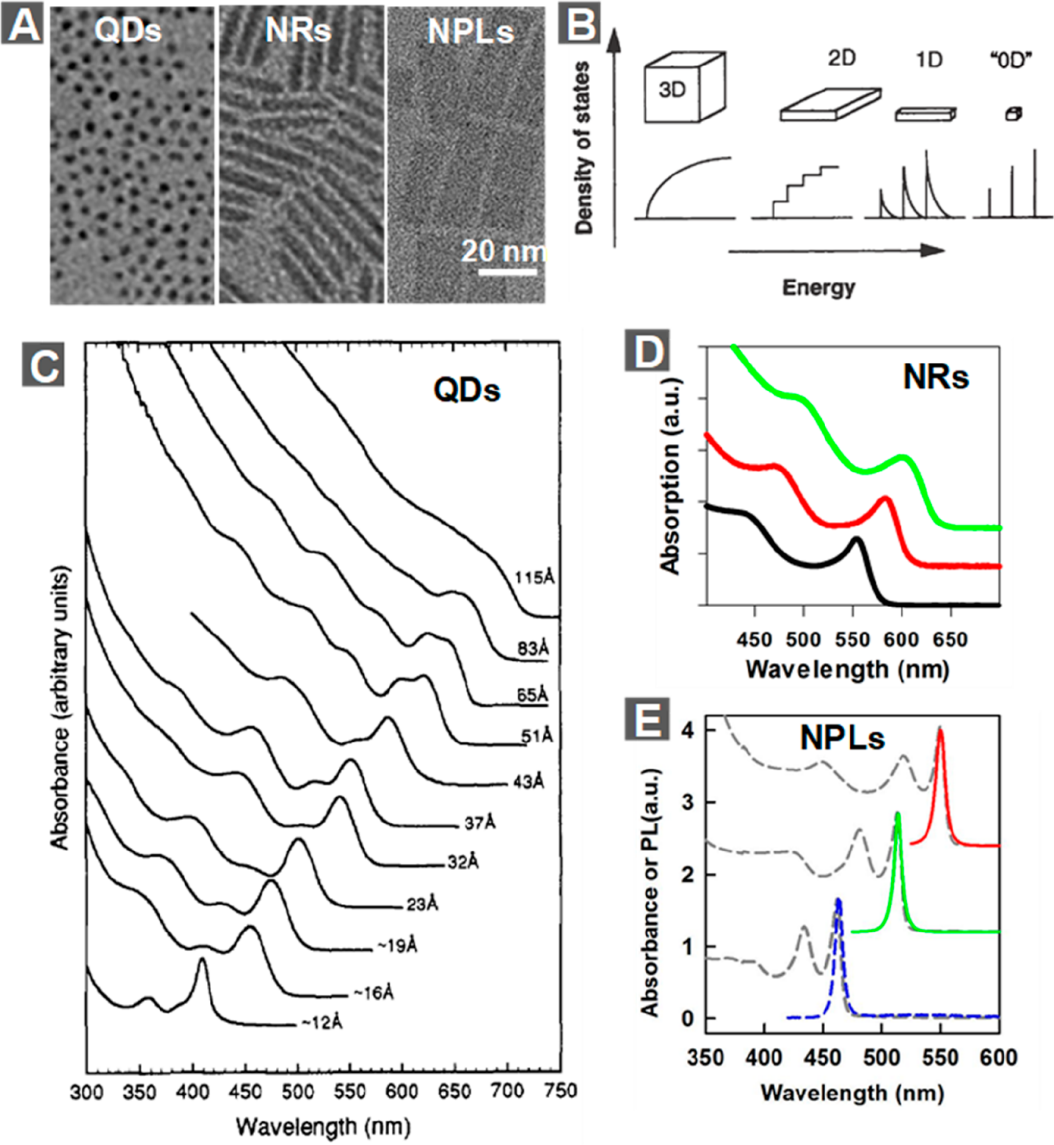
0D, 1D, and 2D nanocrystals. (A) Representative transmission electron microscopy (TEM) images of colloidal 0D, 1D, and 2D CdSe nanocrystals (NCs), which are also termed quantum dots (QDs), nanorods (NRs), and nanoplatelets (NPLs), respectively. (B) Schematic plots of density of states (DOS) as a function of energy for 3D (bulk), 2D, 1D, and 0D systems. (C–E) Static absorption spectra of (C) a series of CdSe QDs with various diameters from 1.2 to 11.5 nm, (D) CdSe NRs with three different diameters, and (E) CdSe NPLs with three different thicknesses. The thickness-dependent photoluminescence (PL) spectra of NPLs are also plotted in (E). (B) Adapted from ref (75). Copyright 1996 American Association for the Advancement of Science. (C) Adapted from ref (8). Copyright 1993 American Chemical Society.

The intrinsic electronic properties of bulk semiconductor crystals are determined by their chemical composition and lattice structure. When the crystal is small enough, its energy levels can also be tuned by its size through the quantum confinement effect.^1−4^ The effect occurs when the crystal dimension is smaller than the exciton Bohr radius, which is usually in the scale of a few to tens of nanometers.^5−7^ These quantum-confined nanocrystals (NCs) can be classified as zero-dimensional (0D) quantum dots (QDs), one-dimensional (1D) nanorods (NRs), and two-dimensional (2D) nanoplatelets (NPLs), with their exciton motions confined in three, two, and one dimensions, respectively (Figure 1A).

Different from the bulk materials, the band gap absorption and emission energy of quantum-confined NCs can be tuned over a wide range by changing the size of the quantum-confined dimensions. Taking CdSe QDs as an example, their size-tunable band gap range from 1.8 to 3.0 eV.^8^ Semiconductor NCs also have a high surface area and a much larger absorption coefficient (typically in the range of ∼10^5^–10^7^ cm^–1^ M^–1^) than molecules.^9−13^ The spatial locations of the conduction band electron and valence band hole can also be controlled by forming NC heterostructures composed of two or more components to enable novel exciton and charge separation properties.^14^ Finally, quantum-confined NCs have also been shown to facilitate multiple exciton generation, in which one high-energy absorbed photon can lead to the generation of two or more lower energy excitons. These features combined to make quantum-confined NCs a unique class of emerging materials for solar energy conversion.^15−21^ Both the solar-to-current conversion efficiency of QD solar cells^22^ and the solar-to-hydrogen conversion efficiency of QD-based photoelectrochemical cells^23^ have been reported to exceed 100%.

Since the initial discovery, various properties of quantum-confined nanocrystals and their applications have been extensively studied in the last 40 years, and the increasing recognition of the importance of these materials has led to the 2023 Nobel Prize in Chemistry to Moungi Bawendi, Louis Brus, and Alexei Ekimov for their pioneering contributions to the discovery and synthesis of quantum dots.^1−4,8^ This Review focuses on the applications of quantum-confined nanocrystals in solar energy conversion and the key process essential to these applications, *i.e.*, charge transfer to and from NCs. Optical excitation of NCs generates bound electron–hole pairs (or excitons) in semiconductor NCs, which can be dissociated to form separated electrons and holes to generate photocurrent and conduct redox reactions in photovoltaic and photosynthetic cells, respectively.^24^ The studies of charge transfer from NCs trace back to the 1980s when Brus and co-workers measured electron transfer from CdS QDs to methyl viologen via transient Raman spectroscopy^25,26^ and Kamat and co-workers studied electron transfer from CdS QDs to methylene blue using nanosecond laser flash photolysis and microwave absorption techniques.^27^ Although much of the research in this field in the late 1980s and 1990s focused on synthesis, emission properties, spectroscopic characterization, and photophysics of quantum dots,^8,28−33^ charge transfer from QDs was studied in the pioneering works by El-Sayed and co-workers^34^ and Zhang and co-workers^35^ using sub-picosecond transient absorption (TA) spectroscopy. Interest in the time-resolved study of charge transfer processes from QDs has grown considerably since then; Kamat and co-workers reported charge transfer from QDs to TiO_2_ nanoparticles in QD solar cells,^36−40^ Nozik and co-workers studied electron and hole transfer from InP QDs and their heterostructures,^41−43^ Klimov and co-workers demonstrated a new approach to the sensitization of Ru complexes via charge transfer from CdSe QDs,^44^ Lian and co-workers reported ultrafast interfacial electron and hole transfer from QDs to molecular acceptors by TA and single-particle photoluminescence (PL) decay,^45−48^ and Alivisatos reported electron transfer from CdSe/CdS NRs to Pt tips and how it affects the light-driven H_2_ generation performance of this semiconductor–metal heterostructure.^49^ These early works are followed by an explosion of detailed studies of charge transfer from quantum-confined QDs, NRs, and NPLs and their applications of photocatalysis and solar energy conversion.

Advances of charge transfer to and from NCs have been covered in some review articles. Early reviews focus on photochemistry and redox reactions on the surface of non-quantum-confined colloidal nanocrystals and nanocrystalline thin films.^50,51^ Interfacial charge transfer in donor–bridge–acceptor systems on nanoparticles and bulk metals^52^ and in solar energy conversion systems, including QD-sensitized solar cells,^16,53,54^ has also been reviewed. Charge transfer dynamics from quantum-confined NCs to molecular acceptors,^55−57^ redox enzymes,^58^ and 2D transition metal dichalcogenides^59^ have been subjects of more recent review articles, including a comprehensive Review published in this journal.^55^ Advances in the use of quantum-confined nanocrystals in photocatalytic reactions, including QDs,^60,61^ NRs,^62,63^ and NPLs,^64^ have also been reviewed. Most of these review articles focus on charge transfer from one nanocrystal morphology or type of electron acceptors. In this Review, we aim to provide a comprehensive review of charge transfer processes from 0D, 1D, and 2D quantum-confined nanocrystals, focusing on their dependence on the nanocrystal dimensionality. This Review will not cover triplet energy transfer from QDs to molecules,^65,66^ and charge transfer from perovskite NCs,^67−70^ which have received intense interest in recent years. Significant advances have also been made in the field of using microcrystals for photocatalysis,^71−73^ which is not covered in this article.

In this Review, we first introduce the electronic structure and optical properties of 0D-2D NCs in section 2, which is followed by a discussion of the recent progresses in understanding wave function engineering and carrier dynamics of 0D–2D NC heterostructures in section 3, single carrier (electron and hole) transfer from NCs in section 4, and multiple exciton generation and dissociation in section 5. In section 6, we discuss the design and performance of NC-based photocatalytic systems, with a focus on charge transfer kinetics. At last, we conclude with a summary of key advances and an outlook for future directions.

## 2 Electronic and Optical Properties of 0D, 1D, and 2D Nanocrystals

### 2.1 Dimensionality and Size Effects in 0D, 1D, and 2D Nanocrystals

The electronic structure and associated optical spectra of low-dimensional nanocrystals (NCs) can be qualitatively understood by considering two important effects in nanoscience: the dimensionality effect^74^ and the size effect.^2^ Dimensionality affects the density of states (DOS) of semiconductors, as qualitatively illustrated in Figure 1B. For a 3D (*i.e*., bulk) semiconductor, the DOS increases as a function of the square root of excessive energy above the band edge.^75^ In 2D materials, the DOS increases with energy in a step-like function form. In 1D materials, the DOS exhibits unique Van Hove singularities at the band edges and decreases as a function of the square root of excessive energy above the band edge. Finally, for 0D QDs, the DOS evolves into atomic-like discrete lines. The size effect is more specifically termed the quantum confinement effect. When the size of the NC is smaller than the characteristic length scale of excitons (the so-called exciton Bohr radius), the exciton energy is determined by the physical size of the nanocrystal.^28,76^ This effect can be approximated by a quantum mechanical “particle-in-a-box” model, which predicts that the transition energy (or the bandgap) of the nanocrystal scales with 1/*L*^2^, with *L* being the length of quantum-confined dimensions (diameter of the 0D QDs and 1D NRs and the thickness of 2D quantum wells).^77^

The simple dimensionality and size effect considerations can capture some essences of the optical spectra of colloidal NCs. Figure 1C presents the size-dependent absorption spectra of colloidal CdSe QDs synthesized using a hot-injection method.^8^ The optical gap of the QDs, as determined from the energy of the first transition peak, increases by ∼1.1 eV when the diameter is reduced from 11.5 to 1.2 nm, which is a direct demonstration of the quantum confinement effect. For NRs and NPLs, quantum confinement only exists in two and one dimensions, respectively, along the diameter and thickness directions. As such, tuning the rod diameter and platelet thickness can change the optical gaps of NRs (Figure 1D)^20,78,79^ and NPLs (Figure 1E),^80,81^ respectively. However, the absorption spectra of QDs, NRs, and NPLs all deviate significantly from their respective DOS spectra. The deviation results from the effects of nonidealized shapes and heterogeneous distribution of sizes, as well as the oversimplification of the realistic band structure of semiconductors and, for NR and NPLs, the neglect of electron–hole Coulomb interactions and their self-interactions (image charge effect).^74,82,83^ In the following, we review a higher-level theory that includes these factors and hence can almost quantitatively reproduce the optical spectra of low-dimensional NCs. Specifically, for QDs, because the confinement energy is much higher than electron–hole binding energy, the latter can be treated as a first-order perturbation;^4,77^ additionally, because the charge distributions of electron and hole virtually compensate each other within the QD, the image charge effect can be ignored.^82^ For NRs and NPLs, however, the electron and hole are at a distance larger than the diameter of the NR or the thickness of the NPL and interact predominantly through the surrounding medium, which usually has a smaller dielectric constant than the semiconductor itself. This strongly enhances the electron–hole interaction as well as their self-interactions, leading to sizable bandgap renormalization and the formation of strongly bound excitons whose optical features dominate the absorption and PL spectra of NRs and NPLs (see the sharp features on Figure 1D and E).^81−83^

### 2.2 Multiband Effective Mass Approximation

**Figure 2:**
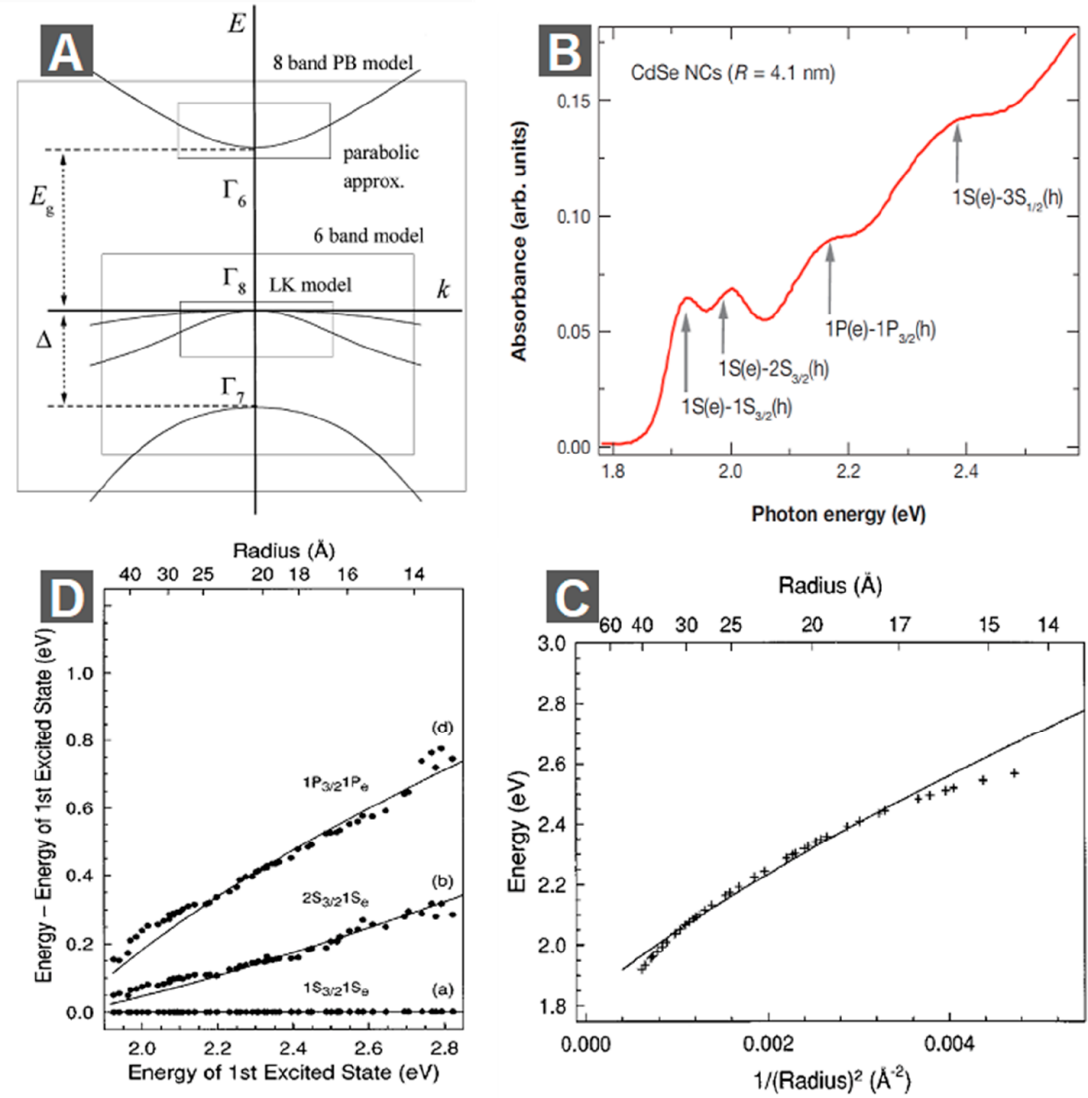
Multiband effective mass approximation (EMA) models. (A) A typical band structure for bulk semiconductors with a zinc blende lattice structure near the Γ-point of the Brillouin zone. The boxes indicate the applicability of various EMA models used for the calculation of electron and hole energy levels and wave functions. (B) Absorption spectrum of CdSe NCs with a mean radius of 4.1 nm. Some of the well-resolved optical transitions are marked by arrows. (C) Energy of the first excited state (1S_3/2_1S_e_) absorption (black crosses) plotted as a function of 1/radius^2^ for CdSe QDs and its comparison with EMA calculation results (black solid line). (D) Energy of higher-lying excited states (2S_3/2_1S_e_ and 1P_3/2_1P_e_; black squares) with respect to that of the first excited state (1S_3/2_1S_e_) plotted as a function of the first excited state transition energy. The black solid lines are EMA calculation results. (A) Adapted from ref (77). Copyright 2000 Annual Reviews. (B) Adapted from ref (92). Copyright 2007 Annual Review. (C and D) Adapted from ref (89). Copyright 1996 American Physical Society.

For quantum-confined NCs, multiband effective mass approximation (EMA) is often employed to obtain a quantitative picture of the electronic states,^77,84^ although plane-wave semiempirical pseudopotential methods have also been used.^85−87^ Within the EMA framework, the wave functions of carriers comprise the envelope wave function part and the Bloch part, which describe carrier motions in the quantum confinement potential and in the rapidly oscillating lattice potential, respectively.^88^ The applicability of various EMA models is determined by the complexity of band structures in various semiconductors.^77^ One of the typical band structures for semiconductors having zinc blende lattice symmetry (such as CdS, CdSe, CdTe, GaAs, InAs, *etc.*) is shown in Figure 2A.^77^ The conduction band (CB) shows a near-parabolic band at the band edge, whereas the valence band (VB) is much more complicated with a heavy-hole band, a light-hole band, and a spin–orbit split-off band.

Efros and co-workers have shown that, for large band gap semiconductors such as CdSe, the CB is sufficiently separated from the VB in energy such that the calculation of electron levels and wave functions near the band edge can be approximated by a single parabolic band EMA model and the calculation for holes near the band edge can be performed under the so-called Luttinger–Kohn (LK) model, which accounts for the light- and heavy-hole bands only (Figure 2A). For holes of higher excitation energy, however, the spin–orbit split-off band must also be included using the so-called six-band model.^63,89^ For narrow band gap semiconductors such as InAs, the mixture between CB and VB needs to be included, which is described by the eight-band Pidgeon–Brown (PB) model.^90,91^ In the following, we briefly introduce the EMA models for CdSe NCs that treat electrons within the single parabolic band approximation and holes within the six-band approximation, as CdSe is arguably the most prototypical material for quantum-confined NCs. For the calculation of narrow band gap semiconductor nanocrystals such as InAs and PbSe, the readers are referred to other references.^71,72^

#### 2.2.1 EMA for QDs

Using the simple single parabolic band EMA approximation for electrons in CdSe QDs, the solved envelope wave function is the product of a spherical harmonic and a spherical Bessel function. The energy levels are labeled by their angular momentum *L*_*e*_ (with *L*_*e*_ = 0, 1, 2, 3... called *S*, *P*, *D*, *F*... respectively) and radial quantum number *n*_e_.^89^ The first excited state for the electron is the 1S_e_ level. Because the valence band of CdSe primarily arises from p atomic orbitals, it has an inherent sixfold degeneracy near the band edge (*k* = 0). The spin–orbit coupling splits this degeneracy into a fourfold degenerate *J* = 3/2 band and a twofold degenerate *J* = 1/2 band. *J* is the total unit-cell angular momentum *J* = *l* + *s*, with *l* and *s* being the orbit and spin angular momentums, respectively. The *J* = 3/2 band is further split into the *J*_*m*_ = ± 3/2 heavy-hole band and the *J*_*m*_ = ± 1/2 light-hole band at *k* > 0. Taking into consideration this complex band structure (Figure 2A) in the six-band EMA model, the solution to a spherical confinement potential shows significant mixing between the bulk valence band levels. As a result, neither the total unit-cell angular momentum *J* nor the envelop angular momentum *L*_*h*_ is a good quantum number. Rather, one needs to introduce the total angular momentum *F*, where *F* = *J* + *L*_*h*_. QD hole states are commonly labeled as *n*_*h*_*L*_*F*_*.* Note that because of the mixing QD hole states nominally labeled as *L*_*h*_ have contributions from both *L*_*h*_ and *L*_*h*_ + 2 spherical harmonics (the so-called “S-D mixing”).^88,89^ For example, the first excited hole state 1S_3/2_ contains three hole components: (*F* = 3/2, *J* = 3/2, *L*_*h*_*=* 0), (*F* = 3/2, *J* = 3/2, *L*_*h*_*=* 2), and (*F* = 3/2, *J* = 1/2, *L*_*h*_*=* 2). The total QD wave function is the product of the individual electron and hole components. The optical selection rules are calculated from the overlap integral between electron and hole envelop functions, which should be *Δn* = 0 and *ΔL = 0* in the simplest case. In reality, however, this selection rule does not apply due to the hole state mixing; for example, transitions from both S and D hole states into S electron states are allowed.

This six-band EMA model captures almost all the important features in the electronic structure of QDs. The absorption spectrum of a R = 4.1 nm CdSe QD sample in Figure 2B clearly shows the features of optically allowed electron–hole pair states, with the three lowest energy states being 1S_e_1S_3/2_, 1S_e_2S_3/2_, and 1P_e_1P_3/2_, consistent with theoretical calculations.^92^Figure 2C compares the experimentally measured and theoretically calculated size-dependent energy for the band edge 1S_e_1S_3/2_ transition, showing good agreement except for very small and large size QDs.^89^ Note that in order to calculate optical transition energies the electron–hole Coulomb interaction needs to be accounted for. For QDs with strong quantum confinement, this binding energy can be treated as a first-order perturbation, and for spherical QDs it is calculated to *E*_*b*_ = −1.8*e*^2^/(*εr*), with ε and *r* being the high-frequency QD dielectric constant and radius, respectively. In addition to the band edge transition, the experimentally measured size-dependent energy difference between 1S_e_2S_3/2_ (or 1P_e_1P_3/2_) and 1S_e_1S_3/2_ transitions (Figure 2D) can also be reproduced from the theory.^89^ Overall, results show that the six-band EMA model allows for a quantitative description of the electronic and optical properties of CdSe QDs.

#### 2.2.2 EMA for NRs and NPLs

EMA models have also been applied to calculate the electronic structures of 1D NRs and 2D NPLs.^81,82,93,94^ Here we briefly review the results on CdSe NRs and NPLs. The basic idea is to treat the dimensions with and without quantum confinement separately using the adiabatic approximation, as the carrier motions along the quantum-confined direction(s) are much faster than those along the unconfined direction(s).^82^ For the calculation of CdSe NRs, Efros and co-workers treat them as ellipsoids with major semiaxis length *b* (the NR axis) much larger than the minor semiaxis length *a* (the NR radius).^82^ The quantum-confined fast motion in the radial direction is first solved using the EMA models and then the axial motion is treated by averaging the position over the fast radial motion.^82^ Similar to QDs,^29,77,84,95^ the excited states of electrons and holes in the radial direction are calculated using the single-band and six-band EMA models, respectively. A distinction from QDs is that, because of symmetry of NRs, electron states are characterized by the angular momentum projection (*m*) on the NR axis and labeled as *n*Σ_*e*_, *n*Π_*e*_... for *|m|* = 0, 1... and *n* is the number of the level for the given symmetry. Due to the mixing effect in the valence band, hole states are characterized by the total angular momentum projection (*j*_*z*_) on the NR axis: *j*_*z*_ = *m* + *J*_*z*_, where *J*_*z*_ is the projection of the hole spin on the NR axis (*J*_*z*_ = ±1/2, ±3/2...). These states are labeled as *n*Σ_*|jz|*_, *n*Π_*|jz|*_..., respectively.

**Figure 3:**
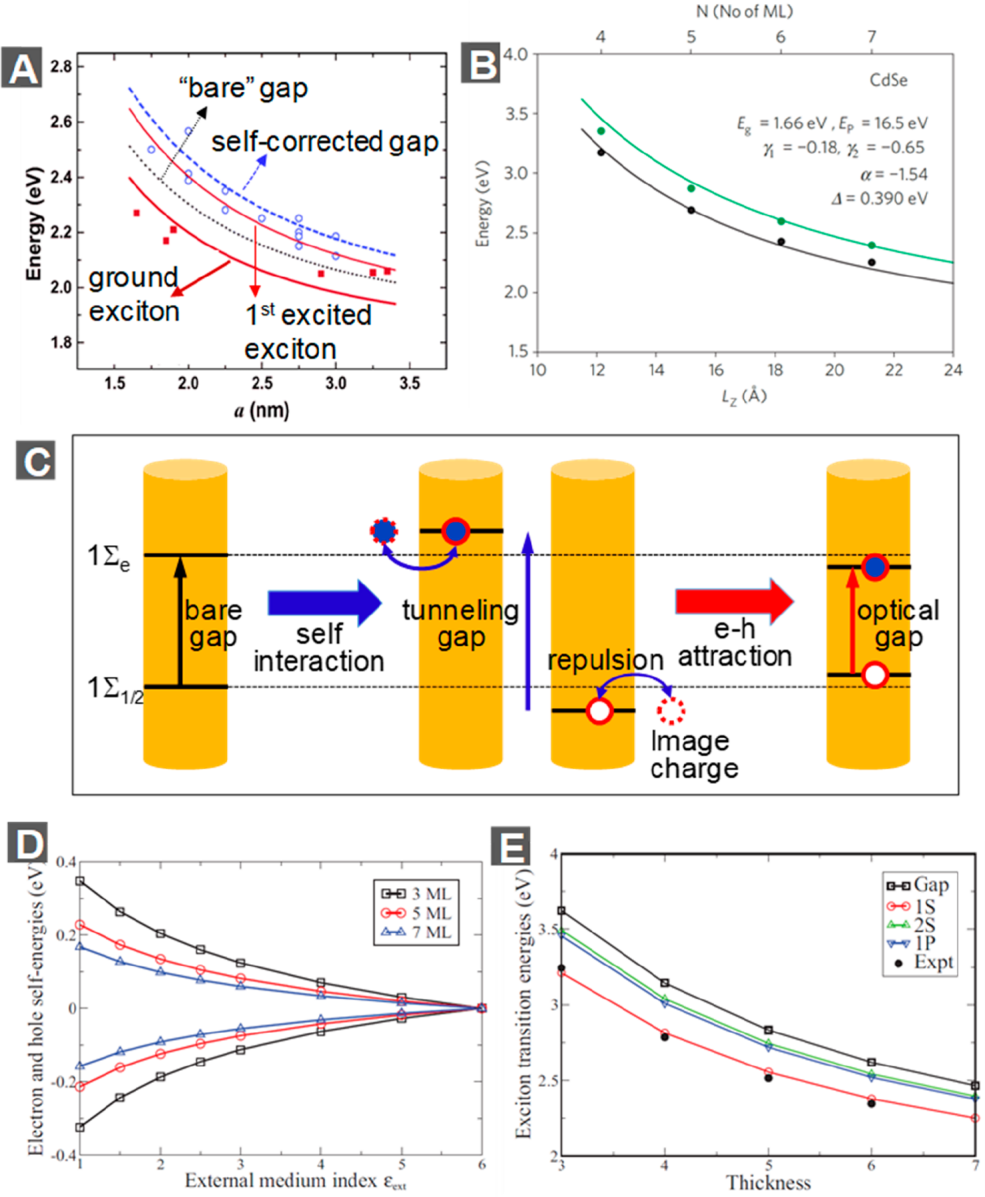
Dielectric confinement effect in NRs and NPLs. (A) Diameter-dependence of the tunneling and optical energy gap in CdSe NRs. Dotted and dashed lines show bare and self-interaction-corrected energy gaps between the 1Σ_1/2_ hole and the 1Σ_*e*_ electron sub-band. Two solid lines show the optical transition energy for the first two optically active 1D excitons. Experimental data for the tunneling and optical gap measured in CdSe NRs from ref (96) are shown by empty circles and filled squares. (B) Thickness-dependent energies of the electron/light-hole (green circles) and electron/heavy-hole (black circles) optical transitions and their comparison with EMA calculations (solid lines). (C) Schematic illustration of the “bare”, tunneling, and optical gaps and dielectric-confinement-induced electron (hole)–image charge-repulsive self-interaction and enhanced electron–hole attraction. (D) Single-particle self-interaction energies as a function of an external medium dielectric constant (ε_*ext*_), with the CdSe dielectric constant fixed at 6, for CdSe NPLs with thicknesses of 3 (black squares), 5 (red circles), and 7 (blue triangles) monolayers calculated using an advanced tight-binding model. (E) Electron/heavy-hole transitions energies calculated for 1S (red circles), 2*S* (green triangles), 1P (blue triangles), and ∞*S* (approaching optical gap; black squares) as a function of the NPL thickness using ε_ext_ = 2. (A) Adapted with permission from ref (82). Copyright 2004 American Chemical Society. (B) Adapted with permission from ref (81). Copyright 2011 Springer Nature. (C) Adapted with permission from ref (20). Copyright 2016 Royal Society of Chemistry. (D and E) Adapted with permission from ref (83). Copyright 2014 American Physical Society.

Based on the optically allowed transitions between these quantum-confined electron and hole levels, Efros and co-workers first calculated the diameter-dependent “bare” gaps of CdSe NRs (Figure 3A), which are defined as the energy difference between the band edge electron and hole states (without the electron–hole binding energy) and should match the gap measured by STM tunneling current experiments. However, Figure 3A shows that the calculated diameter-dependent “bare” gaps for NRs deviate significantly from the tunneling data (by ∼100–200 meV). This is a consequence of bandgap renormalization due to the dielectric confinement effect that strongly enhances charge self-interactions in 1D NRs. As mentioned above, the dielectric confinement effect also strongly enhances the electron–hole binding energy. It is interesting to note that the increases in the charge self-interaction energy and the electron–hole binding energy almost exactly compensate each other. As a result, the exciton transition energy or the optical gap remains largely unaffected by the dielectric confinement effect. For example, Efros and co-workers calculated the electronic structure of 2D CdSe NPLs using an eight-band EMA model.^81^ The eight-band EMA model was used for improved accuracy, although six-band EMA should, in principle, be sufficient for CdSe NPLs. In their calculation, they ignored the contribution of the self-interaction terms as well as the increase of the binding energy and simply used a 2D limit for binding energy: *E*_*b*_ = 4*E*_ex_, where *E*_ex_ is the exciton binding energy in bulk semiconductors. As shown in Figure 3B, this calculation reproduces the thickness-dependent optical gaps of CdSe NPLs. However, if one needs information on the bandgap renormalization effect and electron–hole binding energy in 1D and 2D nanocrystals, the dielectric confinement effect should be calculated, as we review below.

### 2.3 Dielectric Confinement Effect in 1D NRs and 2D NPLs

The dielectric confinement effect arises from the difference between the dielectric constants of 1D and 2D semiconductors (*ε*_s_) and their external medium (*ε*_ext_).^97^ First, it enhances the (repulsive) self-interactions of electrons and holes with their image charges, thus leading to a sizable increase in the quasi-particle gap (bandgap renormalization),^98^ as illustrated in Figure 3C. On the other hand, it also strongly enhances the electron–hole attractive interactions, resulting in a large exciton binding energy that lowers the optical gap considerably from the quasi-particle gap. In their calculation for CdSe NRs, Efros and co-workers calculated Coulomb interactions enhanced by the dielectric contrast effect using *U*(***r***_e_, ***r***_h_) = −*e*^2^/(*ε*_s_|***r***_e_ – ***r***_h_ |) – *eV*(***r***_e_, ***r***_h_) + *eV*(***r***_e_, ***r***_e_) + *eV*(***r***_h_, ***r***_h_), where the first and second terms are electron–hole interaction potentials through inside and outside the NR, respectively, and the last two terms are electron and hole self-interaction potentials.^82^ By averaging *U*(**r**_e_*,***r**_h_) over the electron and hole wave functions in the radial direction, they obtained a 1D potential profile of electron–hole (e-h) Coulomb interaction and electron (hole) self-interaction corrections to their quantum-confined energy levels. As shown in Figure 3A, after adding the self-interaction corrections to the energy difference between the band edge electron and hole states, the “bare” gaps increase by ∼100–200 meV and become consistent with the experimentally measured tunneling gaps. The two lowest optically allowed excitonic transitions obtained by solving the 1D e-h potential are shown in Figure 3A. The diameter-dependent energies for the ground excitonic states match the experimental optical gaps well. Also, it can be seen that the energy of the ground excitonic state is lower than the tunneling gap by ∼150 meV, which reflects the large exciton binding energy in CdSe NRs.

Similar calculations have been performed on 2D CdSe NPLs.^83,99^Figure 3D shows the calculated electron and hole self-interaction energies as a function of the dielectric constant of the external medium *ε*_ext_ for NPLs with different thicknesses.^83^ The self-interaction energies increase considerably as *ε*_ext_ decreases, and this effect is more obvious for thinner CdSe NPLs, indicating that the dielectric confinement effect is stronger for thinner NPLs and for the situation with a smaller *ε*_ext_. For the 3 monolayer (ML) CdSe NPLs, when *ε*_ext_ is 1 (*i.e.*, in the air), electron and hole self-interaction energies reach ∼300 meV and the total bandgap renormalization reaches ∼600 meV. Figure 3E shows the calculated thickness-dependent electron and hole binding energies for *ε*_ext_ = 2, which decrease from ∼100 meV for 3 ML NPLs to ∼50 meV for 7 ML NPLs.^83^ Similar dielectric-contrast-enhanced strongly bound excitons have been observed in many other 1D and 2D material, such as carbon nanotubes, graphene, and monolayer transition metal dichalcogenides.^74,98,100−104^

### 2.4 Exciton Fine Structure and Lifetime in 0D, 1D, and 2D Nanocrystals

Besides the spectral properties, it is also important to understand the dynamic properties of quantum-confined NCs, as they dictate the time window available for charge extractions from these NCs. The electron–hole recombination dynamics in NCs are determined by the trap states and the fine-structures. The trapping process is usually much faster than the radiative recombination process; for example, the hole trapping in CdSe QDs can be as fast as ∼10 fs.^105^ Therefore, in QD ensembles, QDs with traps are dominated by the nonradiative recombination between the CB edge electron and trapped hole, while QDs without traps undergo radiative recombination between band edge carriers with their dynamic properties controlled by the fine structure of their band edge excitons. In 1D NRs and 2D NPLs, other factors such as giant oscillator strength transition effect further modify the band edge exciton radiative lifetime in addition to the fine structure.

**Figure 4:**
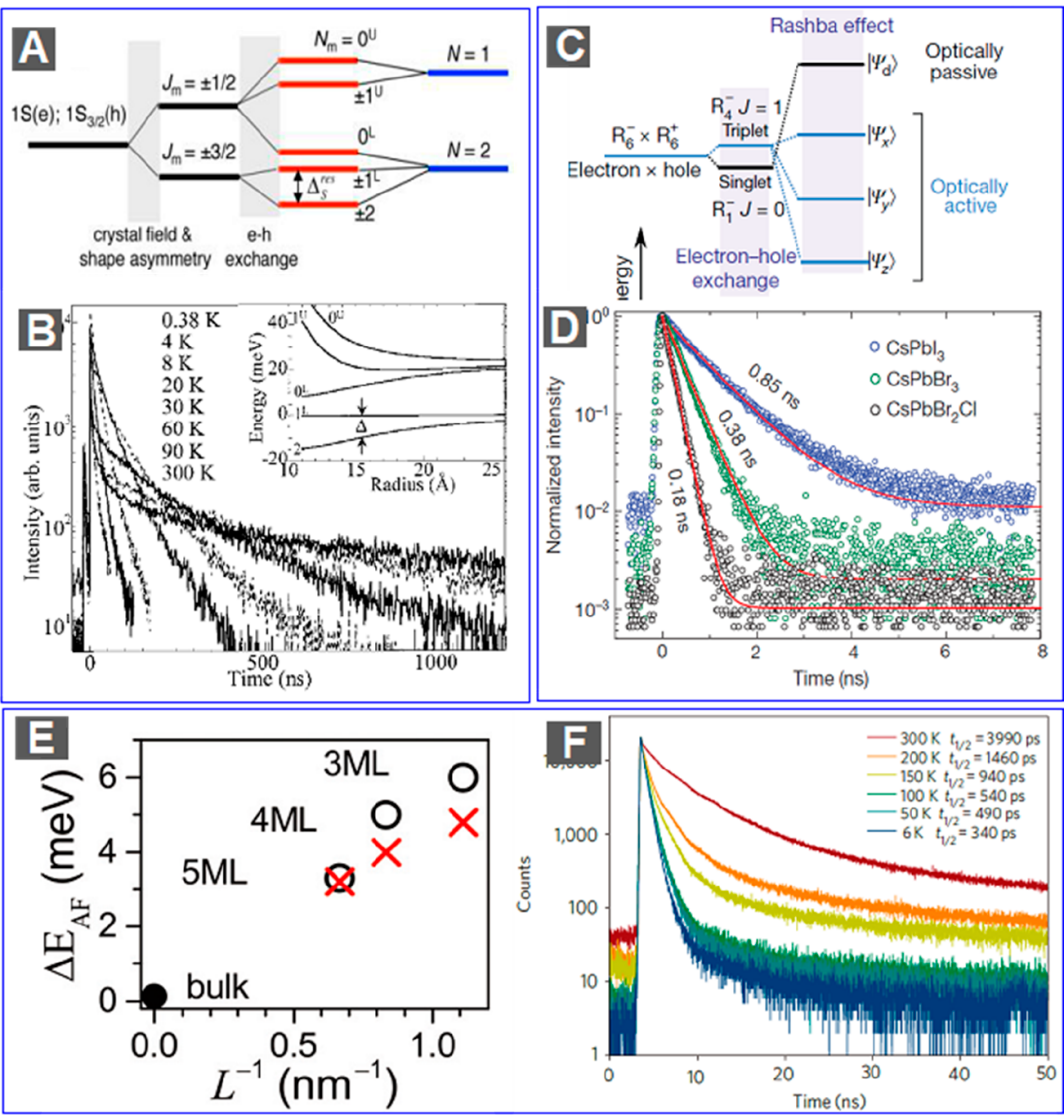
Exciton fine structure and lifetime. (A) Scheme of the fine structure of the band edge 1S_e_–1S_3/2_ excitons of CdSe QDs. The fine structure splitting is mainly introduced by anisotropic effects and e-h exchange interactions. (B) Time-resolved PL decay curves measured for typical CdSe QDs at indicated temperatures. (C) Scheme of the fine structure of the band edge excitons of perovskite NCs. Here the fine structure splitting is mainly introduced by e-h exchange interactions and the Rashba effect under orthorhombic symmetry. (D) Time-resolved PL decay curves measured for single CsPbI_3_, CsPbBr_3_, and CsPbBr_2_Cl perovskite NCs at cryogenic temperatures. (E) Bright–dark splitting of CdSe NPLs (Δ*E*_AF_) as a function of the reciprocal of NPL thickness (1/*L*). Crosses and circles represent results measured by PL line narrowing and temperature-dependent time-resolved PL, respectively. (F) Time-dependent PL decay curves measured for typical CdSe NPLs at indicated temperatures. (A and B) Adapted with permission from ref (88). Copyright 2016 American Chemical Society. (C and D) Adapted with permission from ref (107). Copyright 2018 Springer Nature. (E) Adapted with permission from ref (108). Copyright 2018 Royal Society of Chemistry. (F) Adapted with permission from ref (81). Copyright 2011 Springer Nature.

The fine structure of the band edge 1S_e_–1S_3/2_ excitons of CdSe QDs, as developed by Efros, Bawendi, and co-workers,^28,29^ is schematically shown in Figure 4A. The splitting of the fine structure is mainly induced by the combined effect of short- and long-range electron–hole exchange interactions and anisotropies associated with the crystal field and nanocrystal shape asymmetry.^106^ The exchange interaction is typically weak (at most a few meVs) for bulk inorganic semiconductors and thus is often ignored, unlike the strong interaction that leads to large singlet–triplet splitting in organic materials. In NCs, however, the quantum-confinement-induced strong electron–hole wave function overlap enhances the exchange interaction energy up to tens of meVs. As a result, one should consider a correlated band edge exciton, rather than independent electron and hole, with a total angular momentum of *N* (*N* = 1 or 2). *N* = 1 corresponds to a high-energy optically active exciton (so-called “bright” exciton), whereas *N* = 2 is a lower-energy optically passive exciton (so-called “dark” exciton), as a photon can only carry a momentum of 1. Due to the asymmetric crystal field and NC shape, the good quantum number is the projection of the total angular momentum along the unique crystal axis (*N*_*m*_) and hence the *N* = 1 and 2 states are further split into two manifolds of states (Figure 4A). The new lowest-energy state is still a “dark” exciton characterized by *N*_*m*_ = ±2, and the next higher-energy state is a “bright” exciton with *N*_*m*_ = ±1.

The splitting between the “dark” and “bright” excitonic states is in the range of a few to 10 meV, with smaller QDs having larger splitting due to stronger electron–hole exchange interactions. This splitting controls the temperature-dependent radiative recombination dynamics of band edge excitons. As shown in Figure 4B, at very low temperatures excitons primarily occupy the “dark” state and thus recombine on a μs time scale. With increasing temperatures, excitons more and more occupy the “bright state”, resulting in faster radiative recombination. At room temperature, the thermal energy (∼26 meV) is sufficiently high as compared to the dark–bright splitting energy of a few meV. As a result, excitons are approximately distributed equally between the “dark” and “bright” states, leading to a lifetime of ∼20 ns for typical CdSe QDs. This intrinsic exciton lifetime is longer than the time constants for many interfacial charge transfer from QDs and hence is often not a limiting factor for efficient charge extraction from QDs.

The band edge exciton structures of most QDs can be explained by the picture depicted above. The recently introduced lead halide perovskite nanocrystals (NCs), however, likely feature a different type of fine structure. These NCs have large extinction coefficients and exceptional light-emitting properties; as-synthesized NCs can attain emission quantum yields in the range of 50–90% without any postsynthetic treatments.^109^ To account for these properties, Efros and co-workers theoretically examined the fine structure of perovskite NCs and proposed a structure with a “bright” triplet state as the lowest-energy band edge state.^107,110^ Specifically, because the top of the valence band is contributed by Pb 6*s* and Br 4*p* atomic orbitals with an overall *s*-like symmetry, the band edge hole state has *J*_h_ = 1/2, which is different from that of CdSe QDs. The electron–hole exchange interaction mixes the angular momentum of the electron and hole states to form a *J* = 0 singlet state and a threefold degenerate *J* = 1 triplet state, with the former being “dark” and the latter “bright” (Figure 4C). The singlet state would lie below the triplet state if only considering the electron–hole exchange interaction. These perovskite NCs, however, exhibit a strong Rashba effect due to strong spin–orbital coupling and breaking of inversion symmetry. The reasons for the inversion asymmetry remain unclear but are likely related to cation positional instabilities or surface effects. The Rashba effect inverts the energetic order between the singlet and triplet states, leading to a unique fine structure featuring a lowest-energy “bright” triplet state (Figure 4C). Because of the large oscillator strength of the “bright” triplet state, radiative recombination of band edge excitons in perovskite NCs is very fast (a few ns at room temperature) and accelerates with decreasing temperatures. As shown in Figure 4D, at cryogenic temperatures, the recombination time of band edge excitons can be as fast as 0.18 ns. The fast radiative recombination in perovskite NCs, along with the unique “defect-tolerance” in these materials, is responsible for their exceptional light-emitting performances, which, on the other hand, could also limit the efficiency of charge extraction from these NCs. Nonetheless, ultrafast measurements revealed that interfacial charge transfer from these NCs could be engineered to occur on a ps time scale, thus competing effectively with exciton recombination.^111^

For fine-structures of CdSe NRs, Efros and co-workers found that the optically dark *F*_*z*_ = 0 state (the projection of the total angular momentum along the rod axis) is situated a few meV below the optically bright *F*_*z*_ = ±1 state, which is partially responsible for the linearly polarized emission from NRs in addition to the dielectric polarization effect, similar to QDs.^82^ On the other hand, different from the QDs, the order of fine structure states can depend on both the length and radius of the NR.^112,113^ For fine-structures of CdSe NPLs, Biadala and co-workers measured the PL lifetime of CdSe NPLs as a function of temperatures and applied magnetic fields.^114^ They found that the PL lifetime increases with decreasing temperatures and that at a constant temperature of 4.2 K the PL lifetime decreases with increasing applied magnetic fields. Using similar PL measurements, Shornikova and co-workers reported the splitting between the bright and dark exciton states as ∼3–6 meV for 3–5 ML CdSe NPLs (Figure 4E).^108^ All these observations are qualitatively consistent with those of 0D QDs, suggesting similar band edge fine structures. Moreover, due to the large exciton binding energy, Rydberg series of the band edge excitons in CdSe NPLs can be observed under optical measurements,^115^ similar to transition metal dichalcogenide.^100,116^ In particular, Achtstein and co-workers observed the emission from the p-state of the band edge exciton in 4 ML CdSe NPLs at low temperature (4 K) with an s- and p-state splitting of ∼30 meV and found that the relaxation rate between p- and s-state of band edge exciton strongly depends on the lateral dimension of the NPL and shows a LO-phonon bottleneck.^115^ Unlike 0D QDs, the band edge exciton recombination dynamics 2D NPLs are strongly modified by a so-called giant oscillator strength transition effect, which is induced by a coherent extension of the exciton center-of-mass motion along the unconfined dimensions at low temperature due to the suppressed exciton–phonon scattering.^81,117−119^ This effect leads to a fast radiative recombination of excitons; hence, intrinsic exciton half-lifetimes decrease to sub-ns at low temperatures (<150 K) (Figure 4F).^81,119^ Because of the fast diffusion of excitons/carriers in NRs and NPLs and the fast interfacial charge transfer from them, this lifetime window is also sufficient to enable efficient exciton dissociation.

### 2.5 Effect of Exciton Fine Structure on Transient Absorption Spectral Signatures

Ultrafast pump–probe transient absorption (TA) spectroscopy has been widely used to study the excited charge carrier dynamics in quantum-confined nanocrystals.^120,121^ The photogenerated exciton, or electron and hole, blocks the ground state excitonic transitions due to the state filling in the conduction band (CB) and valence band (VB), causing an exciton bleach (XB) signal in the TA spectrum. The bleach amplitude can be related to the exciton or electron and hole population according to the band edge exciton fine structures^29,122−125^ and can be used as a convenient probe of exciton and carrier dynamics in QDs, such as hot carrier cooling,^126,127^ electron–hole recombination,^128,129^ Auger processes,^130−132^ and charge transfer and recombination.^121,133,134^ It has been reported that both CB electron and VB hole contribute to the band edge XB signal in PbS,^135−137^ PbSe,^121,138,139^ and CsPbX_3_ (X = Cl, Br, I) QDs or nanocrystals^140−142^ due to the band edge state filling effect. However, in cadmium chalcogenide QDs, such as CdSe,^123,124,133,143−145^ CdS,^146^ and CdTe,^134,147^ the XB is dominated by CB electron state filling effect with negligible VB hole contribution. For example, selectively removing electron from the CdSe or CdS QDs leads to complete XB recovery.^148,149^ Same XB signal assignment was applied to their nanorods (NRs)^150−153^ and nanoplatelets (NPLs)^154−156^ counterparts, as well as InP^157,158^ and Cd_3_P_2_ QDs.^159^ The lack of a VB hole state filling effect was assumed to result from the high degeneracy of the hole states at VB top and/or the ultrafast hole trapping in these materials.^160,161^ It is worth noting that, however, the XB decay corresponding to ultrafast hole trapping in CdSe QDs has not been observed even in transient measurements with 10 fs time resolution.^105^

The VB hole-induced bleach signal in TA spectrum in cadmium chalcogenide QDs was not observed until recently when the hole traps were eliminated in core/shell heterostructures or surface passivation.^162−165^ Grimaldi et al. showed that the VB hole contributes to 32 ± 2% of the 1S exciton [1S_3/2_(h)–1S(e)] bleach in CdSe/CdS/ZnS core/shell/shell QDs with a photoluminescence quantum yield (PLQY) of 82%.^162^ This hole contribution was measured from the growth kinetics of the 1S XB when the hole relaxes from the 2S_3/2_ state to the 1S_3/2_ state. A fourfold degeneracy of the 1S_3/2_ hole state at the top of the VB was assumed to account for the one-third hole contribution to the exciton bleach, while the band edge exciton fine structure was not considered. In similar CdSe/CdS core/shell QDs with a PLQY of 81%, He at al. reported a hole contribution of 22 ± 1% by fitting the TA spectrum of the single hole state in QDs after selective removal of the CB electron.^163^ The authors proposed that, in the absence of hole traps, the low hole contribution to the XB signal is mainly due to the exciton fine structure, *i.e.*, the thermal distribution of hole populations in bright and dark exciton states. The hole bleach signal was later reported by Huang et al.^166^ in TA spectra of Cd-based core/shell QDs and by Brosseau et al.^167^ in two-dimensional electronic spectroscopy with high energy and time resolutions. Identification of hole-induced absorption bleach implies possible optical gain in Cd-based QDs and helps to rationalize the design of QD lasers.^162,166^ Moreover, hole-state-filling-induced TA spectra of Cd-based QDs may enable direct probing of the hole transfer process by TA spectroscopy.

## 3 Wave Function Engineering and Carrier Dynamics in 0D, 1D, and 2D Heteronanocrystals

### 3.1 Wave Function Engineering in Heteronanocrystals

**Figure 5:**
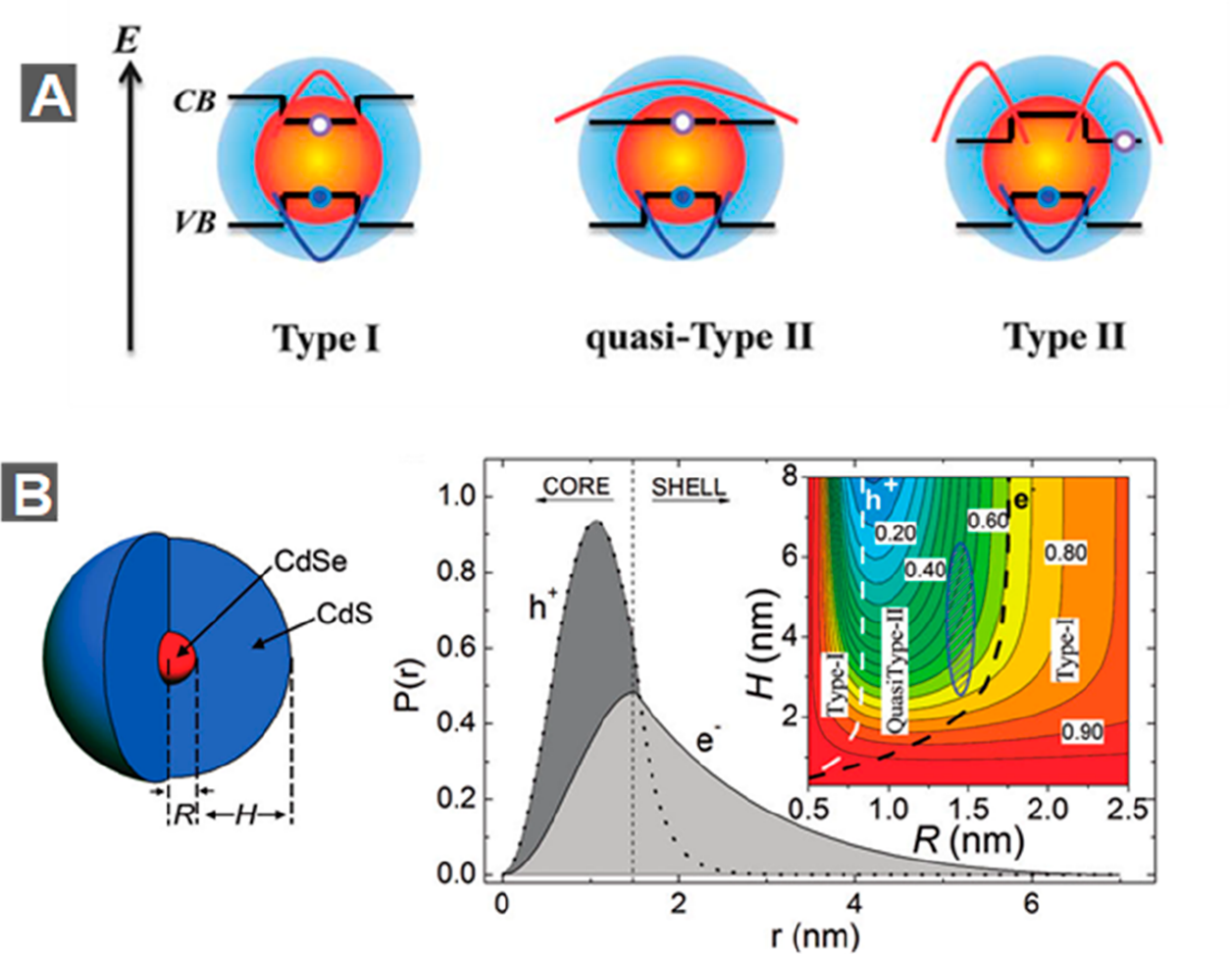
Wave function engineering in heterostructured nanocrystals. (A) Schematic depictions of the band alignments (black lines) and electron (red) and hole (blue) wave function distributions in type I, quasi-type II, and type II core/shell QDs. (B) Schematic of the “giant” NC structure where *R* is the CdSe core radius and *H* is the CdS shell thickness (left). Spatial probability distribution of the hole (dark gray area) and electron (light gray area) for an *R* = 1.5 nm and *H* = 5.0 nm core/shell QD (right). The inset shows a contour plot of the calculated electron–hole overlap integral. White and black lines are boundaries between regions of (*R*, *H*)-space that correspond to different localization regimes. (A) Adapted with permission from ref (168). Copyright 2012 Royal Society of Chemistry. (B) Adapted with permission from ref (179). Copyright 2009 American Chemical Society.

The spectral and dynamic properties of NCs can be further engineered by building heterostructured NCs such as core/shell QDs. In these heterostructures, the spatial distribution of the wave functions of electrons and holes can be tailored by the sizes and compositions of the constituent materials.^168^ This is the concept of “wavefunction engineering”. On the basis of the energy offsets of bulk conduction band (CB) and valence band (VB) edges of the constituent materials, the band alignment in semiconductor heterostructures can be classified as type I, quasi-type II, and type II, as depicted in Figure 5A. In a type I structure, both the CB and VB edges of one component are situated within the band gap of the other component, a typical example for which is CdSe/ZnS. As a result, both the lowest-energy electron and hole wave functions are primarily confined in the low band gap material. In contrast, a type II structure features a staggered band alignment with the lower energy CB and VB edges situated on different components, as exhibited in, *e.g.*, CdSe/CdTe. This type of band alignment should lead to spatial separation of electron and hole wave functions into different domains. A quasi-type II structure is the intermediate case between type I and type II structures, with the two components share the same (or similar) CB or VB edge. For example, the VB edges are similar for CdSe and ZnSe, while their CB edges have an offset of ∼0.9 eV.^169^

However, because of the quantum confinement effect strongly modifying the band edges of semiconductor NCs, it is important to note that the electronic structure of a heterostructured NC can be different from that of its bulk counterpart. To avoid this confusion, we shall use the term “band alignment” only for bulk materials and the term “electronic structure” for NCs. For example, the VB offset between bulk wurtzite CdSe and CdS is large (≥0.45 V) and the CB offset is relatively smaller (≤0.3 V),^170,171^ which is a type I band alignment. However, the electronic structure of CdSe@CdS NRs is tunable from type I (with both the electron and hole confined in CdSe) to quasi-type II (with the hole confined CdSe and the electron delocalized among CdSe and CdS) depending on the sizes of the CdSe and CdS domains.^171−178^

Figure 5B (right) shows the spatial probability distributions (proportional to the square of wave function) of the lowest-energy CB electron and VB hole in CdSe/CdS core/shell QDs with a core radius (*R*) of 1.5 nm and a shell thickness (*H*) of 5.0 nm, as calculated using EMA methods.^179^ The hole wave function is mostly confined in the core, while the electron wave function is delocalized over the whole core/shell, which is a typical signature of quasi-type II electronic structure. Calculations also show that the electron and hole wave function distributions indeed depend sensitively on the *R* and *H* parameters, as illustrated by the calculated electron–hole wave function overlap integral in Figure 5B (right inset). When the shell is very thin (small *H*), both the electron and hole can effectively tunnel into the shell regardless of the core size. As a result, the electron–hole overlap is large, corresponding to the type I regime. Alternatively, in the case of a diminishingly small core, both the electron and hole effectively spill into the shell regardless of the shell thickness, which is also a type I structure; however, this is not a practical case, as it would be very difficult to coat a shell on a diminishingly small core. For a normal core size (*R* > 1 nm), when the shell becomes thicker, a small core with strong electron confinement energy leads to a delocalized electron and a core-confined hole, which is a quasi-type II structure featuring a smaller electron–hole overlap, whereas a larger core with weaker electron confinement energy results in a core-confined electron and hole in the type I regime.

**Figure 6:**
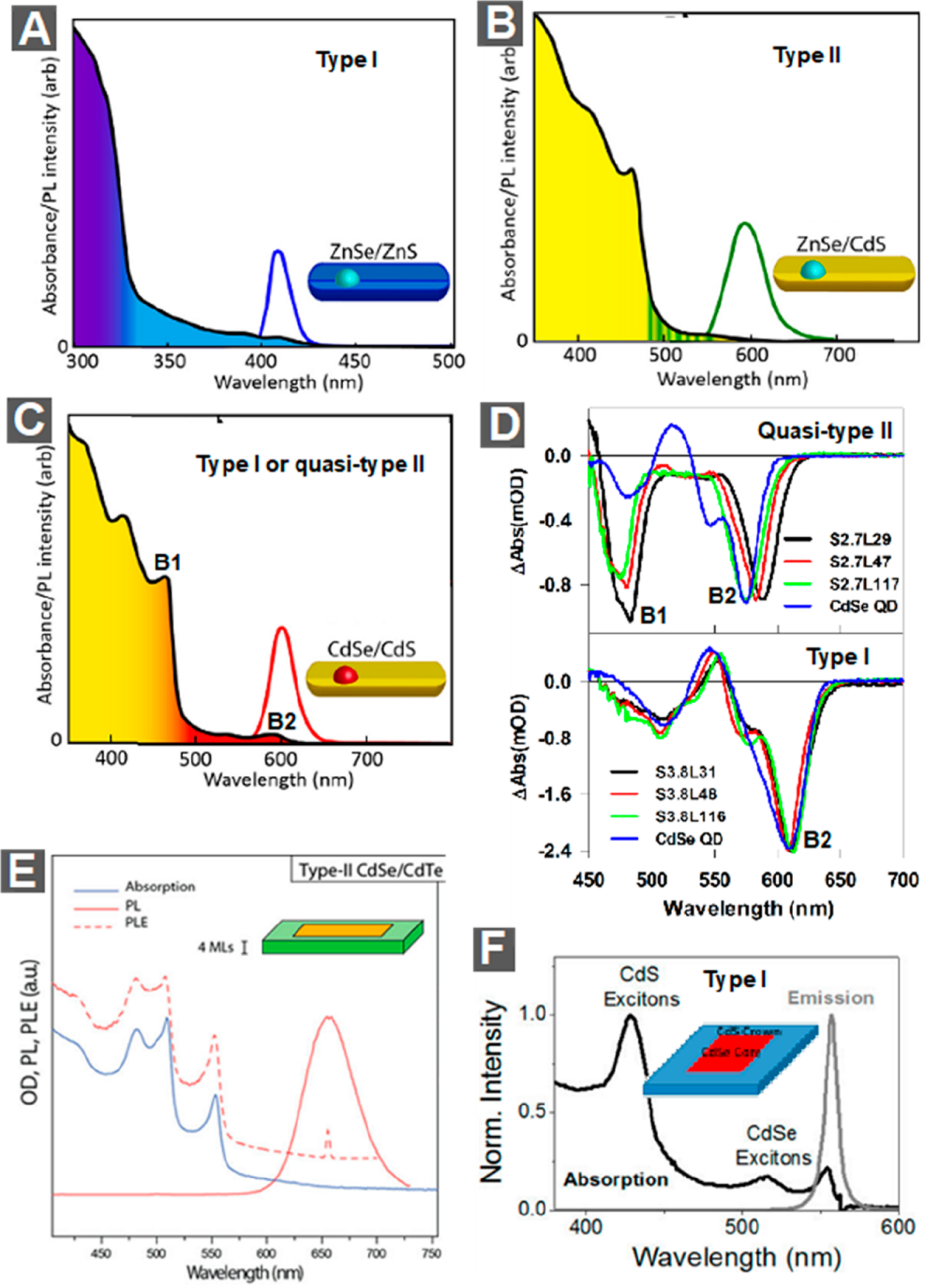
Wave function engineering in 1D hetero-NRs and 2D hetero-NPLs. (A–C) The absorption spectra (shaded regions) and PL spectra (colored lines) of (A) ZnSe@ZnS, (B) ZnSe@CdS, and (C) CdSe@CdS dot-in-rod hetero-NRs. B1 and B2 transition features on CdSe@CdS NRs are labeled. (D) Averaged transient absorption (TA) spectra of CdSe@CdS hetero-NRs with 2.7 nm (top) and 3.8 nm (bottom) diameter CdSe cores and varying rod lengths at 1–2 ps after ∼580 nm excitation. For comparison, TA spectra of corresponding CdSe QDs with similar confinement energies are also shown. (E) Absorption, PL, and PL-excitation (PLE) spectra of type II CdSe@CdTe core/crown hetero-NPLs. The inset shows the scheme of the hetero-NPL. (F) Absorption and PL spectra of type I CdSe@CdS core/crown hetero-NPLs. Inset shows the scheme of the htero-NPL. (A–C) are reproduced with permission from ref (180). Copyright 2013 Elsevier Ltd. (D) Adapted with permission from ref (177). Copyright 2015 American Chemical Society. (E) is reproduced with permission from ref (209). Copyright 2014 American Chemical Society. (F) is reproduced with permission from ref (210). Copyright 2014 American Chemical Society.

The concept of wave function engineering has also been extended to 1D hetero-NRs and 2D hetero-NPLs.^170,180−184^ For example, ZnSe@ZnS dot-in-rod hetero-NRs^185^ have a type I electronic structure (Figure 6A), whereas ZnSe@CdS hetero-NRs^186^ have a type II electronic structure (Figure 6B). Particularly interesting are the CdSe@CdS dot-in-rod hetero-NRs (Figure 6C), which can attain the same type of wave function engineering from type I to quasi-type II as CdSe@CdS core/shell QDs by simply tuning the core sizes and rod diameters (refs (49, 170−176, 181, 182, 187−206)). The absorption spectrum of CdSe@CdS NRs mainly consists of two features, B1 and B2, that are associated with the lowest-energy exciton states of the rod and the core, respectively (Figure 6C). For NRs with relatively small-size cores, the electron wave function is delocalized from the core to the rod, whereas the hole wave function is still confined in the core (*i.e.*, B1 and B2 share the same electronic level but have different hole levels), forming a quasi-type II structure. For samples with larger cores, both the lowest electron and hole levels are located in the core, forming a type I structure. Experimentally, these electronic structures can be readily probed using TA measurements.^173,196,207^ Specifically, one can selectively excite the B2 band of the core and monitor its influence on B1 band of the rod.^177^Figure 6D shows the TA spectra averaged between 1 and 2 ps following B2 excitation for NRs of various lengths with both 2.7 and 3.8 nm diameter seeds. For NRs with a 2.7 nm seed (Figure 6D, top), excitation at B2 leads to instantaneous and simultaneous formation of strong bleaches of both B2 and B1 bands. Since these bleaches are dominated by the CB electron state filling contribution,^207^ this result indicates that B1 and B2 bands share the same CB electronic level, consistent with a quasi-type II electronic structure.^174,207^ In contrast, for 3.8 nm seeded NRs (Figure 6D, bottom), excitation at B2 only generates a strong B2 bleach with negligible B1 bleach. The derivative-like feature near B1 bleach is also observed in CdSe core only QDs and can be attributed to a biexciton-interaction-induced shift of higher energy transitions in the core.^208^ Therefore, these 3.8 nm seeded NRs have a type I electronic structure.

Wave function engineered 2D NPLs have also been reported. For example, growing a CdTe NPL laterally on the edge of a CdSe NPL, or vice versa, leads to core/crown hetero-NPLs with a type II electronic structure (Figure 6E).^209,211−218^ The strongest evidence for the type II heterostructure is the appearance of an absorption tail with lower energy than both CdSe and CdTe band edge absorptions and the emission associated with this tail (Figure 6E),^209^ which is the so-called charge-transfer (CT) transition from the top of the CdTe VB to the bottom of the CdSe CB formed due to strong electronic coupling between the two epitaxially attached domains.^209,211,218^ It is also worth noting that Scholes and co-workers attributed the emission of CdSe/CdTe core/crown NPLs to trapped state emission instead of CT exciton emission, as the latter has a very weak transition strength.^219^ Type I hetero-NPLs with a CdSe core embedded in a CdS crown have also been reported (Figure 6F).^210,213,220,221^ Due to the type I electronic structure, photoexcitation energy generated in the CdS crowns can be effectively funneled in to the CdSe cores where light can be emitted.^210,221^ Owing to the exceptional light-harvesting capability of 2D structures, this funneling mechanism can leads to very high excitation densities at the CdSe cores for light-emitting or conversion applications.^213,217,222,223^

An important difference between 1D hetero-NRs (2D hetero-NPLs) and 0D core/shell QDs is that the band alignment (or electronic structure) is not the only factor that dictates the wave function distributions of the electron and hole. The strong electron–hole binding in 1D and 2D systems also strongly modifies the wave function distributions. As a result, in (quasi-)type II NRs and NPLs, for example, the electron and hole are not completely delocalized over different domains but instead are localized near the charge separation interface.^211^

**Figure 7:**
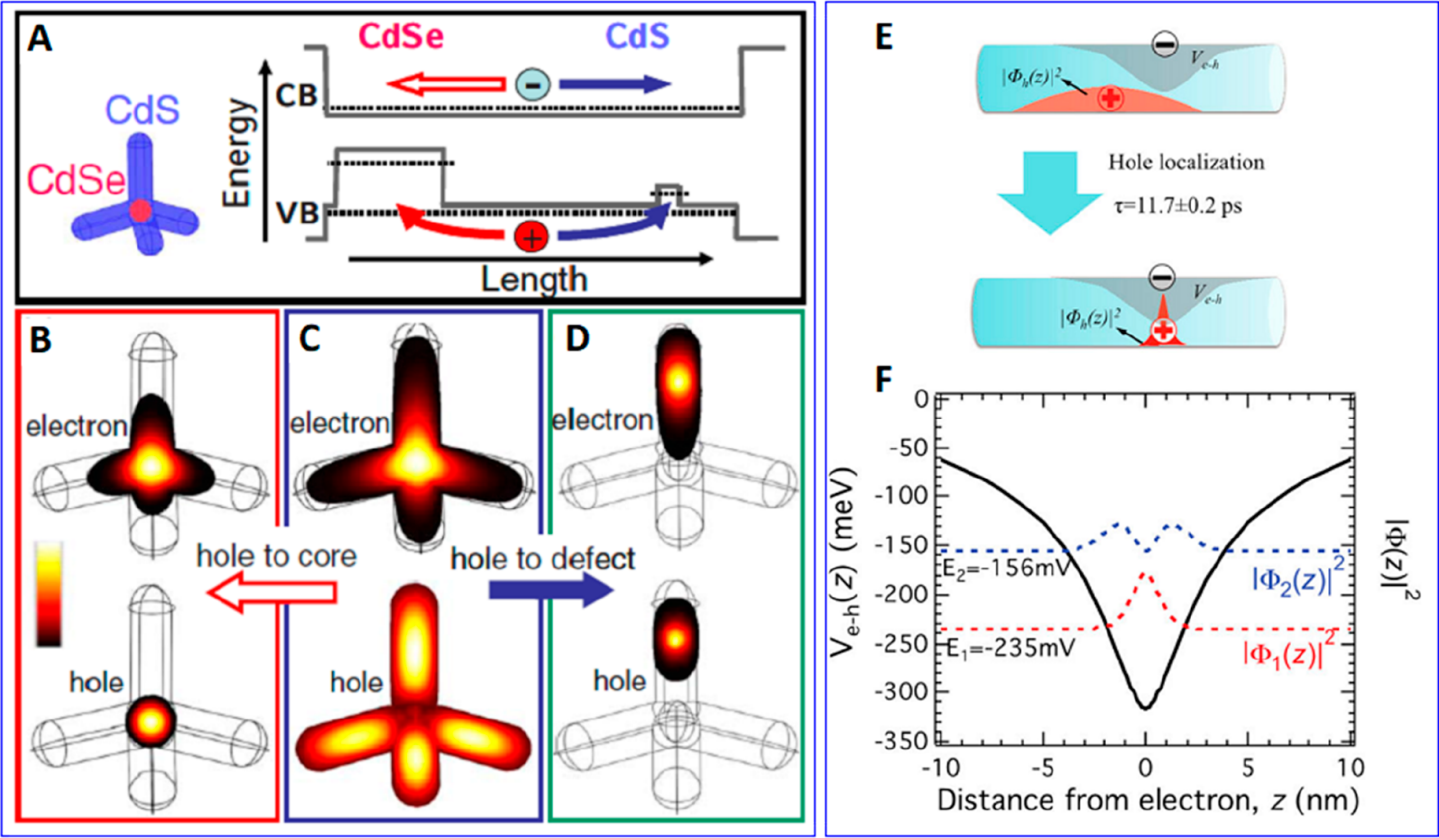
(A–D) Calculated electron and hole wave functions in CdSe@CdS tetrapods. (A) Energy level plotted along the long axis of one of the tetrapod arms. The arrows indicate the real-space dynamics of the electron and hole transfer to the core (red) or to a trapping site (blue). (B) Electron and hole wave functions at the band edge of the structure with the hole confined in the CdSe. (C) Electron and hole at the band edge states of the CdS rod before relaxation of the hole to the CdSe core. (D) The hole wave function is localized in a low-energy trapping site in one of the CdS arms, resulting in the localization of the electron wave function to the same arm. (E) Schematic illustration of the hole localization after ET from a CdSe NR to adsorbed methylene blue. (F) Coulomb potential well (black line) and hole wave function density distribution, in the lowest two states, in charge-separated CdSe NR–MB. (A–D) Adapted with permission from ref (198). Copyright 2010 American Physical Society. (E and F) Adapted with permission from ref (224). Copyright 2016 American Chemical Society.

Mauser and co-workers reported the effect of electron–hole binding on electron and hole wave functions in quasi-type II CdSe@CdS tetrapods (Figure 7A-D).^198^ They calculated the wave functions of the CB electron and VB hole using an EMA model that takes into account e-h Coulomb interactions. The Coulomb attraction energy used in the calculations is 75 meV, which is a lower limit but already significantly affects the electron and hole wave functions. The initially photogenerated electron and hole are delocalized along the CdS rod arms (Figure 7C). The hole wave function is then localized to the CdSe core region due to a large VB offset between the CdSe and CdS. As a consequence of the strong e-h interaction, the electron wave function is also localized in and near the CdSe core instead of delocalized over the CdS arms despite the quasi-type II electronic structure of the tetrapods (Figure 7B). Not only the band edge hole but also a trapped hole can bind strongly to the electron. As shown in Figure 7D, when the hole is localized to a defect state on the CdS arm, the electron wave function is also localized in and near the hole trapping site. In a more recent work, Beard and co-workers showed that even an electron transferred to an acceptor bound to the NR surfaces can strongly localize the hole to near the reduced acceptor.^224^ As schematically shown in Figure 7E, photoexcitation of CdSe NR–methylene blue (MB) complexes leads to electron transfer from the CdS to MB in ∼3.5 ps, as measured by transient absorption spectroscopy. Additional measurements using time-resolved terahertz spectroscopy indicate that the hole remained in the rod, localized around the reduced MB in ∼11.7 ps. Calculations performed by Efros and co-workers show that, because of the strong electron–hole binding energy that acts as a Coulomb potential well for the hole, the wave function of the lowest energy hole in the bound state is localized to a ∼ ±0.8 nm region near the reduced electron acceptor, and the activation energy to detrap the hole from the potential well can be as large as 235 meV.^224^

In some cases, the electron–hole binding energy is stronger than the energy level offset in heterostructures such that it does not simply modify the wave function but rather completely alters the localization behavior of carriers. As demonstrated by Wu and co-workers, the CB level offset in CdSe@CdS dot-in-rod NRs can be probed by doping an electron into the core and observing the localization behavior of the doped electron.^225^ They find that for NRs with relative small CB level offset the electronic structure of the NR probed by electron-doping is quasi-type II whereas transient absorption measurement reveal they are type I NRs. Thus, these NRs behave as a quasi-type II structure for a single CB electron but as a type I structure in the presence of an electron–hole pair. All these reports suggest that for 1D and 2D hetero-NCs the band alignment (electronic structure) and electron–hole coulomb interaction codictate the spatial distribution of the electron and hole wave functions. Furthermore, the interfacial strain, introduced by external force or additional growth of heterostructures, also provides strong impact on electronic structure and carrier dynamics of NCs.^226^ With a sub-100 nm dimension, NC heterostructures can withstand significant elastic deformations, with their equilibrium lattice constant differing from their bulk counterpart. It has been reported that strain can convert the band alignments of CdTe/ZnSe QDs from type I to a type II.^227^ Moreover, strain also strongly affect carrier relaxation and recombination in CdSe/CdTe^228−230^ and CdSe/CdS NR heterostructures.^226^

### 3.2 Charge Transfer and Transport Dynamics Inside Hetero-NCs

**Figure 8:**
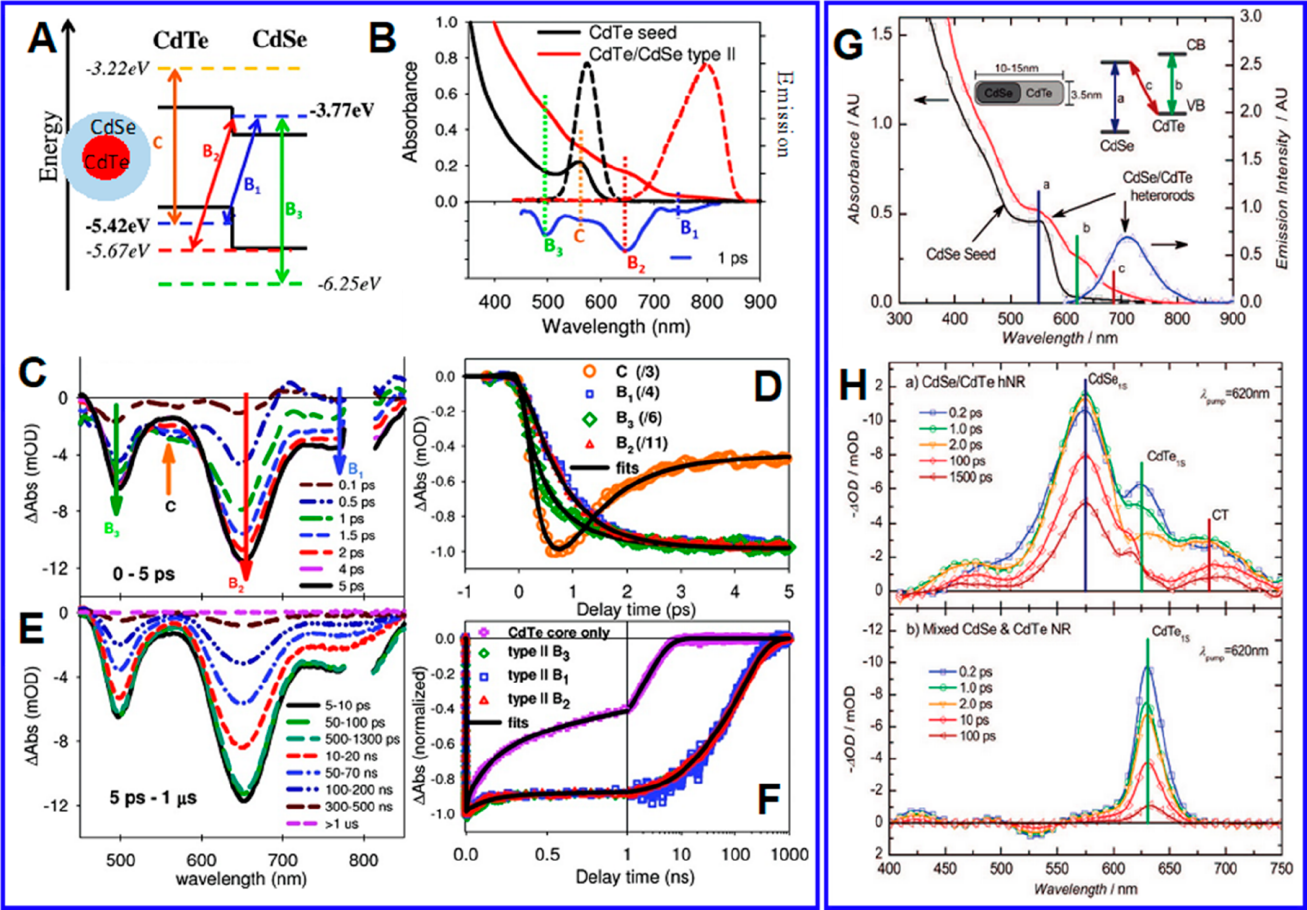
Charge separation dynamics in type II heterostructured nanocrystals. (A) Band alignment diagram of bulk CdTe/CdSe (black solid lines) and calculated quantum-confined energy levels (colored dashed lines) of CdTe/CdSe QDs. B1, B2, B3, and C transitions are indicated by arrows. (B) Absorption (solid) and emission (dashed) spectra of CdTe seeds (black) and type II CdTe/CdSe core/shell QDs (red). Also shown is a TA spectrum of CdTe/CdSe QDs probed at 1 ps after 400 nm excitation, which clearly shows four bleach bands (B1, B2, B3, and C). (C and E) TA spectra of CdTe/CdSe QDs at selected delays in (C) 0–5 ps and (E) 5 ps to 1 μs following 400 nm excitation. (D) Formation and decay kinetics of the bleaches at the B1, B2, B3, and C bands from 0 to 5 ps. These signals have been scaled by factors indicated in the legend for better comparison. (F) Comparison of the bleach recovery kinetics of B1, B2, and B3 bands in CdTe/CdSe QDs with the exciton bleach recovery kinetics of CdTe seeds. (G) Absorption (red) and emission (blue) spectra of CdSe/CdTe NRs. The inset illustrates three absorption bands: lowest-energy exciton states of CdSe (a) and CdTe (b) and charge transfer (CT) transition (c). (H) TA spectra of CdSe/CdTe NRs (top) and mixed CdSe and CdTe NRs (bottom) at indicated time delays following 620 nm excitation (exciting CdTe). (A–F) Adapted with permission from ref^234^. Copyright 2011 American Chemical Society. (G and H) Adapted with permission from ref (235). Copyright 2008 American Chemical Society.

From the standpoint of charge transfer from NCs, wave-function-engineered hetero-NCs are attractive because of their internal charge transfer/separation behaviors that can be used to tailor the rate and yield of charge transfer to external acceptors. As such, it is important to understand charge transfer/separation inside hetero-NCs. For hetero-QDs, we illustrate internal charge separation using CdTe/CdSe type II core/shells (Figure 8A–F).^147^ The absorption spectra of these QDs shows B1, B2, B3, and C features (Figure 8B). According to EMA calculations, B1 and B2 can be assigned to the CT transitions from the CdTe VB to the CdSe CB, and B3 and C can be assigned to the transitions within CdSe and CdTe, respectively (Figure 8A). The TA spectra of core/shell QDs after 400 nm excitation show that the decay of the bleach of the C feature leads to the concomitant growth of the bleach at the B1, B2, and B3 features (Figure 8C), which can be assigned to interdomain electron transfer from the CdTe core to CdSe shell because the bleach of exciton bands is dominated by the contribution of the CB electron. Fitting the kinetics of these features (Figure 8D) reveals that this internal charge separation process occurred with a time constant of ∼0.77 ps. Similar sub-ps processes have been reported in other type II QDs.^231−233^ After initial charge separation, all the TA features that correspond to the CdSe CB edge electrons are long-lived (Figure 8E). Fitting the TA features reveals an excited-state lifetime of 62 ns (half-life) for the CB electron, which is more than one order of magnitude longer than that in CdTe core QDs (Figure 8F). Thus, the wave function engineering approach not only effectively localizes the electron and the hole to the shell and the core, respectively, which facilitates electron transfer to external acceptors and suppresses ensuing charge recombination (illustrated in the next section), but also leads to a longer-lived excited state for more efficient charge extraction.

Internal charge separation was also reported for CdSe/CdTe type II hetero-NRs (Figure 8G and H).^235^ The absorption spectrum of CdSe/CdTe NRs contains a, b, and c bands that can be assigned to the 1Σ excitonic feature of the CdSe, the 1Σ excitonic feature of the CdTe, and the CT transition from the CdTe VB to the CdSe CB, respectively (Figure 8G). The PL spectrum of CdSe/CdTe consists of only the CT band emission (Figure 8G), indicating efficient charge separation between CdSe and CdTe domains. These static spectra features are similar to those of type II QDs. The internal charge separation dynamics was investigated using TA spectroscopy by applying a 620 nm excitation pulse that resonantly excites the CdTe 1Σ band (Figure 8H). The CdTe bleach feature forms instantaneously and then shows significant decay within 2 ps, which is accompanied by the growth of the CdSe and CT band bleach. Analysis of the TA kinetics reveals a CdTe-to-CdSe electron transfer time constant of ∼500 fs. In a related work, Zamkov and co-workers measured carrier dynamics in CdS/ZnSe type II nanobarbells and reported an electron transfer time of ∼0.35 ps from the ZnSe tips into the CdS NR.^236^ These time constants are similar to those reported in CdSe/CdTe core/shell QDs above (∼770 fs^147^) and also 2D core/crown nanoplatelets (∼640 fs^211^).

In principle, the electron transfer time in core/shell QDs only contains interfacial electron transfer and that in hetero-NRs or core/crown nanoplatelets contains both interfacial electron transfer and carrier/exciton transport to the interface. The even faster time constant observed in NRs and nanoplatelets indicates that the carrier/exciton transport in these materials is fast and there likely exists charge transfer barrier in curved core/shell interface in QDs caused by interfacial lattice strain.^237^ These results show that interdomain electron transfer inside these 1D NRs and 2D NPLs are often fast due to fast carrier/exciton transport along the NRs and NPLs and strong electronic coupling between epitaxially grown semiconductor domains.

**Figure 9:**
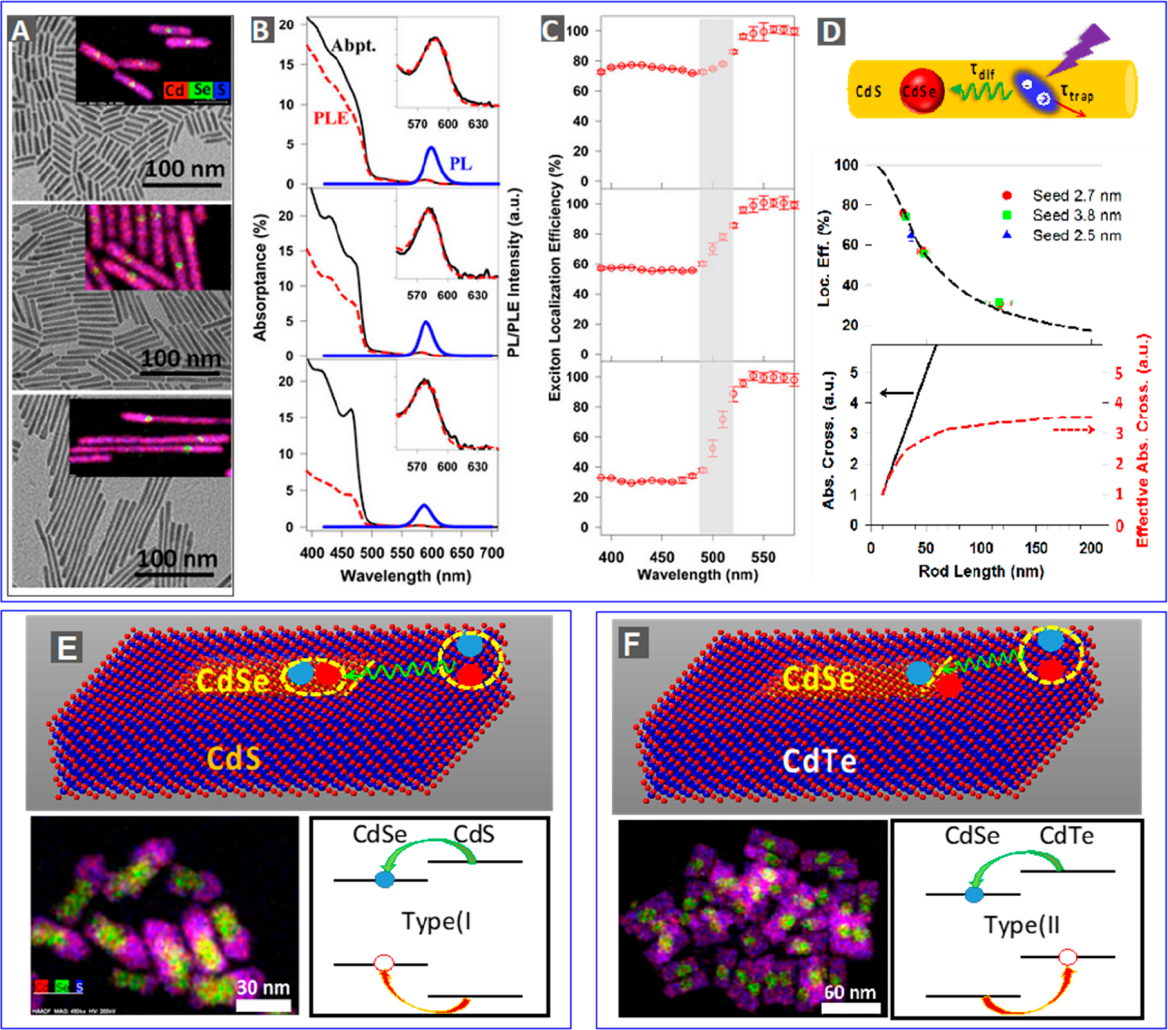
Competition between exciton transport and trapping in hetero-NRs and NPLs. (A) TEM images of CdSe@CdS NRs with 2.7 nm CdSe cores and different lengths (top, 29 nm; middle, 47 nm; bottom, 117 nm). Insets show the corresponding EDX elemental maps indicating the location of the CdSe core (green). (B) Absorptance (black solid line), PL (blue solid line), and PLE (red dashed line) spectra of NRs shown in (A). The PLE and absorptance spectra have been normalized at the lowest energy exciton peak in the core, as shown by the insets. (C) Wavelength-dependent relative PL QYs of the NRs shown in (A). The shaded areas show regions of gradual decrease of relative PL QYs. (D) Scheme showing the competition between exciton transport to the core through 1D diffusion and exciton localization on the rod through hole trapping (top). Measured (symbols) and simulated (dashed line) exciton localization efficiencies in CdSe@CdS NRs (middle). The efficiency is independent of core sizes: 2.5 nm (blue triangles), 2.7 nm (red circles), and 3.8 nm (green squares). CdS rod absorption cross-section (black solid line) and effective CdSe seed absorption cross section (red dashed line) as a function of rod length (bottom). (E) Schematics of crown-to-core exciton diffusive transport, EDX image, and band alignment in type I CdSe/CdS core/crown NPLs. (F) Schematics of crown-to-core exciton diffusive transport, EDX image, and band alignment in type II CdSe/CdTe core/crown NPLs. (A–D) Adapted with permission from ref (177). Copyright 2015 American Chemical Society. (E) Adapted with permission from ref (221). Copyright 2016 American Chemical Society. (F) Adapted with permission from ref (212). Copyright 2017 American Chemical Society.

For 1D NRs and 2D NPLs, surface/interfacial carrier trapping and strong electron–hole binding also play critical roles in the carrier/exciton transport dynamics. These effects in 1D NRs were illustrated in our previous work by studying the length dependence of rod-to-seed exciton localization efficiency in CdSe@CdS NRs.^177^ Transmission electron microscopy (TEM) images and energy dispersive X-ray spectroscopy (EDX) maps can be used to accurately determine the rod-to-seed exciton transport distances (Figure 9A).^181,195^ In these NRs, photoluminescence is dominated by excitons localized in the CdSe seed. Through photoluminescence excitation (PLE) measurements, the rod-to-seed exciton localization efficiency can be determined by comparing the PLE spectra of these NRs to their absorptance spectra (Figure 9B). Specifically, by normalizing the PLE spectra to corresponding absorptance spectra at the B2 band (Figure 9B, inset), the efficiency of exciton localization from the CdS rod into the CdSe seed can be calculated from the ratio of normalized PLE over absorptance.^207^ The ratio is 1 at >520 nm, decreases gradually at 480–520 nm, and finally levels off at <480 nm, where the CdS rod absorption dominates, to ∼76%, 56%, and 30% for NRs with lengths of 29, 47, and 117 nm, respectively (Figure 9C).

The length-dependent exciton localization efficiencies are modeled by a 1D diffusion model that includes exciton surface trapping:^207^, where *D*_X_ is the exciton diffusion constant, *N*(*x*, *t*) is the time- and position-dependent exciton concentration, and τ_Trap_ is the exciton trapping time (0.78 ps).^177^ This model implicitly assumes that charge transfer at the CdSe–CdS interface is much faster than exciton diffusion along the rod. As shown in Figure 9D (middle), with *D*_X_ as the only fitting parameter, we can simultaneously fit the localization efficiency for all NRs. The best fit gives a diffusion constant *D*_X_ of 2.3 cm^2^/s, slightly smaller than the bulk value of 3.2 cm^2^/s.^238−242^ According to this diffusion constant, exciton diffusion over 10 nm takes ∼0.5 ps (and further scales quadratically with diffusion length). Based on the length-dependent exciton localization efficiency in these CdSe@CdS NRs, we define an effective absorption cross section as a product of the rod absorption cross section and the localization efficiency, which represents the cross section for creating excitons in the seed through absorption at the rod.^177^ As shown in Figure 9D (bottom), the effective seed absorption cross section first increases with the rod length and approaches saturation at a rod length of ∼100 nm, with an effective seed absorption cross section saturating at ∼3.5-fold of that of the short rod. This result suggests the existence of an optimal rod length for light harvesting applications. In addition to CdSe@CdS hetero-NRs, competition between exciton transport and interfacial trapping was also reported in CdSe tetrapods, which are homojunctions with a zinc blende CdSe core with four wurtzite CdSe rod arms.^243^ Upon exciting the wurtzite arms, 86% of the excitons are transported to the zinc blende core with a time constant of ∼1 ps, driven by the CB offset across the rod/core quasi-type II interface. The remaining 14% form a charge-separated state across the interface, with the electron in CdSe core and hole trapped at the CdSe rod.

The exciton transport behaviors in 2D hetero-NPLs are different from those in 1D NRs. In our previous work, we compared the PLE (monitored at core emission) and absorptance spectra of CdSe/CdS type I core/crown NPLs to quantify the crown-to-core exciton transport efficiency (Figure 9E).^221^ Using CdSe/CdS core/crown NPLs with the same CdSe core and different CdS crown sizes, we found that the crown-to-core exciton localization efficiency does not depend on the crown size but instead depends on the excitation wavelength, which higher efficiency at higher excitation energy.^221^ Unlike NRs, CdSe/CdS NPLs have negligible surface trap states on the well-passivated basal planes, and their trap states are concentrated at the core/crown interface, independent of the crown size.^244^ The wavelength-dependent transport efficiency is attributed to more efficient transport of “hot” excitons with excessive energy (the energy difference between excitation energy and CdS crown band gap) bypassing the interfacial trap.^221^ Interestingly, for CdSe/CdTe type II core/crown NPLs, the PLE (monitored at CT-exciton emission) and absorptance spectra agree very well with each other, indicating a unity CT-exciton formation efficiency (Figure 9F).^211,212^ The crown-to-core exciton transport efficiency in type II hetero-NPLs is excitation-wavelength-independent, which is likely because the CT-exciton (with the electron in the CdSe core and hole in the CdTe crown) is formed prior to trapping process and does not require the hole to move across the interface.^211,245^ Recently, Rao and co-workers also observed emissions from the CdSe core and CdTe crown of CdSe/CdTe NPLs, indicating the CT exciton formation efficiency is not unity.^218^ Although the emission property may change with the sample quality, all these results have shown that interface of hetero-NPLs is important for both exciton transport and emission.

Due to the uniform quantum confinement along the thickness direction and the giant oscillator strength transition effect, it is speculated that the wave function of the exciton center-of-mass can delocalize throughout the whole NPL, resulting in a ballistic exciton in-plane transport property.^246,247^ Buhro and co-workers studied single CdSe quantum belts and showed that the spatial distribution of the PL intensity is independent of the excitation location.^247^ They attributed this result to the exciton center-of-mass motion delocalizing to the whole quantum belt at the room temperature. However, Ma and co-workers have shown recently that the exciton center-of-mass wave function extension at room temperature (∼160 nm^2^) can be smaller than the NPL lateral dimension.^248^ In our recent works, we showed that the exciton transports diffusively in both CdSe/CdS and CdSe/CdTe core/crown NPLs.^212,221^ Take CdSe/CdS type I hetero-NPLs as an example, the exciton bleach of the CdS crown decays slower in larger CdS crowns, indicating slower exciton transport from the larger CdS crowns; correspondingly, the formation of exciton bleach of the CdSe core, which represents the exciton arrival at CdSe core, is slower at larger CdS crowns.^221^ These size-dependent exciton transport kinetics show that the exciton does not transport ballistically and can be well fitted by 2D in-plane classical diffusion model with a diffusion constants as 2.2 cm^2^/s and 2.5 cm^2^/s for CdS and CdTe NPLs, respectively, close to the diffusion constant in bulk crystals.^212,221^

## 4 Electron and Hole Transfer from Nanocrystals

### 4.1 Nonadiabatic Charge Transfer from QD in a Weak Coupling Regime

In the conventional two-states Marcus model describing electron transfer from a discrete reactant state to a discrete product state, the nonadiabatic electron transfer (ET) rate can be described by eq 4.1.^134,249−251^
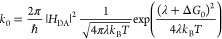
4.1Here λ is the reorganization energy of the donor–acceptor system associated with the ET process due to electron–nuclear interaction, |*H*_DA_|^2^ is the electronic coupling strength between initial and final states, and Δ*G*_0_ is the free energy difference between the initial state and final state (−Δ*G*_0_ is the so-called driving force). Next we will discuss how these factors affect ET rates in QD–acceptor systems and how this model should be modified to count the unique excitonic properties of QDs.^134^

#### 4.1.1 Reorganization Energy

The total reorganization energy λ contains the inner-sphere contribution (λ_i_) from the nuclear displacement of the donor–acceptor system and the outer-sphere contribution (λ_o_) from the solvent dielectric response (λ = λ_i_ + λ_o_). Typically, QD contributes negligibly (<10 meV) to λ_i_ because of its weak electron–phonon coupling,^252−254^ and λ_i_ mostly comes from acceptor molecules, which is often in the range of a few hundred meV and can be computed theoretically.^251,255,256^

The solvent molecules contribute to the charge transfer reorganization energy (λ_o_) mostly through the orientation polarization, and λ_o_ can be estimated using dielectric continuum model^257^
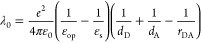
4.2where ε_op_ and ε_s_ are solvent high-frequency optical and static dielectric constants, respectively (therefore, the first bracket reflects the pure solvent orientation contribution); *d*_D_ and *d*_A_ are diameters of spherical donor and acceptor cavities, respectively; and *r*_DA_ is the center to center distance of the spherical donor and acceptor cavities. According to eq 4.2, a nonpolar solvent (such as hexane) contributes negligibly to the reorganization energy because of vanishing orientation contribution. Indeed, Ellis et al. observed similar ET kinetics of QD–methyl viologen (MV^2+^) complexes dispersed in hexane and under vacuum, consistent with negligible reorganization energy from a nonpolar solvent.^250^

Various experiments have been attempted to examine the dependence of the ET rate on solvent polarity and thus the effect of reorganization energy.^258−260^ Cui et al. showed that ET rates between CdSe/ZnS QDs and pyromellitimide did not show a clear correlation with the solvent reorganization energy.^258^ Similarly, Hyun et al. reported that ET rates from a PbS QD to 10-dodecylanthracene-9-thiol did not correlate with solvent reorganization energy but increased with the static dielectric constant.^259^ Unfortunately, the acceptor molecules in these studies are soluble in solvents and the ratio between QD donor and molecular acceptors, which affects the apparent ET rate, is poorly controlled, hindering a careful study on solvent reorganization energy.

Charge transfer from QDs to semiconductor films (*e.g.*, TiO_2_) in principle can circumvent the problem of a poorly defined QD to adsorbed acceptor ratio. Hyun et al. reported that ET rates from a PbS QD to colloidal TiO_2_ nanoparticles showed weak solvent dependence^260^ and attributed it to the small solvent contribution as a result of large TiO_2_ nanoparticles. In principle, the solvent molecules around PbS QD (with much smaller size compared to TiO_2_) should contribute to the ET reorganization energy. It appears that the solvent-dependent ET rates from QDs as predicted by eq 4.2 have not yet been observed. While better experiment designs may be needed, another possibility could be that the model in eq 4.1 does not adequately describe the ET from QDs,^134^ as will be discussed later.

#### 4.1.2 Electronic Coupling

Electronic coupling strength dependence can be most conveniently examined in donor–bridge–acceptor (D-B-A) complexes, in which the donor–acceptor coupling strength depends on the donor chemical nature and geometrical factors, acceptor, and bridge.^261^ If ET occurs by tunneling through the bridge, the electronic coupling strength, and thus the ET rate, depends exponentially on the donor–acceptor distance.^261,262^

**Figure 10:**
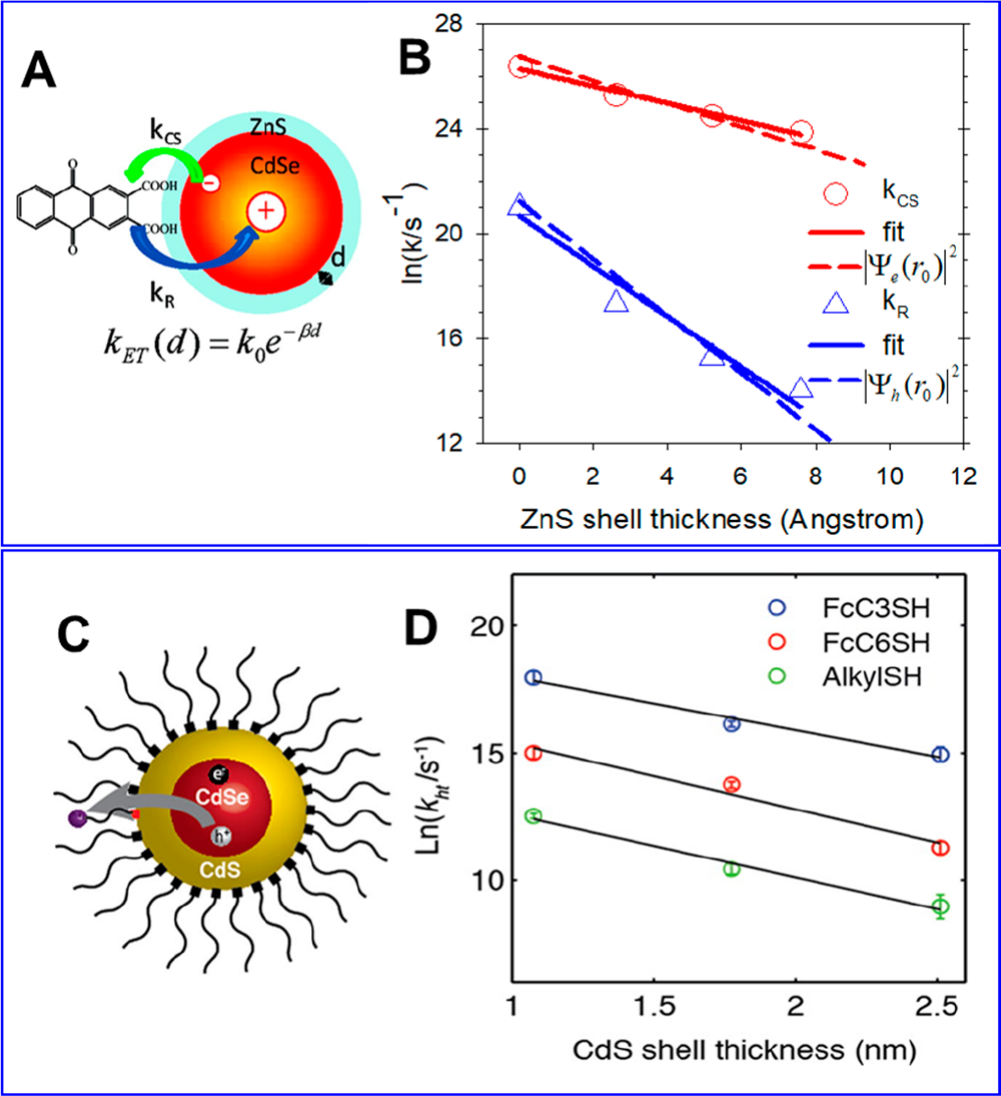
(A) Scheme showing electron transfer (charge separation) and back electron transfer (charge recombination) processes in CdSe/ZnS core/shell QD–anthroquinone molecule complexes. (B) Plot of the logarithm of *k*_CS_ (red circles) and *k*_R_ (blue triangles) rates as a function of the ZnS shell thickness. Also shown are the calculated electron (red dashed line) and hole (blue dashed line) densities at the shell surface as a function of the shell thickness. (C) Scheme showing hole transfer from CdSe/CdS core/shell QD to surface adsorbed ferrocene molecules. (D) Plot of the logarithm of hole transfer rate as a function of CdS shell thickness for three different hole acceptor molecules. (A and B) Adapted with permission from ref (263). Copyright 2010 American Chemical Society. (C and D) Adapted with permission from ref (264). Copyright 2015 American Chemical Society.

An unique and precise way to tune the QD–acceptor distance and ET electronic coupling strength is through an inorganic barrier layer between the QD and acceptor with controlled thickness, *e.g.*, using core/shell QDs.^147,263−267^ Using a CdSe/ZnS type I core/shell QD-anthraquinone (AQ) complex and by varying ZnS shell thickness (Figure 10A), Zhu et al. found both the electron transfer (or charge separation, *k*_CS_) and back electron transfer (or charge recombination, *k*_R_) rates decay exponentially with the shell thickness (*d*).
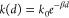
4.3Here *k*_*0*_ is the CT rate at *d* = 0 and the exponential decay constants β were reported to be 0.35 ± 0.03 Å^–1^ and 0.91 ± 0.14 Å^–1^ for the electron and hole transfers, respectively.^263^ More interestingly, the ET and HT rate decay constants agree well with the exponential decrease of 1S electron and hole surface density, respectively (Figure 10B). This result confirms that the ZnS shell serves as a tunneling barrier for the electron and hole transfer and slows down their rates by decreasing the electronic coupling with the adsorbate. Besides electron transfer, Ding et al. systematically investigated the hole transfer process from CdSe/CdS core/shell QDs to three different ferrocene hole-accepting molecules (Figure 10C) and found a general exponential dependence of hole transfer rates on the CdS shell thickness (Figure 10D).^264,265^ Similar shell-thickness-dependent CT behavior has been also observed in ZnTe/CdSe core/shell QDs.^267^ The specific ET (HT) attenuation factor β varies for different systems and depends on the electron (hole) effective mass in shell materials and the core/shell band offset (potential barrier). This suggests exciting opportunities for independently controlling ET and HT (or BET) rates from QDs and leads to the idea of “wavefunction engineering” for controlling charge separation and recombination process in heterostructures.^168^ For an in-depth discussion about wave function engineering and its application, reader can refer to the review paper in ref (168).

**Figure 11:**
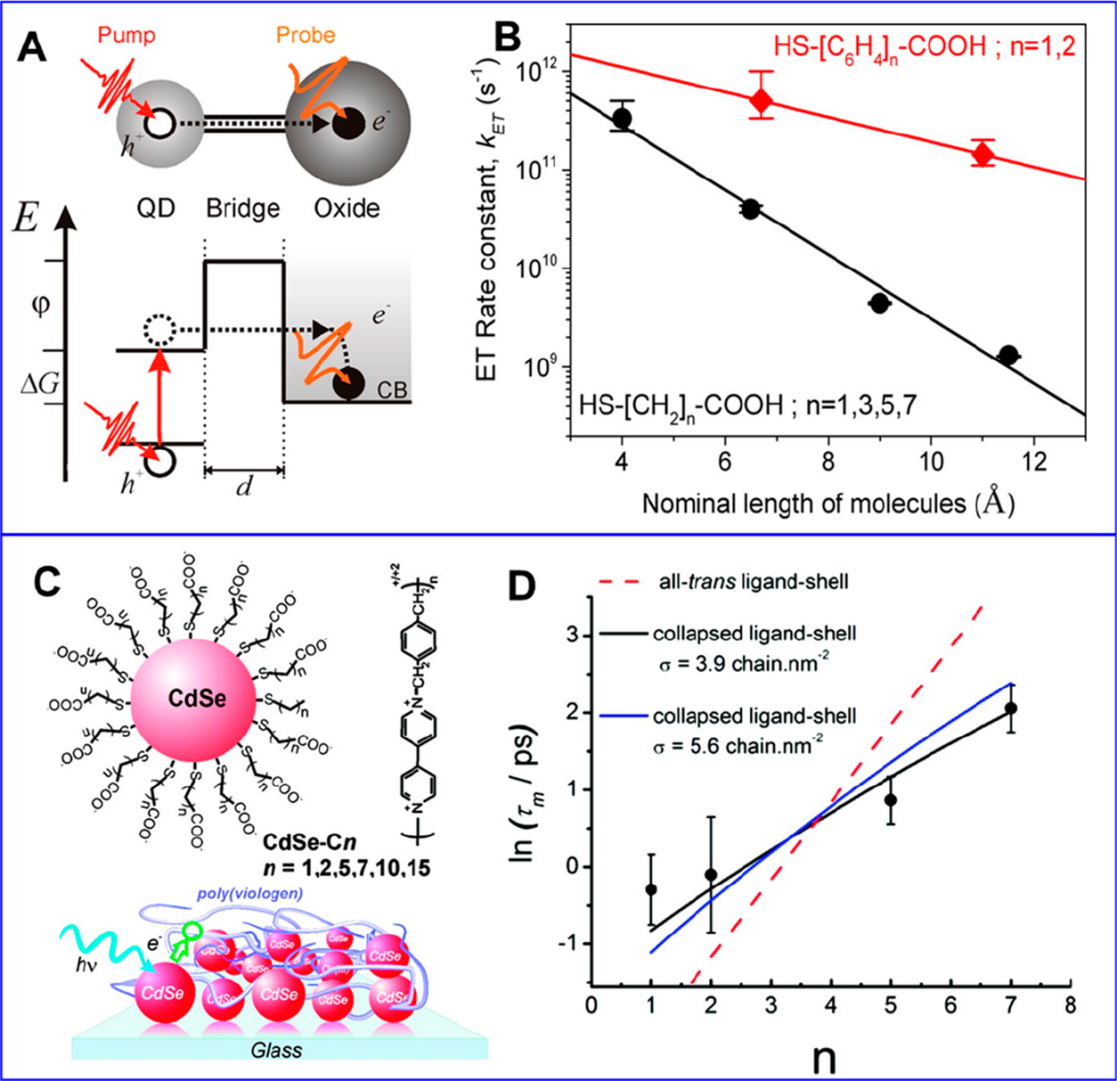
(A) Schematic depiction of time-resolved THz photoconductivity measurements on a (CdSe)QD–bridge–(TiO_2_) oxide system and relevant energetics of donor, bridge, and acceptor. (B) ET rate constants vs molecular bridge length for *n*-methylene-based bridges (HS–[CH_2_]_*n*_–COOH, with *n* = 1, 3, 5, 7; black dots) and *n*-phenylene-based bridges (HS–[C_6_H_4_]_*n*_–COOH, with *n* = 1, 2; red squares). Solid lines are best fits to eq 4.3. (C) Scheme of mercaptocarboxylic acid-coated CdSe QDs with different bridge lengths, the polyviologen (PV) structure, and multilayer films of QDs coated by mercaptocarboxylic acids and PV on glass substrates. (D) Plot of the logarithm of the electron transfer time constant for PV/CdSe-C_*n*_ films as a function of *n*. The red dashed line shows the best-fit curve assuming electron tunneling across all-*trans* ligands. Solid lines show the predictions using collapsed-shell model. (A and B) Adapted with permission from ref (268). Copyright 2013 American Chemical Society. (C and D) Adapted with permission from ref (269). Copyright 2011 American Chemical Society.

Another way to change the donor–acceptor distance is to vary the length of the bridge molecule, which has been extensively applied in both QD-semiconductor film systems^269−272^ and QD–molecule acceptor systems.^260,268,273^ Wang et al. studied the ET process from CdSe QDs to TiO_2_ films linked by *n*-methylene-based SH–[CH_2_]_*n*_–COOH (*n* = 1, 3, 5, 7) or *n*-phenylene-based SH–[C_6_H_4_]_*n*_–COOH (*n* = 1, 2) as a function of *n* using optical pump-terahertz probe spectroscopy (Figure 11A).^268^ They observed a clear exponential decay of the ET rate as a function of the bridge length with an attenuation factor of β = 0.75 ± 0.06 Å^–1^ for the *n*-methylene bridges, which agrees quantitatively with values from conductance measurements.^274^ On the other hand, the bridge geometry can also dramatically alter the influence of bridge length on the ET rate and leads to a much weaker dependence.^269−271^ Tagliazucchi et al. measured photoinduced electron transfer from CdSe QDs with a HS–(CH_2_)_*n*_–COOH ligand (*n* = 1, 2, 5, and 7) to poly(viologen) as a function of *n* (Figure 11C) and observed that the attenuation of the ET rate constant with *n* is weaker than that expected from the decay of the electron tunneling probability across extended all-*trans* mercaptocarboxylic acids but can be well described by electron tunneling across a collapsed ligand shell (Figure 11D).^269^ Morris-Cohen et al. even found that ET rates from QD to alkylcarboxylate-functionalized viologens are independent of the number of methylene groups between the carboxylic acid and the bipyridinium core,^270^ suggesting that the dominant ET pathway is a through-space pathway from the QD to the bipyridinium core directly adsorbed on the QD surface, bypassing the bridging chain. These results indicate the importance of molecular-level morphology of the bridge ligands and suggest that ET studies could also provide a sensitive probe of the QD ligand shell structure and dynamics.

In addition to the bridge length and geometry, the chemical nature of the bridge and anchor groups has also been shown to affect the ET rates.^260,268,272,275−277^ As shown in Figure 11B, the attenuation factor β decreases from 0.75 Å^–1^ for a methylene bridge to 0.29 Å^–1^ for a phenylene bridge due to stronger interunit coupling and the smaller barrier height in the latter.^268^ Hyun et al. compared the ET rate from a PbS QD to TiO_2_ nanoparticles with four different anchor groups (carboxylic acid group, phosphonic acid, silane group, and sulfonic acid) binding to TiO_2_ and observed that sulfonic acid yielded the highest ET rate.^260^

In principle, the electron transfer coupling matrix element, |*H*_DA_|^2^, can be computed using the effective one-electron model that has been successfully applied for inter- and intramolecular ET processes.^261^ However, full *ab initio* electronic structure calculations of QDs of realistic sizes remain challenging. Computational studies of ET processes in QD–acceptor complexes have been carried out for smaller nanoclusters at the TD-DFT level^278^ or larger particles (comparable to experiments) at the empirical pseudopotential level.^279^ Motivated by the experimental observations that the ET and HT coupling matrix element depend on the electron (hole) density that extends outside the QDs (Figure 10B),^263^ a hybrid method was developed to compute |*H*_DA_|^2^ in QD–acceptor complexes.^251^ The computed ET rates were found to be in reasonable agreement with the measured values, suggesting that such approximate methods may worth further investigation.^251^

#### 4.1.3 Driving Force

In QD–acceptor complexes, Δ*G* comes from three main contributions: (1) the potential energy difference between the donor (*e.g.*, QD 1S electron, *E*_1Se_) and acceptor orbitals (*E*_A/A^–^_), (2) the electron–hole Coulombic interaction in both the initial excited state (*E*_e-h_) and the final charge-separated state (*E*_CS_), and (3) the charging energy (*E*_c_) from the initially neutral system to the charge-separated state. With all three contributions, the total free energy change is
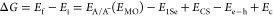
4.4The redox potential energy for 1S electron (or hole) level of the QD or acceptors (*E*_A/A^–^_) can be directly obtained from electrochemical method, *i.e.*, cyclic voltammetry^280−284^ (solution phase) or photoelectron spectroscopy^285^ (dry films). The 1S_e_ (1S_h_) level of QDs of known sizes can also be estimated using effective mass approximation.^77,281^ The latter three terms in eq 4.4, *E*_CS_*, E*_e-h_ and *E*_C_, cannot be measured directly from experiments but can be calculated using a simple model (see refs (134) and (253) for the detailed process). Because of a partial cancellation of *E*_CS_ and *E*_e-h_*,* the overall e-h Coulombic interaction contribution to the free energy change is weakened.

#### 4.1.4 Charge Transfer from QD to Molecular Acceptors with Discrete States

According to the classical Marcus ET model (eq 4.1), with increasing driving force (−Δ*G*), the ET rate increases in the normal regime (−Δ*G* < λ), reaches a maximum at the barrierless regime (at −Δ*G* = λ), and decreases in the inverted regime (−Δ*G* > λ). The prediction of the existence of the inverted regime has been the most prominent feature of Marcus ET theory and has been experimentally observed in molecular donor–acceptor systems.^286^ QDs with discrete and tunable electronic levels provide an ideal system to explore the driving force dependence and test theoretical models for describing ET from nanoscale excitonic systems. Changing QD size changes the ET driving force, and ET rates from the photoexcited QDs as a function of QD sizes to molecular acceptors^134,287−292^ and metal oxide semiconductor films^37,253,254,293,294^ have been extensively studied. Here, we focus on ET to molecular adsorbates; ET from QDs to metal oxide semiconductor will be discussed in the next section.

**Figure 12:**
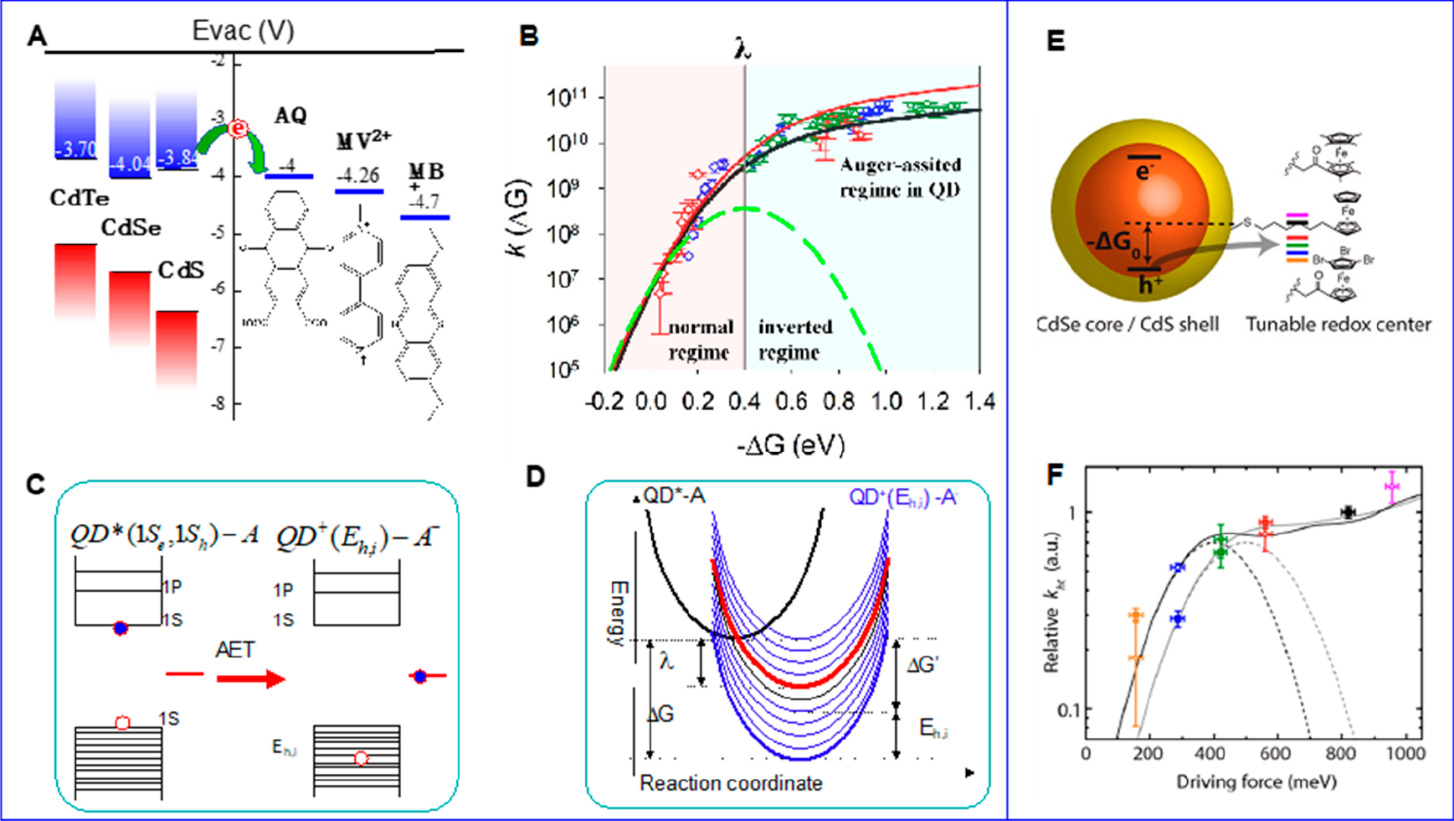
(A) Schematic diagram of bulk conduction band edge positions of CdX (X = S, Se, Te) and reduction potentials of acceptor molecules (vs vacuum). (B) Measured (symbols) and predicted (lines) ET rates as a function of the driving force according to the conventional (green dashed line with λ of 0.4 eV) and Auger-assisted (red and black solid lines with λ of 0.4 and 0.5 eV, respectively) ET models. (C) Auger-assisted model for ET from QDs where ET can be coupled with a change in the hole energy level. (D) Marcus representation showing the energy of the reactant and product states as a function of the nuclear displacement along the ET coordinate. (E) Schematic of hole transfer from CdSe/CdS core/shell QDs to adsorbed ferrocenes. (F) Plot of the relative hole transfer rate constant as a function of the driving force. The dashed lines show behavior expected from a two-state Marcus model. The solid lines show behavior expected from the Auger-assisted model. Reorganization energies of 400 (black) and 500 meV (gray) were used. (A–D) Adapted with permission from ref (134). Copyright 2014 American Chemical Society. (E and F) Adapted with permission from ref (291). Copyright 2015 American Chemical Society.

An increase of ET rates with decreasing QD sizes has been generally observed. Early studies of ET in QD–acceptor complexes have covered a relatively small driving force range (<0.6 eV), and a critical test of ET models, especially the presence of Marcus inverted regime, has not been possible.^287−290^ Zhu et al. investigated ET processes from CdS, CdSe, and CdTe QDs of different sizes to three molecular acceptors, namely, methylene blue (MB^+^), methylviologen (MV^2+^), and anthraquinone (AQ), as shown in Figure 12A, to cover a wide driving force range (0–1.3 eV).^134^ The observed ET rates increase monotonically with increasing driving force (or decreasing QD size) regardless of QD compositions and acceptor redox potentials (Figure 12B symbols). With the estimated total reorganization energy of ∼0.4 eV, this result is in marked contrast with the conventional two-state ET model (eq 4.1 and Figure 12B, green dashed line). Considering enhanced electron–hole Coulomb interaction in QDs, the authors proposed an Auger-assisted ET model to successfully account for the observed driving force dependence.

In the Auger-assisted model, the excess energy of the transferred electron can be conserved by the excitation of 1S holes to a higher level (with energy *E*_h,*i*_ below the 1S hole), in addition to exciting vibrations of the lattice and acceptor molecules (Figure 12C). Because of the quasi-continuum nature of the hole states in these QDs, there is a manifold of product states (QD + [*E*_h,*i*_] – A^*–*^) with the excited hole at different levels (*E*_h,*i*_). The total ET rate is the sum of Auger-assisted ET rates to these product states.
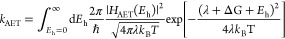
4.5

The electronic coupling strength in Auger-assisted ET model, *H*_AET_(*E*_h_), depends not only on the overlap of the 1S electron and acceptor orbitals (as in conventional ET) but also on the electron–hole Coulomb interaction.

According to the Auger-assisted ET model, the ET rates increase rapidly with the driving force at −Δ*G* < λ, which is similar to the conventional ET model (Figure 12B, left area). However, the ET rate continues to increase with driving force even when −Δ*G* > λ due to the presence of the continuum of product states, to which ET can occur with effective driving forces (−Δ*G*′= (−Δ*G*) – *E*_h_) ranging from 0 (hole excitation takes all the free energy change) to −Δ*G* (hole is not excited) (as shown in Figure 12D). The regime where −Δ*G* > λ is denoted as the Auger-assisted regime (Figure 12B, right area) because ET occurs most effectively with the excitation of holes, overcoming the unfavorable Franck–Condon overlap in the conventional Marcus inverted regime. As shown in Figure 12B, the Auger-assisted ET model can satisfactorily explain the experimental data, confirming that it is the appropriate model for ET from QDs.

The driving force effect on the hole transfer process from QDs to molecular acceptors has also been examined.^291^ Olshansky et al. measured hole transfer from photoexcited CdSe/CdS QDs to six ferrocene derivatives as hole acceptors, spanning a driving force range of 150–950 meV (Figure 12E). Similar to ET, the hole transfer rate increases with increasing driving force without an inverted regime. The authors explain their observation using an Auger-assisted mechanism in which the electron intraband excitation accompanies interfacial hole transfer process (Figure 12F). It should be noted that compared to the quasi-continuous hole states, the electron states in the conduction band are more sparsely spaced, which leads to the nonmonotonic dependence of the calculated hole transfer rate on the apparent driving force.

Auger-assisted charge transfer from QDs is fundamentally different from that in molecular donor–acceptor and bulk semiconductor–adsorbate complexes. In bulk semiconductors, the electron–hole interaction is weak and they behave as independent carriers. Although Coulomb interaction is strong in molecule, the density-of-electronic states is too sparse to enable an efficient Auger-assisted ET process. Furthermore, Auger-assisted ET only requires that electron–hole coupling is stronger than electron–phonon coupling and there is a large density of states, especially at high energy. Since these conditions can be readily met in excitonic nanomaterials (nanorods, nanosheets, and nanotubes), Auger-assisted ET should be a general model for charge transfer from these nanomaterials. It would be very interesting to experimentally verify the general applicability of this model beyond the observation in QDs discussed above.

Importantly, in a recent study, the conventional Marcus inverted region has been observed in charge transfer processes in nanocrystal-molecule complexes.^295^ The key to this observation is to measure charge transfer from the single-electron states of nanocrystals to surface-anchored molecules in the absence of strongly Coulomb-coupled holes. Specifically, through rational design of the energy level alignment in nanocrystal–molecule complexes, photoexcitation can create transient charge-separated states, for which the electrons are located in the conduction band of 0D QDs or 2D NPLs whereas the holes are in the surface-adsorbed molecules; the ensuing charge recombination processes were followed by transient absorption spectroscopy. By further tuning the electron transfer driving forces through the quantum confinement effect, this measurement unambiguously revealed a Marcus inverted region that had been hidden by the Auger-assisted mechanism. This result thus provides strong support to the Auger-assisted mechanism for charge transfer from excitonic states of these low-dimensional materials.

#### 4.1.5 Charge Transfer from QD to Metal Oxide Semiconductors with Continuous States

ET from a QD to TiO_2_, SnO_2_, and ZnO metal oxide (MO) semiconductor films has been extensively studied,^37,253,254,260,268,293,296^ in part due to its important role in QD photovoltaic devices. Previous studies on dye-sensitized oxide films have already shown that, because of the continuous accepting states in MO conduction bands, the conventional two-state (single donating state and single accepting state) Marcus ET model (eq 4.1) has to be extended to a many-state Marcus ET model by integrating over all possible accepting states.^252,297−299^
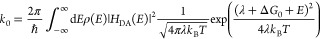
4.6Here ρ(*E*) is the density of accepting states in the metal oxide CB, including both bulk and defect states,^253,298^ and Δ*G*_0_ is the driving force for electron transfer to the band edge of MO.

**Figure 13:**
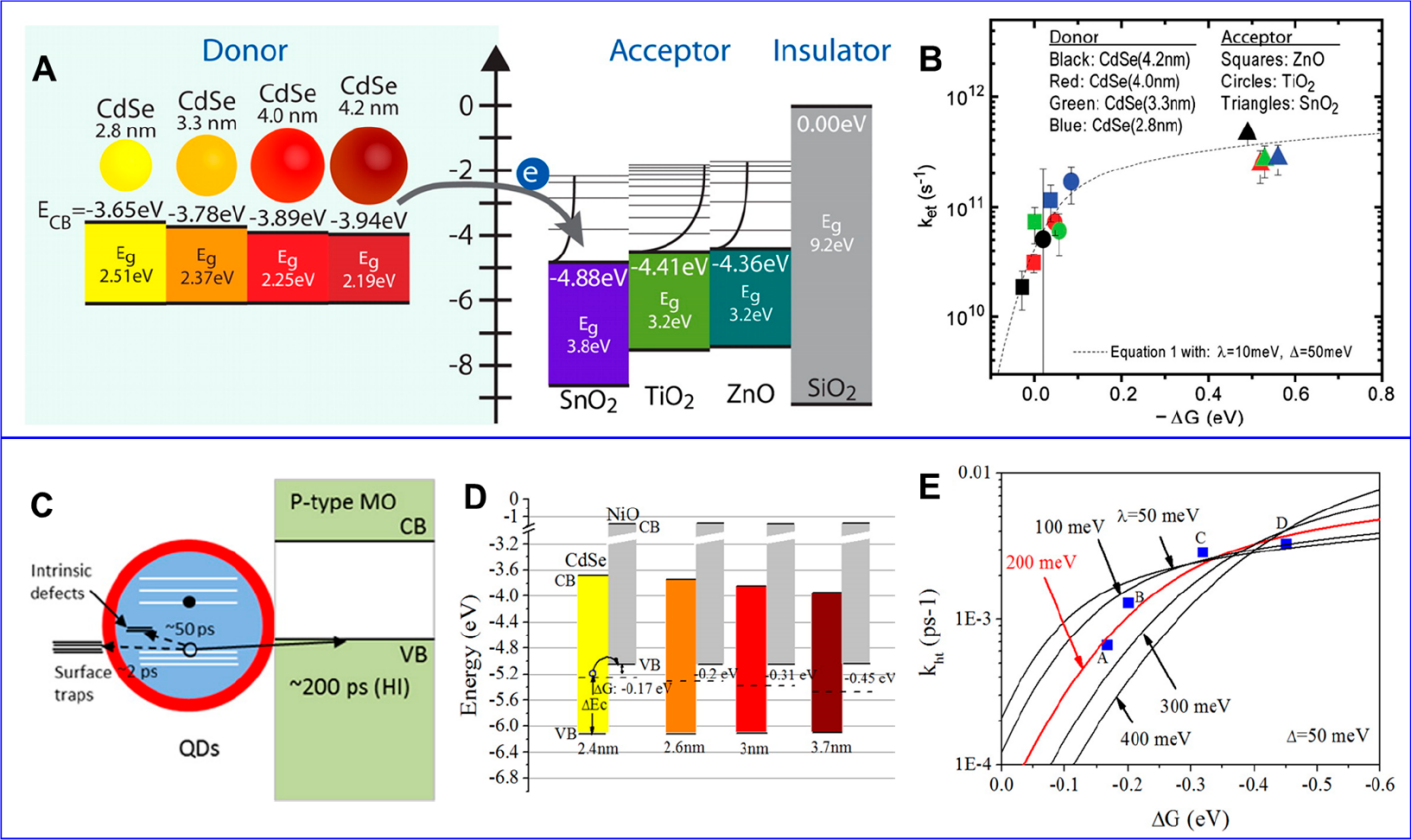
(A) Diagram of the relative electronic energy differences between CdSe donating species and MO accepting species for all CdSe–MO combinations. (B) Global plot of all CdSe (donor) to MO (acceptor) electron transfer data and trace of eq 4.6 with λ = 10 meV and Δ = 50 meV. (C) Scheme showing the hole transfer process when QDs are attached to a p-type hole acceptor semiconductor. (D) Energy alignment of the CdSe QD–NiO system of different QD sizes (2.5, 2.6, 3.0, and 3.7 nm). (E) Hole injection rate vs driving force of QDs attached to NiO (blue squares) and fitting curves (black and red lines). (A and B) Adapted with permission from ref (253). Copyright 2011 National Academy of Sciences. (C and D) Adapted with permission from ref (294). Copyright 2014 American Chemical Society.

To test this many-state ET model in QD-MO systems, Tvrdy et al. examined ET from CdSe QDs of four different sizes to different metal oxide films (SnO_2_, TiO_2_, and ZnO) (Figure 13A).^253^ With increasing −Δ*G*_0_, they found a sharp increase in ET rates at small driving forces, followed by a modest increase when the driving force significantly exceeds the reorganization energy. This trend can be well described by eq 4.6, as shown in Figure 13B. According to the many-state ET model, the ET rate is the sum of ET rates to all available states and is determined by the density of accepting states. When −Δ*G*_0_ < λ, accepting states are below and near the band edge, where density of states increases rapidly with driving force. At −Δ*G*_0_ > λ, the ET rate occurs to states above the band edge, the density of which varies slowly with increasing energy (scaling with). Similar ET behavior has been observed in CdSe QD-ZnO^293^ and PbS QD-SnO_2_^254^ systems, which have also been successfully described by eq 4.6.

Besides the more extensively reported electron transfer process, its counterpart, hole transfer to MO, has also been investigated by Zheng and co-workers using a CdSe QD as the donor and p-type NiO film as the hole acceptor (Figure 13C).^294^ The hole transfer from CdSe QDs to NiO was observed to occur on the time scale of a few hundreds of picoseconds. This HT rate is more than an order of magnitude slower than the ET rate, which could be attributed to the larger effective mass of holes, giving rise to a smaller surface hole density and hence the HT coupling strength. The competition between hole transfer and hole trapping on similar time scale leads to low HT yield from CdSe QDs. The authors tuned the HT driving force by changing the size of CdSe QDs (Figure 13D). The HT rate increases with the driving force, which can also be well described by eq 4.6 with a relatively large reorganization energy of 200 meV (Figure 13E). Due to the complex electronic states and dynamics of holes in CdSe QDs, hole transfer process from a QD might deviate from simple direct transfer mechanism, as will be discussed in the next section.

It should be noted that eq 4.6 predicts a driving force dependence that is similar to the Auger-assisted ET, but the fundamental origins are quite different. For ET in QD-MO, the continuum of products states comes from a continuum of acceptor electronic levels (*i.e.*, CB of MO), while in QD–molecule complexes it is caused by the quasi-continuum in the donor hole levels. As discussed above, the Auger-assisted ET pathway significantly enhances the ET rate in the inverted regime; since the inverted regime is not present in QD-MO, the effect of Auger-assisted pathways may not be significant.

#### 4.1.6 Direct vs Trap-Mediated Hole Transfer

**Figure 14:**
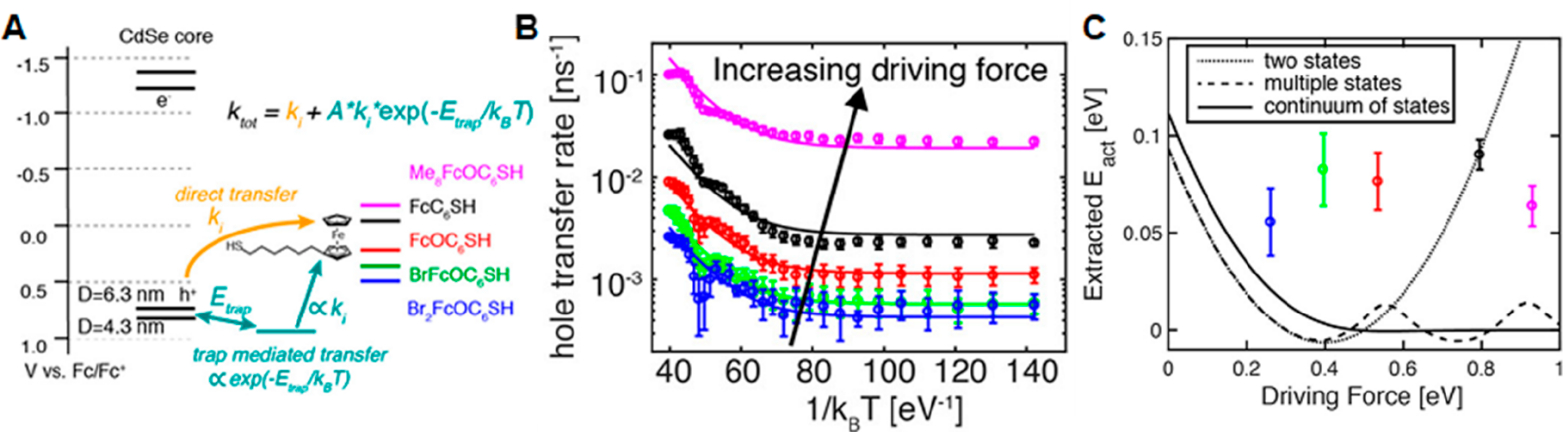
(A) Schematic of the model that assumes the charge transfer rate is a sum of two pathways: direct transfer and trap-mediated transfer. (B) Arrhenius plots for the hole transfer rate from 6.3 nm QDs to five ferrocene molecules with fits to the trap-mediated model. (C) Driving force dependence of extracted effective activation energies (Arrhenius slopes from the high-temperature regime) for HT from 6.3 nm QDs to five ferrocene molecules and that of direct transfer models assuming two states (conventional Marcus model) or multiple final states (Auger-assisted mode). Adapted with permission from ref (292). Copyright 2017 American Chemical Society.

The discussions above on the QD CT process assumes electron (hole) transfers directly from well-defined lowest energy 1S state in conduction (valence) band, which is a reasonable assumption for electrons in cadmium chalcogenide QD and holes in high quality core/shell QDs. In reality, due to the presence of ill-defined and undercoordinated surface atoms, carriers, especially holes, in a cadmium chalcogenide QD are known to undergo reversible/irreversible traps,^300,301^ which could affect the charge transfer pathways and dynamics. To differentiate the direct transfer vs trap-mediated transfer process, Olshansky et al. examined the HT process from CdSe/CdS core/shell QDs to different ferrocene hole-accepting molecules as a function of temperature (Figure 14A). Overall, the HT rate increases with temperature for all acceptors and all QDs. The Arrhenius plot of hole transfer rates exhibits an activated regime at higher temperatures and a weak temperature-dependent regime at low temperatures (Figure 14B). The extracted activation energies in the high-temperature regime are almost the same for a given QD, regardless of hole acceptors or driving force. This constant activation energy across all driving forces (100–900 meV) contradicts the activation energy in the Marcus model assuming direct hole transfer from the QD valence band to hole acceptors (Figure 14C). Based on the temperature-dependent results, the authors proposed a model where, besides direct hole transfer from valence band, an additional dominant hole transfer pathway is through a shallow and reversible trap (Figure 14A). The latter relies on thermal excitation of the hole into a trap state higher than the valence band edge and is therefore temperature-dependent. This model contrasts with simple direct transfer mechanism that is often assumed and necessitates a careful study on charge transfer process. It should be noted that only reversible traps that can contribute to fluorescence are investigated here, while irreversible traps, which lead to nonradiative recombination, are neglected in this study.

### 4.2 Adiabatic Charge Transfer from QD in the Strong Coupling Regime

For QDs with large exciton Bohr radii, such as PbS (∼20 nm) and PbSe (∼46 nm), a large portion of the wave functions of electrons and holes extends outside the QD due to the strong quantum confinement.^302^ When these QDs are attached directly or through a short bridge to bulk semiconductors such as TiO_2_, the electronic coupling can be strong and electron transfer from the QD to semiconductors can fall in the adiabatic regime.^137,296,303^ One consequence of the strong electronic coupling is the broadening of the discrete electronic state in QDs resulting from mixing with the continuum states in semiconductors. The Newns–Anderson model for chemisorption can be used to describe this spectral broadening.^304,305^ The density of states (ρ) of the broadened electron level in QDs as a function of energy *E* is given by
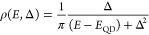
4.7where *E*_QD_ is the energy of a discrete QD state prior to broadening and Δ is the half width of the broadened level and is related to the coupling strength. According to the Heisenberg uncertainty principle, a homogeneous spectral line with a full width at half-maximum of 2Δ corresponds to a lifetime of τ = ℏ/2Δ of the electron in this broadened level. This electron lifetime reflects the electron packet propagation time between the strongly coupled QD state and the TiO_2_ CB, or the adiabatic electron transfer time.

**Figure 15:**
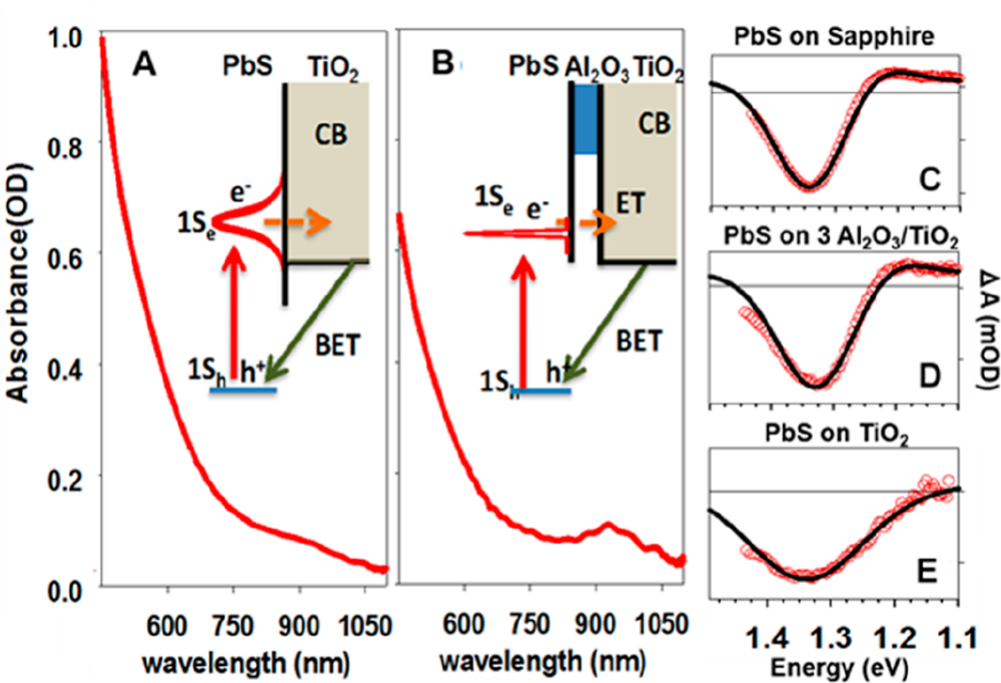
(A and B) Static absorption spectra of PbS QD on TiO_2_ (A) without and (B) with an insulating Al_2_O_3_ spacer. The inset shows a schematic depiction of the varying degrees of broadening of the 1S electron level as a result of interaction with the TiO_2_ CB. (C–E) Simulated (black lines) and measured (red circle) 1S exciton bleach spectra for PbS QDs (C) on sapphire windows, (D) on 3Al_2_O_3_/TiO_2_, and (E) on TiO_2_. Adapted with permission from ref (137). Copyright 2010 American Chemical Society.

Yang et al. reported the spectral broadening due to strong electronic coupling and ultrafast electron transfer from PbS QDs to a TiO_2_ semiconductor film.^137^ As shown in Figure 15A and B, the static absorption spectra of PbS QDs adsorbed directly on TiO_2_ show a broadened 1S exciton band, and the extent of broadening decreases significantly in the presence of an insulating Al_2_O_3_ spacer layer between the QD and TiO_2_. This spectral broadening can be more clearly seen from the transient absorption spectra (Figure 15C–E). Compared to QDs on a sapphire substrate, the 1S exciton peak for PbS QDs on TiO_2_ films is significantly broadened. Inserting a relatively thick Al_2_O_3_ insulating layer between PbS QDs and TiO_2_ mostly recovered the peak width, indicating decreased electronic coupling with the Al_2_O_3_ insulating layer. Fitting these spectra reveals homogeneous broadening (and electron transfer lifetime) for the QD on TiO_2_ of ∼49 meV (∼6.5 fs). Time-resolved measurements showed that the ET time from QD to TiO_2_ was faster than the instrument time resolution (∼150 fs), in line with the results obtained from the spectral broadening. The adiabatic electron transfer from PbSe QDs to TiO_2_ (with ET time of ∼10 fs) has also been theoretically predicted.^306^ This ultrafast ET rate suggests the possibility of extracting hot electron before electron cooling, which will be discussed in the next section.

It should be noted there are a wide range of reported ET times from lead chalcogenide QDs to TiO_2_. Wise and co-workers found that the ET from PbS QDs to TiO_2_ colloidal particles was on the time scale of several to tens of ns.^260,281^ The reason for the different coupling strength was unclear, which could be due to sensitive dependence of the coupling strength on the interfacial ligand structure.

### 4.3 Hot Electron Transfer from Nanocrystals

**Figure 16:**
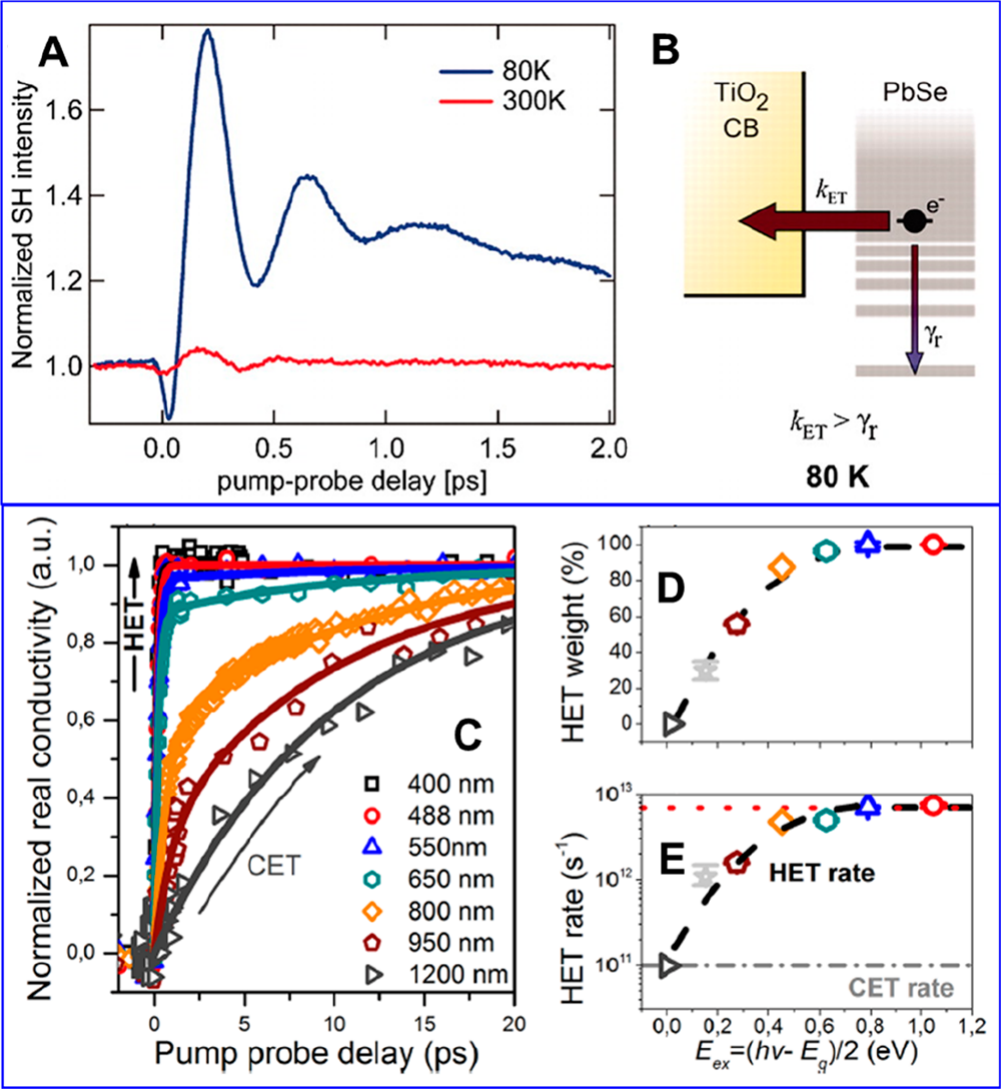
(A) Time-resolved second harmonic response of the TiO_2_ surface coated with 1.5 monolayers of hydrazine-treated 3.3 nm PbSe nanocrystals. The large increase in the SHG intensity is indicative of efficient electron transfer from PbSe to TiO_2_. (B) Illustration of the competing pathways of interfacial electron transfer and intra-quantum dot relaxation. (C–E) Photon-energy-dependent hot electron transfer (HET) from PbS QDs to SnO_2_ probed by time-resolved THz spectroscopy. (C) Excitation-wavelength-dependent electron transfer (ET) dynamics (from 400 to 1200 nm) for PbS QDs with ∼2.7 nm diameter. The solid lines are biexponential fits based on hot and cold electron transfer (HET on sub-ps time scales and CET on a ∼10 ps time scale; see arrows). (D) The weight of the fast HET component in the dynamics shown in panel C vs hot electron excess energy in the QDs. (E) HET rates vs the excess energies of hot electrons. The CET rate was found to be independent of the excess energy and fixed to 10.2 ps (gray dotted-dash line). In panels D and E, the dashed black lines are to guide the eye; the red dotted line represents the time resolution of our setup. (A and B) Adapted with permission from ref (303). Copyright 2010 American Association for the Advancement of Science. (C–E) Adapted with permission from ref (307). Copyright 2018 American Chemical Society.

The discussions above mostly focus on electron/hole transfer from lowest-energy 1S state, as hot carrier cooling in typical QDs occurs usually on an ultrafast (<300 fs) time scale through Auger-type energy transfer between electrons and holes and/or to ligand vibrations and phonons.^308,309^ Hot carrier cooling represents one of the major efficiency losses in solar energy conversion devices, leading to the so-called Shockley–Queisser limit. The ultrafast adiabatic electron transfer from QDs to semiconductor films in a strong coupling regime discussed above suggests the possibility of extracting hot carrier before thermalization (Figure 16B), which potentially can overcome the Shockley–Queisser limit and lead to hot carrier solar cell with 66% theoretical efficiency. Indeed, Tisdale et al. observed hot ET in PbSe QD-sensitized TiO_2_ (with an ET time of ∼31 fs) using time-resolved second harmonic generation (SHG) spectroscopy at 80 K (Figure 16A).^303^ Negligible hot ET was observed at room temperature, presumably due to faster electron cooling at higher temperatures. Using CdSe QDs as electron acceptors, Grimaldi et al. also observed fast hot ET from PbSe QDs to CdSe QDs assembled in a quantum dot heterojunction solid.^310^ The hot ET efficiency increases with excitation energy and reaches ∼4.5% when the excitation photon energy approaches the onset of CdSe absorption. Recently, Wang et al. directly nucleated PbS QDs onto SnO_2_ films and performed a comprehensive study of hot ET from PbS QDs to SnO_2_. They observed hot ET up to unity quantum efficiency from PbS QDs at room temperature.^307^ As shown in Figure 16C, both hot (<150 fs) and cold ET (∼10 ps) were observed, depending on excitation photon energy, giving rise to the nonsingle exponential kinetics. The hot ET amplitude (Figure 16D) and rate (Figure 16E) increase with the excitation photon energy, *i.e.*, excess energies of hot electrons, due to the increased density of states of both the donor and acceptor at higher energies. The authors also reported enhanced hot ET efficiency by increasing the QD size or by lowering the temperature. The hot ET efficiency depends on the kinetic competition between the hot ET rate (*k*_ET_) and the hot electron thermalization rate (γ_r_) in QDs.

Hot ET has also been observed in perovskite nanocrystal–molecular acceptor systems.^311,312^ Li et al. investigated the hot electron cooling and extraction from colloidal MAPbBr_3_ perovskite nanocrystals and observed room temperature hot electron extraction (with efficiency up to 83%) from a colloidal MAPbBr_3_ nanocrystal film to a 4,7-diphenyl-1,10-phenanthroline hot-electron extraction layer within 0.2 ps.^311^ Using time-resolved terahertz spectroscopy, Sarkar et al. measured the electron and hole transfer process from a CsPbBr_3_ nanocrystal to surface adsorbed benzoquinone and phenothiazine molecules as electron and hole acceptors, respectively.^312^ They observed a major ultrafast hot carrier transfer channel within their time resolution (<300 fs) and a secondary cooled carrier transfer process to molecular acceptors on the order of tens to hundreds of ps. These proof-of-concept studies on model systems suggest the possibility of employing colloidal nanocrystals to extract hot carriers and circumvent energy loss in solar energy conversion applications, but it still remains challenging to implement the hot carrier extractions in real devices, as the light harvesting layer, the energy selective contacts, and the interface have to be carefully designed and engineered.

### 4.4 Electron Transfer from 2D Nanocrystals: The Effect of Dimensionality

**Figure 17:**
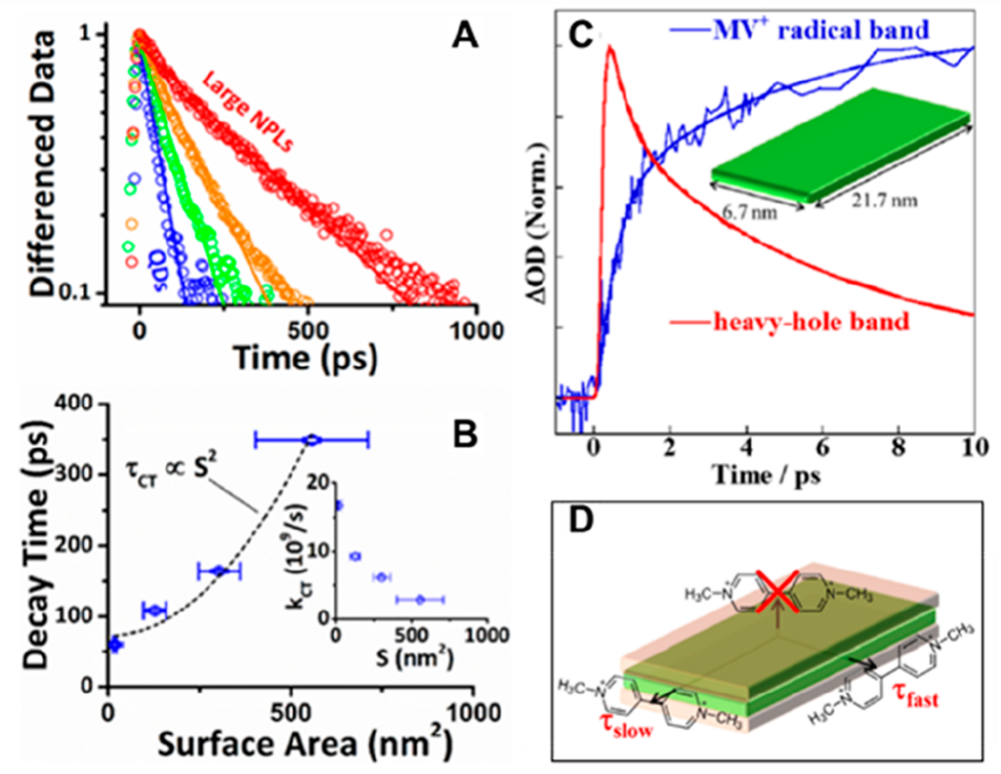
(A) The time-resolved PL decay kinetics of CdSe NPLs with four different lateral areas (circles). The solid lines are the exponential fits to the kinetics. (B) The PL quenching lifetime (1/*k*_CT_) as a function of the CdSe NPL lateral area. The inset shows the PL quenching rate (*k*_CT_) as a function of the NPL lateral area (S). (C) The transient absorption kinetics of exciton bleach (red curve) and MV^+^ radical (blue curve). The inset shows the lateral size of the CdSe NPLs. (D) Scheme of surface-dependent MV^2+^ adsorption on CdSe NPLs. (A and B) Adapted with permission from ref (313). Copyright 2016 American Chemical Society. (C and D) Adapted with permission from ref (314). Copyright 2016 American Chemical Society.

The ET mechanism in 2D nanocrystals, in particular, 2D CdSe NPLs, has also been studied. In 0D QDs, because of the quantum confinement in all three dimensions, the electron and hole are completely localized. 2D NPLs have unconfined lateral dimensions extending to tens to hundreds of nm. This increased lateral dimension brings the question of how dimensionality affects the ET rate from 2D NPLs. Schaller and co-workers reported a lateral-area-dependent ET from CdSe NPLs to MV^2+^.^313^ They used time-resolved PL quenching of CdSe NPL-MV^2+^ complexes to characterize the ET time (Figure 17A), which is shown to scale with the square of the NPL lateral area (S^2^) (Figure 17B). They explained this experimental result by assuming that the ET rate is proportional to the square of the product of wave functions for the charge-separated state (Ψ^CT^) and initial state (Ψ^S^). The former (Ψ^CT^) is independent to NPL lateral area considering the transferred electron is fully localized on MV^+^ radical, while the in-plane normalization factor in the latter results in an area dependence (Ψ^S^ ∝ 1/*S*). As a result, the ET time scales with the reciprocal of *S*^2^. This mechanism has assumed that the exciton center-of-mass wave function extends throughout the whole NPLs. However, the exciton center-of-mass coherent area (∼160 nm^2^) may be smaller than the NPL lateral area^248^ as discussed in section 3.2, in which case the fast exciton in-plane diffusion may come into play, but the relevant studies are still lacking.

Tamai and co-workers proposed a different mechanism for lateral-area-dependent ET from CdSe NPLs to MV^2+^ based on a transient absorption spectroscopic study.^314^ They observed biexponential ET kinetics from both the exciton bleach and MV^+^ radical kinetics (Figure 17C) and found slower ET rates from larger NPLs. They attributed this size-dependent ET rate to the size-dependent electronic coupling between CdSe and MV^2+^. It is suggested that MV^2+^ molecules selectively adsorb on the edges of NPLs, while the basal plane of NPLs is well passivated with oleic acid (Figure 17D). In this case, the higher amount of adsorbed MV^2+^ on the longer edges give to a faster ET rate compared to that in the shorter edges. Although this proposed mechanism explains the fast and slow components of biexponential transient absorption kinetics, which represent the ET from long and short edges of NPLs, respectively, the direct evidence of how MV^2+^ adsorbed on NPL surfaces is still lacking.

These reports indicate that the dimension change of NCs affects the ET mechanism in two ways: (1) the exciton in-plane motion, which includes center-of-mass coherent delocalization and diffusion, and (2) anisotropic adsorption of acceptors on NC surfaces. Both aspects require more extensive studies for further understanding of ET mechanisms in 1D and 2D NCs.

## 5 Multiple Exciton Generation and Dissociation from Nanocrystals for Generation III Photovoltaics

### 5.1 Theoretical Interpretations of Multiple Exciton Generation

In the bulk semiconductors, a carrier with sufficiently high kinetic energy can lose its excess energy by promoting a bound electron from the valence band to the conduction band via multicarrier Coulomb interactions, and this process is rationalized by impact ionization (II). Analogous to their bulk counterparts, quantum-confined nanocrystalline semiconductors can also generate multiple excitons by absorbing a single high-energy photon, known as multiple exciton generation (MEG) or carrier multiplication (CM). Several mechanisms have been proposed to interpret this phenomenon,^315−318^ and three of them will be highlighted here.

**Figure 18:**
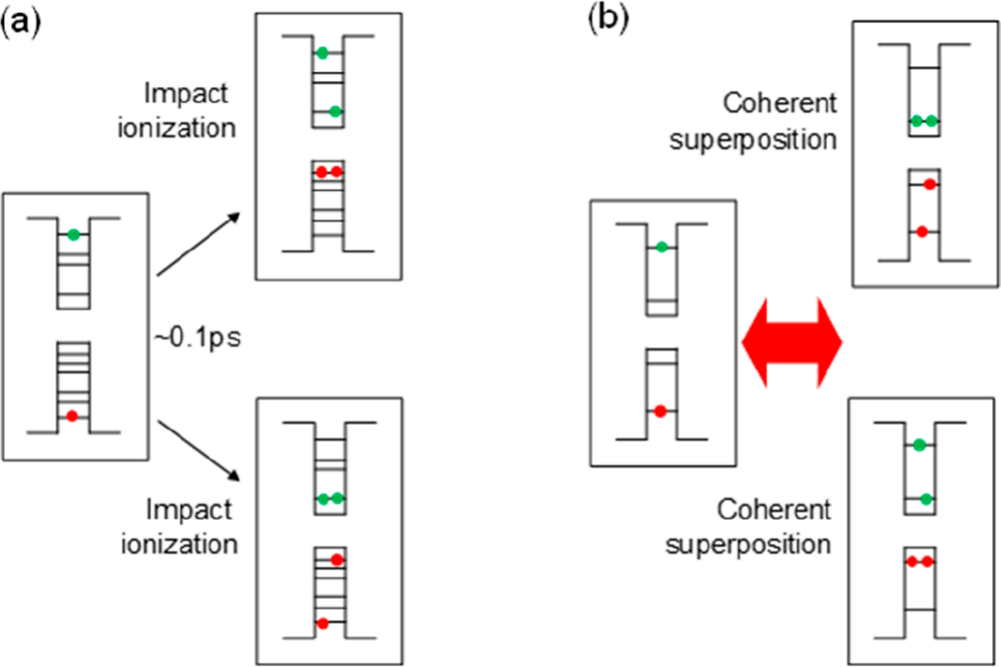
Proposed multiple exciton generation mechanisms. (a) The impact ionization of carriers with high kinetic energy create an additional electron–hole pair. (b) The Coulomb interaction creates a coherent superposition between a single exciton state and multiple exciton states.

According to Franceschetti et al., II can be used to explain the MEG process,^315^ predicting its rate and threshold. As shown in Figure 18A, the photogenerated high-energy single exciton can result in two biexciton states (with a hot electron or hot hole) via II. In this physical picture, II must compete with the high-energy single-exciton relaxation via phonon emission, which is on a time scale of few ps for lead chalcogenide QDs. According to the Fermi–Golden rule, the II rate is proportional to the transition probability between the initial (single exciton state) and final states (biexciton state), and this transition is induced by the carrier Coulomb interaction, which is treated as a perturbation. Taking into account the density of states (DOS) and selection rules, the computational results indicate that the II rate increase nearly drastically as the energy increases due to the steep growth of the DOS of the final states. When the energy reaches 2.1-fold of the band gap energy (2.1*E*_g_), the II rate surpasses the biexciton Auger recombination rate that is nearly energy-independent, and when the energy is larger than 2.7*E*_g_ the former is approximately two orders of magnitude larger than the latter, which is also comparable to hot carrier cooling rate. Therefore, the II rate calculation suggests that II rate may account for the MEG rate at high energy. To satisfy energy conservation, the II process in QDs only requires either the electron or hole in the single exciton with excess energy (energy above the respective band edge) larger than *E*_g_. As indicated in Figure 18A, the energy levels in conduction and valence bands for PbSe QDs were believed asymmetric rather than “mirror-like”, and the absorbed photon with energy in the range of 2–3*E*_g_ could distribute its energy primarily to the electron so that the electron gains excess energy larger than *E*_g_. Thus, the threshold of MEG also falls in the range of 2–3*E*_g_. Based on this model, the MEG threshold for PbSe QDs with a 3.1 nm diameter is predicted as 2.2*E*_g_. Note that the II rate at the threshold energy may not compete with the carrier relaxation, resulting in a negligibly small MEG efficiency.

According to Shabaev et al., MEG in QDs can also be explained by the optical excitation of a coherent superposition between isoenergetic states, single and multiple exciton states, that are strongly coupled by Coulomb interactions.^316^ Briefly, the Coulomb coupling among the single and multiple exciton states can no longer be treated as a perturbation when these states have the same energy and thus the eigenstates of the Hamiltonian including this Coulomb coupling will be mixed states of the single and various multiple exciton states or a superposition between these states. The coherence cannot be formed among nonresonant states due to the weak coupling. As shown in Figure 18B, a photon with energy in the range of 2–3*E*_g_ will only create superpositions between a single exciton state and biexciton states because of the energy conservation. The superposition is damped due to the thermalization of either the single or the biexciton components with thermalization rate of γ_1_ and γ_2_, respectively. In the case of strong coupling, the oscillation frequency (*γ*_*c*_) between the two coherent states is much higher than both γ_1_ and γ_2_ and thus the MEG efficiency is determined by the competition of the two damping channels, with a larger ratio of γ_2_/γ_1_ resulting in a higher MEG efficiency. In the case of weak coupling, when *γ*_*c*_ ∼ γ_2_, the MEG efficiency can still be high if γ_2_/γ_1_ ≫ 1. However, when *γ*_*c*_ ≪ γ_2_, γ_1_, optical excitation of a single exciton, instead of superposition, is dominant, and the MEG efficiency will be negligibly small. Assuming a mirror-like conduction and valence bands for lead chalcogenide QDs, the energy of the single exciton state has to be higher than that of the 2P exciton in order to find resonant biexciton states with the same energy. Thus, the threshold of MEG in these QDs has been predicted as ∼3*E*_g_. It should be noted that if the valence band has a higher DOS than conduction band near band edge then the MEG threshold should be lower than 3*E*_g_.

In addition, Klimov and co-workers have proposed a model of direct photogeneration of multiexcitons by a single photon absorption via virtual single-exciton states.^317^ Similar to the indirect optical transitions in bulk semiconductors, photoexcitation of multiexciton states is also forbidden because of momentum conservation.^317^ However, according to second-order perturbation theory, the Coulomb interaction between multiple exciton states and virtual single exciton states can give a nonvanishing oscillator strength for the direct multiple exciton state excitations with the assistance of virtual single exciton states. In contrast to the dephasing of coherent superposition model, this perturbation approach allows low or moderate coupling strength between the single and multiple exciton states.

### 5.2 Statistical Description of the Multiple Exciton Dynamic Behavior

**Figure 19:**
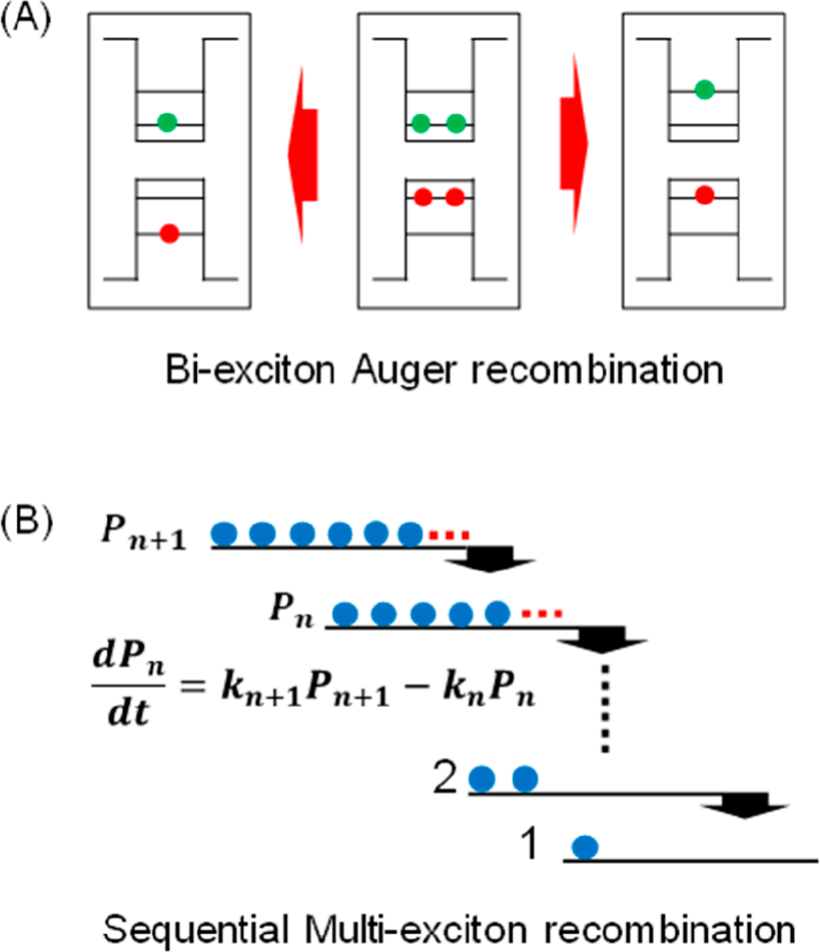
(a) Illustration of biexciton Auger recombination. (b) Illustrative scheme of cascade Auger recombination in QDs.

To characterize MEG, understanding of multiple exciton dynamics in quantum-confined systems is required. Furthermore, this knowledge is also desirable for the development of QD-based optoelectronic applications such as LEDs and lasers. When a semiconductor nanocrystal has multiple electron–hole pairs (or excitons), one of the electron–hole pairs can recombine by promoting a third charge carrier to a higher energy level due to the Coulomb interaction, known as Auger recombination, which is usually more efficient than the radiative recombination (Figure 19A). In bulk and nanocrystalline semiconductors with low exciton binding energies, Auger recombination occurs via three-particle-involved Coulomb interactions, which are responsible for the third-order recombination channel.^319^ In nanocrystals with strong exciton binding energies, Auger recombination occurs between two excitons.^151,320^ Thus, the different Auger recombination mechanisms give different recombination rate scaling laws with the number of exciton or electron–hole pairs (*n*): *k*_*n*_ ∝ *n*^2^(*n*–1) and *k*_*n*_ ∝ *n*(*n*–1) for three-particle and two-particle (or bimolecular) mechanisms, respectively.^92,321−324^

After optical excitation, the initial distribution of exciton number per dot in a QD ensemble is governed by the Poisson equation:^208^
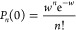
5.1where *P*_*n*_(0) is the probability of QDs with *n* photogenerated excitons per dot and *w* is the average exciton number per dot. As shown in Figure 19B, the multiexcitons in a QD decay sequentially via Auger recombination. As a consequence, the statistical distribution of excitons in QDs will vary as a function of time after optical excitation. The kinetics of *P*_*n*_ will be described by
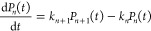
5.2where *k*_*n*+1_ and *k*_*n*_ are the decay rate constant for *n* + 1 and *n* exciton states, respectively, which should obey the aforementioned Auger recombination rate scaling law. Therefore, the average multiple exciton annihilation (MEA) kinetics for a QD ensemble can then be simulated by combining a complete set of coupled eq 5.2 with an initial condition defined by eq 5.1.

**Figure 20:**
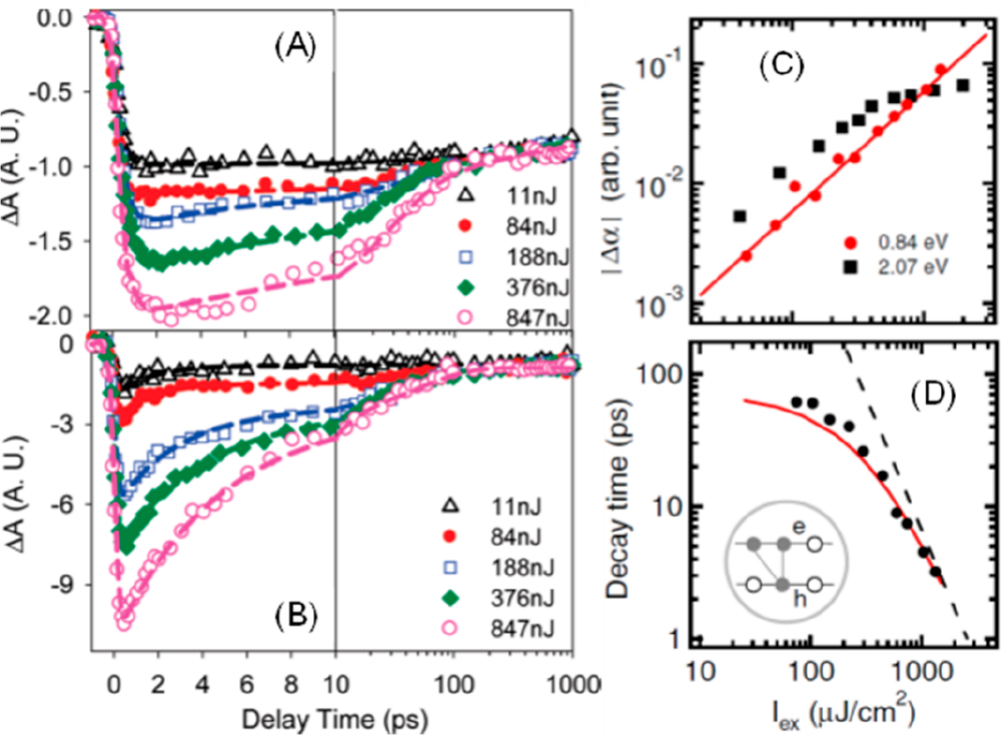
Comparison of (A) 1S and (B) 1P exciton bleach recovery kinetics at different pump intensities obtained from TA measurement of CdSe QDs. The kinetic traces have been normalized to the same value at a 1 ns delay time. (C) Maximum amplitude of the photoinduced absorption signal (0.84 eV) and 1S exciton bleach signal (2.07 eV) as a function of pump intensity for CdSe QDs. (D) Auger decay time extracted by probing the kinetics of photoinduced absorption signal as a function of the excitation intensity. The red curve shows the calculated result using the quantized Auger recombination model with three-carrier collisions. (A and B) Adapted with permission from ref (325). Copyright 2010 American Chemical Society. (C and D) Adapted with permission from ref (326). Copyright 2009 The Physical Society of Japan.

Although the photon energy of visible light is below the threshold of MEG for CdSe QDs, multiple excitons can still be generated by the absorption of multiple photons per dot under high-intensity optical excitation. The average number of excitons per dot (*w*) is proportional to the excitation intensity. The dependence of the first exciton (1S) bleach recovery kinetics of CdSe QDs on excitation intensity is displayed in Figure 20A.^325^ The kinetics traces have been scaled by normalizing their amplitude at 1 ns in order to emphasize the MEA component. The larger magnitude of the fast component for higher pump intensity is due to the greater fraction of dots with multiple excitons. Before ∼200 ps, the kinetics show a fast decay component, attributed to MEA via Auger recombination. After ∼200 ps, all the kinetic traces show negligible decay, indicating the completion of MEA and the resultant long-lived single excitons. Since the 1S bleach in CdSe QD TA spectra mainly arises from the state filling of the two-fold degenerate 1S electron level, QDs with multiple excitons (*n* ≥ 2) all contribute equally to 1S exciton bleach amplitude, and the fast decay component only reflects the biexciton annihilation rate. Thus, these kinetic traces can be fitted by the following equation:
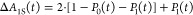
5.3where the first and second terms on the right side represent the contributions from QDs with multiple excitons (*n* ≥ 2) and a single exciton. Because the biexciton recombination rate constant (*k*_2_) is orders of magnitude larger than that for single excitons (*k*_1_), the differential rate equation set (eq 5.2) for one and two excitons can be approximately decoupled, and eq 5.3 is then expressed as two isolated exponential components:
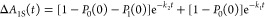
5.4where the first and the second terms on the right side account for the decay caused by biexciton and single exciton recombinations, respectively. The prefactors consist of the initial Poisson distribution of QDs, which constrains the relative amplitudes of the two decay components. This equation suggests that the QDs with at least two excitons contribute to the biexciton decay and those with at least one exciton (also including those with multiple excitons) contribute to the single exciton decay. *k*_2_ given by this fitting is consistent with reported values for CdSe QDs. *k*_2_^–1^ has also been shown to be size-dependent and increases nearly linearly with the QD volume. As mentioned before, the 1S bleach signal for CdSe QDs is not sensitive to the occupation of higher exciton states and thus the higher order MEA (*n* > 2) should have negligible contribution to the 1S bleach recovery kinetics. Nonetheless, Klimov et al. have reported the higher-order multiexciton state (*n* = 2, 3, 4) lifetimes by successive subtraction of carrier decay kinetics under different pump intensities.^30^ The carrier kinetics was converted from 1S exciton bleach recovery kinetics through a phenomenological fitting equation. In principle, when *n* > 2, the electrons start to populate the 1P electron levels, leading to the bleach of the 1P exciton transition. Indeed, additional fast decay components have been observed in the 1P bleach recovery kinetics, as shown in Figure 20B, from which an averaged lifetime of n (*n* > 2) exciton states was estimated to be 2.6–4.5 ps.^325^ A more detailed analysis was hindered by the presence of other TA signals, such as the multicarrier interaction-induced spectral shift. Kanemitsu and co-workers reported that a photoinduced absorption (PA) signal for CdSe QDs in the near IR region of TA spectra is proportional to the pump intensity.^326^ This linear relationship holds even in a high intensity region where 1S bleach signal magnitude is saturated (Figure 20C), implying that the kinetics of PA may include the higher-order MEA information. The analysis the PA kinetics for different intensities based on the Poisson statistical model reveals the time constant of the MEA process in CdSe QDs, which is well described by the three-particle Auger recombination model (Figure 20D).

The three-particle Auger recombination model works for free carriers (electron and hole) with small binding energies that are strongly confined in a small region as in QDs. In 1D NRs and 2D NPLs, where dielectric confinement effects enhance the exciton binding energy as discussed in section 2, excitons rather than free carriers dominate at the band edge. In this case, the recombination tends to happen between the bound electron–hole pairs following the bimolecular Auger recombination model. Using similar transient spectroscopic methods, bimolecular Auger recombination has been reported by Klimov and co-workers for PbSe NRs^324,327^ and Siebbeles and co-workers for both CdSe only and CdSe/CdS/ZnS core/shell NPLs.^246^

**Figure 21:**
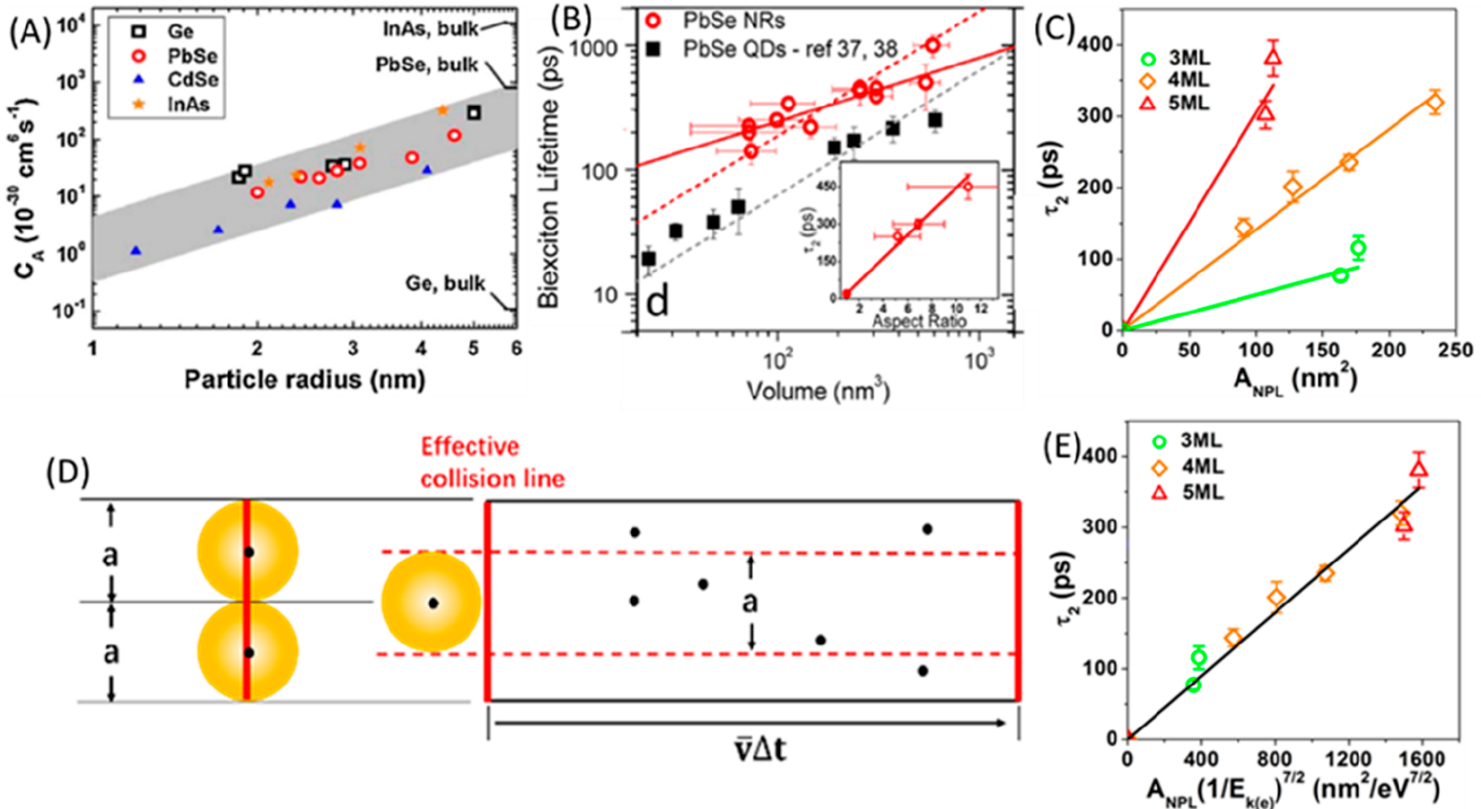
(A) Auger constants of different QDs as a function of the QD radius. (B) The biexciton lifetimes of PbSe QDs (black squares) and NRs (red circles) as a function of the NC volume. (C) The biexciton lifetime of CdSe NPLs with different thicknesses and lateral areas as a function of the NPL area (*A*_NPL_). The solid lines are the linear fits of NPLs with the same thickness. (D) Scheme of the classic 2D biexciton collision model. *a* is the exciton diameter, *v̅* is the mean velocity of exciton in-plane motion, and Δ*t* is the time spacing between collisions. (E) The biexciton lifetime of CdSe NPLs with different thicknesses and areas as a function of the product of the NPL area (A_NPL_) and the quantum-confined energy of the electron (*E*_k(e)_) to the order of −7/2. (A) Adapted with permission from ref (328). Copyright 2009 American Physical Society. (B) Adapted with permission from ref (327). Copyright 2013 American Chemical Society. (C–E) Adapted with permission from ref (329). Copyright 2017 American Chemical Society.

There are mainly three ways to control the MEA rates: (1) NC size, (2) electron–hole wave function overlap, and (3) confinement potential abruptness. Klimov et al. first reported that the Auger constant (*C*_A_) of CdSe QDs scales with the QD volume.^30^ Following this pioneering work, the Auger constants of other QDs, with both direct and indirect band gap such as PbSe, InAs, and Ge QDs, have been reported to scale with QD volume, showing a “universal volume scaling law” (Figure 21A).^328,330−332^ The *N*-exciton Auger recombination lifetime (τ_*N*_) follows , where *V* is the QD volume and *R* is the QD radius, so that the Auger lifetime of QDs also scales with QD volume.^30,328^ Note that this assumes a continuous carrier density in QDs similar to bulk materials, which is not accurate because the recombination occurs as sequentially quantized steps in QDs with only several electron–hole pairs.^333^ Although the insight of this “universal volume scaling law” observed in QDs remains unclear, it shows that the Auger recombination in QDs is significantly different from that in bulk materials. Auger recombination in indirect band gap bulk semiconductors is orders of magnitude slower than that in their direct band gap counterparts due to the requirement of momentum conservation, while this momentum conservation is relaxed by strong quantum confinement in QDs.^328^ Biexciton lifetimes of 1D PbSe NRs have also been reported to scale with the NR volume following this “universal volume scaling law” (Figure 21B).^334,335^

The volume-scaled Auger lifetime in 1D NRs is surprising because 1D NRs contain both quantum-confined and non-quantum-confined dimensions and the volume-scaled Auger lifetime indicates these two different dimensions contribute equally to Auger recombination in NRs. Heinz and co-workers reported an exciton-collision-based Auger recombination model for 1D carbon nanotubes and showed the Auger lifetime scales with nanotube length.^336^ Recently, using CdSe NPLs with well-controlled lateral area and thickness, Lian and co-workers studied the area- and thickness-dependent Auger recombination in CdSe NPLs and found that the biexciton Auger lifetime does not scale with NPL volume, breaking the “universal volume scaling law”.^329^ Instead, the biexciton Auger lifetime scales linearly with the NPL lateral area for NPLs with the same thickness and increases dramatically in thicker NPLs (Figure 21C). A new two-step Auger recombination model is proposed by Lian and co-workers for 2D NPLs: (1) excitons diffuse and collide to each other frequently within NPLs due to the fast exciton in-plane transport^212,221^ and (2) each collision has a finite probability for Auger recombination.^337^ The lateral area dependence is due to the binary exciton collision (Figure 21D): the exciton in the middle of the effective collision line, within time interval Δ*t*, can collide with any excitons whose center (black dots) are in the collisional square so that the collision frequency (*F*_C_) scales with the reciprocal of the NPL lateral area. The Auger probability is considered similar to that reported in similar quantum wells and is determined by the quantum-confined energy of electrons in the conduction band (*E*_*k*(e)_).^338^ This two-step Auger recombination model fits the biexciton Auger lifetime of CdSe NPLs excellently (Figure 21E) and may also work for other 1D and 2D nanomaterials. These size-dependent Auger recombination studies have clearly shown that the Auger rates of NCs can be tuned by their sizes and dimensions. For example, biexciton lifetime in CdSe NPLs (hundreds of ps)^329,339^ is much longer than that in QDs (tens of ps);^30^ in particular, the biexciton lifetime in 2D NPLs can be extended to several ns by increasing the thickness through shell coating.^340,341^

**Figure 22:**
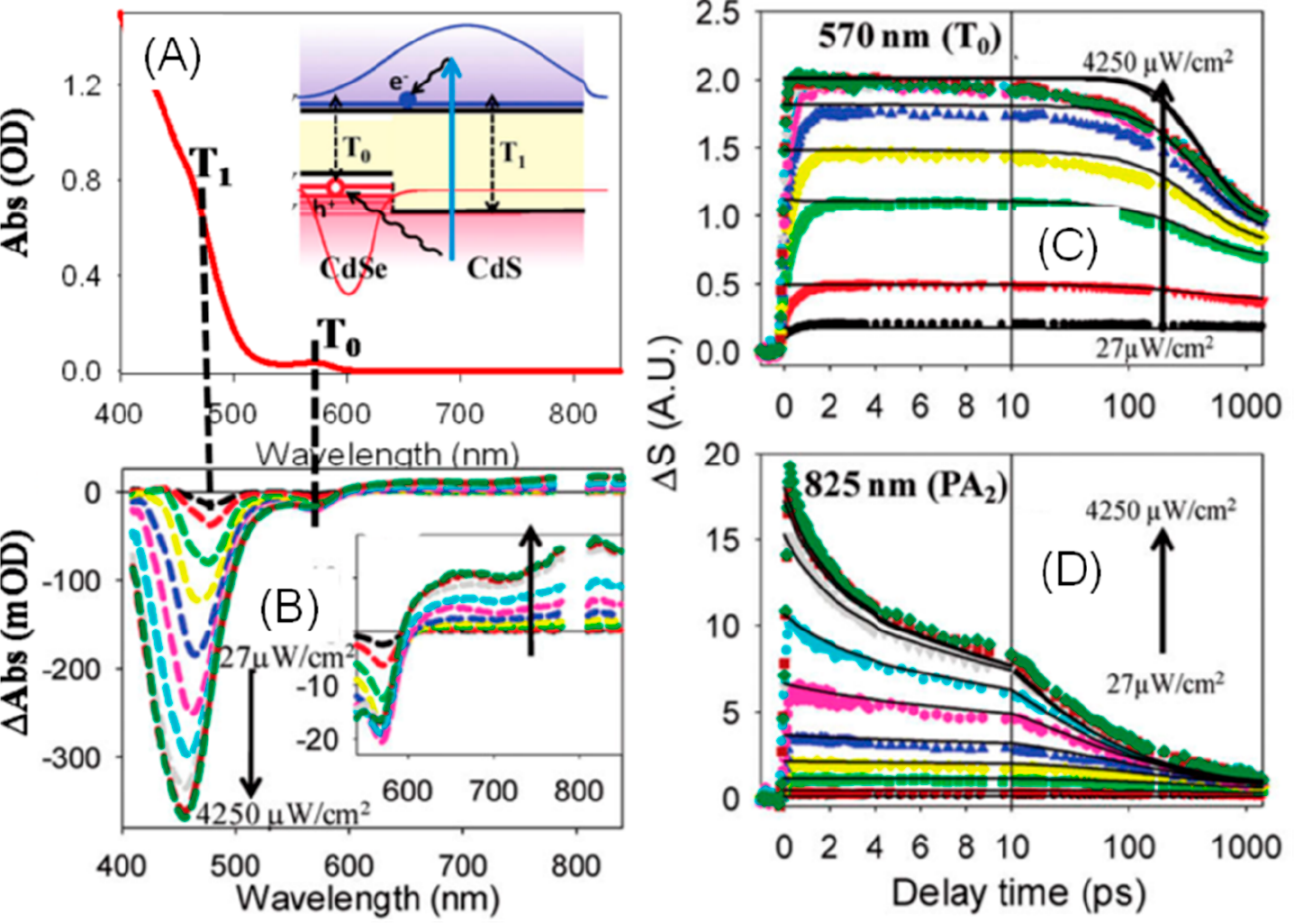
MEA in quasi-type II CdSe/CdS core/shell QDs. (A) UV–vis absorption spectrum. The inset shows a schematic energy level diagram, lowest energy electron and hole wave functions, and T0 and T1 optical transitions. (B) TA spectra at 0.3 ps after 400 nm excitation at different intensities. The inset shows an expanded view of the TA spectra between 500 and 840 nm. (C and D) Normalized TA kinetics (symbols) at (C) T0 and (D) PA bands and fits (solid curves) to the independent carrier Auger recombination model. Adapted with permission from ref (133). Copyright 2012 American Chemical Society.

The MEA rate can also be controlled by electron–hole wave function engineering such as constructing core–shell or interfacial alloy because the Auger recombination rate depends on electron–hole overlap.^133^ As shown in Figure 22A, CdSe/CdS core/shell QDs with a 1.2 nm core radius and 2.2 nm shell thickness have a quasi-type II band alignment and thus the electron wave function spreads over both core and shell, while the hole wave function is confined in the core. The peak at 575 nm and shoulder at 475 nm in the linear absorption spectrum correspond to the lowest-energy CdSe core-based transition (T0) and CdS shell-based transition (T1), respectively. The TA spectra of CdSe/CdS QDs (Figure 22B) show three spectral signatures: two negative peaks at the T0 and T1 positions and a broad positive signature at a wavelength longer than 650 nm. The two negative peaks have been attributed to the bleach of T0 and T1 absorption due to the state-filling effect. The broad positive signal has been assigned to photoinduced absorption (PA), more specifically, hole-induced absorption, and this assignment is supported by selective charge removal experiments. As pump intensity increases, the T0 bleach band gradually increases and saturates when exciton per dot exceeds two because of the twofold degeneracy of the 1S electron state. The T1 bleach shows blue shifting of the peak position and broadening of bandwidth due to the dynamic Burstein–Moss shift, indicating the quasi-continuum nature of the T1 transition. Like the near-IR PA signal for CdSe QDs, the PA signal of these core–shell QDs is also proportional to exciton numbers in QDs, providing a convenient probe to follow the MEA process.
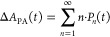
5.5The T0 and PA kinetics are simultaneously fitted by eqs 5.3 and 5.5, respectively (Figure 22C and D), giving a biexciton lifetime of ∼440 ps that is much longer than that for CdSe QDs with similar confinement energy.

Moreover, it has been shown theoretically that the abrupt confinement potentials of NCs break the strict momentum conservation and enhance the Auger recombination rate, while smooth confinement potentials of NCs suppress Auger recombination.^342−346^ As a result, alloyed interface in core/shell QDs are reported to suppress the QD blinking.^347−349^ Klimov and co-workers used giant core/shell QDs with multiple shells to provide a smoothly changed confinement potential for the QDs, which extended the biexciton Auger lifetime to ∼1.3 ns, and achieved the optical gain by direct current electrical pumping.^350^ Compared to the size-tuning method, which is limited by the NC size within a quantum-confined size region, interfacial alloying is a more efficient way to suppress the Auger recombination.^351^

### 5.3 Observations of Multiple Exciton Generation

Time resolved spectroscopies have been exploited to study the MEG in QDs because of the distinct lifetimes of single and multiple excitons. Under extremely low pump intensity, the probability of QDs with multiple excitons created by multiple photon absorption, according to the Poisson statistics, is negligibly small, and under such condition, MEG, if it occurs, will be the primary source for producing multiple excitons that lead to a fast decay component of exciton dynamics. Thus, the fast MEA can be employed as indicator of MEG under low-intensity excitation. In 2004, Schaller and Klimov noticed that when *hν*_pump_ > 3*E*_g_, a fast component in the first exciton bleach kinetics of the PbSe QD solution persisted even when the pump intensity was attenuated to a condition with average number of excitons (*w*) of less than 0.25.^331^ This fast component was not observed when *hν*_*pump*_ was reduced to less than 2*E*_g_. The time constant of this fast decay for the high-energy and low-intensity pump was then shown to be consistent with that of the biexciton Auger recombination and thus this fast decay component was ascribed to the short lifetime of multiple excitons generated by MEG. In 2005, Nozik and co-workers reported MEG in both PbS and PbSe QDs.^352^ They found that under similar pump intensity (*w* ∼ 0.25) the amplitude corresponding to the fast component increased with increasing *hν*_pump_ after exceeding a threshold (∼3*E*_g_). Additional ultrafast spectroscopic studies report the observation of MEG occurring in PbS,^353^ PbSe,^354^ PbTe,^355^ InAs,^332,356^ Si,^357^ InP,^358^ CdSe,^359,360^ CdTe,^361^ CdSe/CdTe core/shell QDs,^362^, PbSe/PbS core/shell QDs,^363^ CuInSe_2_ nanocrystals,^364^ and SWCNTs.^365^

However, during the same time controversy also arose about the reported occurrence or efficiency of MEG in some QDs. Nair and Bawendi reported that the quantitative analysis of biexciton and exciton signatures in time-resolved photoluminescence (TRPL) kinetics of CdSe and CdTe QDs shows no evidence of MEG when *hν*_pump_ > 3*E*_g_.^366^ Furthermore, a report from Pijpers and co-workers regarding MEG in InAs NCs was withdrawn by the authors because they could not reproduce the results and conclusion.^356^ Ruhman and co-workers reported negligible MEG in InAs/CdSe/ZnSe core–shell QDs at *hν*_*pump*_ > 3.8*E*_g_.^367^ Researchers also questioned that if the multiplication process in QDs was enhanced compared to bulk semiconductors. Bonn and co-workers indicated that for given *hν*_*pump*_, the carrier multiplication process in PbS and PbSe QDs may occur less efficiently than their bulk counterparts, and the enhancement of the multiplication arising from quantum confinement may be offset by the reduced DOS in QDs.^368^ Contrary to this argument, Beard and co-workers found that the efficiency of this multiplication process was enhanced by at least two times in PbSe QDs compared to their bulk counterpart, and they also argued that the MEG yield should be plotted as a function of *hν*_pump_/*E*_g_ because *E*_g_ is the fundamental unit of energy required to produce additional electron–hole pair in a given QD.^369^

**Figure 23:**
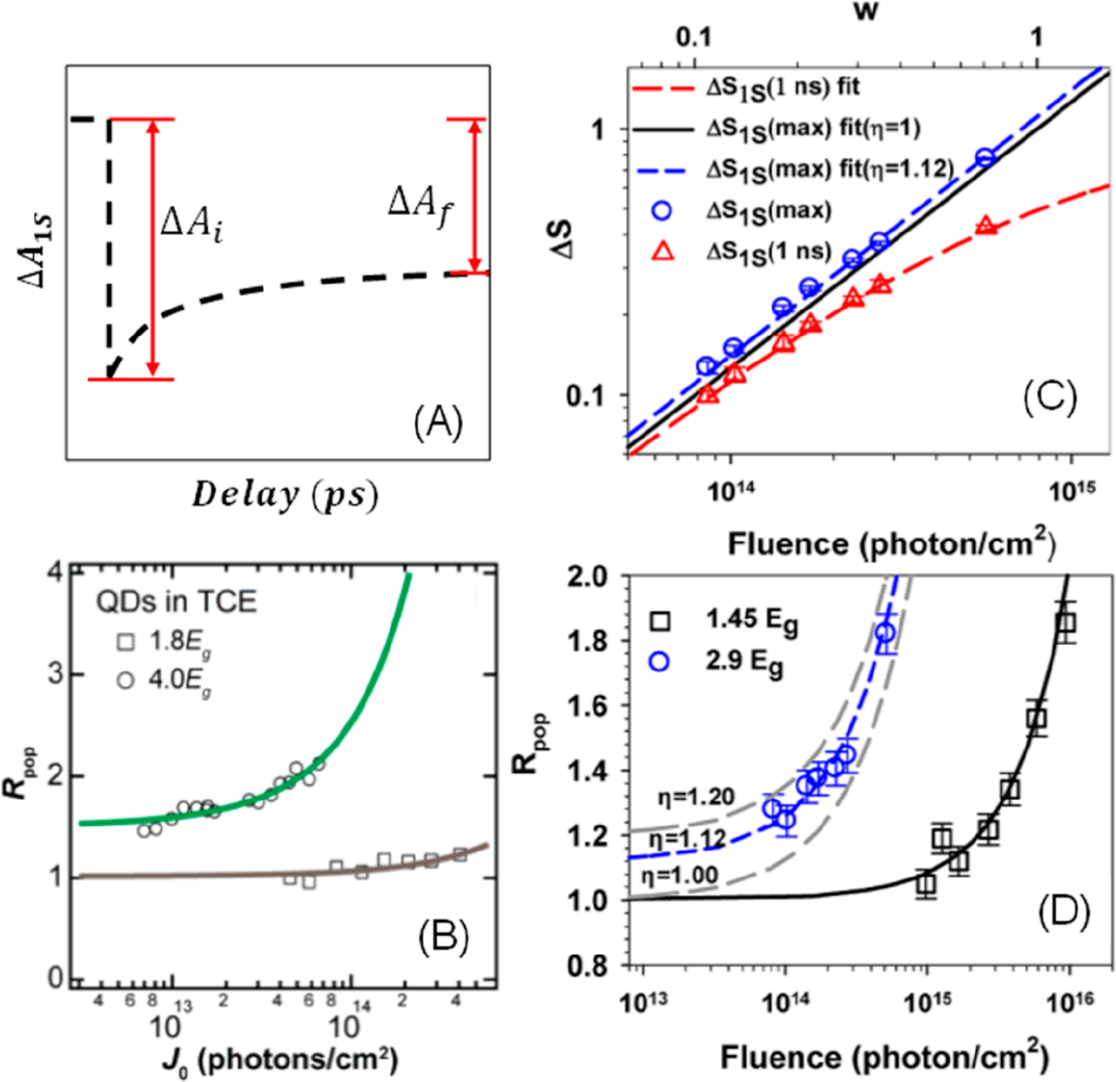
(A) Schematic illustration of 1S exciton TA kinetics (black dash curve). The amplitudes at short (Δ*A*_i_) and long (Δ*A*_f_) delays are proportional to the initially generated exciton and initially excited QDs, respectively. (B) MEG yield extraction for a PbSe QD solution. The symbols are the ratio of Δ*A*_i_/Δ*A*_f_ for different pump intensities. The curves represent eq 5.9 in the main text. (C) The plot of normalized TA signals (Δ*S*) of Δ*A*_i_ and Δ*A*_f_ (symbols) as a function of pump photon fluence. The scaling factor is defined in the main text. The lines and curve represent eqs 5.6 and 5.8, respectively, in the main text. (D) The ratio of Δ*S* at short and long delays at different pump photon fluences. The dashed curves represent the ratio of eq 5.6 to eq 5.8 in the main text. Adapted with permission from ref (136). Copyright 2012 American Chemical Society.

Beside the aforementioned controversy, the large inconsistency of the reported MEG yields led to the re-examination of the MEG determination methods.^370,371^ Several artifacts from the experimental design or data analysis have been uncovered to be responsible for the overestimation of the MEG yields reported in some of the earlier literatures. In principle, the number of excitons is proportional to TA amplitude under low pump intensity, and the MEG yield (η) can be extracted from the kinetics as the ratio between the initial and long delay amplitude (Figure 23A), Δ*A*_i_/Δ*A*_f_, where Δ*A*_i_ and Δ*A*_f_ represent the number of initially generated excitons and initially excited QDs, respectively. However, photocharging or photodegradation of QDs under high photon energy illumination can also cause similar fast exciton kinetics decay due to trion recombination or trapping, which leads to an overestimation of the MEG yield.^136,372,373^ This artifact can be avoided by vigorously stirring or flowing the samples during the measurement.^373^ Furthermore, another source of overestimation might be subtle to notice, yet its influence can be significant. Because the QD absorption coefficient increases drastically with the photon energy, the assumption of a homogeneous optical excitation along the light path in a QD solution is not valid. For this reason, multiphoton absorption is not negligible under high *hν*_pump_ even when *w* (average exciton number per dot) is very small, giving rise to overestimation of the MEG yield.^371,374^ This overestimation can also be reduced or eliminated by taking proper data analysis or experimental methods.

### 5.4 Quantification of Multiple Exciton Generation Yield

To avoid the overestimation stemming from multiple photon absorption, several methods have been developed to more accurately determine the MEG yield from TA measurements. In the first method, the samples were measured with different pump intensities. As the intensity decreases, the exciton kinetics approach to the same decay, indicative of the negligible contribution of multiphoton absorption events.^371^ Under such circumstances, η (MEG yield) can be determined from Δ*A*_i_/Δ*A*_f_ (Figure 23A).

Following this concept, the MEG yield at infinitely low pump intensity can be extrapolated from fitting of intensity-dependent TA signals. For lead chalcogenide QDs, the 1S exciton state is eightfold degenerate (including the spin degeneracy).^375^ According to the Poisson statistics, Δ*A*_i_ can be expressed as
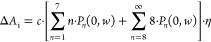
5.6where *P*_*n*_(0, *w*) represents the Poisson probability of QDs absorbing *n* photons when the average exciton number per dot is *w* and *c* is the effective absorption cross section of the sample at the probe wavelength. When the pump intensity is low, the first term in the square bracket of eq 5.6 is dominant and thus Δ*A*_i_ is proportional to the pump intensity (Figure 23B). Thus, eq 5.6 can be simplified as
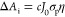
5.7where *J*_0_ is the pump photon flux, and *σ*_p_ is the effective absorption cross section of the samples at the pump wavelength. Δ*A*_f_ is recorded from the TA kinetics when MEA is completed, and its amplitude is proportional to the probability of optically excited QDs (including those with both single and multiphoton absorption events). Δ*A*_f_ is expressed as
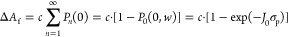
5.8Note that Δ*A*_i_ and Δ*A*_f_ are recorded from the normalized kinetics as shown in Figure 23A.^136^ The ratio of Δ*A*_i_/Δ*A*_f_ is now expressed as^357^
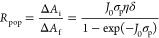
5.9where δ accounts for the single-exciton loss during the MEA process. For well-passivated QDs, δ is approximated to 1 because the single-exciton recombination is orders of magnitude slower than multiple-exciton recombination. As shown in Figure 23C, the measured *R*_pop_ is plotted as a function of the pump photon flux and eq 5.9 is applied as a fitting function with *σ*_p_ and η as fitting parameters. To improve the accuracy of the fit, a set of control *R*_pop_ values of the same sample were collected well below the MEG threshold so that η is known as 1. Thus, the η given by the fitting is equivalent to extrapolating η at infinitely small pump intensity so that the influence of multiphoton absorption can be minimized. Given the large degeneracy of 1S state, Δ*A*_*i*_ fell in the low pump intensity region (linear region) in most MEG reports. Thus, the MEG yield has been determined accurately by using either eq 5.6 (Figure 23D) or eq 5.7 (Figure 23C).

Another approach was developed by Ruhman and co-workers.^374^ Briefly, the concentration of the QD samples as well as the pump intensities was carefully controlled so as to obtain an identical multiphoton absorption contribution between large *hν*_pump_ (above MEG threshold) and small *hν*_pump_ (control experiment) pumping conditions. Thus, any difference in the number of generated excitons can be attributed to the MEG process. The accuracy of this measurement depends critically on the ability to measure the relative pump intensity, which can be difficult because of the variations of beam profiles and pump/probe overlap.

### 5.5 Strategies to Enhance Multiple Exciton Generation

**Figure 24:**
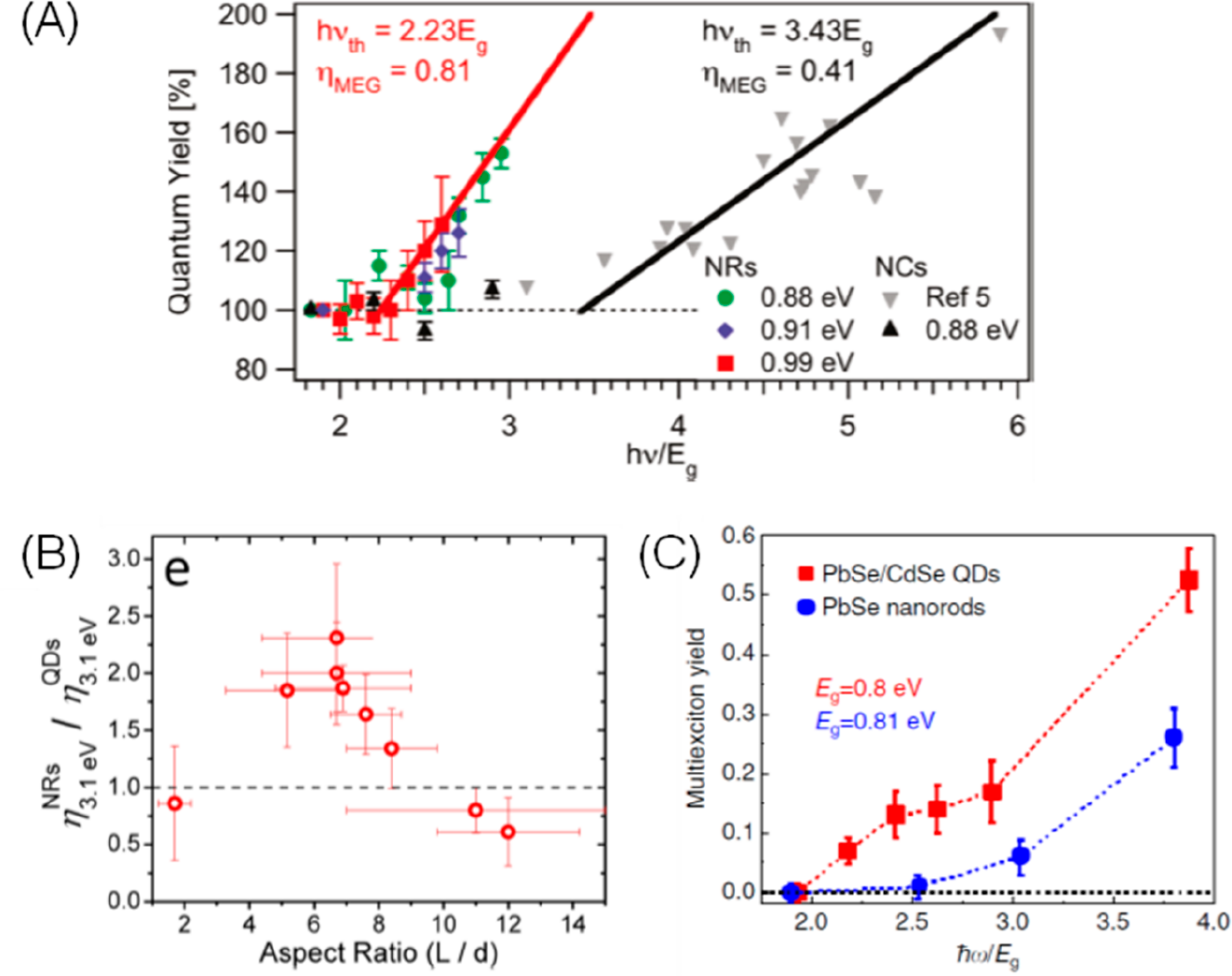
MEG enhancement. (A) Comparison of MEG in nanorods and nanocrystals. The MEG yield is plotted against *hν*_pump_/*E*_g_ for NRs and QDs with different *E*_g_. (B) The MEG enhancement factor for NRs as a function of the NR aspect ratio. (C) Comparison of the MEG yield in PbSe/CdSe QDs and PbSe NRs at various values of *hν*_pump_/*E*_g_. (A) Reprinted with permission from ref (376). Copyright 2011 American Chemical Society. (B) Reprinted with permission from ref (327). Copyright 2013 American Chemical Society. (C) Reprinted with permission from ref (377). Copyright 2014 Springer Nature.

Cunningham and co-workers have reported that, compared with 0D QDs with similar *E*_g_, the MEG yield in PbSe 1D NR is enhanced by a factor of 2 at *hν*_pump_ ∼3*E*_g_, and the MEG threshold (determined as 2.23*E*_g_) is also significantly reduced (Figure 24A).^376^ This enhancement has been attributed to the increased Coulombic interaction in NRs. Later, Klimov and co-workers further explored this MEG enhancement strategy by examining the influence of aspect ratio of the NRs.^327^ To quantitatively assess the MEG enhancement, a MEG enhancement factor is defined as the MEG yield in NRs divided by that in QDs with the same *E*_g_, which exhibits a maximum at an aspect ratio (length/diameter) of 6–7 (Figure 24B). For NRs with larger (>10) or smaller (<4) aspect ratios, MEG is not enhanced compared to QDs. In addition to shape controlling of nanocrystals, Klimov and co-workers recently have demonstrated that thick-shell PbSe/CdSe nanostructures can enhance the MEG yield by a factor of 4 over conventional PbSe QDs, and the enhancement is also accompanied by a considerable reduction of the MEG threshold (Figure 24C).^377^ The nanostructure and the band alignment in these core–shell QDs enable the high-energy holes to reside in the CdSe shell for longer time, evidenced by the cross-band emission at the visible region, and thus increases the probability of MEG by retarding the competitive hot carrier cooling process. Recently, Mohammed and co-workers reported the colloidal Ag_2_S QDs (band gap of 1.23 eV) as a new class of MEG materials due to their high photostability, low toxicity with a MEG threshold of 2.28*E*_g_, and efficiency of 173% at 3.2*E*_g_ excitation.^320^ Furthermore, Parkinson and co-workers reported the synthesis of the single-layered Ag_2_S nanoplatelets, which are expected to possess an enhanced Coulombic interaction due to the 2D morphology.^378^ Siebbeles and co-workers reported highly efficient MEG in 2D PbS nanosheets recently with MEG threshold about 3 eV (∼4*E*_g_), similar to PbS QDs but with higher MEG efficiency than QDs at the same *hv*_pump_/*E*_g_ ratio.^379^

Recently, the emerging perovskite nanocrystals have offered another platform for the study of MEG, and the highly efficient MEG yield in these weakly confined QDs has been attributed to the slow hot carrier relaxation.^380−386^ Highly efficient MEG with low threshold energy has been observed in formamidinium lead iodide and cesium lead iodide perovskite nanocrystals.^380,381^ Li and co-workers found that in the lead iodide perovskite nanocrystal the MEG efficiency could be tuned by partially substituting the lead ion by tin, and in this report the MEG threshold approached twice the bandgap.^385^

### 5.6 Multiple Exciton Generation in Electronically Coupled QD Solids

The aforementioned MEG measurements have been conducted on QDs dispersed in solution, which are electronically isolated from one another. Nonetheless, many QD photovoltaic applications, such as solar cells,^387−389^ photoelectodes,^23^ and photodetectors^390^ are based on the coupled and compact QD solids in which the charge carriers can diffuse from one dot to another. Thus, it is critical to understand and control the MEG in these electronically coupled QD solids for MEG-based devices.

**Figure 25:**
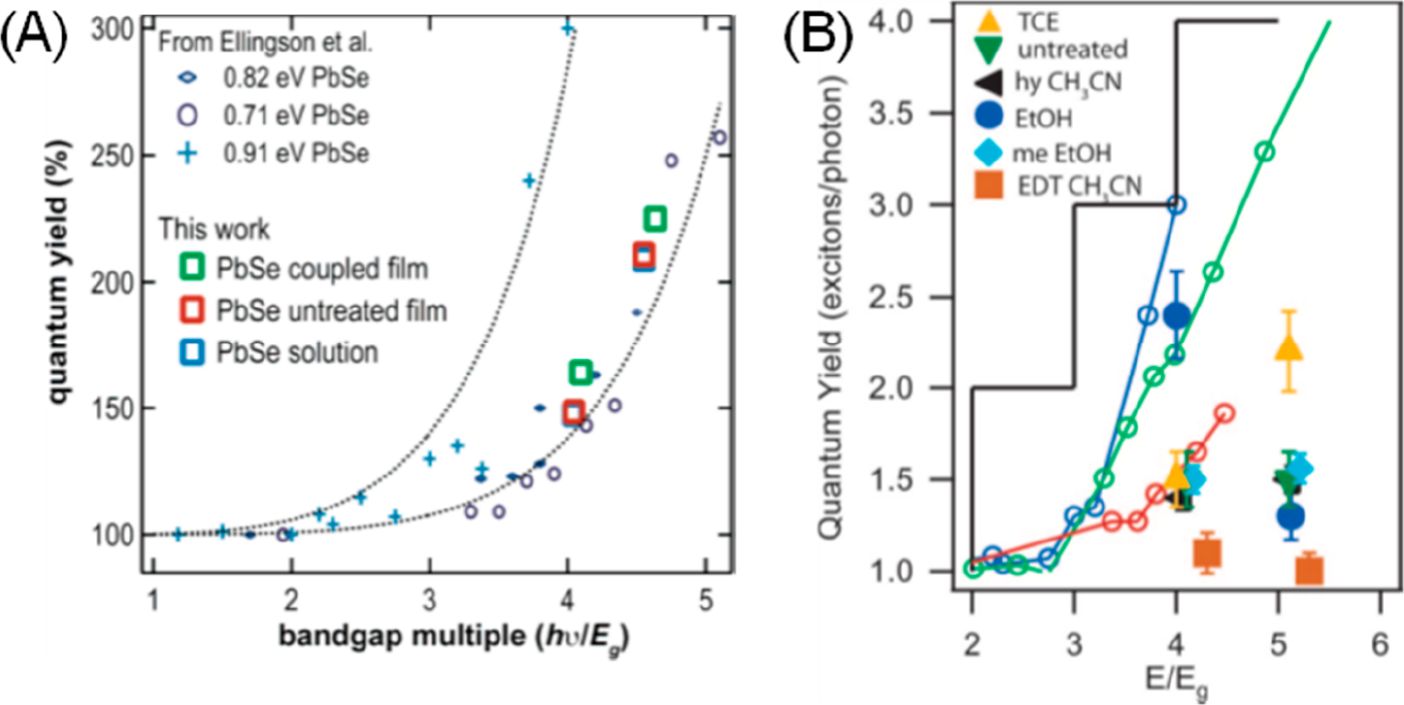
(A) The comparison of MEG yield for PbSe QD films and QD solutions. Note that the QYs for QDs in solution and untreated films are identical. (B) The comparison of MEG yield as for the indicated QD solids between QD solution and the ideal MEG yield (achieving *n* excitons at *n* times *E*_g_). (A) Reprinted with permission from ref (391). Copyright 2007 American Chemical Society. (B) Reprinted with permission from ref (392). Copyright 2009 American Chemical Society.

Compared with QD solutions, the TA studies of PbSe QD films have indicated the decreased single exciton lifetime and increased biexciton lifetime, and these changes are escalated when the interdot distance is shortened after hydrazine treatment due to delocalization of the exciton wave function.^391^ However, distinguishable changes of the MEG yield between the QD solution and the films was not observed (Figure 25A).^391^ Further experiments revealed a particularly interesting result in that the MEG yield showed strong dependence upon the post-chemical treatments (Figure 25B), and several interpretations of this result were proposed, such as ligand-modified hot carrier cooling, relaxation of quantum confinement due to the coupling, and/or introduction of dopants.^392^ These studies have inspired the development of MEG-enhanced solar cells based on chemically treated QD films.^22,23^ It should be noted that to accurately extracted the MEG yield in the films, the value of both σ and δ in eq 5.9 were experimentally determined, which were different from those in QD solution.

**Figure 26:**
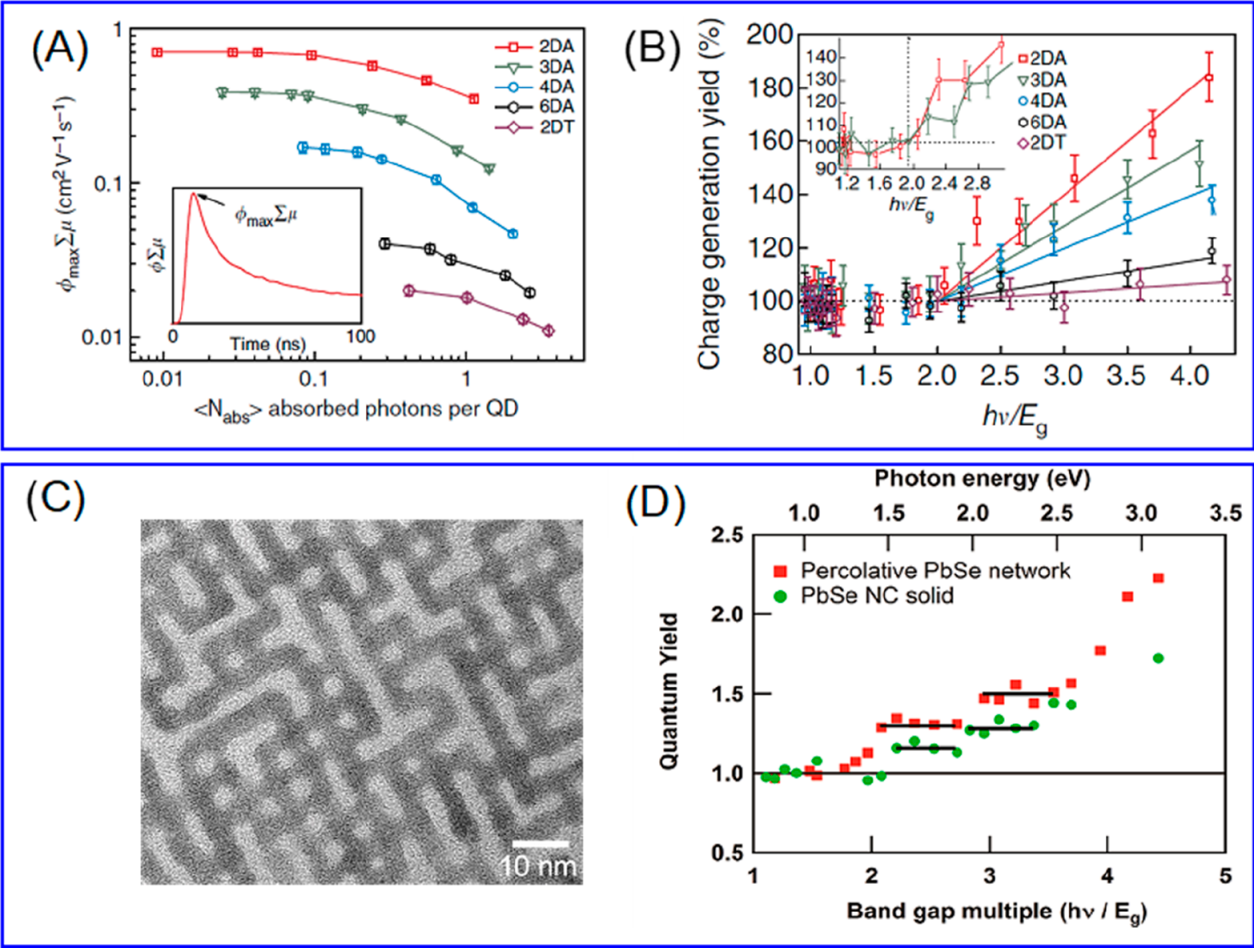
(A) Photoconductivity of the QD films as a function of the average number of absorbed photons per QD for QD films with various cross-linking molecules. The inset shows a typical photoconductance transient and the point that corresponds to the maximum value. (B) MEG yield as a function of *hν*_pump_/*E*_g_ for all films. The lines are linear fits to the data assuming an energy threshold of 2*E*_g_. (C) TEM image of a monolayer percolative PbSe network (scale bar represents 10 nm). (D) MEG yield as a function of *hν*_pump_/*E*_g_ (bottom axis) and as a function *hν*_pump_ (top axis) for the percolative PbSe network and the PbSe NC solid. (A and B) Reprinted with permission from ref (393). Copyright 2013 Springer Nature. (C and D) Reprinted with permission from ref (394). Copyright 2018 American Chemical Society

For QD film photovoltaic devices, higher carrier mobility is always desirable to achieve a longer diffusion length and less carrier loss. The mobility can be controlled by tuning the interdot spacing of the films. Siebbeles and co-workers have demonstrated that the MEG yield varies with the carrier mobility in PbSe QD films.^393^ In this experiment, the carrier mobility in QD films was systematically tuned over more than two orders of magnitude by changing the cross-linking molecules (Figure 26A). As shown in Figure 26B, the determined MEG yield of the films progressively decreases as the carrier mobility increases. The film with the smallest mobility, treated with 1,2-ethanedithiol (purple diamonds), shows the least MEG yield, whereas the film with the largest mobility, treated with 1,2-ethanediamine (red squares), shows the highest MEG yield. This experiment further reveals that the threshold energy of the MEG in these films is 2*E*_g_, much lower than the reported values for dispersed QDs. More recently, Siebbeles and co-workers showed that, compared with a QD solid, MEG is more efficient in a percolative network of directly connected PbSe QDs (Figure 26C).^394^ It was found in this study that the MEG yield in this percolative QD network increased in a step-like fashion with *hν*_pump_ and was higher than that for the QD solids with weaker interdot coupling (Figure 26D), implying the dependence of MEG on coupling strength.

### 5.7 Multiple Exciton Dissociation via Charge Transfer

**Figure 27:**
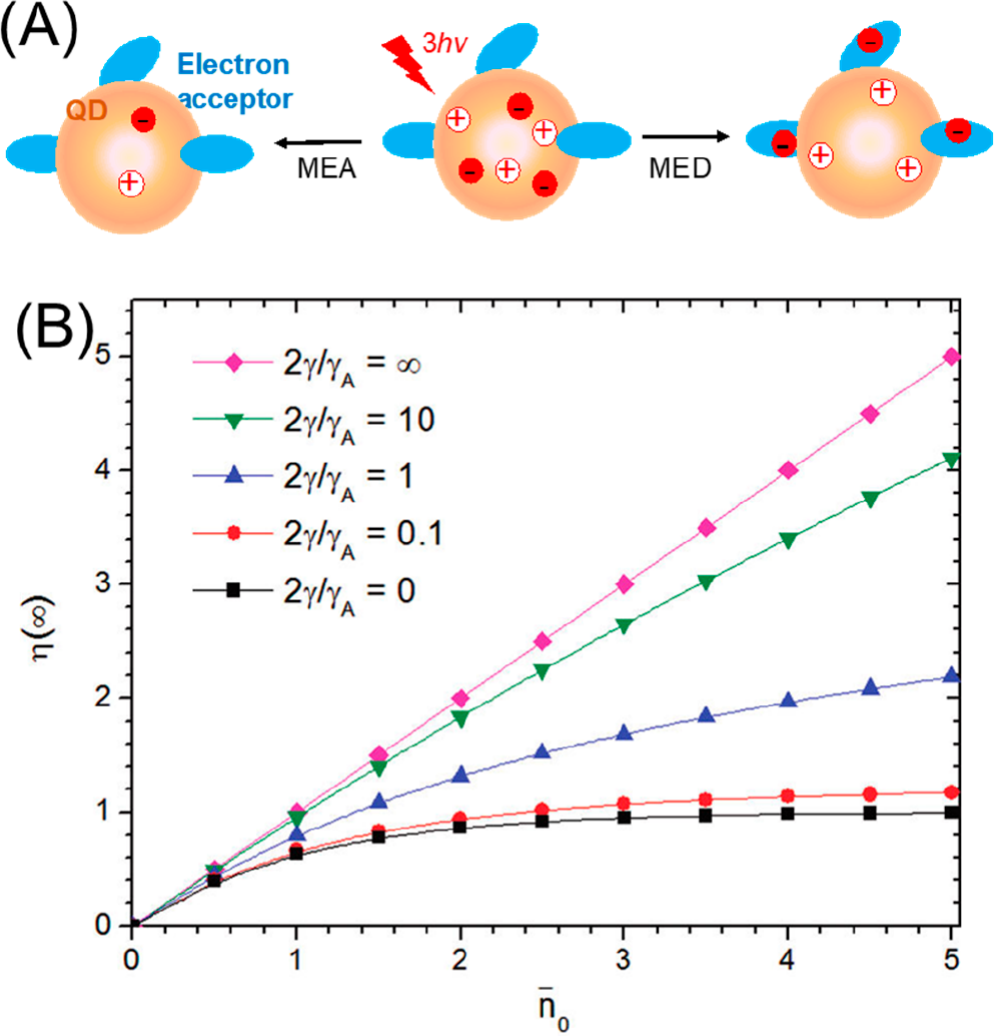
(A) Schematic illustration of MED via electron transfer to adsorbed molecular electron acceptors. (B) The calculated ultimate yield of dissociated excitons per dot as a function of the average initial exciton number per dot calculated for various values of the ratio of ET rate and biexciton decay rate (γ/*γ*_A_). The calculation is based on the Poissonian initial exciton distribution model described in the main text. (B) Reprinted with permission from ref (395). Copyright 2009 American Chemical Society

QDs can generate and accumulate multiple excitons through MEG resulting from the absorbance of either one high-energy photon or multiple photons. For QD-based solar cells or photocatalysts, efficient photon-to-charge conversion requires a complete exciton dissociation. Owing to the strong quantum confinement, the electron and hole wave functions extends beyond the QD surfaces, which facilitates the exciton dissociation either via interdot charge transfer in coupled QD films or via interfacial charge transfer in QD–molecule complexes. Unfortunately, the spatial confinement of QDs also enhances the exciton Auger recombination process, leading to ultrafast multiple exciton decay via MEA. Thus, the quantum yield of multiple exciton dissociation (MED) depends on the competition between charge transfer and MEA. As illustrated in Figure 27A, the electron transfer (ET) from QDs to molecular adsorbates is exploited to dissociate the multiple excitons in QDs. The ET process occurs on time scales of sub-ps to tens of ps, and the dependence of ET rates (γ) on various factors have been discussed in section 4. In parallel to the ET process, the multiple excitons can also decay via MEA. The biexciton lifetime in QDs is on the time scale of tens of ps, and its decay rate constant is denoted as *γ*_A_. As discussed previously, the MEA rate increases quickly with the number of excitons per dot, *k*_*n*_ = 1/2*γ*_*A*_*n*^2^(*n* – 1). Thus, as the exciton number increases, the MEA rate will eventually exceed the ET rate, giving rise to a maximum number of dissociated exciton (*n*_max_). Assuming the average adsorbates per dot is much larger than the average exciton number per dot, Hilczer and Tachiya derived the analytical expression describing the MED yield based on the Poissonian exciton distribution model.^395^ According to this analysis, *n*_max_ is governed by the ratio of γ/*γ*_A_ and bounded between two limits (Figure 27B) under various pump intensities: 1 when γ/*γ*_A_ ∼ 0, corresponding to solely single exciton dissociation, and *n* when γ/*γ*_A_ ∼ ∞, corresponding to complete MED. To experimentally determine *n*_max_, the molecular adsorbate TA signals are normally used as indicators. When the ET rate from QDs to the adsorbate is much faster than single exciton recombination, a complete exciton dissociation can be assumed under very low pump intensity when the single excitation event dominates. Then, the measured adsorbate TA signals is linearly scaled to match the average exciton number per dot determined from QD TA signal, and the same scaling factor is applied to the adsorbate TA signals for all different pump intensities. Thus, these scaled TA signals (Δ*AM*) represent the number of dissociated excitons, which can also be predicted from the Poisonian exciton distribution model:
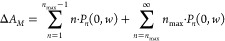
5.10where *P*_*n*_(0, *w*) is the Poissonian exciton distribution term that is defined in eq 5.1.

**Figure 28:**
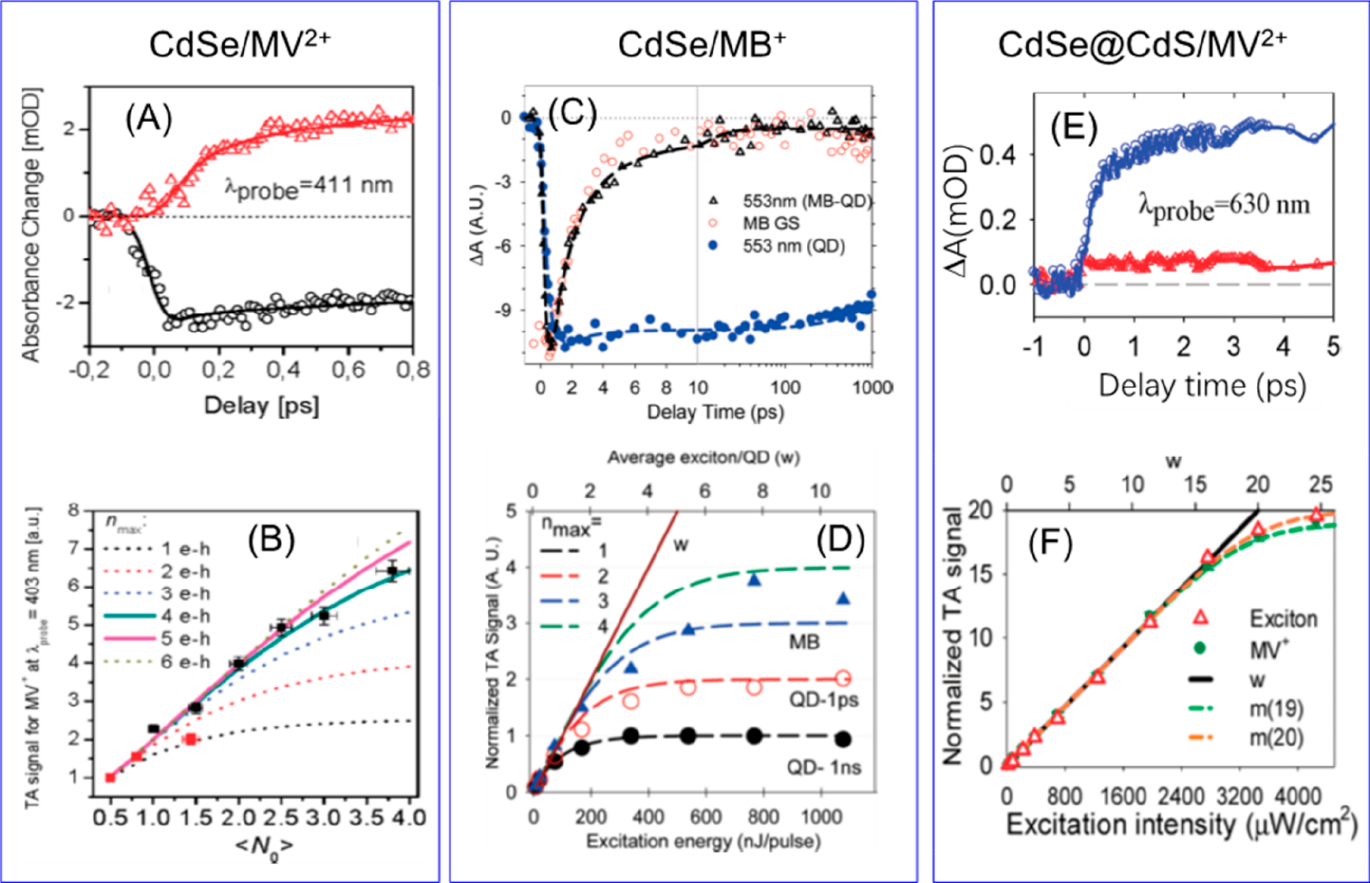
(A) TA kinetics probed at the absorption band of the MV^•+^ radical (411 nm) with (red triangle) and without (black circles) adsorbed MV^2+^. The time constant of ET from QD to MV^2+^ is extracted as 70 fs by fitting the kinetics traces (solid curves). (B) Comparison of the experimental (symbol) and predicted (curves) MV^•+^ TA signal as a function of the average initial exciton number per dot. (C) The formation of ground-state bleach (GSB) of MB^+^ is consistent with the 1S exciton bleach decay, suggesting the ET from QD to MB^+^. The comparison of TA kinetics of 1S exciton between QDs and the QD-MB^+^ complex also suggests this fast ET process. The kinetics fitting gives the ET time constant as 2.2 ps. (D) Comparison of the experimental (blue triangle) and predicted (curves) MB^+^ GSB and 1S exciton bleach signal as a function of pump intensity. (E) TA kinetics probed at the absorption band (630 nm) of the MV^•+^ radical with (blue circles) and without (red triangle) adsorbed MV^2+^. (F) Comparison of the experimental (green solid circle) and predicted (dash curves) MV^•+^ TA signal and 1S exciton bleach signal (red triangle) as a function of the pump intensity. (A and B) Reprinted with permission from ref (396). Copyright 2009 American Chemical Society. (C and D) Reprinted with permission from ref (325). Copyright 2010 American Chemical Society. (E and F) Reprinted with permission from ref (133). Copyright 2012 American Chemical Society.

It has been experimentally demonstrated that the multiple excitons generated by multiple photon absorption can be efficiently dissociated via ET to adsorbates in competition with MEA in nanocrystals. The complexes of CdSe QDs and efficient electron acceptors, methyl viologen (MV^2+^) and methylene blue (MB^+^), have been used as prototypes to study MED from QDs.^133,151,325,396^ As shown in Figure 28A, C, and E, the ET from QDs with a single exciton to MV^2+^ and MB^+^ are on the time scales of sub-ps and several ps, respectively, both of which are much faster than the single exciton lifetime. The ET-induced adsorbed TA signals, absorption of methyl viologen radical (MV^•+^), or the bleach of methylene blue (MB^+^) in TA spectra have been used to measure *n*_max_. As shown in Figure 28B, D, and F, the scaled MV^•+^ absorption and MB^+^ bleach signals (symbols), representing the number of dissociated excitons, are compared with the predicted values given by eq 5.10 (curves) with different hypothetical *n*_max_ values. These comparisons imply that the experimental *n*_max_ values in CdSe-MV^2+^, CdSe-MB^+^, and CdSe@CdS-MV^2+^ are around 5, 3, and 19, respectively. Compared with MB^+^, the faster ET rate to MV^2+^ results in the larger *n*_max_. Compared with CdSe QDs, the spatial distribution of electron and hole wave functions in the quasi-type II CdSe@CdS QDs retains the ultrafast ET rate while greatly reducing the MEA rate (biexciton lifetime 440 ps). In other words, this core/shell structure enables a large γ/*γ*_A_ ratio, which is believed to the reason for the large *n*_max_. In Figure 28F, the consistence between the scaled exciton and MV^•+^ signals suggests a unity MED yield at the corresponding pump intensity. In addition to wave function engineering, Lian and co-workers have also demonstrated another approach to enhance MED yield exploiting the scaling law for MEA.^151^ Compared to the spherical QDs, the exciton binding energy in a 1D quantum rod (QR) is significantly enhanced, arising from the dielectric confinement of the surrounding medium. As a result, the MEA in QRs is expected to proceed via exciton–exciton collision following the two-particle Auger recombination model. According to the scaling law, the MEA rate increases cubically with the exciton number in QDs because of three-particle Auger recombination, while it increases quadratically with exciton numbers in QRs because of two-particle Auger recombination. Thus, the MEA, especially for high-order multiple exciton states, is slowed in QRs. Consequently, the experimentally determined *n*_max_ in CdSe QR-MV^2+^ complexes is increased up to 21.

More recently, multiple exciton dissociation in CdS-Pt NRs has also been reported.^397^ In CdS NRs, rapid hole trapping leads to a long-lived biexciton lifetime (1/*k*_2_^A^) of 2.0 ± 0.2 ns and multiexciton Auger recombination processes that follow a Carrier-Collision model, *k*_*n*_^A^ = *n*^2^(*n* – 1)/4*k*_2_^A^. In CdS-Pt nanorods, because of ultrafast electron transfer from CdS to Pt (with a half-life time of 5.6 ± 0.6 ps), multiple electrons can be transferred to the Pt from the multiple exciton states. The half-life of charge-separated state decreases from 10 μs in single charge-separated state to 42 ns in nine charge-separated states. This result suggests metal-tipped NCs can be a promising platform for exploiting the unique multiple exciton properties of NCs to drive multiple electron chemistry.

### 5.8 MEG Enhanced Multiple Carrier Extraction

**Figure 29:**
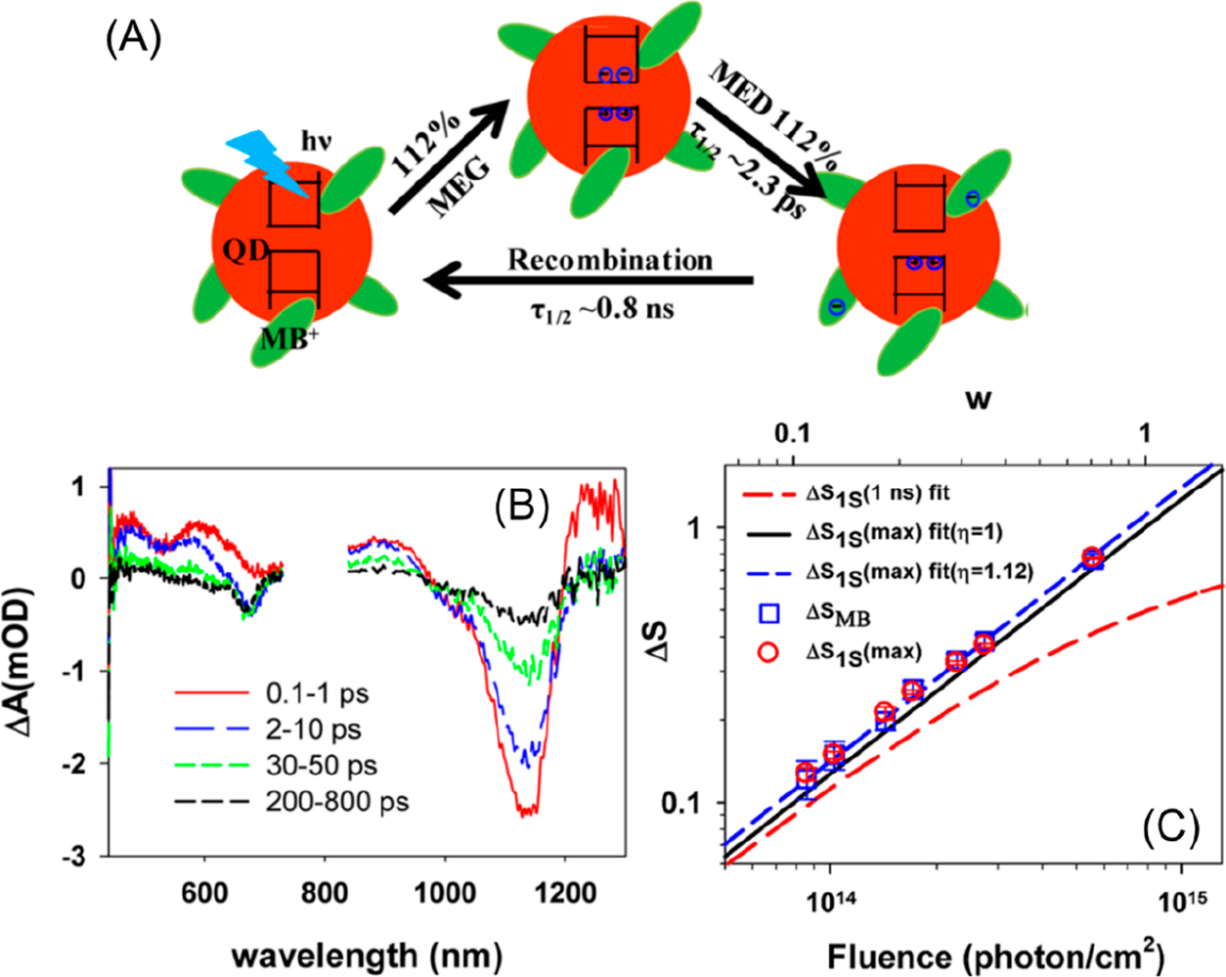
(A) Schematic illustration of MEG and the following MED in the PbS/MB^+^ complex. (B) The TA spectra at indicated delays. The fast recovery of 1S exciton bleach and formation of MB^+^ GSB suggest the ET from PbS to MB^+^. (C) Comparison of the experimental (blue square) and predicted (dash curves) MB^+^ GSB TA signals and the 1S exciton bleach signal (red circle) as a function of pump intensity. Reprinted with permission from ref (136). Copyright 2012 American Chemical Society.

The method of using ET to dissociate excitons has also been extended to dissociate the multiple excitons created by MEG in lead sulfide (PbS) QDs (Figure 29A).^136^ The complex of PbS QD adsorbed by MB^+^ was used as a model to demonstrate this idea. The TA spectra of PbS-MB^+^ show two prominent features: the 1S exciton bleach at 1140 nm and the MB^+^ ground-state bleach (GSB) at 667 nm (Figure 29B). The former is due to the state filling of both the 1S electron and hole levels, and the latter indicates ET from the excited QDs to MB^+^ molecules. The analysis of kinetics of these two features reveals the time constant of ET from the QD single exciton state to MB^+^ to be around 2.3 ps. The MEG efficiency in free PbS QDs for *hν*_pump_ = 2.9*E*_g_ was quantified as 112% using the methods introduced previously. Then, PbS-MB^+^ complex solutions with the same concentration as free QDs were examined under the identical experimental condition to measure the MEG and the following MED in the QD–acceptor complexes. Using the similar MED analysis approach for CdSe QDs, the ET-induced MB^+^ GSB signal was scaled to determine the dissociated excitons. As shown in Figure 29C, the scaled MB^+^ GSB signals are consistent with the normalized 1S exciton bleach signals at different pump intensities, suggesting that all excitons are dissociated in PbS-MB^+^ complexes and the presence of efficient electron acceptors does not influence the MEG efficiency of PbS QDs. This study has demonstrated that ultrafast interfacial ET can be an efficient way for extracting multiple excitons generated by the MEG process.

**Figure 30:**
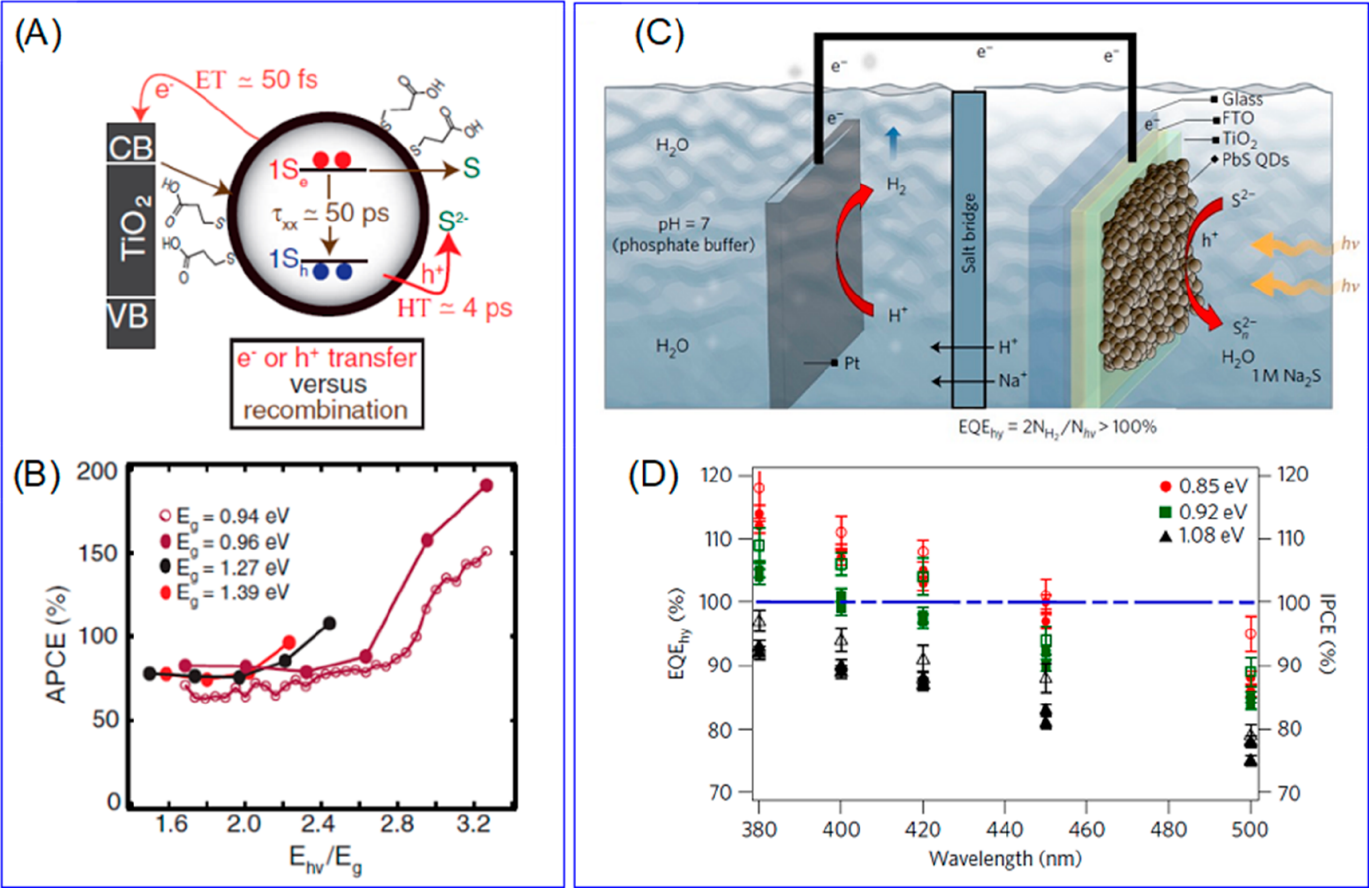
(A) Illustrative representation of a QD adsorbed on a TiO_2_ single crystal and the expected charge transfer process. 1S_e_ and 1S_h_ refer to the lowest electron and hole states, respectively. (B) APCE values of a QD-sensitized TiO_2_ electrode as a function of the illumination photon energy. (C) Schematic of the PEC apparatus. The QD layers serves as photon absorber of the photoanode. Photogenerated holes oxidize sulfide in one compartment while electrons reduce hydrogen in the other compartment at the dark Pt cathode. A salt bridge connects the two compartments and transports H^+^ and Na^+^. (D) EQE (filled symbols) for hydrogen generation induced by PbS QD photoelectrodes with various *E*_g_ in Na_2_S aqueous electrolyte. IPCE (open symbols) at the range of 380–500 nm is also illustrated for comparison. (A and B) Reprinted from ref (398) with permission from AAAS. (C and D) Reprinted with permission from ref (23). Copyright 2017 Springer Nature.

Besides the dispersed QD–adsorbate systems, MEG-enhanced photocurrent and hydrogen generation have also been demonstrated in PbS QD-integrated photoelectrochemical systems. Parkinson and co-workers observed the MEG effect in a photoelectrochemical cell where light was captured by a PbS QD monolayer chemically bound to a TiO_2_ single crystal electrode.^398^ The excitons generated in PbS were dissociated via electron injection into the TiO_2_ electrode, and the remaining holes were shuttled to the counter electrode by the electrolyte to form photocurrent (Figure 30A). Taking into account the loss from uncaptured photons, the absorbed photon-to-current efficiency (APCE) was reported to reach as high as 180% at large *hv*_pump_/*E*_g_ because of MEG (Figure 30B). The APCE measurements also indicated that the threshold of MEG in a PbS QD-sensitized TiO_2_ electrode was QD-size-dependent, falling in a range of 2.4–3*E*_g_. Recently, Beard and co-workers demonstrated that extra carriers produced via MEG in PbS QD-based photoelectrodes can be used for hydrogen generation with a quantum yield above unity.^23^ The electrochemical device was composed of a PbS QD photoanode and a Pt cathode that were placed into two compartments connected by a salt bridge (Figure 30C). The PbS QD multilayer film in the photoanode captures photons. The resultant electrons are extracted by the TiO_2_ substrate and flow to the Pt cathode for hydrogen evolution, while the holes in the QD film are filled by oxidizing the surrounding sulfide ions in the electrolyte. Since no external bias was applied, the driving force for the whole chemical reactions should be provided by the photovoltage arising from the illumination of the photoanode and the chemical potential difference in the two compartments. Thanks to the nearly unity Faraday efficiency, both the incident photon-to-current efficiency (IPCE) and the external quantum yield of the photon-to-hydrogen conversion exceed 100% due to MEG at *hv*_pump_/*E*_g_ > 2.7 (Figure 30D).

**Figure 31:**
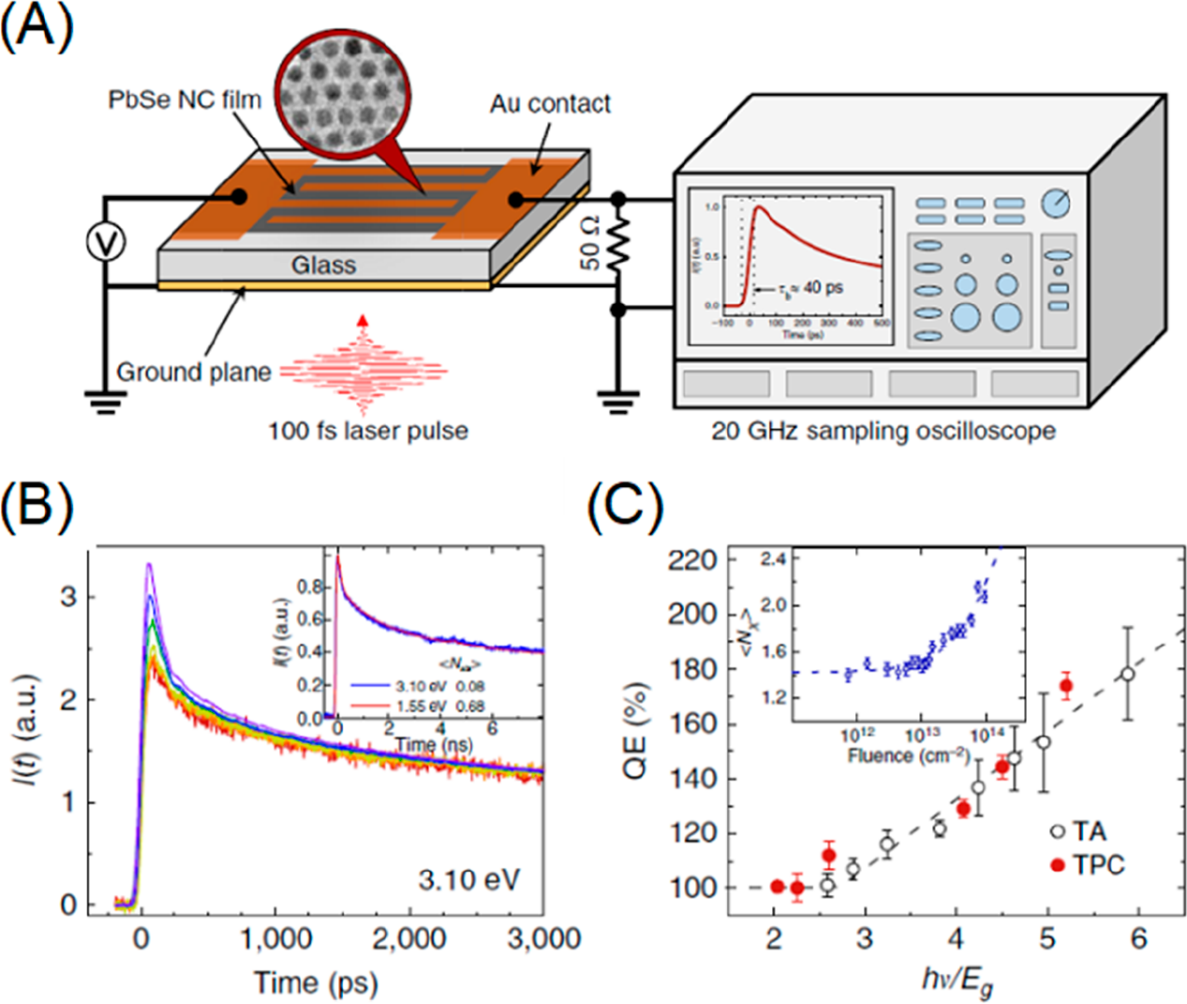
(A) Illustration of the transient photocurrent detection apparatus. A photoconductive switch comprises a thin film of PbSe QDs deposited on a glass substrate with top interdigitated Au contacts. Photocurrent is excited by short laser pulses and monitored with a 20 GHz sampling oscilloscope. (B) Tail-normalized transient photocurrent traces with excitation above the MEG threshold show the persistence of a fast Auger decay component in the limit of low pump fluences, indicating the presence of MEA. The inset shows that the low-fluence transient photocurrent kinetics measured with 3.1 eV photons can be reproduced using 1.5 eV excitation but with a much higher pump intensity. (C) Comparison of the MEG yields determined from the transient photocurrent measurements (red solid circles) of the QD films to those from TA measurements of the QD solutions. Reprinted with permission from ref (399). Copyright 2015 Springer Nature.

To take advantage of MEG in QD solar cells, the MEG-resultant excitons should be efficiently dissociated and extracted from QD films. Klimov and co-workers has demonstrated that MEG can also contribute to the photocurrent extracted from a PbSe QD film.^399^ As shown in Figure 31A, a PbSe QD film with strong interdot coupling was sandwiched between a gold ground plane and gold top contact. One contact of the switch is biased with an adjustable d.c. voltage, while the other is connected to the input port of a fast sampling oscilloscope with a 20 GHz bandwidth. The switch is triggered with 100 fs laser pulses. The overall system response time is 40 ps. In analogy to the MEA kinetics in TA measurements, the detected transient photocurrents have also exhibit the fast decay component from Auger recombination, which is also exploited to uncover the multiple exciton contribution by applying the Poissonian exciton distribution model (Figure 31B). The low-fluence transient photocurrent traces measured with *hν*_pump_ above the MEG threshold can be reproduced by pumping the sample below the MEG threshold with a higher pump intensity (inset of Figure 31B). The consistent kinetic traces imply the presence of multiple excitons for both pump conditions despite different origins: MEG in the former case and multiple photon absorption in the latter case. At *hν*_pump_ = 4.5*E*_g_, the MEG yield of the QD film could be extracted as 144% from the analysis of the pump-intensity-dependent transient photocurrents (inset of Figure 31C). The MEG yields have been determined for different *hv*_pump_/*E*_g_ ratios using this optoelectronic measurement, which is in agreement with those obtained from the spectroscopic measurements.

**Figure 32:**
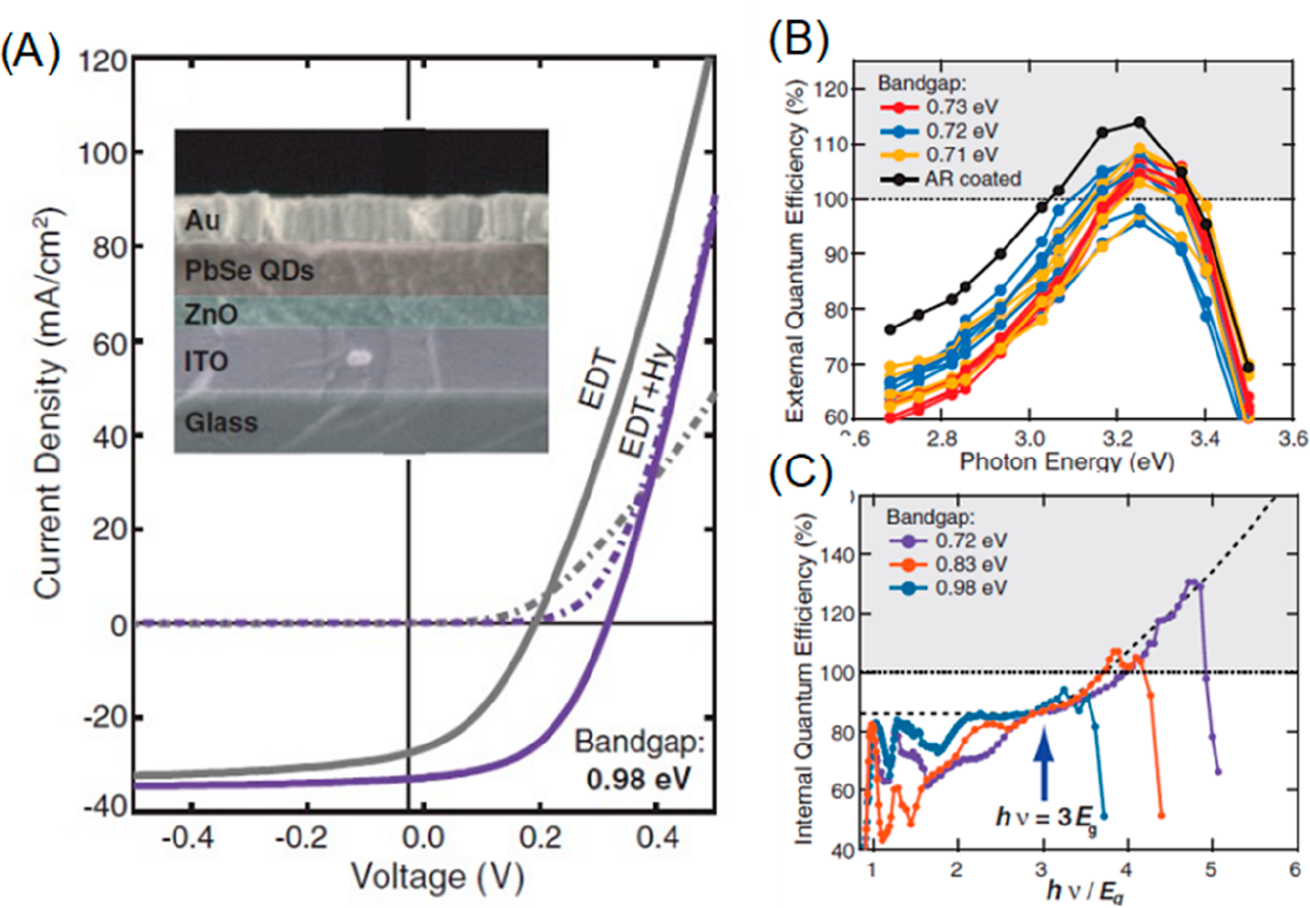
(A) Comparison of current–voltage characteristics under simulated AM1.5G illumination of devices assembled from EDT- and EDT+hydrazine-treated QD films. The inset shows structure of the PbSe QD solar cells. (B) EQE peaks for different independent solar cells made with QD *E*_g_ ∼ 0.7 eV. (C) Collected IQE curves vs *hv*/*E*_g_ for three solar cells with different values of *E*_g_. The dashed curve represents the predicted MEG yield based on eq 5.9 in main text. Reprinted from ref (22) with permission. Copyright 2011 AAAS.

The MEG-induced photocurrent enhancement in a QD solar cell was first demonstrated by Beard and co-workers, and the external quantum efficiency (EQE) maximum of 114% was reported for the best device.^22^ In this report, the solar cells were fabricated in a planar multilayer heterojunction structure (Figure 32A, inset). The PbSe QD films were deposited on the substrate in a layer-by-layer fashion with a 1,2-ethanedithiol (EDT) and hydrazine treatment. Compared with the EDT-only treatment, this cotreatment method leads to significant improvement of the solar cell performance (Figure 32A). The EQE spectra of most tested devices (15 out of 18) with QD *E*_g_ ∼ 0.7 eV show peak values exceeding 100% near 3.2 eV, and the champion device gives a peak EQE of 107% (Figure 32B). To further assess the MEG efficiency and threshold, the internal quantum efficiency spectrum, obtained by only considering the absorbed photons, is plotted as a function of the normalized photon energy (*hv*/*E*_g_). The internal quantum efficiency (IQE) curves for different devices exhibit nearly constant short-circuit collection yields (∼85%) until the photon energy surpasses the MEG threshold, after which the IQE increases to a peak efficiency (Figure 32C). Taking into account the 15% intrinsic carrier loss, these IQE curves are qualitatively consistent with the modeled MEG efficiency as a function of *hv*/*E*_g_ (eq 5.9), evidencing the MEG enhancement in these devices when *hv* is above the threshold. The IQE drops at higher photon energy (>3.3 eV) because of the absorption of ITO. The IQE spectrum also indicates that the MEG threshold for the device with a QD *E*_g_ of 0.72 eV is around ∼2.8*E*_g_.

## 6 Photocatalysis Using Nanocrystals

The efficiency of a photocatalytic reaction depends on the efficiencies of three important processes: light absorption, charge separation, and catalytic turnover.^60,206,400−402^ In this context, quantum-confined NCs are ideal light-harvesters to drive photocatalytic reactions. The quantum confinement effect leads to strong light–matter interaction that enhances the light absorption cross sections of these NCs. Artemyev and co-workers quantified the NC intrinsic absorption coefficient (absorption coefficient per unit NC volume) via inductively coupled plasma atomic emission spectroscopy and a continuum absorption Lorentz local field model.^13^ In particular, they showed that the intrinsic absorption coefficient of 2D NPLs is even further enhanced compared to that of 1D NRs and 0D QDs due to higher optical field penetration along the basal plane, where the local field factor along these directions approaches unity.^13^ Meanwhile, the charge separation properties of 0D, 1D, and 2D NCs can be precisely engineered via both the quantum confinement effect and wave function engineering approaches, as we review above. Compared to 0D QDs, 1D NRs and 2D NPLs provide an additional knob to tune the charge separation distances by tailoring the length or area of their unconfined dimensions. In addition, surface defects or atoms on NCs in some cases function as catalytic sites themselves,^403,404^ although the efficiency of photocatalytic reactions using these “intrinsic” catalytic sites tends to be low. There are several ways to design efficient photocatalytic systems using NCs, such as NC–molecular catalyst hybrids, NC–enzyme hybrids, NC–redox mediator–catalyst triads, and NC–metal heterojunctions. It is important to emphasize that most of these studies focus on the photoreduction half-reaction, with the photogenerated holes removed by the use of sacrificial electron donors (or hole acceptors). In this section, we review the structure, charge separation mechanism, and efficiency-limiting factors in these systems, and we also introduce their applications in emerging photocatalytic schemes such as photoredox catalysis and photoreforming.

### 6.1 General Principle and System Design

#### 6.1.1 Nanocrystal–Molecular Catalyst or Enzyme Hybrids

**Figure 33:**
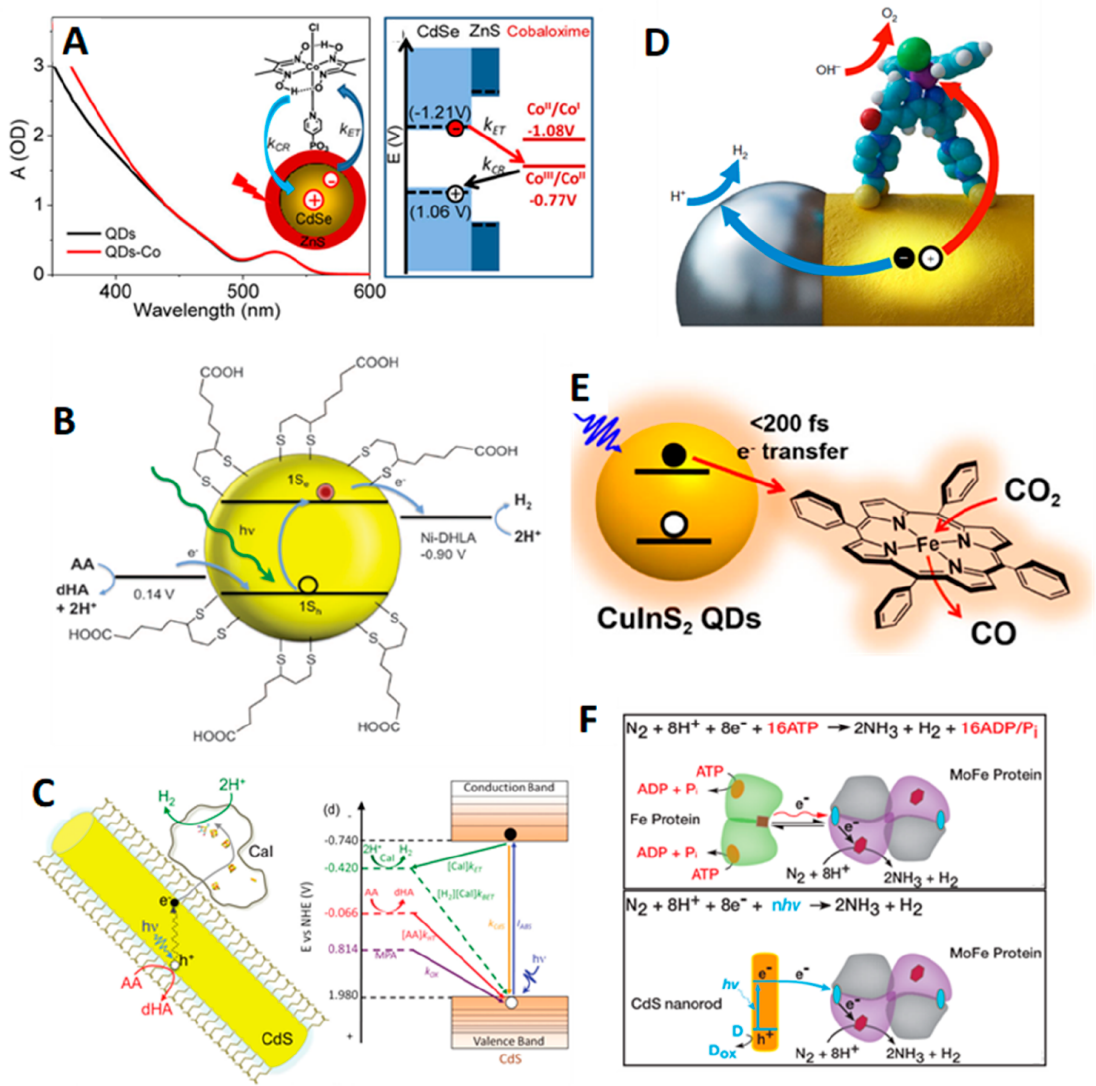
Design of various NC–molecular catalyst or enzyme systems. (A) CdSe/ZnS core/shell QDs coupled with a cobaloxime molecular catalyst for H_2_ generation. (B) CdSe QDs coupled with an in situ formed Ni-DHLA catalyst for H_2_ generation. (C) CdS NRs coupled with *Clostridium acetobutylicum* [FeFe]-hydrogenase I (CaI) for H_2_ generation. (D) CdS-Pts coupled with a Ru(tpy)(bpy)Cl_2_-based oxidation catalyst for overall water splitting. (E) CuInS_2_ QDs coupled with a *meso*-tetraphenylporphyrin iron(III) chloride (FeTPP) catalyst for the reduction of CO_2_ to CO. (F) CdS NRs coupled with a nitrogenase molybdenum–iron (MoFe) protein for the reduction of N_2_ to NH_3_. (A) Adapted with permission from ref (409). Copyright 2012 American Chemical Society. (B) Adapted with permission from ref (413). Copyright 2012 American Association for the Advancement of Science. (C) Adapted with permission from ref (418). Copyright 2012 American Chemical Society. (D) Adapted with permission from ref (425). Copyright 2014 Nature Publishing Group, Macmillan Publishers Limited. (E) Adapted with permission from ref (426). Copyright 2017 American Chemical Society. (F) Adapted with permission from ref (427). Copyright 2016 American Association for the Advancement of Science.

These hybrids combine the light-harvesting and charge-donating capabilities of NCs with the catalytic performance of artificial transition-metal-based molecular catalysts or natural enzymes. Cobaloxime-based molecules have long been known to catalyze the reduction of protons to molecular H_2_.^405−408^ Chen and co-workers demonstrate that photogenerated electrons in CdSe/ZnS core/shell QDs can be transferred to surface-attached cobaloxime molecules with high efficiency and can be subsequently used to reduce protons to H_2_ (Figure 33A).^409^ In related studies, other Co-based^410^ and Ni-based molecules^411,412^ are also shown to be efficient electron acceptors for NCs and catalysts for H_2_ generation. In addition to these specially designed molecular catalysts, it has been found that many transition metal complexes formed *in situ* by adding transitional metal salts into water-soluble NCs are efficient molecular catalysts for H_2_ generation.^409,410,413−415^ The pioneering work by Krauss and co-workers reported that a Ni–dihydrolipoic acid (DHLA) complex formed on the surface of CdSe QDs catalyzed H_2_ generation with a quantum efficiency of >36% (Figure 33B).^413^ Similarly, Wu and co-workers reported several similar *in situ* formed catalysts by adding nickel or cobalt salts to aqueous solutions of QDs with mercaptopropionic acid (MPA) ligands.^416^ For the rational design and improvement of these *in situ* catalysts, their structures and reaction mechanisms need to be elucidated. There have also been extensive efforts on photodriven H_2_ generation using NCs coupled to natural enzymes^417−419^ (such as [FeFe]- and [FeNi]-hydrogenases) or their artificial analogues typically containing Fe–S carbonyl assemblies to mimic the functional subsites of hydrogenase.^420−423^ These works are mostly motivated by the fact that natural enzymes can efficiently catalyze the reduction of proton into H_2_ with minimal overpotentials, which is typically unachievable with man-made metallic or organometallic catalysts. For example, King and co-workers have shown that complexes of MPA-capped CdS NRs with *Clostridium acetobutylicum* [FeFe]-hydrogenase I (CaI) can photocatalyze H_2_ generation with an apparent quantum efficiency (AQE) of ∼20% under illumination at 405 nm (Figure 33C),^418^ which is superior to the performance of Pt-tipped CdS NRs measured under similar conditions (with AQE < 10%).^49,424^

Among the various photocatalytic reactions, proton reduction to H_2_ is a relatively simple one in terms of both thermodynamic and kinetic aspects. Indeed, by optimizing various charge separation and catalytic process in a system, near-unity efficiency H_2_ generation has already been achieved.^428,429^ In recent years, researchers have attempted to tackle more challenging reactions such as water oxidation, CO_2_ reduction, and N_2_ fixation. These reactions involve many more electrons and are kinetically more demanding than H_2_ generation. In these efforts, many efficient catalysts have been developed and, in principle, quantum-confined NCs can be coupled with these catalysts to perform photodriven reactions. In a recent report, Stolarczyk and co-workers demonstrate that Pt-tipped CdS NRs can efficiently transfer photogenerated holes to Ru(tpy)(bpy)Cl_2_-based water oxidation catalysts to generate O_2_ (Figure 33D).^425^ As a result, this system, for the first time, can perform overall water-splitting using colloidal NCs, although the AQE is low (∼0.27%). As for CO_2_ reduction, Weiss and co-workers report that CuInS_2_/ZnS QDs with very energetic CB edges (∼ −2.5 V vs SCE) can transfer electrons to a *meso*-tetraphenylporphyrin iron(III) chloride (FeTPP) in <200 fs, which is leveraged to selectively reduce CO_2_ to CO (Figure 33E).^426^ The AQE of the reaction remains low (∼0.01%), but is already many-fold better than similar systems using Ir(ppy)_3_ (∼0.0013%) or 9-cyanoanthracene (0.0008%) sensitizers. N_2_ fixation is challenging because the cleavage of the nitrogen-nitrogen triple bond has a very large activation barrier. To date, very few man-made catalysts for the reduction of N_2_ to NH_3_ under mild conditions have been reported,^430−433^ while natural nitrogenase enzymes can perform N_2_ fixation under ambient conditions using chemical energy released from the hydrolysis of adenosine 5′-triphosphate (ATP). King and co-workers show that it is possible to replace the chemical energy with the photoexcitation energy to reduce N_2_ to NH_3_ using CdS NRs coupled with molybdenum–iron (MoFe) nitrogenase (Figure 33F).^427^ The turnover rate of the photodriven reaction already reaches 63% of the ATP-coupled reaction rate, showing the great potential of this hybrid approach in N_2_ fixation.

#### 6.1.2 Nanocrystal–Redox Mediator–Catalyst Triads

This type of systems utilizes a redox mediator to transfer charge between NCs and catalysts, which is partially inspired by natural photosynthetic systems. Charge recombination between NCs and catalysts can in principle be suppressed in this design. Fundamentally, this design also enables more mechanistic insights into the photocatalytic systems as it allows for detailed spectroscopic studies by using the well-established spectroscopic feature of redox mediators as a handle. For example, photoreduction of methyl viologen (MV^2+^) is often used as a model reaction to examine the performance of photocatalytic systems because MV^2+^ is a well-known one-electron acceptor,^151,434^ and redox mediator for H_2_ evolution.^206,435,436^

**Figure 34:**
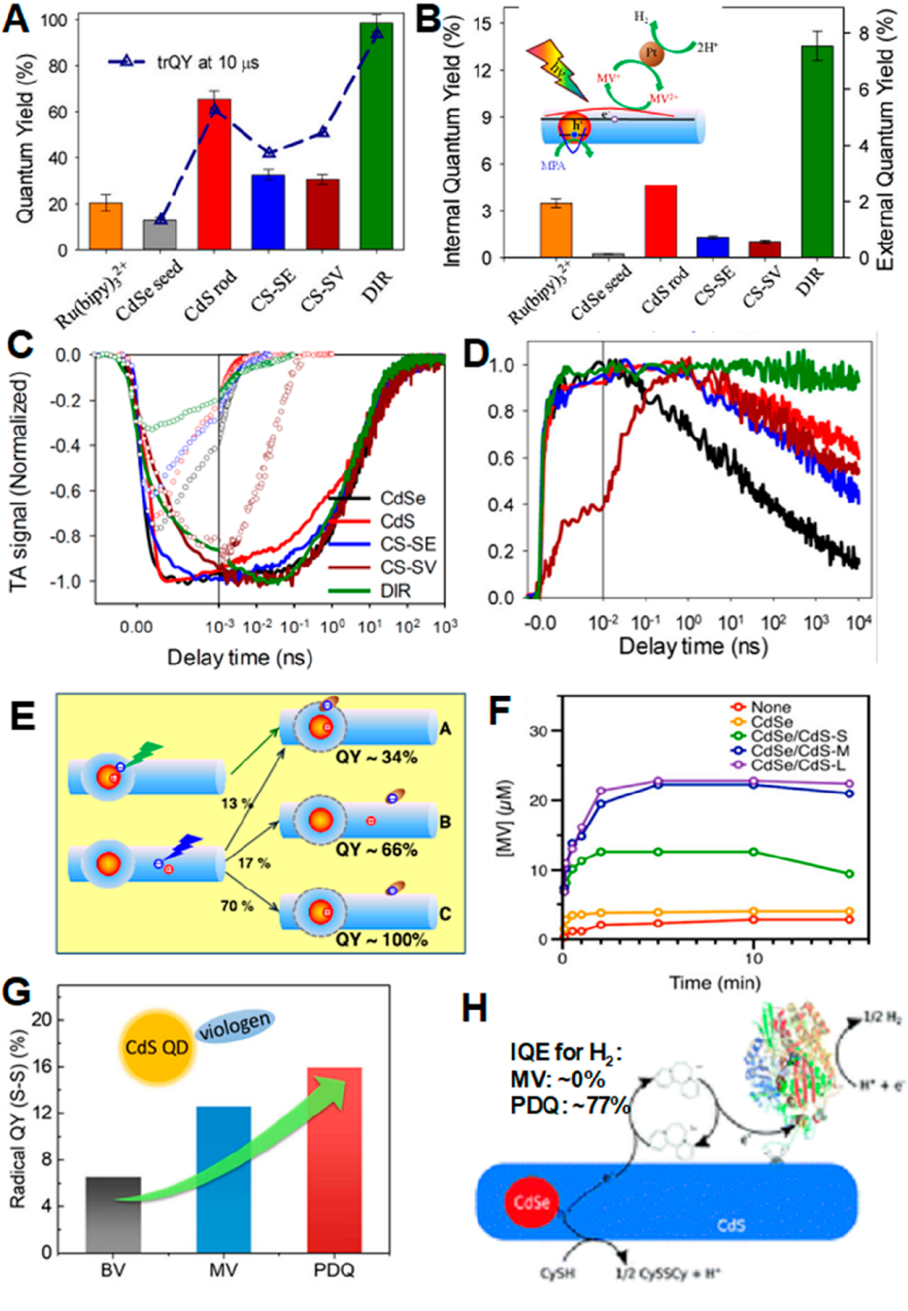
NC–redox mediator systems for photocatalysis. (A) Steady-state MV^•+^ radical photogeneration QYs using different light harvesters: Ru(bipy)_3_^2+^, CdSe QD seed, CdS NR, CdSe@CdS dot-in-rod NRs, CdSe/CdS core/shell QDs of similar lowest exciton energy (CS-SE), and CdSe/CdS core/shell QDs of similar volume (CS-SV) as the CdSe@CdS NRs. Also plotted are the transient QYs (open triangles) at 10 μs obtained from TA measurements. For experimental conditions, see ref (206). (B) Internal (left axis) and external (right axis) QYs for H_2_ generation from the systems in (A) coupled with Pt nanoparticles, as illustrated by the scheme in the inset. (C) Electron transfer kinetics from various NCs to MV^2+^ measured from the TA kinetics of exciton bleach features. The lines and circles are NCs without and with MV^2+^, respectively. (D) Formation and decay kinetics of MV^•+^ radicals for various NCs. The color coding is the same as that in (C). (E) Charge separation mechanisms and MV^•+^ radical generation QYs of ZnSe@CdS dot-in-rod NRs under 415 (bottom) and 550 nm (top) excitations. The partition ratios and MV^•+^ generation QYs of different states are labeled. (F) The concentration of MV^•+^ radicals generated by photoreduction using CdSe NRs and CdSe@CdS dot-in-rod NRs with different lengths (S, short, ∼25 nm; M, medium, ∼32 nm; L, long, ∼54 nm). (G) Radical generation QY for CdS QDs coupled with three different mediators, BV^2+^, MV^2+^, and PDQ^2+^. (H) Scheme of a H_2_ generation system comprising CdSe@CdS rod-in-rod NRs, PDQ^2+^ redox mediators, and [NiFe] hydrogenase. The H_2_ generation QYs for using MV^2+^ and PDQ^2+^ mediators are indicated. (A–D) Adapted with permission from ref (206). Copyright 2012 American Chemical Society. (E) Adapted with permission from ref (437). Copyright 2014 Royal Society of Chemistry. (F) Adapted with permission from ref (438). Copyright 2015 American Chemical Society. (G) Adapted with permission from ref (439). Copyright 2018 American Chemical Society. (H) Adapted with permission from ref (440). Copyright 2017 Royal Society of Chemistry.

In a previous study, Lian and co-workers compared the performances of various NCs and also the benchmark Ru(bipy)_3_^2+^ molecule for MV^•+^ generation and H_2_ generation.^206^ These NCs include CdSe core-only QDs, CdSe@CdS core/shell QDs, CdS NRs, and CdSe@CdS dot-in-rod NRs. The as-synthesized NCs were transferred to the aqueous phase by replacing native ligands with MPA ligands that also function as sacrificial electron donors. When measured under the same conditions, the radical generation QYs show the following trend (Figure 34A): CdSe@CdS NRs (∼98%) > CdS NRs (∼65%) > CdSe@CdS core/shell QDs (∼31%) > Ru(bipy)_3_^2+^ (∼20%) > CdSe QD (∼11%).^206^ Pt nanoparticles were added as electron acceptors for MV^•+^ radicals and as H_2_ evolution catalysts. As expected, the H_2_ generation efficiencies follow the trend of those for MV^•+^ generation (Figure 34B). However, the absolute values for the former are much lower than those for the latter. In particular, the MV^•+^ generation efficiency is essentially unity for CdSe@CdS NRs, but the internal quantum efficiency for H_2_ generation is only ∼13%.

In order to rationalize the different performances between these NCs, TA measurements were performed. Both the decay of exciton bleach features of NCs (Figure 34C) and the formation of a MV^•+^ radical absorption band (Figure 34D) can be used to follow NC-to-MV^2+^ electron transfer kinetics. As the exciton bleach in NC-MV^2+^ complexes decays much faster than free NCs, the electron transfer yield is near 100% for all NCs. However, the lifetimes of MV^•+^ radicals generated by these NCs are different (Figure 34D), reflecting the different recombination rates between MV^•+^ and holes in NCs. Specifically, the recombination is negligible within 10 ps for CdSe@CdS NRs, accounting for their near-unity MV^•+^ generation yield. Detailed measurements showed that the NR-to-MPA hole transfer process (0.31 ns) was much faster than charge recombination between MV^•+^ radicals and holes in NRs (320 ns). In contrast, the fast recombination in core-only QDs leads to large recombination loss and a small steady-state QY.^206^ CdSe@CdS NRs show the best MV^•+^ generation performance compared to both CdSe@CdS core/shell QDs with similar confinement energy and QDs with similar total NC volume because the former and the latter suffer from fast charge recombination and slow charge separation, respectively. This comparison highlights the importance of NC morphology in controlling charge separation and recombination for efficient photocatalysis.

A further demonstration of the role of the morphology of hetero-NRs in promoting charge separation and suppressing charge recombination was reported in our excitation wavelength dependence study of MV^•+^ generation using type II ZnSe@CdS dot-in-rod NRs.^437^ The radical generation yield was ∼90% under 415 nm excitation but became much lower (∼34%) under 550 nm excitation. Detailed spectroscopic studies show that the excitation wavelength controls the charge separation and recombination pathways for NR-MV^2+^ complexes (Figure 34E). In the case of 550 nm excitation, excitons are generated near the ZnSe core and the NRs essentially function as ZnSe@CdS core/shell QDs. The excitons are dissociated by electron transfer to MV^2+^ to form charge-separated state A. Due to the relatively fast charge recombination rate of this state, the MV^•+^ generation yield is only ∼34% for this state. In contrast, in the case of 415 nm excitation, a competition between exciton localization to near the core, exciton trapping along the rod, and exciton dissociation by electron transfer to MV^2+^ leads to the formation of three charge-separated states A, B, and C. State C, in particular, has a unity MV^•+^ generation yield, as the charges are separated over a longer distance. The partition between states A, B, and C leads to an averaged MV^•+^ generation yield of ∼90% under 415 nm excitation. This mechanism also explains the rod-length-dependent MV^•+^ generation yield reported for CdSe@CdS dot-in-rod NRs by Kamat and co-workers (Figure 34F).^438^ Longer NRs presumably give a higher partition ratio for the charge-separated state C, as it becomes more difficult for exciton transport to the core to compete with direct exciton dissociation by electron transfer to MV^2+^. In a related study, Lian and co-workers also found this type of competition between exciton localization and dissociation for the homojunction CdSe tetrapods.^243^

In addition to NC morphologies, the type of redox mediator used in the reaction also strongly affects the yields of radical generation and subsequent H_2_ generation. In a recent work, Lian and co-workers compared the photoreduction efficiency of three mediators, namely, propyl-bridged diquat (PDQ^2+^), MV^2+^, and benzyl viologen (BV^2+^), using CdS QDs.^439^ The steady-state radical generation yields follow the trend of BV^2+^ < MV^2+^ < PDQ^2+^, which appears to contradicts the reduction potentials of these mediators (−370, −448, and −550 meV vs NHE for BV^2+^, MV^2+^, and PDQ^2+^, respectively). TA measurements indicate the electron transfer yields are near unity for all three mediators; however, charge recombination rates between radicals and holes in CdS QDs follow the trend of BV^2+^ > MV^2+^ > PDQ^2+^, likely because charge recombination falls in the Marcus inverted region. Thus, the steady state radical generation yields are limited by charge recombination in this system. The redox potential of the mediator not only affects its own reduction efficiency but also controls the efficiency of the coupled H_2_ generation reaction. Dyer and co-workers studied light-driven H_2_ generation from a system consisting of CdSe@CdS NRs, MV^2+^, or PDQ^2+^ mediators and [NiFe]-hydrogenases.^440^ They found that although the electron transfer rate from NRs to MV^2+^ is much faster than that for PDQ^2+^ due to a larger electron transfer driving force for the former, the IQE of H_2_ generation is ∼77% for PDQ^2+^ but is essentially zero for MV^2+^, as MV^•+^ cannot donate electrons to the [NiFe]-hydrogenase. It is concluded that in these systems the redox potential of the mediators should be chosen to balance the electron transfer from NCs to mediators and the subsequent electron transfer from mediators to catalysts.

#### 6.1.3 All-Inorganic Heteronanocrystals for Photocatalysis

**Figure 35:**
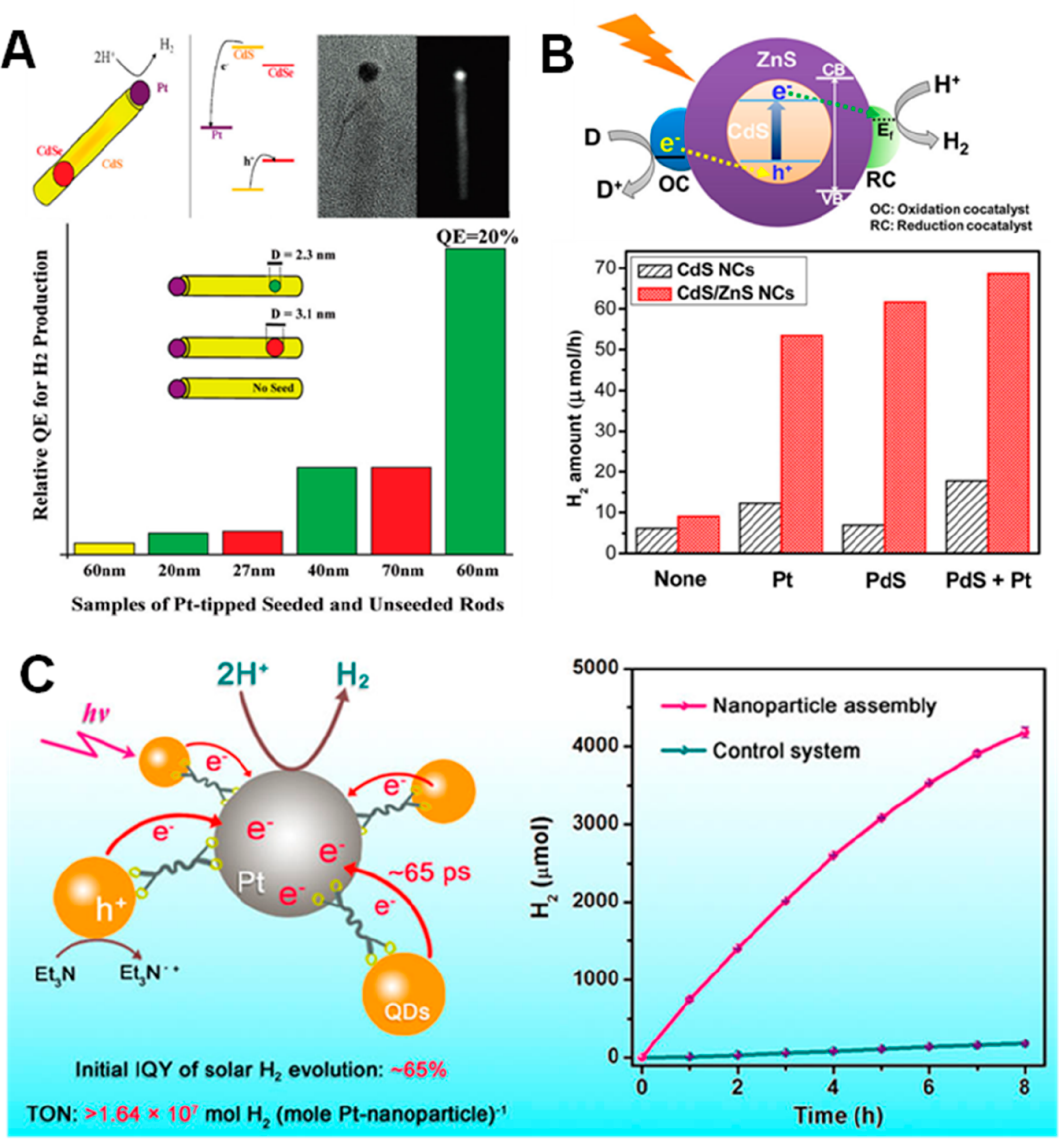
All-inorganic NC systems for photocatalysis (A) Structural scheme (left), energy level scheme (middle), and TEM images (right) of the all-inorganic CdSe@CdS-Pt NRs (top). Relative quantum efficiency of H_2_ generation for NRs with different core sizes and rod lengths (bottom). (B) Scheme of the CdS/ZnS core/shell QDs loaded with dual cocatalysts for H_2_ generation. Amounts of generated H_2_ for different system designs (bottom). (C) Scheme of a self-assembled framework for H_2_ generation comprising cross-linked CdSe QDs and Pt nanoparticles (left). Amounts of generated H_2_ for the assembly and an unassembled control (right). (A) Adapted with permission from ref (49). Copyright 2010 American Chemical Society. (B) Adapted with permission from ref (455). Copyright 2013 American Chemical Society. (C) Adapted with permission from ref (456). Copyright 2017 American Chemical Society.

All-inorganic hetero-NCs combine the light-harvesting and charge-donating capabilities of quantum-confined semiconductor NCs with the catalytic functions of another type of nanoparticles (often metal) to realize the goal of an “all-in-one” photocatalytic system. Banin and co-worker reported the synthesis of Au-tipped CdSe NRs as the first example for this type of heterostructure.^441^ These Au-tipped NRs have been applied in photocatalysis reactions such as dye degradation and H_2_ generation,^442,443^ suggesting charge separation at the semiconductor–metal interfaces. Later, Habas and co-workers reported the synthesis of Pt-tipped CdS NRs by thermal reduction of Pt(II) salts.^444^ These NRs are more suited for solar-to-fuel conversion, as Pt is one of the best catalysts for the hydrogen evolution reaction.^445^ In a related work, Dukovic and co-workers achieved photodeposition of Pt on CdS NRs,^446^ where the electrons generated by photoexcitation of CdS NRs were used to reduce Pt salts and led to nucleation of multiple Pt nanoparticles on CdS NRs. Metal (including Au, Pt, and Ni) decorated NPLs have also been reported.^154,223,447,448^ These Pt-tipped NRs and NPLs have been intensely studied for solar-driven H_2_ generation reactions.^49,424,447−453^ In particular, Alivisatos and co-workers demonstrate in their pioneering work that the photocatalytic H_2_ generation efficiencies of Pt-tipped CdSe@CdS dot-in-rod NRs (CdSe@CdS-Pt NRs) can be systematically tuned using the CdSe core sizes and CdS rod lengths (Figure 35A).^49^ Specifically, they find that for NRs with the same core size, the H_2_ generation efficiency increases with the rod length, presumably because of increase charge separation distance in longer NRs; when rod lengths are comparable, NRs with a smaller CdSe core (2.3 nm) show higher activity than those with a larger core (3.1 nm), also presumably due to a better charge separation enabled by the more delocalized electrons for smaller cores. Kuno and co-workers first reported the light-driven H_2_ generation performance of Ni-decorated CdS NPLs in aqueous solution with apparent quantum efficiencies of ∼64% for the first 2 h and ∼25% for 40 h, demonstrating a great potential of 2D NPLs in photosynthesis systems.^448^ Bigall and co-workers studied how the H_2_ generation performances of CdSe/CdS core/crown NPL-Pt heterostructures change with core and crown size and found that the larger the core and crown, the better the performance.^223^

Recently, Amirav and co-workers showed that the functionalities of CdSe@CdS-Pt NRs can be further expanded by adding an oxidation catalyst, as exemplified by the Ru-CdSe@CdS-Pt NRs.^454^ These NRs are good models for fully integrated all-inorganic artificial photosynthetic systems, but their photocatalytic performances have not been tested. In a related study, Li and co-workers reported CdS core and CdS@ZnS core/shell QDs coloaded with reduction catalyst Pt and oxidation catalyst PdS (Figure 35B).^455^ They found that the dual-catalysts-loaded QDs indeed have a better H_2_ generation performance than QDs loaded with either Pt or PdS only, presumably because the best charge separation can be achieved in dual-catalysts-loaded samples. It is also interesting to note that for CdS core QDs Pt-loaded samples perform better than PdS-loaded ones, while for CdS/ZnS core/shell QDs PdS-loaded samples perform better. It suggests that hole extraction from core/shell QDs by sacrificial donors is difficult and an oxidation catalyst can improve the photocatalytic performance by accelerating hole transfer.

All-inorganic heterostructures are not limited to those with directly attached domains. They can be fabricated by cross-linking NCs with catalytic nanoparticles, as demonstrated by Wu and co-workers.^456^ This strategy is important, as the attachment of catalytic nanoparticles such as Pt onto small-size NCs such as strongly quantum-confined QDs is challenging. In addition, in the case of a 1:1 QD/Pt ratio, the absorbance of Pt will dominate over that of the QDs, diminishing the apparent QY of a photocatalytic system. Wu and co-workers effectively overcome these issues by designing a self-assembled architecture of QDs and Pt nanoparticles where these two are jointed together by molecular polyacrylate (Figure 35C, left). Many QDs can be attached with one Pt nanoparticle to ensure light absorption dominated by QDs; meanwhile, the distance between QDs and Pt can be tuned by the length of the polyacrylate chain such that electron transfer from QDs to Pt is still efficient. As a result of this unique design, the system achieved very fast H_2_ generation (Figure 35C, right) with an internal quantum yield of ∼65%. This strategy can in principle be extended to various combinations of NCs and catalytic nanoparticles, greatly expanding the scope of all-inorganic photocatalysts.

### 6.2 Charge Separation in Photocatalytic Systems Using Nanocrystals

In all system designs described above, a critical step determining their photocatalytic efficiencies is the charge separation and recombination between the light-harvesting NCs and catalytic molecules or particles. In the redox mediator approach, charge separation and recombination between NCs and mediators are often relatively easy to measure due to the well-established spectral features of these mediators. As such, these have been discussed above along with the system descriptions. In comparison, such a spectroscopic handle is often not as straightforward to establish for molecular or nanoparticle catalysts. In this section, we review progress made toward understanding charge separation and recombination dynamics between NCs and molecular or nanoparticle catalysts.

#### 6.2.1 Charge Separation in Nanocrystal–Molecular (Enzyme) Catalyst (Or Enzyme) Hybrids

Due to the large number of binding sites available on the surface of NCs, molecular or enzyme catalysts usually interact with NCs through the so-called static quenching mechanism, *i.e.*, charge transfer from NCs to surface-adsorbed catalysts. This is important as the exciton lifetimes of NCs are typically much shorter than the triplet excited states of molecular light-harvesters such as Ru and Ir complexes. While the latter can achieve efficient charge separation via the diffusion controlled dynamic quenching mechanism,^457^ a short NC–catalyst distance is required for efficient charge separation for NCs.

**Figure 36:**
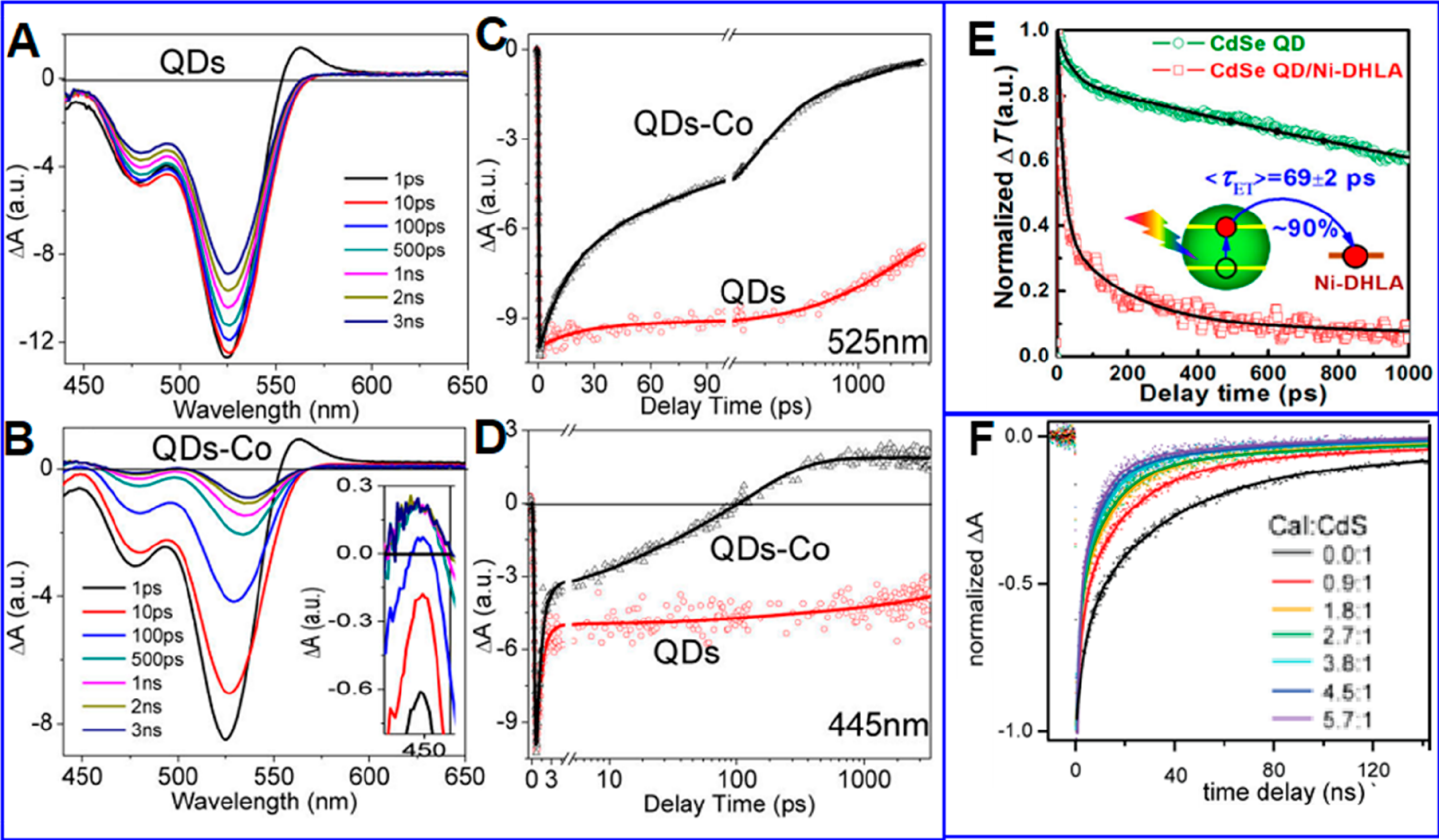
Charge separation dynamics in NC–catalyst hybrids. (A and B) TA spectra of QDs (A) without and (B) with the cobaloxime catalyst. (C and D) TA kinetics probed at (C) 525 nm and (D) 445 nm for QDs without (red) and with the cobaloxime catalyst (black). (E) TA kinetics of CdSe QDs with (red) and without (green) the Ni-DHLA catalyst. (F) TA decay kinetics (at 470 nm) of CdS–CaI samples with varying CaI/CdS ratios. (A–D) Adapted with permission from ref (409). Copyright 2012 American Chemical Society. (E) Adapted with permission from ref (459). Copyright 2015 American Chemical Society. (F) Adapted with permission from ref (419). Copyright 2014 American Chemical Society.

Chen and co-workers studied charge separation between CdSe/ZnS core/shell QDs and surface-adsorbed cobaloxime hydrogen evolution catalysts using TA spectroscopy.^409^ The exciton bleach recovery in QD–cobaloximes is much faster than that of free QDs (Figure 36A and B). Energetic considerations indicate that electron transfer is the only exciton quenching mechanism by cobaloxime. Thus, the accelerated bleach recovery suggest fast electron transfer from the CB of QDs to the Co(III)/Co(II) redox level of cobaloxime. Fitting the bleach recovery kinetics reveals an electron transfer time constant of ∼105 ps (Figure 36C). In addition to following the exciton bleach feature, they find that the TA spectra of QD-cobaloximes at >100 ps exhibit a positive TA feature that is absent in free QDs (Figure 36B inset). This feature could arise from the absorption of Co(II) or charge-separation-induced absorption shifts of QDs via a transient stark effect. In either case, it confirms electron transfer from QDs to cobaloxime; fitting the kinetics at this feature gives a consistent electron transfer time constant (Figure 36D). The kinetics of this feature also suggests that the charge-separated state is long-lived, showing negligible decay within 3 ns.

Hydrogen evolution requires the reduction of cobaloxime by two electrons. In the experiments performed under single exciton conditions (Figure 36A–D), the second electron transfer from QDs to the Co(II)/Co(I) redox level of cobaloxime is not observable. By exciting QDs with at least two excitons, one can, in principle, realize two electron reduction of cobaloxime. In reality, there exists an intrinsic challenge for this goal. Specifically, the ultrafast electron transfer observed by Chen and co-workers is enabled by the presence of 66 cobaloxime molecules on each QD. As the reduction potential of Co(II)/Co(I) is ∼0.3 V more negative than that of Co(III)/Co(II), the second electron should preferentially transfer to an unreduced cobaloxime, resulting in two catalysts in the Co(II) state instead of one catalyst in the desired Co(I) state. On the other hand, if there is only one cobaloxime molecule per QD, the electron transfer rate would be much slower due to the additive nature of electron transfer channels. Besides, as we review above, realizing a 1:1 QD/catalyst ratio is not easy due to the Poisson distribution for the adsorption events, where the probability of a QD to adsorb *n* catalyst is *P*_*n*_ (see eq 5.1 and details of the Poisson distribution in section 5.2). Thus, the ultimate goal should be to assemble QD–catalyst complexes in a 1:1 ratio while ensuring efficient charge separation at this ratio. Recently, Wasielewski and co-workers reported the simultaneous transfer of two electrons from one CdS QD to an attached extended-viologen cylophane.^458^ Such demonstration for realistic hydrogen evolution or other types of catalysts, however, is still lacking.

There have been extensive studies on the charge separation kinetics in many other NC–molecular catalyst or enzyme systems. For example, electron transfer from CdSe QDs to the *in situ* generated Ni-DHLA catalysts was studied using TA spectroscopy by Krauss and co-workers (Figure 36E).^459,460^ It was reported that an electron transfer time constant of ∼69 ps and transfer yield of ∼90% can be realized in QD–Ni-DHLA complexes with a QD/Ni-DHLA ratio of ∼1:20^459^ by comparing the exciton bleach recovery kinetics of QDs with and without Ni-DHLA (Figure 36E). This efficient charge separation is a prerequisite for the efficient H_2_ generation (AQE ∼ 36%). Dukovic and co-workers studied the charge separation dynamics in CdS NR-hydrogenase (CaI) hybrids using TA spectroscopy (Figure 36F), as well as by comparing the exciton bleach recovery kinetics of NRs with and without CaI.^419^ They reported an electron transfer time of ∼100 ns and yield of ∼47% for a CaI/NR ratio of 1:1. The charge separation yield increases with the CaI:NR ratio (Figure 36F) and reaches ∼89% for a CaI/NR ratio of 10:1. Their previous work showed that the AQY of light-driven H_2_ generation from NR–CaI hybrids with a CaI/NR ratio of 0.67:1 was ∼20%, for which the charge separation yield should be <47%. This H_2_ generation performance is exceptional given that Pt-tipped CdS NRs with virtually 100% charge separation yields display AQYs of <10% (see more discussions in the following section). The authors speculate that the electron injected to the distal F-cluster of CaI diffuses inside the enzyme before being captured by the active site (H-cluster) that is several nm away from the F-cluster, effectively suppress charge recombination between the catalytic center and the hole located in the NR and/or oxidized sacrificial donors. This mechanism showcases the advantage of natural enzymes over man-made catalysts.

It is noteworthy that in the studies shown in Figure 36E and F and many related examples, electron transfer kinetics is derived solely from the exciton bleach feature of NCs. A more unambiguous demonstration should include the observation of signals from reduced products, which, on the other hand, also allows for measuring the lifetime of charge-separated states. However, these signals are often too weak to be resolved or simply absent in the commonly studied UV–vis–IR ranges. In recent years, X-ray TA spectroscopy has proven to be a unique tool to probe charge transfer dynamics, as X-ray absorption is sensitive to the oxidation state of the elements in catalytic centers. For example, Huang and co-workers reported direct evidence for the reduction of Co(III) to Co(II) upon electron transfer from QDs to cobaloxime catalysts using X-ray TA based on a synchrotron light source.^461^ The time resolution of this type of X-ray TA is often limited to hundreds of ps. Recent developments in X-ray free electron lasers (FEL) offer a better solution for X-ray TA by use of femtosecond X-ray pulses. This technique is becoming a powerful tool to study charge transfer in catalysts in the years to come.

#### 6.2.2 Electron and Energy Transfer from Photoexcited Nanocrystals to Attached Metals

**Figure 37:**
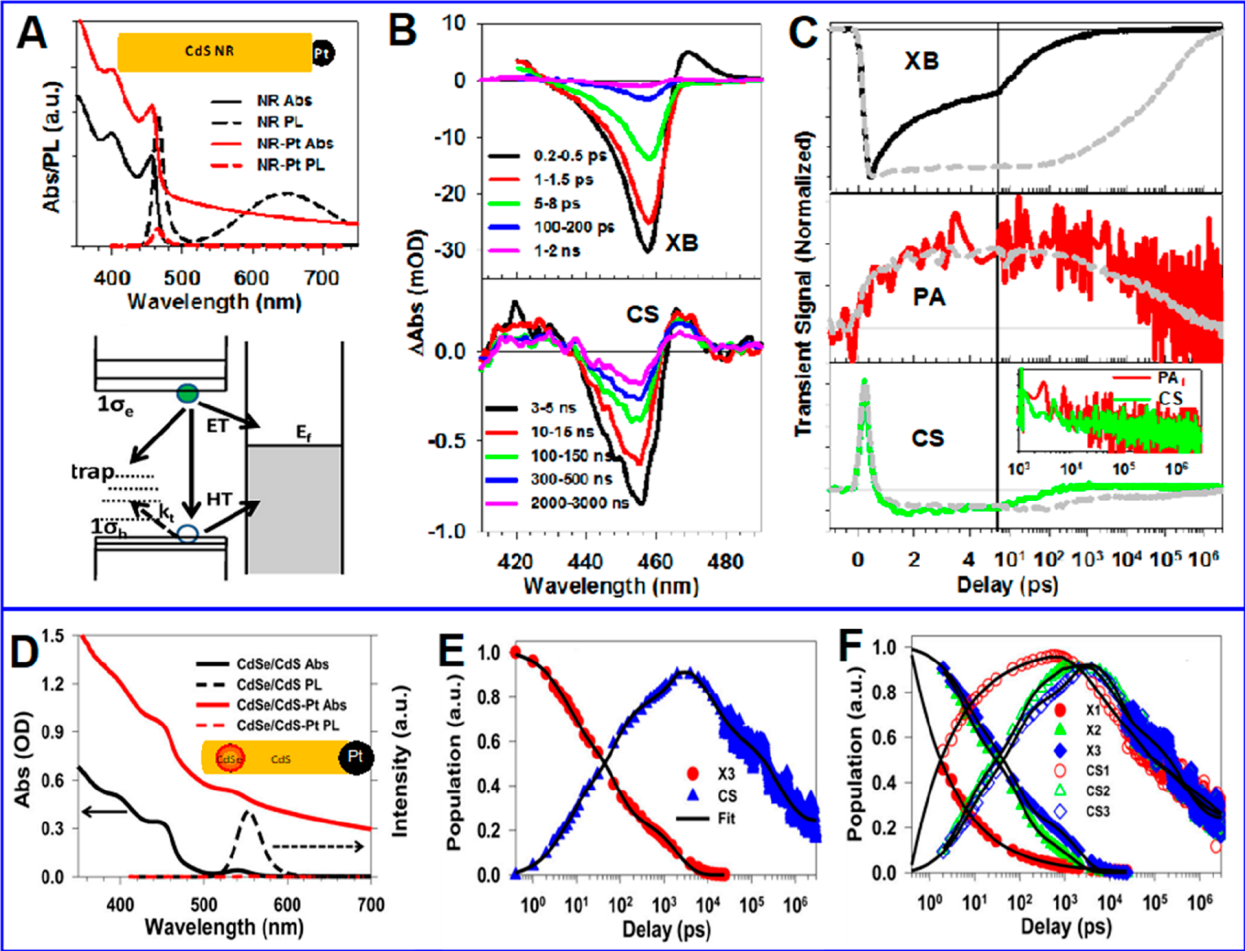
Charge transfer in NR-Pt heterostructures. (A) Absorption (solid lines,) and emission (dashed lines) spectra of CdS NRs (black) and CdS-Pt NR heterostructures (red) (top). Schematic energy levels and possible exciton quenching pathways in CdS-Pt NR heterostructures (bottom). (B) TA spectra of CdS-Pt NRs at indicated time delays following 400 nm excitation. (C) TA kinetics of exciton bleach (XB, black solid line, top), photoinduced absorption (PA, red solid line, middle), and charge-separated state (CS, green solid line, bottom) spectral features for CdS-Pt NRs. Also shown for comparison are corresponding kinetics for free CdS NRs (gray dashed lines) at the same wavelengths. The inset shows a comparison of CS and PA kinetics in CdS-Pt after 1 ns. (D) Absorption (solid lines, left axis) and emission (dashed lines, right axis) spectra of CdS NRs (black) and CdS-Pt NR heterostructures (red). (E) TA kinetics probed at the exciton bleach (red circles) and charge-separated state (blue triangles) features of CdSe@CdS-Pt NRs following excitation of the CdSe core. (F) TA kinetics probed at three different exciton bleach features (X1, X2, and X3) and three corresponding charge-separated state features (CS1, CS2, and CS3) of CdSe@CdS-Pt NRs following excitation of the CdS rod. (A–C) Adapted with permission from ref (450). Copyright 2012 American Chemical Society. (D–F) Adapted with permission from ref (424). Copyright 2014 American Chemical Society.

The interaction mechanisms between semiconductor NCs and attached metallic catalysts are intrinsically more complex than those between NCs and molecular catalysts due to the continuous bands of metals as compared to the discrete levels of molecules. Based on a typical band alignment between metal and semiconductor using CdS and Pt as an example (Figure 37A, bottom), the metallic domain can quench the excitons in the semiconductor domain via electron, hole, and energy transfer channels. For photodriven H_2_ generation, only electron transfer is the useful mechanism. Thus, it is fundamentally important to understand how electron transfer competes with energy and hole transfer channels and how long-lived charge separation can be achieved for the rational design of such integrated semiconductor–metal photocatalysts.

In our previous study, Lian and co-workers investigated the effect of a metal tip on exciton dynamics in the semiconductor NR using CdS-Pt NRs as a model system.^450^ The absorption spectrum of CdS-Pt NRs contains both the excitonic absorption bands of CdS NRs and the broad absorption of Pt tips (Figure 37A, top).^462^ Both band edge and trap state emissions of CdS NRs are strongly quenched by the Pt tips. To determine whether the quenching mechanism of excited CdS NRs by Pt is electron, hole, or energy transfer, TA spectroscopy was applied to investigate the detailed carrier dynamics.^450^ TA spectra of CdS-Pt NRs excited at 400 nm show the fast recovery of the exciton bleach (XB) feature of CdS NRs on a few ps time scale (Figure 37B, top) and the formation of a set of derivative-like spectral features (Figure 37B, bottom). Kinetics analysis shows that during the decay of XB (Figure 37C, top) the photoinduced absorption PA feature (from 500 nm to NIR; not shown in Figure 37B) that has been assigned to trapped holes remains unaffected (Figure 37C, middle). This comparison suggests that the decay of XB is a result of electron transfer rather than hole or energy transfer processes, as the latter should also remove the hole. This charge separation process is also responsible for the formation of the derivative-like spectral features via the stark effect in the CdS^+^-Pt^–^ charge-separated state (CS). It is proposed that the ultrafast (∼0.7 ps) hole trapping on CdS NRs suppresses both hole and energy transfer pathways, enabling a near-unity electron transfer yield.^450^ The ensuing back electron transfer process from the Pt to CdS, *i.e.*, charge recombination, can be followed using both PA and CS features (Figure 37C, middle and bottom). Kinetics fitting reveals half-lives of ∼3.4 ps and ∼1.2 μs for charge separation and recombination processes, respectively. This large asymmetry in charge separation and recombination rates is also enabled by the hole trapping, as it sets up a long distance between the electron in Pt and the hole in the trap site, leading the long-lived charge-separated state. Based on this study, ultrafast hole trapping is the key to the photocatalytic H_2_ production from CdS-Pt NRs.^49,453^

Hole trapping facilitates charge separation in CdS-Pt NRs, but the nature of these hole trap sites remains unclear. A recent computational study using a semiperiodic density functional theory model for CdS NC surfaces supports the common assumption that hole trapping states are related to the nonbonding sp^3^ orbitals of sulfur atoms on the NC surfaces.^463^ Nonetheless, a way to control the spatial location and energetic depth of the trapping sites is lacking. In contrast, as we review above, in CdSe@CdS dot-in-rod NRs, the VB hole can be localized to the CdSe core with known and controllable location and depth by tuning the rod length and core size.^49,173^ As shown Figure 37D, the absorption spectrum of CdSe@CdS-Pt NRs contains features from both domains, and the emission of CdSe@CdS NRs is efficiently quenched by the Pt tips. The quenching dynamics in CdSe@CdS-Pt NRs was also investigated by TA spectroscopy.^424^Figure 37E shows the TA dynamics of CdSe@CdS-Pt NRs excited by a 540 nm pulse, which selectively excites the CdSe core. Similar to CdS-Pt NRs, the core exciton bleach (XB) feature quickly recovers and a derivative-like charge-separated state (CS) signal grows concomitantly, suggesting electron transfer from the CdSe core to the Pt tip. Fitting the kinetics reveals charge separation and recombination half-lives of ∼43.5 ps and ∼211 ns, respectively.^424^ Thus, this system also shows near-unity yield and long-lived charge separation. Similarly efficient charge separation was found for ZnSe@CdS-Pt NRs^464^ and CdSe@CdS-Pt octapods.^465^ For all these seeded structures, hole transfer is suppressed by ultrafast localization of the hole to the seed, and energy transfer is suppressed by the long distance between seed exciton and the Pt tip. Figure 37F shows the TA dynamics of CdSe@CdS-Pt NRs excited by a 400 nm pulse, which is more complicated than the case of 540 nm excitation due to the competition between exciton trapping on the rod, exciton localization to the core and exciton localization to the bulb region surrounding the core. Detailed analysis shows that the three types of excitons (*i.e.*, X1 trapped on the rod, X2 trapped on the bulb, and X3 localization to the core) have distinct electron transfer dynamics, generating three types of charge-separated states, namely, CS1, CS2 and CS3. Fitting the kinetics in Figure 37F reveals half-lives of 1.75, 30.1, and 43.5 ps for charge separation and 102, 211, and 211 ns for charge recombination for X1, X2, and X3 excitons, respectively. Thus, charge separation yields for all three types of exciton also approach unity in the case of 400 nm excitation. Note that the high charge separation yield and long-lived charge-separated states for X3 are enabled by ultrafast hole localization to the CdSe core, whereas those for X1 and X2 are enabled by ultrafast hole trapping on CdS surfaces.

**Figure 38:**
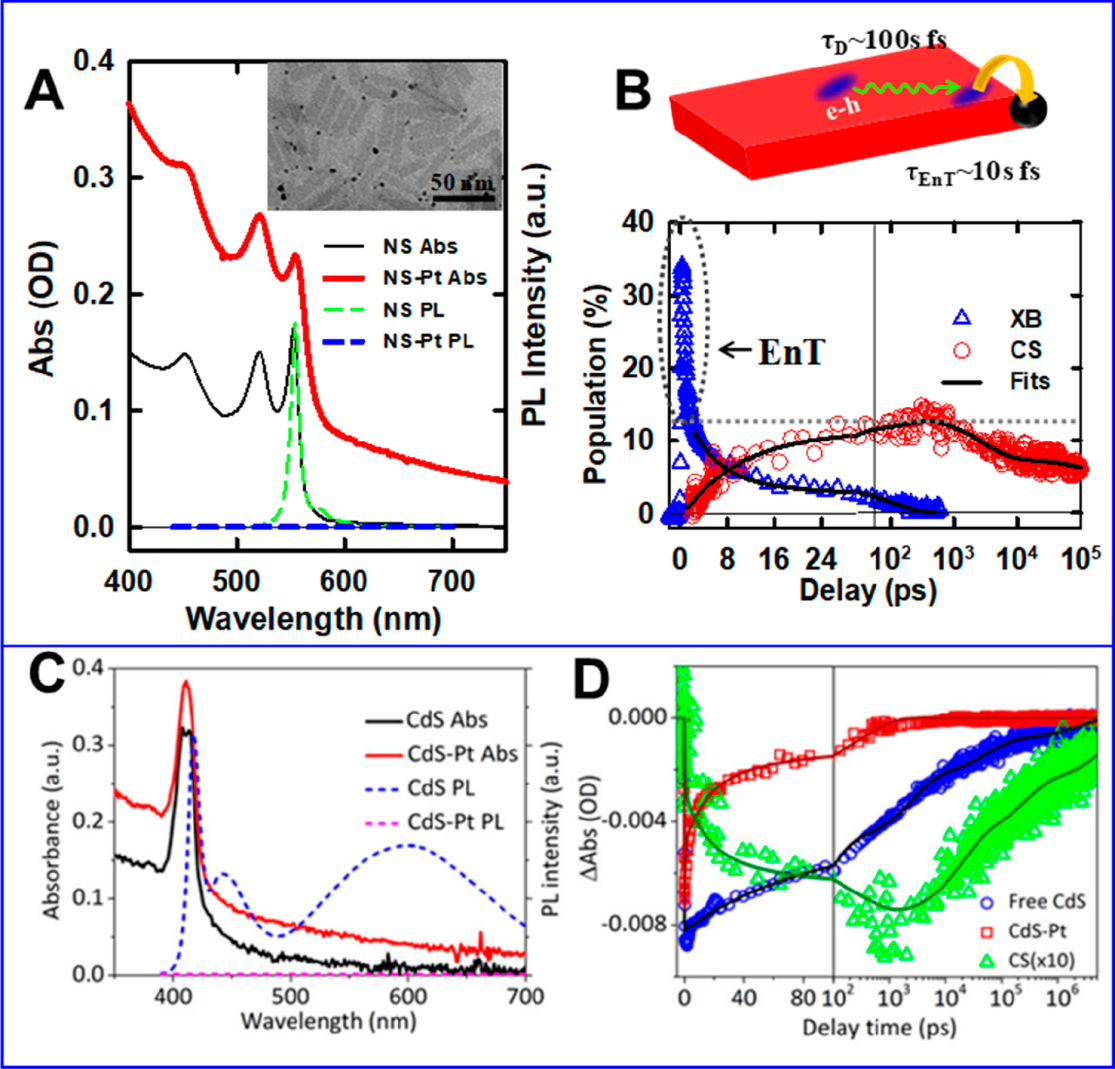
Charge and energy transfer in NPL-Pt heterostructures. (A) Absorption and emission spectra of CdSe NPLs (black solid line and green dashed line, respectively) and CdSe-Pt NPLs (red solid line and blue dashed line, respectively). Inset is a TEM image of the CdSe-Pt NPLs. (B) TA kinetics probed at the exciton bleach (blue triangles) and charge-separated state (red circles) features of CdSe-Pt NPLs following 400 nm excitation (bottom). As indicated in the figure, ∼87% of excitons in CdSe NPLs are quenched by energy transfer (EnT) to Pt. Proposed mechanism for the ultrafast EnT quenching that comprises exciton diffusion inside the NPLs on the hundreds of fs time scale, followed by interfacial EnT on the tens of fs time scale (top). (C) Absorption and emission spectra of CdS NPLs (black solid line and blue dashed line, respectively) and CdS-Pt NPLs (red solid line and pink dashed line, respectively). (D) Comparison of exciton bleach (XB) kinetics at 416 nm of CdS NPLs (blue circles) and CdS-Pt NPLs (red squares) and the scaled charge-separated state (CS) kinetics (green triangles). (A and B) Adapted with permission from ref (154). Copyright 2014 American Chemical Society. (C and D) Adapted with permission from ref (447). Copyright 2013 American Chemical Society.

The importance of hole trapping or localization for efficient charge separation in NC–metal heterostructures is further corroborated by our recent studies of exciton quenching dynamics in Pt-decorated CdSe^154^ and CdS NPLs.^447^ These NPLs have an atomically precise thickness of only a few monolayers (MLs) and are strongly quantum-confined in the thickness direction.^80,81,466−469^Figure 38A shows the absorption spectrum of 5 ML CdSe NPLs with the sharp and discrete excitonic band arising from the precise quantum confinement in their thickness. These CdSe NPLs are well passivated upon synthesis, as implied by their relatively high PLQY of ∼36% for core-only samples and the absence of a trap-related emission band in their PL spectrum (Figure 38A). TA and time-resolved PL measurements show no evidence for the sub-ps hole trapping observed in CdS NRs.^154^ Upon Pt decoration, the PL of CdSe NPLs was quenched by 100% (Figure 38A). The TA measurements show that the exciton bleach (XB) of CdSe completely recovers within ∼100 ps for CdSe-Pt NPLs (Figure 38B, bottom), consistent with PL quenching. However, the analysis of the charge-separated state (CS) signatures indicates that the decay of CS and the formation of XB are not well correlated. Specifically, the decay of 86.6% of the XB amplitude within ∼1 ps does not lead to the formation of CS, whereas only the decay of the remaining 13.4% of XB is accompanied by the formation of CS. Based on this observation, we propose that the majority of excitons (∼86.6%) are quenched by ultrafast exciton transport to the CdSe/Pt interface, followed by rapid energy transfer (schematically shown in Figure 38B, top). 13.4% of excitons are dissociated to form the desired CdSe^+^-Pt^–^ charge-separated state in ∼9.4 ps, and the charge recombination half-life is ∼75 ns, both enabled by some CdSe NPLs in the ensemble exhibiting hole trapping. Note, however, that this hole trapping is not as fast and efficient as that in CdS NRs.

Our more recent study on CdS-Pt NPLs demonstrates very different hole trapping and exciton quenching mechanisms from CdSe-Pt NPLs.^447^ The absorption spectrum of CdS NPLs shows discrete excitonic bands, but their PL spectrum shows emission from both the lowest-energy excitonic band and two much broader trap-related bands (Figure 38C). Ultrafast PL measurements show a hole trapping time constant of ∼0.2 ps for CdS NPLs, which is even faster than that of CdS NRs. Both excitonic and trap-related emission bands are completely quenched by the decorated Pt nanoparticles (Figure 38C). TA measurements show that, because of the ultrafast and efficient hole trapping, the quenching mechanism of excitons in CdS NPLs by Pt is dominated by interfacial electron transfer to form charge separation states (CS). As shown in Figure 38D, the decay of XB and the formation of CS correlated well. Fitting these kinetics reveals charge separation and recombination half-lives of ∼8.8 ps and ∼96.2 ns, respectively. These time constants are similar to those reported for CdSe-Pt NPLs, but the charge separation yield is near unity for CdS-Pt NPLs. The comparison CdSe-Pt and CdS-Pt NPLs clearly demonstrates the essential role of hole trapping in enabling selective and efficient electron transfer in these semiconductor–metal heterostructures.

### 6.3 Determining and Improving the Efficiency Limiting Steps in Photocatalytic Systems Using Nanocrystals

#### 6.3.1 Photocatalytic Efficiency-Limiting Steps

**Figure 39:**
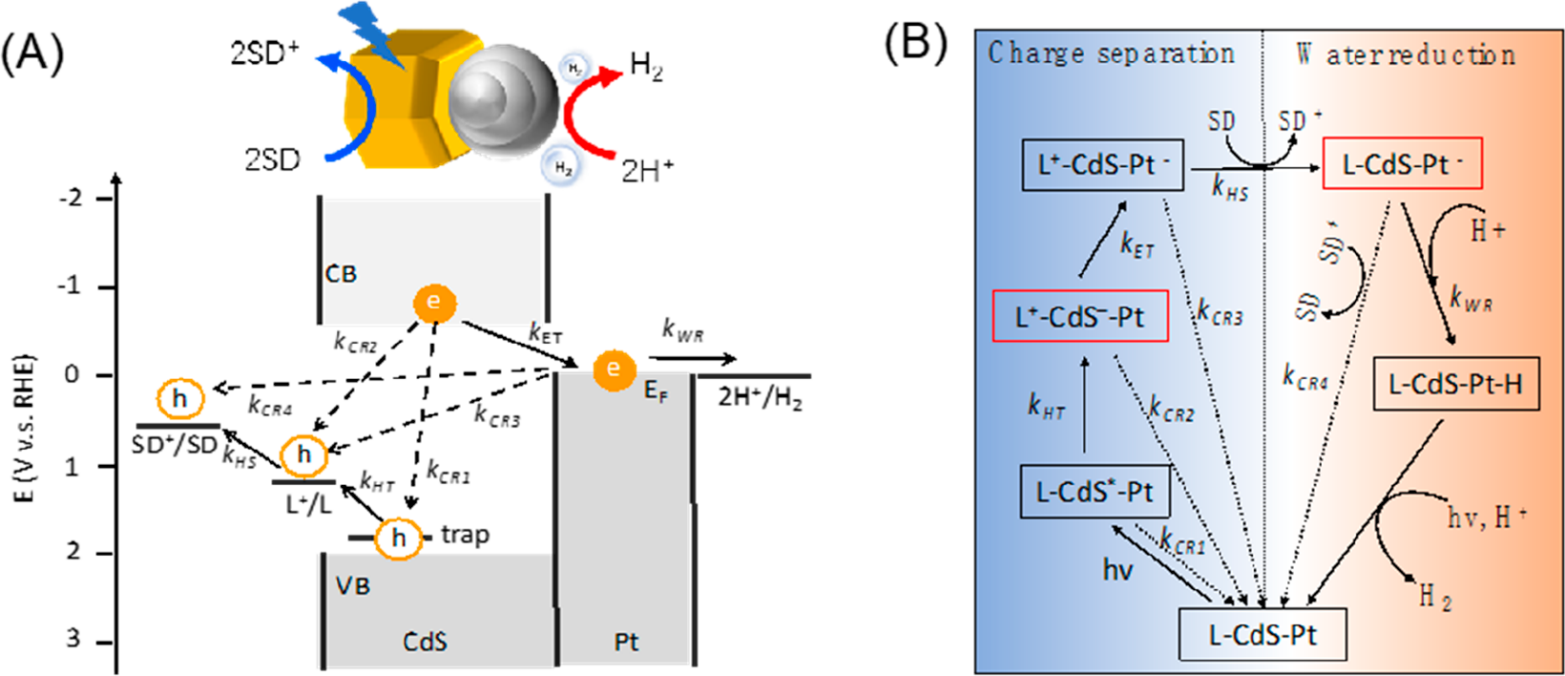
Photodriven H_2_ generation using CdS-Pt NRs. (A) Key energy levels and elementary processes and (B) simplified kinetic model with key intermediate states and processes for light-driven H_2_ generation using L-CdS-Pt nanorods. L indicates the MUA capping ligands on the CdS surface. Forward processes (solid arrows): hole transfer from the trapped exciton state to surface ligand L (with a rate constant *k*_HT_), electron transfer from the CdS to Pt (*k*_ET_), hole transfer from the oxidized surface ligand L^+^ to sacrificial donor SD (*k*_HS_), and water reduction on a reduced Pt particle (*k*_WR_). Each process competes with a charge recombination process (*k*_CR*i*_, *i* = 1–4, dashed arrows), which determines their quantum efficiency. The overall quantum efficiency of light-driven H_2_ generation is the product of the quantum efficiencies of the elementary steps. Also shown in (A) is a schematic of overall light-driven H_2_ generation. (A and B) Adapted with permission from ref (470). Copyright 2022 American Chemical Society.

Based on the works we review in the previous section, electron transfer from quantum-confined NCs to attached catalysts including molecules, enzymes, and metals are fast and the charge separation efficiencies approaches unity in many systems. In contrast, their photocatalytic quantum efficiencies for multielectron reactions such as H_2_ generation are often far from unity.^49,424,453^ The complete light-driven H_2_ generation process involves many forward and backward steps, and the competition of these processes determines the overall quantum efficiency. These competitive processes have been illustrated in recent studies of efficiency-limiting steps in the overall light-driven H_2_ generation process in CdS-Pt NRs using l-cysteine as a the sacrificial electron donor and mercaptoundecanoic acid (MUA) as the NR surface capping ligand and the initial hole acceptor, where the sacrificial donor removes the hole from the NRs and undergoes the oxidation half-reaction.^470,471^ As shown in Figure 39A, starting from an exciton state in the nanorod, the overall light to H_2_ generation using CdS-Pt NRs involves multiple forward electron and hole transfer steps indicated by solid arrows: ET from the excited CdS to Pt, a two-electron/two proton water/proton reduction (WR) process on Pt, hole transfer (HT) from the CdS valence band or trap states to surface ligands (L = 11-MUA), hole transfer from the surface ligand to the sacrificial donor (l-cysteine) in solution, which is referred to as the hole scavenging (HS) step, and finally the oxidation reaction occurring on the hole scavenger or sacrificial donor (not shown). These forward steps compete with a series of backward e-h charge recombination steps (also known as the back electron transfer step), which are indicated by dashed arrows. It was proposed that because of the time separation of many of these electron and hole transfer processes the overall process can be described by a simplified kinetics model shown in Figure 39B, which involves four sequential kinetics stages with their own quantum efficiencies. The excitation of a L-CdS-Pt NR creates an exciton state (L-CdS*-Pt) that undergoes fast trapping of the VB hole on a sub-ps time scale. In the HT stage starting with L-CdS*-Pt, hole transfer to the MUA ligand to form L^+^-CdS^–^-Pt (with a rate constant of *k*_HT_) competes with the electron–hole recombination (*k*_CR1_) within CdS* to determine the hole transfer QE (QE_HT_). In the ET stage, starting with L^+^-CdS^–^-Pt, ET from CdS^–^ to Pt (*k*_ET_*)* to form the charge-separated state (L^+^-CdS-Pt^–^) competes with charge recombination between CdS^–^ and L^+^(*k*_*CR2*_), giving rise to the ET QE (QE_ET_). In the hole scavenging stage, starting with L^+^-CdS-Pt^–^, hole transfer from L^+^ to SD in solution (*k*_HS_) to forms L-CdS-Pt^–^ competes with charge recombination between L^+^ and Pt^–^ (*k*_CR3_) to determine the hole scavenging QE (QE_HS_). The resulting L-CdS-Pt^–^ is a long-lived species that can continue on to the slow water or proton reduction steps with a quantum efficiency of QE_WR_. Although the detailed mechanism of the two-electron/two-proton H_2_ generation process on Pt is complex, it can be assumed that it has a rate limiting step and its competition with charge recombination, likely between Pt^–^ and oxidized SD, controls its QE. Within this simplified sequential kinetics model, the overall QE of the light-driven H_2_ production process can be considered as the product of the four elementary stages: QE_H2_ = QE_HT_ × QE_ET_ × QE_HS_ × QE_WR_. Although the rates of individual steps likely change in different systems and the kinetics models should be modified, we believe that decomposing the overall light-to-H_2_ conversion process into its elementary stages facilitates the determination of key efficiency-limiting factors. In the following, we review several studies of NC-based photocatalytic H_2_ generation systems to illustrate how these steps affect the quantum yield of the overall reactions.

**Figure 40:**
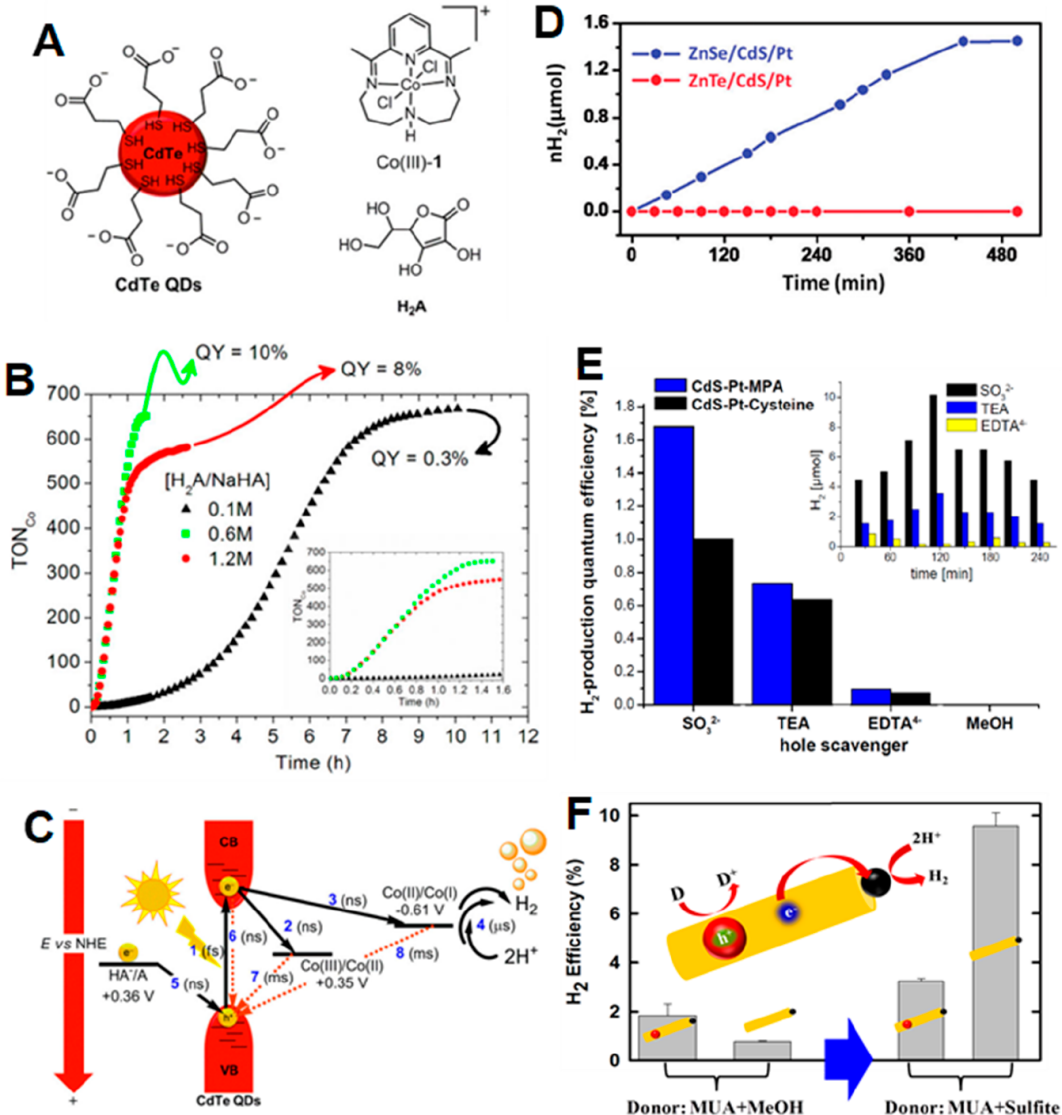
Photocatalytic efficiency-limiting factors for NC-based systems. (A) A photocatalytic H_2_ generation system using CdTe QDs as light-harvesters, Co(III)-**1** as the H_2_ evolution catalyst, and ascorbic acid (H_2_A) as the sacrificial electron donor. (B) H_2_ turnover number per Co catalyst (TON_Co_) as a function of time for the system in (A) with varying concentrations of H_2_A/NaHA donors. The inset shows the long-term performance. (C) Scheme summarizing the energetics and kinetics parameters for the system in (A). The time scales for the various charge separation and recombination processes in the system are labeled. (D) Comparison of H_2_ production traces from MUA-capped ZnSe@CdS-Pt (blue) and ZnTe@CdS-Pt (red) NRs. (E) Comparison of H_2_ generation quantum efficiencies for MPA- and cysteine-capped CdS-Pt NRs using SO_3_^2–^, TEA, EDTA^4–^, and MeOH as sacrificial electron donors. The inset shows hydrogen evolution as a function of time. (F) H_2_ generation quantum efficiencies for both MUA-capped CdSe@CdS-Pt and CdS-Pt NRs in an aqueous solution with SO_3_^2–^ or MeOH as the added sacrificial electron donor. (A–C) Adapted with permission from ref (410). Copyright 2014 American Chemical Society. (D) Adapted with permission from ref (452). Copyright 2011 American Chemical Society. (E) Adapted with permission from ref (472). Copyright 2012 American Institute of Physics. (F) Adapted with permission from ref (424). Copyright 2014 American Chemical Society.

For NC–molecular catalyst systems, Llobet and co-workers investigated all the involved reactions in a model system comprising CdTe QDs, a molecular cobalt catalyst, and ascorbic acid as sacrificial electron donor (Figure 40A); while the former accepts the electrons from QDs for the H_2_ reduction half-reaction, the latter consumes the holes from CdTe QDs for the oxidation half-reaction.^410^ The importance of hole transfer to sacrificial donors is illustrated by the donor-concentration-dependent H_2_ generation behavior (Figure 40B). When the concentration of the donor increases from 0.1 to 0.6 M, the QY for H_2_ generation is enhanced by 33-fold from 0.3% to 10%. A higher donor concentration improves the hole transfer rate for it compete with and suppress charge recombination between catalysts and holes in NCs. When the donor concentration further increases from 0.6 to 1.2 M, the QY slightly drops, likely due to saturation of the hole transfer rate. The authors have also measured all the charge transfer and recombination time constants in the reaction. The results of these time scales are summarized in the scheme in Figure 40C. They find that electron transfer from QDs to catalysts occurs fast enough and efficiently (nanosecond time scale) whereas the charge recombination and catalysis are much slower (microsecond to millisecond time scales). On the basis of these measurements, they conclude that for this system efficiency improvements should focus on the catalytic rate enhancement, which should be at least on the hundreds of ns time scale.

Many research groups including us have studied the efficiency-limiting factors for NC–metal heterostructures. As the electron transfer yields for these structures are generally high, hole removal by sacrificial electron donors is often a limiting step.^424,452,472,473^ Zamkov and co-workers show that the H_2_ generation rate of MUA-capped ZnSe@CdS-Pt NRs is ∼300-fold faster than that of MUA-capped ZnTe@CdS-Pt NRs measured under the same conditions (Figure 40D), where methanol was used as the sacrificial donor for the hole removal from the ZnSe or ZnTe cores inside the NRs.^452^ The difference is attributed to the fact that initial hole transfer from the ZnSe core to the surface hole acceptor ligand (MUA) is energetically allowed, while it is not favored for the ZnTe core, which is confirmed by their time-resolved PL decay measurements. The importance of hole removal is also confirmed by Berr and co-workers in their study of H_2_ generation from CdS-Pt NRs in the presence of different sacrificial donors.^472^ As shown in Figure 40E, various molecules were used as sacrificial donors for the hole removal from the NRs. The highest efficiency for cysteine-capped NRs is achieved using SO_3_^2–^ as the donor (1.7%), and it drops to 0.7% for TEA, 0.1% for EDTA^4–^, and below the detection limit for MeOH. The same trend is observed for MPA-capped NRs but with systematically higher efficiencies. This efficiency trend positively correlates the reducing power (*i.e.*, more negative oxidation potentials) of sacrificial donors: SO_3_^2–^ > TEA > EDTA^4–^ > MeOH. The authors further show that faster hole transfer not only improves H_2_ generation QYs but also is essential for the stability of the photocatalytic systems. TEM images reveal obvious shortenings and aggregations of NRs with MeOH and EDTA^4–^ as the hole acceptors, suggesting that the holes in NRs, if not scavenged fast enough, oxidize and etch their own surfaces.

Our recent study also shows that the photocatalytic efficiency of H_2_ generation from CdS-Pt and CdSe@CdS-Pt NRs depends sensitively on sacrificial electron donors.^424^ In particular, we find that the relative performance between these two types of NRs depends on the choice of sacrificial electron donors for removing the holes from the NRs (Figure 40F). With MeOH as the sacrificial electron donor, we observe higher efficiency for CdSe@CdS-Pt NRs (1.8%) than for CdS-Pt NRs (0.8%). The efficiencies of both NRs are improved with SO_3_^2–^ as the electron donor, but CdS-Pt NRs have an efficiency (9.6%) higher than CdSe@CdS-Pt NRs (3.2%). These generation efficiencies correlate positively with the hole transfer rates measured with time-resolved PL decay.^424^ We suspect that because of their strong coupling with phonons,^474,475^ trapped holes require a large reorganization energy and/or driving force for fast hole transfer.^257^ Therefore, the rates for hole transfer from CdS NRs to weakly reductive MeOH and MUA are slower than that for CdSe@CdS NRs. When using strongly reductive SO_3_^2–^ as the donor, the driving force is not a limiting parameter and hence the trapped holes on CdS NR surfaces are more easily accessed by donors than the core-confined holes in CdSe@CdS NRs.

#### 6.3.2 Approaches to Improving Photocatalytic Efficiency

**Figure 41:**
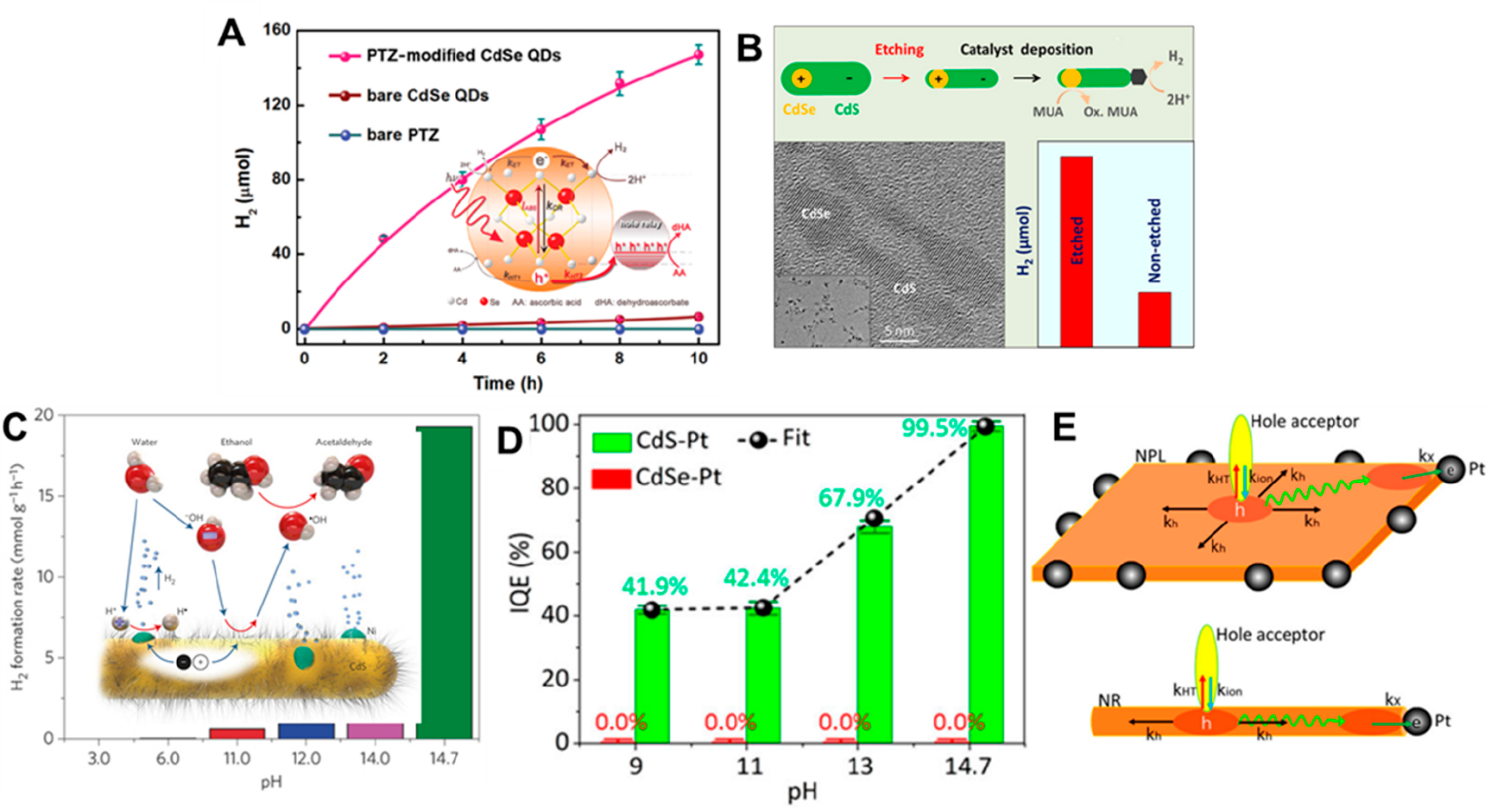
Approaches to suppress recombination and enhance photocatalytic efficiency. (A) Light-driven H_2_ evolution of bare CdSe QDs, PTZ-modified CdSe QDs, and bare PTZ from 10.0 mL of ascorbic acid aqueous solution (0.2 M) at pH = 4.0. (B) CdSe@CdS-Pt NRs with an exposed CdSe core prepared via chemical etching (top). Representative TEM image of etched CdSe@CdS NRs (bottom left). Comparison of the H_2_ generation efficiency of etched and nonetched CdSe@CdS-Pt NRs (bottom right). (C) H_2_ generation rates from cysteine-capped CdS-Ni NRs as a function of pH. The inset shows a scheme of OH^–^ hole-shuttle-mediated photocatalytic generation of H_2_. At high pH, *i.e.*, high hydroxyl anion concentration, the photoexcited holes in CdS NRs oxidize hydroxyl anions to hydroxyl radicals, which carry away the positive charges and in turn oxidize ethanol to acetaldehyde. The electrons are transferred to the Ni nanoparticle and catalyze H_2_ formation. (D) Light-driven H_2_ generation internal quantum efficiency (IQE) for CdS-Pt (green column) and CdSe-Pt NPLs (red column) at varying pH levels. The black dots with dashed line is a fit to the pH-dependent IQE of CdS-Pt NPLs. (E) Scheme of charge recombination processes in 2D NPL-Pt and 1D NR-Pt heterostructures in aqueous solution at pH < 13 when the major electron donors are cysteine molecules. It is assumed that the holes transferred to cysteine (with a rate of *k*_HT_) can be ionized back to the NPLs and NRs (with a rate of *k*_ion_). The holes diffuse along the NPLs and NRs (with a rate of *k*_h_) to the NC–Pt interface, where they recombine with electrons in Pt (with a rate of *k*_*X*_). The 2D morphology can effectively slow down *k*_h_ and thus retard charge recombination. (A) Adapted with permission from ref (476). Copyright 2016 John Wiley & Sons. (B) Adapted with permission from ref (451). Copyright 2013 American Chemical Society. (C) Adapted with permission from ref (473). Copyright 2014 Springer Nature. (D and E) Adapted with permission from ref (447). Copyright 2018 American Chemical Society.

Wu and co-workers reported that modifying CdSe QDs with hole-accepting ligands, such as phenothiazine (PTZ), strongly enhanced the photocatalytic H_2_ generation efficiency of CdSe QDs in aqueous solution and also the photoelectrochemical H_2_ generation efficiency of the CdSe QD-sensitized photocathode.^476^ The choice of the hole-accepting ligand is based on previous extensive studies showing that PTZ is a good acceptor for II–VI group NCs, capable of scavenging holes on the ps to ns time scale. As shown in Figure 41A, the rate of H_2_ evolution from PTZ-modified CdSe QDs is ∼40-fold that of bare CdSe QDs when using ascorbic acid as the sacrificial donor. It is proposed that PTZ ligands extract holes rapidly and efficiently from CdSe QDs and the generated PTZ^+^ radicals react with ascorbic acids to transform the latter into diascorbic acid. As hole transfer from QDs to PTZ is faster than that from QDs directly to ascorbic acid, this shuttling mechanism suppresses charge recombination between electrons on catalytic sites and holes in QDs and thus improves the photocatalytic efficiency. A parallel approach to accelerate hole transfer is to engineer the morphology of NCs. For example, Zamkov and co-workers reported that CdSe@CdS-Pt NRs etched with peroxides display a H_2_ generation rate that is ∼3–4-fold that of unetched ones (Figure 41B). They found that this simple chemical etching procedure led to partial dissolution of the CdS shell surrounding the CdSe core and thus facilitated physical contacts between CdSe core and electron donors for fast hole transfer.^451^

Faster hole transfer to sacrificial donors can suppress charge recombination between electrons on catalytic sites and holes in NCs, but it does not guarantee efficient photocatalysis. Indeed, many previous studies on Pt-tipped NRs, for example, show that the hole transfer to donors (ps to ns time scale) is much faster than the recombination between electrons on Pt and holes in NRs (tens of ns to μs time scale), while the H_2_ generation efficiency of these systems is generally below 10%. This is likely because the chemical transformation of donors used in the reaction (such as alcohol and thiol molecules) is slow such that there is significant efficiency loss to recombination between electrons on Pt and holes in donors. Simon and co-workers recently reported a mechanism to effectively accelerate the oxidation of sacrificial donors and thus improve the H_2_ generation efficiency of Ni decorated CdS NRs.^473^ They found that the H_2_ generation rates increased significantly with pH (Figure 41C). In particular, at pH 14.7, an external QY of 53% (or internal QY up to 71%) has been achieved. They propose that, at high pH, the concentration of hydroxyl anions (OH^–^) is high enough to shift their oxidation potential above the VB and hole-trapping levels of CdS NRs such that OH^–^ anions become hole scavengers. The small size of OH^–^ anions allows for their fast diffusion and facile permeation through the ligand shell to reach holes in CdS NRs for fast hole transfer. This is similar to the shuttling mechanism of PTZ-modified QDs introduced above. However, a more important role of OH^–^ anions is that their oxidized products, hydroxyl radicals (OH^•^), are known to be a very reactive species that are able to oxidize ethanol molecules to acetaldehyde with a high rate. In this way, chemical transformation of ethanol donors is significantly accelerated, leading to a high photocatalytic efficiency. More recently, this strategy was applied by Amirav and co-workers to CdSe@CdS-Pt NRs, and a near perfect internal QY (100%) of light-driven H_2_ generation has been achieved at pH ≥15.^428^

Besides the expected hole removal role, the potential undesired roles of the sacrificial donors also need to be carefully examined. For example, the sacrificial donor-involved oxidation half-reaction itself could generate H_2_, in parallel with the H^+^ or H_2_O reduction half-reaction.^477,478^ For widely used sacrificial donors like methanol, ascorbic acid, S^2–^, and triethanolamine (TEOA), it has been reported that oxidation of methanol and ascorbic acid can produce CO_2_ and H_2_, oxidation of S^2–^ leads to sulfur and H_2_, and oxidation of TEOA generates nitride acid, CO_2_, and H_2_. With TEOA as the sacrificial donor, the oxidation half-reactions can contribute up to 34% of the total H_2_ generated in some cases.^477^ In these cases, in addition to the photoreduction of water or proton, H_2_ was also produced by consuming the holes from the NCs. Moreover, in some cases, sacrificial donor radicals (*e.g.*, alkoxy radicals as the oxidized alcohol) generated by the oxidation process can react with electrons again before they get oxidized and reduce back to their original form.^479^ This process consumes the electrons that are supposed to be used for the H_2_ reduction half-reaction. Therefore, the measurement of the H_2_ generation quantum efficiency in systems using sacrificial donors should carefully account for the contributions of the oxidation of sacrificial donors in addition to the desired photoreduction half-reaction. Control experiments of the effects on the oxidation of sacrificial donors should be provided to fairly determine the true light-driven reduction efficiency of H^+^ or H_2_O.

In addition to facilitating hole transfer to donors and the chemical transformation of oxidized donors, one can improve the photocatalytic efficiency by suppressing charge recombination between electrons on catalysts and holes in NCs and/or holes in donors via NC morphology control. For example, our recent study of light-driven H_2_ generation from CdS-Pt NPLs reveals the key role of the 2D morphology in suppressing charge recombination as compared to the 1D morphology.^447^ We examined the pH-dependent internal QYs of CdS-Pt NPLs and found that they also displayed near-unity QYs at pH 14.7 (Figure 41D). An important difference between CdS-Pt NPLs and the CdS-Pt and CdSe@CdS-Pt NRs introduced above is that NPLs have QYs higher than 40% at pH <13, whereas QYs of NRs typically drop to <20%. We propose that, at pH <13, thiol ligands instead of OH^–^ anions are the dominant hole acceptors. In this case, charge recombination, especially recombination between electrons on Pt and holes in thiols (*k*_CR2_), is the key efficiency-limiting factor. We numerically simulated this recombination process for CdS-Pt NPLs and NRs by assuming that charge recombination proceeds via ionization of the holes in thiol ligands back to NPLs or NRs, followed by diffusion along the hole trapping sites to CdS–Pt interface where recombination takes place (Figure 41E). The simulation shows that charge recombination in 2D NPLs is over five-fold slower than that in 1D NRs when the same rate constants for elementary steps and lateral size (same length for NRs and square NPLs) are used. The simple physical underpinning is that the 2D morphology requires many more random walk steps for the hole to reach the recombination interface than the 1D one.

**Figure 42:**
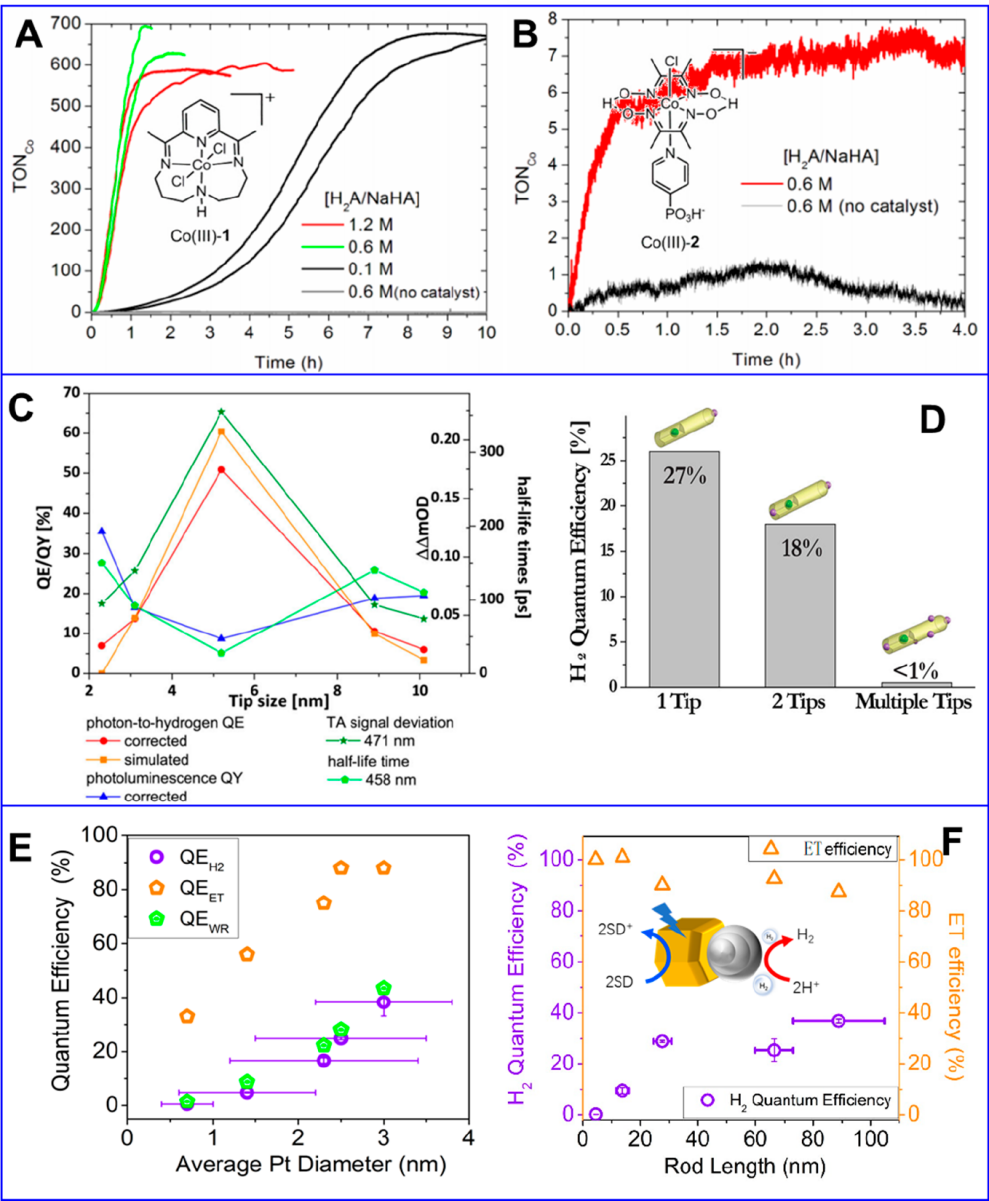
Other factors affecting photocatalytic efficiency. (A and B) H_2_ turnover number per catalyst (TON_Co_) as a function of time for a CdTe QD-based system using (A) Co(III)-**1** and (B) Co(III)-**2** catalysts. Experiments performed using different concentrations of H_2_A/NaHA donors are shown as the indicated colored lines; experiments performed in the absence of catalysts are shown as gray lines. The structures of Co(III)-**1** and Co(III)-**2** are shown in the sets. (C) PL QYs (corrected for the metal absorption contribution) of CdSe@CdS-Ni NRs (blue triangles), TA signal difference at 471 nm between NRs with and with Ni tips (a measure of charge separation efficiency; green stars), corrected and simulated photon to H_2_ efficiencies (red circles and orange squares, respectively), and exciton bleach recovery half-life time (green pentagons) displayed as a function of the metal domain size. The optimal charge separation efficiency and H_2_ generation efficiency are achieved at a Ni tip size of ∼5 nm. (D) H_2_ generation efficiency of CdSe@CdS NRs decorated with a single, double, or multiple Pt nanoparticles. (E) Photodriven H_2_ production internal quantum efficiency QE_H2_ of CdS-Pt NRs as a function of the average Pt tip size. Also shown is the ET quantum efficiency QE_ET_ determined from transient absorption kinetics and the water reduction quantum efficiency C*QE_WR_ calculated by the ratio of QE_H2_ and QE_ET_. (F) Quantum efficiencies of H_2_ production (purple circles) and electron transfer (orange triangles) as a function of the CdS rod length. (A and B) Adapted with permission from ref (410). Copyright 2014 American Chemical Society. (C) Adapted with permission from ref (482). Copyright 2018 American Chemical Society. (D) Adapted with permission from ref (483). Copyright 2015 American Chemical Society. (E) Adapted with permission from ref (470). Copyright 2022 American Chemical Society. (F) Adapted with permission from ref (471). Copyright 2022 American Chemical Society.

The photocatalytic efficiency of NC–catalyst systems can also be improved by optimizing the catalysts, as fast catalytic turnover (large *k*_cat_) favorably outcompetes various charge recombination processes. The importance of catalysts is illustrated in the work by Llobet and co-workers. They compared light-driven H_2_ generation from CdTe QDs coupled with two different cobalt catalysts, Co(III)-**1** and Co(III)-**2**, under the same conditions. Co(III)-**1** and Co(III)-**2** have the same active Co(III) center but different stabilizing ligands. As shown in Figure 42A and B, the H_2_ generation rate of Co(III)-**1** is almost two orders of magnitude faster than that of Co(III)-**2**. This comparison also highlights the essential role of ligand design in enhancing the efficiency of molecular catalysts. The fastest molecular catalysts reported to date are the so-called Dubois-type catalysts with a H_2_ turnover frequency of >10^5^ s^–1^.^480,481^ Mechanistic studies on these catalysts point to a key role of pendant amines on the ligands that function as proton relays for the formation and cleavage of the H–H bond.^480^ This precisely controlled delivery of protons is proposed to be critical in natural hydrogenase enzymes.

For nanoparticle- or nanocluster-based catalysts, the importance of catalyst size is often noticed.^484^ Catalyst size affects the efficiency of a photocatalytic reaction through the size-dependent Coulomb blockade charging energy for charge transfer and/or the size-dependent activity of catalytic sites. For example, Amirav and co-workers studied Ni-tipped CdSe@CdS NRs with different tip sizes for light-driven H_2_ generation (Figure 42C).^482^ The internal QY for H_2_ generation increases from ∼7% for the 2.3 nm tip to ∼14% for the 3.1 nm tip and ∼51% for the optimal 5.2 nm tip size, which is followed by a drop to ∼11% for the 8.9 nm tip and finally to ∼6% for the 10.1 nm tip size. This efficiency trend is found to correlate well with the charge separation efficiency determined from TA measurements (Figure 42C). The authors propose that the Coulomb blockade charging energy and a size-dependent Schottky barrier decreases and increases, respectively, with increasing Ni sizes. These two opposing trends result in an optimal Ni size for highest charge separation yield. Similarly, Banin and co-workers also reported an optimal size for the Au domain for light-driven H_2_ generation from Au-tipped CdS NRs.^485^ In an earlier work, Schweinberger and co-workers examined the effect of Pt cluster size on light-driven H_2_ generation in Pt-decorated CdS NRs.^486^ In their experiment, the size of Pt clusters deposited on CdS NR films can be controlled with an atomic level of precision by using a high-frequency laser ablation technique.^487^ They found that the H_2_ generation rate initially increased with cluster size, peaked at Pt46 (a cluster with 46 Pt atoms), and then decreased with cluster size. They propose that the electron transfer rate from the NR to the cluster and that from the cluster to the proton increases and decreases, respectively, with the cluster size.

More recently, Lian and co-workers investigated the detailed mechanisms of light-driven H_2_ generation in CdS-Pt nanorods through systematic variation of the Pt size and CdS rod length.^470,471^ Using CdS-Pt with the same NR size and length but a different Pt tip size, it was reported that the QE_H2_ of CdS-Pt increases from 0.5 ± 0.2% to 38.3 ± 5.1% by nearly two orders of magnitude when the Pt catalyst size increases from 0.7 ± 0.3 to 3.0 ± 0.8 nm, respectively (Figure 42E). This trend is consistent with the results of Amirav and co-workers (Figure 42C),^482^ and Banin and co-workers,^485^ in the small size regime. Using transient absorption spectroscopy, they directly measure the electron transfer rate as a function of the Pt diameter (Figure 42E). The observed trend was understood by a simplified kinetic model shown in Figure 39 A and B, in which the overall efficiency for light-to-H_2_ generation is assumed to be a product of the quantum efficiencies of sequential hole transfer (QE_ET_), electron transfer (QE_HT_), hole scavenging (QE_HS_), and water reduction (QE_WR_) steps. The quantum efficiencies of both the electron transfer and water reduction steps were shown to increase with the Pt sizes. This work suggests that catalyst size can affect both charge separation and catalysis efficiencies, and both should be considered in designing efficient semiconductor–metal hybrid photocatalysts. This work also reveals that the ET rate from the CdS NR to the Pt tip decreases at smaller Pt diameter following a scaling law of *d*^5.6^, suggesting the challenge in designing nanorod–catalyst hybrid electrodes with small clusters, single-atom catalysts or molecular catalysts. In a related work, Lian and co-workers examine the rod length dependence of light-to-H_2_ generation in CdS-Pt nanorods (Figure 42F).^471^ In addition to the H_2_ generation efficiency, the charge separation and recombination rates were also measured by transient absorption spectroscopy. They observed that QE_H2_ increases from 0.2 ± 0.0% in quantum dots to 27.8 ± 0.4% at 28 nm NRs and changes negligibly at longer rod lengths, and QE_HT_ decreases from 92% at short rod lengths to 70% at long rod lengths (Figure 42F). Analyzing the results using the model shown in Figure 39A and B, Lian and co-workers concluded that the quantum efficiency for hole removal by the sacrificial electron donor (QE_HS_) changes with the rod length and is the key factor for the observed rod-length-dependent QE_H2_.

Yet another important but often overlooked factor influencing photocatalytic performances of multielectron photocatalytic reactions is the number of catalysts immobilized on each NC.^483^ Taking the H_2_ evolution reaction as the example, generating one H_2_ molecule requires sequential absorption of two photons. If there are multiple catalysts on one NC, the first and second photogenerated electrons might be transferred onto two different catalysts. Considering Coulomb repulsion between electrons, this is more likely to take place than the situation of two electrons being transferred to the same catalyst.^488^ As a result, it is difficult to accumulate two electrons on the same catalyst as required for the H_2_ evolution reaction. Indeed, Amirav and co-workers compared light-driven H_2_ generation performances using CdSe@CdS NRs decorated with a different number of Pt sites (Figure 42D) and found that the QY of NRs decorated with one Pt catalyst was 27%, higher than that of NRs having two Pt catalysts (18%) and that of NRs with multiple Pt catalysts (<1%).^483^ Similar trends were observed for Pt-decorated CdSe NRs by Bang and co-workers^489^ and for Pt-decorated CdS NRs by Stolarczyk and co-workers.^490^ Therefore, for many of the multielectron solar-to-chemical conversion reactions, such as O_2_ evolution and CO_2_ reduction,^491−494^ one should control the number of catalysts on each NC to be approximately one for the successful accumulation of multiple redox equivalents on the catalyst. This, in turn, can minimize the amount of precious elements (such as Pt, Ru, Re...) used in photocatalysts for cost-effective solar-fuel generation.

### 6.4 Emerging Photocatalytic Applications Using NCs

**Figure 43:**
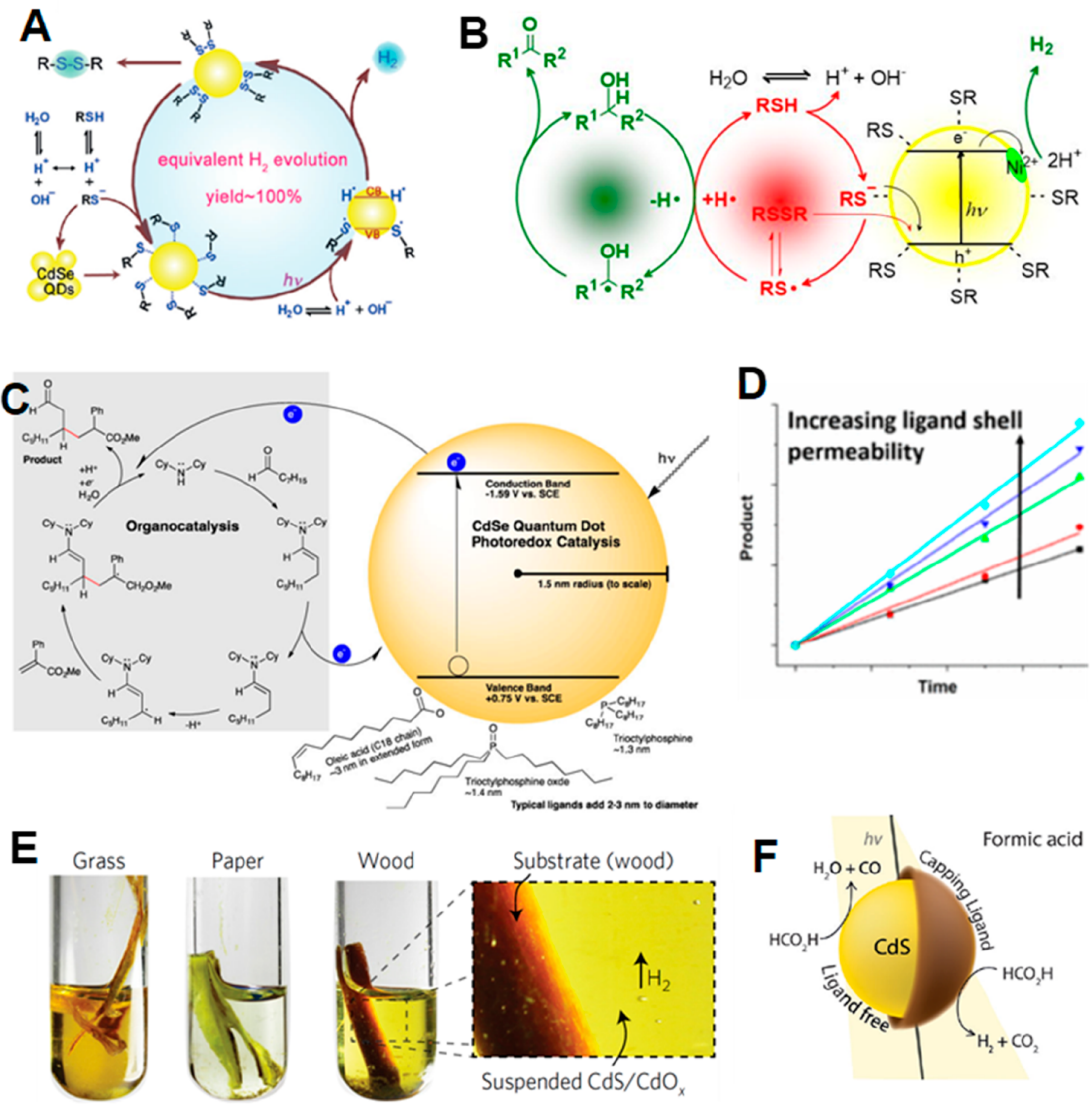
Emerging photocatalytic applications of NCs. (A) Proposed mechanism for the photocatalytic conversion of thiol ligands on QD surfaces to simultaneously generate disulfides and H_2_. (B) The thiyl radicals generated by the photooxidation of thiol ligands on QD surfaces can be intercepted to perform selective oxidation of alcohols into carbonyl compounds. (C) Photoredox synthesis using CdSe QDs. The photogenerated electron and hole in QDs can act as a one-electron reductant and oxidant, respectively, to perform organocatalysis. (D) Amount of C–C coupling product as a function of time for QDs with varying ligand shell permeabilities. (E) CdS QDs coated with CdO_*x*_ shells can perform photoreforming of lignocellulose from various crude sources into H_2_. (F) Selective photocatalytic conversion of formic acid into either H_2_ or CO using CdS QDs, with the product selectivity controlled by the solvent and particle capping ligand. (A) Adapted with permission from ref (497). Copyright 2014 John Wiley & Sons. (B) Adapted with permission from ref (498). Copyright 2017 John Wiley & Sons. (C) Adapted with permission from ref (499). Copyright 2017 American Chemical Society. (D) Adapted with permission from ref (500). Copyright 2017 American Chemical Society. (E) Adapted with permission from ref (501). Copyright 2017 Springer Nature. (F) Adapted with permission from ref (502). Copyright 2015 John Wiley & Sons.

The photocatalytic reactions reviewed above are primarily photoreduction reactions utilizing the photogenerated electrons for product formation. The photogenerated holes are often scavenged by sacrificial donors. The chemical transformation of donors induced by the hole is essentially a photooxidation reaction. Therefore, with appropriate choices of donors, it is possible to simultaneously perform photoreduction and photooxidation half reactions to fully utilize the potential energy stored in both photogenerated electrons and holes in NCs.^495−497^ For example, thiols that are frequently used as sacrificial electron donors for light-driven H_2_ generation can be transformed into disulfides by the holes.^497^ Given the importance of disulfide formation reactions in many systems, Wu and co-workers investigated photodriven disulfide formation using CdSe QDs (Figure 43A).^497^ They found that indeed disulfides formed quantitatively (*i.e.*, with chemical yields approaching 100%) from a variety of thiol molecules and H_2_ was generated with the same near unity chemical yield. The QYs of these reactions were not reported. More recently, Wu and co-workers further demonstrated that the thiyl radicals generated from hole oxidation of thiol ligands on CdSe QDs, before being homocoupled to disulfides, can be intercepted by alcohols to perform selective oxidation of alcohols to aldehydes or ketones (Figure 43B).^498^ These reactions can attain high chemical yields (>90%), high site-selectivity (>90%), and good functional group tolerance, suggesting that photocatalysis using NCs may find important applications in the industrial transformation of alcohols.

Photoredox catalysis, sharing the same principle with common photocatalysis, has emerged as a very important research area in organic synthesis. In these reactions, Ru or Ir complexes are frequently used as photosensitizers. The triplet excited states of these complexes can perform one-electron reduction and oxidation reactions to induce important C–C formation schemes. It is thus a natural idea to replace the organometallic complexes with NCs considering the excellent light-harvesting and charge-donating capabilities of NCs. Krauss and co-workers reported that a single-sized CdSe QD (3.0 nm) was able to photocatalyze five different types of photoredox reactions, including β-alkylation, β-aminoalkylation, dehalogenation, amine arylation, and decarboxylative radical formation.^499^ Without optimizations, the efficiency of QDs is already on par with or superior to that of optimal organometallic catalysts reported in the literature, presumably due to the aforementioned advantages of QDs in terms of light-harvesting and charge-donating. In addition, the QD catalysts are effective at extremely low loadings that are 17 orders of magnitude lower than organometallic catalysts. While this work demonstrates the performance and scope of QDs in photoredox catalysis, the work reported almost at the same time by Weiss and co-workers provides mechanistic insights into photoredox catalysis using QDs.^500^ They studied the reaction of C–C coupling between 1-phenylpyrrolidine (PhPyr) and phenyl *trans*-styryl sulfone driven by photoexcited CdS QDs and found that photooxidation of PhPyr by QDs was the rate-limiting step, consistent with the more sluggish hole transfer compared to electron transfer typical for NCs. The rate of the reaction can be improved by ∼2.3-fold by using QDs with mixed oleate and octylphosphonate ligands, as it increases the ligand permeability and thus facilitates interfacial charge transfer between QDs and substrate molecules (Figure 43D). Photoredox catalysis using engineered core/shell QDs has been reported by the König group^503^ and Peng group.^504^

Photocatalysis using NCs also find broad applications in photoreforming substrates into high-added-value products. Reisner and co-workers reported light-driven photoreforming of lignocellulose (including cellulose, hemicellulose and lignin) to H_2_ using CdS QDs (Figure 43E).^501^ As lignocellulose is the most abundant form of biomass on earth, its photoreforming into H_2_ may provide a cost-effective approach to large-scale clean production of H_2_. The reaction takes place in basic aqueous solutions where CdS QDs form CdS/CdO_*x*_ core/shells *in situ*, which enables the long-term stability of the system. The external QY of the reaction under 430 nm monochromatic light is ∼1.2%, while the QY for photoreforming MeOH reaches 17.7% using the same system, suggesting that photooxidation of the polysaccharide chains is the rate-limiting step. In addition, the system was shown to even be able to reform unprocessed lignocellulose, such as wood and paper. The same group also reported selective photoreforming of formic acid, which is considered as a promising energy carrier and hydrogen storage material, into either H_2_ or CO using photoexcited CdS QDs.^502^ Using MPA-capped CdS QDs in combination with a cobalt cocatalyst, the system releases H_2_ with >99% selectivity and the external QY under 460 nm monochromatic light reaches ∼21.2%. Using ligand-free charge-stabilized CdS QDs (QD-BF4), the same system releases CO with >95% selectivity and a 19.7% external QY.

NC crystals have been used for many more photocatalytic reactions than we can list here. To name some of them, Garcia-Garibay and co-workers reported the reduction of aromatic azides to amines using CdS and CdSe QDs back in 2004,^505^ Weiss and co-workers reported reduction of nitrobenzene to aniline using CdS QDs,^506^ Ford and co-workers reported the oxidation of 1,1-dithiooxalate to carbon disulfide using CdSe QDs,^496^ Pandey and co-workers reported the aerobic oxidation of boronic acids using CdSe QDs,^507^ and Egap and co-workers reported controlled light-mediated radical polymerization using CdSe QDs.^508^ These reactions involve very different chemicals and substrates, but the physical underpinnings are similar. In general, fast and efficient charge separation and suppressed charge recombination are desired for these reactions. As such, further improvements in their QYs should be achievable by using engineered 0D, 1D and 2D NCs and related heterostructures to facilitate charge separation and suppress recombination losses in these reactions.

While it is essential to suppress the recombination of charge-separated states for the photoredox reactions discussed above, in recent years it has been discovered that charge recombination in QD–molecule complexes can actually generate the triplet excited states of the molecules as long as the energies of the charge-separated states are higher than those of the molecular triplets.^509−523^ Thus, sequential charge transfer in QD–molecule complexes opens a pathway to sensitizing the triplet states of the surface-anchored molecules. It turns out that these molecular triplets are also important species involved in many organic photochemical reactions, such as isomerization and [2 + 2] cycloaddition.^524−528^ Therefore, QD–molecule complexes can find a broad scope of applications well beyond photoredox catalysis. In principle, they can be adopted in any demonstrated organic reactions driven by charge transfer or energy transfer using Ru- or Ir-based metal–organic complexes.

## 7 Summary and Outlook

In this Review, we review the progress in understanding charge transfer processes from quantum-confined nanocrystals in model NC–acceptor complexes and in photocatalytic systems. We first introduce the key size-dependent electronic and optical properties of 0D, 1D, and 2D nanocrystals according to the theoretical model of the multiband effective mass approximation. The ability to tune the energetics of conduction band electrons and valence band holes in quantum-confined NCs through their size, morphology, and composition has led to extensive effort in developing nano-heterostructures with desired spatial distributions of electrons and holes and significantly improved properties compared to single-component nanocrystals. We discuss representative time-resolved spectroscopic studies of charge transport and transfer dynamics within these nano-heterostructures.

Electron and hole transfer from quantum-confined nanocrystals and their heterostructures to acceptors have been extensively studied. Because of the strong e-h Coulomb interaction and relatively small energy spacing of the conduction and valence band levels, both electron and hole transfer from the excitonic states of quantum dots were found to follow the Auger-assisted electron transfer model. According to this model, for electron (hole) transfer with a large driving force, the excess energy can be used to excite the hole (electron) to higher energy levels, thus creating a manifold of product states and avoiding the unfavorable Franck–Condon overlap in the Marcus inverted regime. Although the Auger-assisted charge transfer model was only confirmed experimentally for ET and HT from quantum dots, it should be a general model for charge transfer from exciton states of many 0D, 1D, and 2D quantum-confined nanocrystals. Similar to molecular donor–acceptor systems, the ET coupling strength can be conveniently controlled by adding insulating spacers between the NCs and acceptors. A unique way to turn the electronic coupling strength from quantum-confined nanocrystals is to turn their size, which affects the amplitude of wave functions outside the crystal. Unlike molecular chromophores, carriers in nanocrystals can be trapped and can be transferred by a trap-mediated pathway. Most of the ET and HT processes reported so far have been described in the nonadiabatic charge transfer limit. In the presence of strong coupling between the NC and acceptor, ET can occur in the adiabatic limit, especially for strongly quantum-confined nanocrystals. In this limit, NCs show significantly broadened exciton bands due to strong coupling with the acceptor. When ET or HT rates become faster than hot carrier cooling rates within the nanocrystals, hot carriers can be extracted by charge transfer to acceptors. For charge transfer from 1D and 2D nanocrystals, the internal transport of excitons and carriers also plays a role in determining the charge transfer kinetics to acceptors.

The phenomenon of multiple exciton generation has been observed in nanocrystals of various semiconducting materials and has attracted intense research interest because it could be potentially utilized to boost the power conversion efficiency of NC-based photovoltaic devices. In contrast to single-exciton states, the multiple exciton states decay via the Auger process, a significantly faster decay channel, leading to drastically different exciton recombination kinetics. With an assumption of a Poissonian photon absorption in QD ensembles, the number of excitons per dot and their decay pattern can be quantitatively described, and the MEG yield can then be quantified based on this method. For isolated QD–molecular electron acceptor complexes, the multiple exciton states, created either from multiple photon absorption or MEG, can be completely dissociated through ultrafast electron transfer from the QD to the molecule. It has also been demonstrated that the photocurrent of photovoltaic and photoelectrochemical devices based on QD films can also be enhanced by MEG.

Photocatalysis using hybrid nanocrystal/catalysts has been successfully demonstrated, often focusing on the reduction half-reaction with the help of sacrificial electron donor to remove holes. We reviewed representative examples of three major types of NC systems for photocatalysis: NC–molecular catalyst (or enzyme) complexes, NC–mediator–catalyst complexes, and all-inorganic NC–metal particle heterostructures. The mechanisms of light-driven initial charge transfer from NCs to catalysts have been examined by time-resolved spectroscopy in these systems, often monitoring the distinct spectral signature of CB electrons in these NCs and/or PL quenching. While the initial charge transfer can be efficient in some systems, this process alone is not sufficient to guarantee efficient photocatalysis. The overall light-to-H_2_ conversion efficiency depends on many forward and backward processes, and detailed mechanistic studies provide helpful guidance for improving the quantum efficiency. Key progresses in such mechanistic studies are also discussed in this Review. Finally, we discuss emerging applications of NCs in photoredox chemistry.

Through the extensive studies in the last 40 years, the field has made significant advances in understanding charge separation and recombination in quantum-confined nanocrystal–acceptor complexes and demonstrated their applications in photocatalysis. There are many opportunities for making further advances in the field. Compared to the light-driven reduction half reactions using quantum-confined NCs, the oxidation half-reactions of the photogenerated holes are less understood. Sacrificial hole acceptors (or electron donors) are often used to rapidly remove the photogenerated holes to suppress electron hole recombination and to improve the efficiency of slow photoreduction reactions. However, this also inevitably introduces the oxidized complexes/radicals and side reactions of the sacrificial donors in the system. For example, the oxidized sacrificial donor can participate in the reduction process and consume the electrons that are supposed to be used for H_2_ generation, thus reducing the efficiency. Furthermore, the stability and activity of the sacrificial donors directly determine the oxidation half-reaction and thus the long-term stability and the overall efficiency of the photocatalytic system.

There are opportunities to utilize both the photogenerated electrons and holes for alternative desired photocatalytic reactions. This will require further advances in nano-heterostructure or system design that can produce much longer-lived charge-separated states. NC-based photocatalysis of organic transformations is an emerging field that is attracting intense recent interest.^529−533^ These reactions are often conducted in organic solvents, which are more benign toward NCs than aqueous environments. Moreover, such reactions are targeted for high-added-value small molecules and therefore long-term stability might not be a necessity for a practical system. The combination of material composition and size tunability can in principle afford NC-based photosensitizers with desired reduction, oxidation, or sensitization capabilities required for many existing photodriven organic transformations. More specifically, they can be developed as viable replacements for the commonly used noble metal Ru- or Ir-based metal–organic complexes. Although the current mainstream NCs are based on Cd or Pb elements, which might eventually limit their widespread adoption as photosensitizers, NCs free of these elements (such as ZnSe,^528^ CuInSe_2_^534^) are also being developed for efficient organic transformations.

So far, most studies focus on the performance of model systems and insights of detailed mechanisms, and there are no known reports of long-term stability tests and large-scale applications. The former is limited by the long-term stability of chemical components (*e.g.*, QDs, sacrificial donors, molecular catalysts/enzymes...) involved in the photocatalytic systems. The latter brings more fundamental and technological challenges, including but not limited to materials production and photon collection on a large scale, transfer of charge carriers in a macroscopic QD-based system (*e.g.*, QD films), and the diffusion of chemical components and reaction efficiency in a large-scaled solution. Significant progress has been made in the field of photocatalysis using microcrystals, as either a single entity or in a Z scheme, for unassisted photodriven complete water splitting (*i.e.*, both water oxidation and proton reduction),^71−73^ which may offer interesting design rules that are applicable to nanocrystal photocatalysis. In addition to band offsets, it would be interesting to explore the possibility to create build-in fields to drive charge separation in nano-heterostructures, similar to those in microcrystals and bulk semiconductors. There have been successful demonstrations of combining redox mediators with nanocrystals to drive light-driven H_2_ generation, and further investigation of this approach will likely yield promising improvements, learning from the lessons of natural photosynthetic systems and the microcrystal photocatalysis. Impressive quantum efficiencies have been reported in NC-based systems for the light-driven H_2_ evolution half-reaction, but light-driven CO_2_ reduction is still relatively inefficient and is an important area for further research. In Pt tipped nanorods, the use of nano-heterostructures with designed electron and hole spatial distribution has led to the improvement of light-driven H_2_ generation. It would be potentially beneficial to explore similar approaches in a nanocrystal/molecular catalyst hybrid if strategies for selectively binding molecular catalysts to a specific location of the heterostructure can be developed.

Another potentially promising direction is to use NC solid films instead of colloidal solutions. These films can be used as photoelectrodes for either reduction or oxidation reactions without the need for sacrificial agents. While these films have been used extensive in solar cells, they remain under-explored in photocatalysis or photoelectrochemistry. The fabrication of the films inevitably brings complexity or challenges, such as the stacking order, the ligand on the NCs, the uniformity of size and the energy levels, and the optimized NC spacing in the solids for efficient charge and energy transport. There have been reports of using nanocrystal assembly to improve light harvesting, but further research would be needed to fully exploit the power of this approach, drawing on lessons of natural light harvesting antenna systems. Moreover, the toxicity of the widely used NCs, such as chalcogenides and perovskites involving heavy metals (*e.g.*, Cd, Pb...), is still a challenge for their industrial use. Therefore, the replacement of heavy metals and the synthesis of new NCs with similar or better optical and photocatalytic properties are still necessary.

In summary, with the advances in the synthetic and assembly methodologies, the understanding of charge separation and recombination properties, and the development of design rules, we are hopeful that more advanced nanocrystal-based photocatalytic systems will be developed in the near future.

## References

[ref1] Ekimov A. I.; Onushchenko A. A. Quantum Size Effect in Three-Dimensional Microscopic Semiconductor Crystals. Jetp Lett. 1981, 34, 345–348.

[ref2] Brus L. E. A simple model for the ionization potential, electron affinity, and aqueous redox potentials of small semiconductor crystallites. J. Chem. Phys. 1983, 79, 5566–5571. 10.1063/1.445676.

[ref3] Rossetti R.; Nakahara S.; Brus L. E. Quantum size effects in the redox potentials, resonance Raman spectra, and electronic spectra of CdS crystallites in aqueous solution. J. Chem. Phys. 1983, 79, 1086–1088. 10.1063/1.445834.

[ref4] Brus L. E. Electron--electron and electron-hole interactions in small semiconductor crystallites: The size dependence of the lowest excited electronic state. J. Chem. Phys. 1984, 80, 4403–4409. 10.1063/1.447218.

[ref5] Burda C.; Chen X.; Narayanan R.; El-Sayed M. A. Chemistry and Properties of Nanocrystals of Different Shapes. Chem. Rev. 2005, 105, 1025–1102. 10.1021/cr030063a. 15826010

[ref6] Henry C. Size Effects on Structure and Morphology of Free or Supported Nanoparticles. In Nanomaterials and Nanochemistry, Bréchignac C., Houdy P., Lahmani M., Eds.; Springer, 2007; pp 3–34.

[ref7] El-Sayed M. A. Small Is Different: Shape-, Size-, and Composition-Dependent Properties of Some Colloidal Semiconductor Nanocrystals. Acc. Chem. Res. 2004, 37, 326–333. 10.1021/ar020204f. 15147173

[ref8] Murray C. B.; Norris D. J.; Bawendi M. G. Synthesis and characterization of nearly monodisperse CdE (E = sulfur, selenium, tellurium) semiconductor nanocrystallites. J. Am. Chem. Soc. 1993, 115, 8706–8715. 10.1021/ja00072a025.

[ref9] Yu W. W.; Qu L.; Guo W.; Peng X. Experimental Determination of the Extinction Coefficient of CdTe, CdSe, and CdS Nanocrystals. Chem. Mater. 2003, 15, 2854–2860. 10.1021/cm034081k.

[ref10] Li J.; Chen J.; Shen Y.; Peng X. Extinction coefficient per CdE (E = Se or S) unit for zinc-blende CdE nanocrystals. Nano Res. 2018, 11, 3991–4004. 10.1007/s12274-018-1981-4.

[ref11] Hens Z.; Moreels I. Light absorption by colloidal semiconductor quantum dots. J. Mater. Chem. 2012, 22, 10406–10415. 10.1039/c2jm30760j.

[ref12] Yeltik A.; Delikanli S.; Olutas M.; Kelestemur Y.; Guzelturk B.; Demir H. V. Experimental Determination of the Absorption Cross-Section and Molar Extinction Coefficient of Colloidal CdSe Nanoplatelets. J. Phys. Chem. C 2015, 119, 26768–26775. 10.1021/acs.jpcc.5b09275.

[ref13] Achtstein A. W.; Antanovich A.; Prudnikau A.; Scott R.; Woggon U.; Artemyev M. Linear Absorption in CdSe Nanoplates: Thickness and Lateral Size Dependency of the Intrinsic Absorption. J. Phys. Chem. C 2015, 119, 20156–20161. 10.1021/acs.jpcc.5b06208.

[ref14] Zhu H. M.; Lian T. Q. Wavefunction engineering in quantum confined semiconductor nanoheterostructures for efficient charge separation and solar energy conversion. Energ Environ. Sci. 2012, 5, 9406–9418. 10.1039/c2ee22679k.

[ref15] Nozik A. J. Quantum dot solar cells. Physica E: Low-dimensional Systems and Nanostructures 2002, 14, 115–120. 10.1016/S1386-9477(02)00374-0.

[ref16] Kamat P. V.; Tvrdy K.; Baker D. R.; Radich J. G. Beyond Photovoltaics: Semiconductor Nanoarchitectures for Liquid-Junction Solar Cells. Chem. Rev. 2010, 110, 6664–6688. 10.1021/cr100243p. 20973544

[ref17] Nozik A. J.; Beard M. C.; Luther J. M.; Law M.; Ellingson R. J.; Johnson J. C. Semiconductor Quantum Dots and Quantum Dot Arrays and Applications of Multiple Exciton Generation to Third-Generation Photovoltaic Solar Cells. Chem. Rev. 2010, 110, 6873–6890. 10.1021/cr900289f. 20945911

[ref18] Kramer I. J.; Sargent E. H. The Architecture of Colloidal Quantum Dot Solar Cells: Materials to Devices. Chem. Rev. 2014, 114, 863–882. 10.1021/cr400299t. 24053639

[ref19] Carey G. H.; Abdelhady A. L.; Ning Z.; Thon S. M.; Bakr O. M.; Sargent E. H. Colloidal Quantum Dot Solar Cells. Chem. Rev. 2015, 115, 12732–12763. 10.1021/acs.chemrev.5b00063. 26106908

[ref20] Wu K.; Lian T. Quantum confined colloidal nanorod heterostructures for solar-to-fuel conversion. Chem. Soc. Rev. 2016, 45, 3781–3810. 10.1039/C5CS00472A. 27043714

[ref21] Lu H.; Huang Z.; Martinez M. S.; Johnson J. C.; Luther J. M.; Beard M. C. Transforming energy using quantum dots. Energ Environ. Sci. 2020, 13, 1347–1376. 10.1039/C9EE03930A.

[ref22] Semonin O. E.; Luther J. M.; Choi S.; Chen H.-Y.; Gao J.; Nozik A. J.; Beard M. C. Peak External Photocurrent Quantum Efficiency Exceeding 100% via MEG in a Quantum Dot Solar Cell. Science 2011, 334, 1530–1533. 10.1126/science.1209845. 22174246

[ref23] Yan Y.; Crisp R. W.; Gu J.; Chernomordik B. D.; Pach G. F.; Marshall A. R.; Turner J. A.; Beard M. C. Multiple exciton generation for photoelectrochemical hydrogen evolution reactions with quantum yields exceeding 100%. Nat. Energy 2017, 2, 17052 10.1038/nenergy.2017.52.

[ref24] Gratzel M. Photoelectrochemical cells. Nature 2001, 414, 338–344. 10.1038/35104607. 11713540

[ref25] Rossetti R.; Beck S. M.; Brus L. E. Direct observation of charge-transfer reactions across semiconductor: aqueous solution interfaces using transient Raman spectroscopy. J. Am. Chem. Soc. 1984, 106, 980–984. 10.1021/ja00316a027.

[ref26] Rossetti R.; Brus L. E. Picosecond resonance Raman scattering study of methylviologen reduction on the surface of photoexcited colloidal cadmium sulfide crystallites. J. Phys. Chem. 1986, 90, 558–560. 10.1021/j100276a014.

[ref27] Kamat P. V.; Dimitrijevic N. M.; Fessenden R. W. Photoelectrochemistry in particulate systems. 6. Electron-transfer reactions of small cadmium sulfide colloids in acetonitrile. J. Phys. Chem. 1987, 91, 396–401. 10.1021/j100286a029.

[ref28] Bawendi M. G.; Steigerwald M. L.; Brus L. E. The Quantum Mechanics of Larger Semiconductor Clusters (″Quantum Dots″). Annu. Rev. Phys. Chem. 1990, 41, 477–496. 10.1146/annurev.pc.41.100190.002401.

[ref29] Efros A. L.; Rosen M.; Kuno M.; Nirmal M.; Norris D. J.; Bawendi M. Band-edge exciton in quantum dots of semiconductors with a degenerate valence band: Dark and bright exciton states. Phys. Rev. B 1996, 54, 4843–4856. 10.1103/PhysRevB.54.4843. 9986445

[ref30] Klimov V. I.; Mikhailovsky A. A.; McBranch D. W.; Leatherdale C. A.; Bawendi M. G. Quantization of multiparticle Auger rates in semiconductor quantum dots. Science 2000, 287, 1011–1013. 10.1126/science.287.5455.1011. 10669406

[ref31] Zhang J. Z. Ultrafast Studies of Electron Dynamics in Semiconductor and Metal Colloidal Nanoparticles: Effects of Size and Surface. Acc. Chem. Res. 1997, 30, 423–429. 10.1021/ar960178j.

[ref32] Little R. B.; Burda C.; Link S.; Logunov S.; El-Sayed M. A. Charge Separation Effects on the Rate of Nonradiative Relaxation Processes in Quantum Dots–Quantum Well Heteronanostructures. J. Phys. Chem. A 1998, 102, 6581–6584. 10.1021/jp9822687.

[ref33] Zhang J. Z. Interfacial Charge Carrier Dynamics of Colloidal Semiconductor Nanoparticles. J. Phys. Chem. B 2000, 104, 7239–7253. 10.1021/jp000594s.

[ref34] Logunov S.; Green T.; Marguet S.; El-Sayed M. A. Interfacial Carriers Dynamics of CdS Nanoparticles. J. Phys. Chem. A 1998, 102, 5652–5658. 10.1021/jp980387g.

[ref35] Evans J. E.; Springer K. W.; Zhang J. Z. Femtosecond studies of interparticle electron transfer in a coupled CdS–TiO2 colloidal system. J. Chem. Phys. 1994, 101, 6222–6225. 10.1063/1.468376.

[ref36] Sant P. A.; Kamat P. V. Interparticle electron transfer between size-quantized CdS and TiO2 semiconductor nanoclusters. Phys. Chem. Chem. Phys. 2002, 4, 198–203. 10.1039/b107544f.

[ref37] Robel I.; Kuno M.; Kamat P. V. Size-Dependent Electron Injection from Excited CdSe Quantum Dots into TiO2 Nanoparticles. J. Am. Chem. Soc. 2007, 129, 4136–4137. 10.1021/ja070099a. 17373799

[ref38] Robel I.; Subramanian V.; Kuno M.; Kamat P. V. Quantum Dot Solar Cells. Harvesting Light Energy with CdSe Nanocrystals Molecularly Linked to Mesoscopic TiO2 Films. J. Am. Chem. Soc. 2006, 128, 2385–2393. 10.1021/ja056494n. 16478194

[ref39] Kamat P. V. Quantum Dot Solar Cells. Semiconductor Nanocrystals as Light Harvesters. J. Phys. Chem. C 2008, 112, 18737–18753. 10.1021/jp806791s.

[ref40] Kongkanand A.; Tvrdy K.; Takechi K.; Kuno M.; Kamat P. V. Quantum Dot Solar Cells. Tuning Photoresponse through Size and Shape Control of CdSe–TiO2 Architecture. J. Am. Chem. Soc. 2008, 130, 4007–4015. 10.1021/ja0782706. 18311974

[ref41] Blackburn J. L.; Ellingson R. J.; Mićić O. I.; Nozik A. J. Electron Relaxation in Colloidal InP Quantum Dots with Photogenerated Excitons or Chemically Injected Electrons. J. Phys. Chem. B 2003, 107, 102–109. 10.1021/jp026746w.

[ref42] Blackburn J. L.; Selmarten D. C.; Ellingson R. J.; Jones M.; Micic O.; Nozik A. J. Electron and Hole Transfer from Indium Phosphide Quantum Dots. J. Phys. Chem. B 2005, 109, 2625–2631. 10.1021/jp046781y. 16851267

[ref43] Dimitrijević N. M.; Rajh T.; Ahrenkiel S. P.; Nedeljković J. M.; Mićić O. I.; Nozik A. J. Charge Separation in Heterostructures of InP Nanocrystals with Metal Particles. J. Phys. Chem. B 2005, 109, 18243–18249. 10.1021/jp051201y. 16853347

[ref44] Sykora M.; Petruska M. A.; Alstrum-Acevedo J.; Bezel I.; Meyer T. J.; Klimov V. I. Photoinduced Charge Transfer between CdSe Nanocrystal Quantum Dots and Ru–Polypyridine Complexes. J. Am. Chem. Soc. 2006, 128, 9984–9985. 10.1021/ja061556a. 16881606

[ref45] Boulesbaa A.; Issac A.; Stockwell D.; Huang Z.; Huang J.; Guo J.; Lian T. Ultrafast Charge Separation at CdS Quantum Dot/Rhodamine B Molecule Interface. J. Am. Chem, Soc. 2007, 129, 15132–15133. 10.1021/ja0773406. 18001027

[ref46] Huang J.; Huang Z.; Jin S.; Lian T. Exciton Dissociation in CdSe Quantum Dots by Hole Transfer to Phenothiazine. J. Phys. Chem. C 2008, 112, 19734–19738. 10.1021/jp808291u.

[ref47] Huang J.; Stockwell D.; Huang Z.; Mohler D. L.; Lian T. Photoinduced Ultrafast Electron Transfer from CdSe Quantum Dots to Re-bipyridyl Complexes. J. Am. Chem. Soc. 2008, 130, 5632–5633. 10.1021/ja8003683. 18393497

[ref48] Issac A.; Jin S.; Lian T. Intermittent Electron Transfer Activity from Single CdSe/ZnS QDs. J. Am. Chem. Soc. 2008, 130, 11280–11281. 10.1021/ja8043085. 18680292

[ref49] Amirav L.; Alivisatos A. P. Photocatalytic Hydrogen Production with Tunable Nanorod Heterostructures. J. Phys. Chem. Lett. 2010, 1, 1051–1054. 10.1021/jz100075c.

[ref50] Kamat P. V. Photochemistry on nonreactive and reactive (semiconductor) surfaces. Chem. Rev. 1993, 93, 267–300. 10.1021/cr00017a013.

[ref51] Hagfeldt A.; Graetzel M. Light-Induced Redox Reactions in Nanocrystalline Systems. Chem. Rev. 1995, 95, 49–68. 10.1021/cr00033a003.

[ref52] Adams D. M.; Brus L.; Chidsey C. E. D.; Creager S.; Creutz C.; Kagan C. R.; Kamat P. V.; Lieberman M.; Lindsay S.; Marcus R. A.; et al. Charge Transfer on the Nanoscale: Current Status. J. Phys. Chem. B 2003, 107, 6668–6697. 10.1021/jp0268462.

[ref53] Ponseca C. S.; Chábera P.; Uhlig J.; Persson P.; Sundström V. Ultrafast Electron Dynamics in Solar Energy Conversion. Chem. Rev. 2017, 117, 10940–11024. 10.1021/acs.chemrev.6b00807. 28805062

[ref54] Zhang Y. H.; Wu G. H.; Liu F.; Ding C.; Zou Z. G.; Shen Q. Photoexcited carrier dynamics in colloidal quantum dot solar cells: insights into individual quantum dots, quantum dot solid films and devices. Chem. Soc. Rev. 2020, 49, 49–84. 10.1039/C9CS00560A. 31825404

[ref55] Harris R. D.; Bettis Homan S.; Kodaimati M.; He C.; Nepomnyashchii A. B.; Swenson N. K.; Lian S.; Calzada R.; Weiss E. A. Electronic Processes within Quantum Dot-Molecule Complexes. Chem. Rev. 2016, 116, 12865–12919. 10.1021/acs.chemrev.6b00102. 27499491

[ref56] Tkachenko N. V. Photoinduced Charge Separation in Semiconductor-Quantum-Dot/Organic-Molecule Hybrids. ChemPhotoChem. 2018, 2, 112–120. 10.1002/cptc.201700161.

[ref57] Zhu H.; Yang Y.; Wu K.; Lian T. Charge Transfer Dynamics from Photoexcited Semiconductor Quantum Dots. Annu. Rev. Phys. Chem. 2016, 67, 259–281. 10.1146/annurev-physchem-040215-112128. 27215815

[ref58] Utterback J. K.; Ruzicka J. L.; Keller H. R.; Pellows L. M.; Dukovic G. Electron Transfer from Semiconductor Nanocrystals to Redox Enzymes. Annu. Rev. Phys. Chem. 2020, 71, 335–359. 10.1146/annurev-physchem-050317-014232. 32074472

[ref59] Asaithambi A.; Kazemi Tofighi N.; Ghini M.; Curreli N.; Schuck P. J.; Kriegel I. Energy transfer and charge transfer between semiconducting nanocrystals and transition metal dichalcogenide monolayers. Chem. Commun. (Camb) 2023, 59, 7717–7730. 10.1039/D3CC01125A. 37199319 PMC10281493

[ref60] Li X.-B.; Tung C.-H.; Wu L.-Z. Semiconducting quantum dots for artificial photosynthesis. Nature Reviews Chemistry 2018, 2, 160–173. 10.1038/s41570-018-0024-8.

[ref61] Hu Q.; Yu X.; Gong S.; Chen X. Nanomaterial catalysts for organic photoredox catalysis-mechanistic perspective. Nanoscale 2021, 13, 18044–18053. 10.1039/D1NR05474K. 34718365

[ref62] Wu K.; Lian T. Quantum confined colloidal nanorod heterostructures for solar-to-fuel conversion. Chem. Soc. Rev. 2016, 45, 3781–3810. 10.1039/C5CS00472A. 27043714

[ref63] Wu K.; Zhu H.; Lian T. Ultrafast Exciton Dynamics and Light-Driven H2 Evolution in Colloidal Semiconductor Nanorods and Pt-Tipped Nanorods. Acc. Chem. Res. 2015, 48, 851–859. 10.1021/ar500398g. 25682713

[ref64] Li Q. Y.; Lian T. Q. Exciton dissociation dynamics and light-driven H-2 generation in colloidal 2D cadmium chalcogenide nanoplatelet heterostructures. Nano Research 2018, 11, 3031–3049. 10.1007/s12274-018-2024-x.

[ref65] Han Y.; He S.; Wu K. Molecular Triplet Sensitization and Photon Upconversion Using Colloidal Semiconductor Nanocrystals. ACS Energy Letters 2021, 6, 3151–3166. 10.1021/acsenergylett.1c01348.

[ref66] Xu Z.; Huang Z.; Jin T.; Lian T.; Tang M. L. Mechanistic Understanding and Rational Design of Quantum Dot/Mediator Interfaces for Efficient Photon Upconversion. Acc. Chem. Res. 2021, 54, 70–80. 10.1021/acs.accounts.0c00526. 33141563

[ref67] Mondal N.; De A.; Das S.; Paul S.; Samanta A. Ultrafast Carrier Dynamics of Metal Halide Perovskite Nanocrystals and Perovskite-Composites. Nanoscale 2019, 11, 9796–9818. 10.1039/C9NR01745C. 31070653

[ref68] Palabathuni M.; Akhil S.; Singh R.; Mishra N. Charge Transfer in Photoexcited Cesium-Lead Halide Perovskite Nanocrystals: Review of Materials and Applications. Acs Appl. Nano Mater. 2022, 5, 10097–10117. 10.1021/acsanm.2c01550.

[ref69] Liu X. C.; Zeng P.; Chen S. H.; Smith T. A.; Liu M. Z. Charge Transfer Dynamics at the Interface of CsPbX3 Perovskite Nanocrystal-Acceptor Complexes: A Femtosecond Transient Absorption Spectroscopy Study. Laser Photonics Rev. 2022, 16, 2200280 10.1002/lpor.202200280.

[ref70] DuBose J. T.; Kamat P. V. Energy Versus Electron Transfer: Managing Excited-State Interactions in Perovskite Nanocrystal-Molecular Hybrids. Chem. Rev. 2022, 122, 12475–12494. 10.1021/acs.chemrev.2c00172. 35793168

[ref71] Wang Z.; Inoue Y.; Hisatomi T.; Ishikawa R.; Wang Q.; Takata T.; Chen S.; Shibata N.; Ikuhara Y.; Domen K. Overall water splitting by Ta3N5 nanorod single crystals grown on the edges of KTaO3 particles. Nature Catalysis 2018, 1, 756–763. 10.1038/s41929-018-0134-1.

[ref72] Hisatomi T.; Kubota J.; Domen K. Recent advances in semiconductors for photocatalytic and photoelectrochemical water splitting. Chem. Soc. Rev. 2014, 43, 7520–7535. 10.1039/C3CS60378D. 24413305

[ref73] Wang Y.; Suzuki H.; Xie J.; Tomita O.; Martin D. J.; Higashi M.; Kong D.; Abe R.; Tang J. Mimicking Natural Photosynthesis: Solar to Renewable H2 Fuel Synthesis by Z-Scheme Water Splitting Systems. Chem. Rev. 2018, 118, 5201–5241. 10.1021/acs.chemrev.7b00286. 29676566 PMC5968435

[ref74] Brus L. Size, Dimensionality, and Strong Electron Correlation in Nanoscience. Acc. Chem. Res. 2014, 47, 2951–2959. 10.1021/ar500175h. 25120173

[ref75] Alivisatos A. P. Semiconductor Clusters, Nanocrystals, and Quantum Dots. Science 1996, 271, 933–937. 10.1126/science.271.5251.933.

[ref76] Steigerwald M. L.; Brus L. E. Semiconductor crystallites: a class of large molecules. Acc. Chem. Res. 1990, 23, 183–188. 10.1021/ar00174a003.

[ref77] Efros A. L.; Rosen M. THE ELECTRONIC STRUCTURE OF SEMICONDUCTOR NANOCRYSTALS1. Annu. Rev. Mater. Sci. 2000, 30, 475–521. 10.1146/annurev.matsci.30.1.475.

[ref78] Li L. -s.; Hu J.; Yang W.; Alivisatos A. P. Band Gap Variation of Size- and Shape-Controlled Colloidal CdSe Quantum Rods. Nano Lett. 2001, 1, 349–351. 10.1021/nl015559r.

[ref79] Wu K.; Zhu H.; Lian T. Ultrafast Exciton Dynamics and Light-Driven H2 Evolution in Colloidal Semiconductor Nanorods and Pt-Tipped Nanorods. Acc. Chem. Res. 2015, 48, 851–859. 10.1021/ar500398g. 25682713

[ref80] Ithurria S.; Dubertret B. Quasi 2D Colloidal CdSe Platelets with Thicknesses Controlled at the Atomic Level. J. Am. Chem. Soc. 2008, 130, 16504–16505. 10.1021/ja807724e. 19554725

[ref81] Ithurria S.; Tessier M. D.; Mahler B.; Lobo R. P. S. M.; Dubertret B.; Efros A. L. Colloidal nanoplatelets with two-dimensional electronic structure. Nat. Mater. 2011, 10, 936–941. 10.1038/nmat3145. 22019946

[ref82] Shabaev A.; Efros A. L. 1D Exciton Spectroscopy of Semiconductor Nanorods. Nano Lett. 2004, 4, 1821–1825. 10.1021/nl049216f.

[ref83] Benchamekh R.; Gippius N. A.; Even J.; Nestoklon M. O.; Jancu J. M.; Ithurria S.; Dubertret B.; Efros A. L.; Voisin P. Tight-binding calculations of image-charge effects in colloidal nanoscale platelets of CdSe. Phys. Rev. B 2014, 89, 035307 10.1103/PhysRevB.89.035307.

[ref84] Ekimov A. I.; Hache F.; Schanne-Klein M. C.; Ricard D.; Flytzanis C.; Kudryavtsev I. A.; Yazeva T. V.; Rodina A. V.; Efros A. L. Absorption and Intensity-dependent Photoluminescence Measurements on CdSe Quantum Dots: Assignment of the First Electronic Transitions. J. Opt. Soc. Am. B 1993, 10, 100–107. 10.1364/JOSAB.10.000100.

[ref85] Wang L.-W.; Zunger A. Pseudopotential calculations of nanoscale CdSe quantum dots. Phys. Rev. B 1996, 53, 9579–9582. 10.1103/PhysRevB.53.9579. 9982505

[ref86] Mićić O. I.; Cheong H. M.; Fu H.; Zunger A.; Sprague J. R.; Mascarenhas A.; Nozik A. J. Size-Dependent Spectroscopy of InP Quantum Dots. J. Phys. Chem. B 1997, 101, 4904–4912. 10.1021/jp9704731.

[ref87] Hu J.; Wang; Li L. -s.; Yang W.; Alivisatos A. P. Semiempirical Pseudopotential Calculation of Electronic States of CdSe Quantum Rods. J. Phys. Chem. B 2002, 106, 2447–2452. 10.1021/jp013204q.

[ref88] Pietryga J. M.; Park Y.-S.; Lim J.; Fidler A. F.; Bae W. K.; Brovelli S.; Klimov V. I. Spectroscopic and Device Aspects of Nanocrystal Quantum Dots. Chem. Rev. 2016, 116, 10513–10622. 10.1021/acs.chemrev.6b00169. 27677521

[ref89] Norris D. J.; Bawendi M. G. Measurement and assignment of the size-dependent optical spectrum in CdSe quantum dots. Phys. Rev. B 1996, 53, 16338–16346. 10.1103/PhysRevB.53.16338. 9983472

[ref90] Banin U.; Lee C. J.; Guzelian A. A.; Kadavanich A. V.; Alivisatos A. P.; Jaskolski W.; Bryant G. W.; Efros A. L.; Rosen M. Size-dependent electronic level structure of InAs nanocrystal quantum dots: Test of multiband effective mass theory. J. Chem. Phys. 1998, 109, 2306–2309. 10.1063/1.476797.

[ref91] Efros A. L.; Rosen M. Quantum size level structure of narrow-gap semiconductor nanocrystals: Effect of band coupling. Phys. Rev. B 1998, 58, 7120–7135. 10.1103/PhysRevB.58.7120.

[ref92] Klimov V. I. Spectral and Dynamical Properties of Multilexcitons in Semiconductor Nanocrystals. Annu. Rev. Phys. Chem. 2007, 58, 635–673. 10.1146/annurev.physchem.58.032806.104537. 17163837

[ref93] Bartnik A. C.; Efros A. L.; Koh W. K.; Murray C. B.; Wise F. W. Electronic states and optical properties of PbSe nanorods and nanowires. Phys. Rev. B 2010, 82, 195313 10.1103/PhysRevB.82.195313.

[ref94] Yang J.; Wise F. W. Electronic States of Lead-Salt Nanosheets. J. Phys. Chem. C 2015, 119, 26809–26816. 10.1021/acs.jpcc.5b08207.

[ref95] Norris D. J.; Efros A. L.; Rosen M.; Bawendi M. G. Size Dependence of Exciton Fine Structure in CdSe Quantum Dots. Phys. Rev. B 1996, 53, 16347–16354. 10.1103/PhysRevB.53.16347. 9983473

[ref96] Katz D.; Wizansky T.; Millo O.; Rothenberg E.; Mokari T.; Banin U. Size-Dependent Tunneling and Optical Spectroscopy of CdSe Quantum Rods. Phys. Rev. Lett. 2002, 89, 086801 10.1103/PhysRevLett.89.086801. 12190490

[ref97] Chan Y.; Caruge J.-M.; Snee P. T.; Bawendi M. G. Multiexcitonic two-state lasing in a CdSe nanocrystal laser. Appl. Phys. Lett. 2004, 85, 2460–2462. 10.1063/1.1795368.

[ref98] Vietmeyer F.; McDonald M. P.; Kuno M. Single Nanowire Microscopy and Spectroscopy. J. Phys. Chem. C 2012, 116, 12379–12396. 10.1021/jp3010162.

[ref99] Achtstein A. W.; Schliwa A.; Prudnikau A.; Hardzei M.; Artemyev M. V.; Thomsen C.; Woggon U. Electronic Structure and Exciton–Phonon Interaction in Two-Dimensional Colloidal CdSe Nanosheets. Nano Lett. 2012, 12, 3151–3157. 10.1021/nl301071n. 22625408

[ref100] Chernikov A.; Berkelbach T. C.; Hill H. M.; Rigosi A.; Li Y.; Aslan O. B.; Reichman D. R.; Hybertsen M. S.; Heinz T. F. Exciton Binding Energy and Nonhydrogenic Rydberg Series in Monolayer WS2. Phys. Rev. Lett. 2014, 113, 076802 10.1103/PhysRevLett.113.076802. 25170725

[ref101] Brus L. Commentary: Carbon Nanotubes, CdSe Nanocrystals, and Electron–Electron Interaction. Nano Lett. 2010, 10, 363–365. 10.1021/nl904263b. 20058897

[ref102] Ye Z.; Cao T.; O’Brien K.; Zhu H.; Yin X.; Wang Y.; Louie S. G.; Zhang X. Probing excitonic dark states in single-layer tungsten disulphide. Nature 2014, 513, 214–218. 10.1038/nature13734. 25162523

[ref103] He K.; Kumar N.; Zhao L.; Wang Z.; Mak K. F.; Zhao H.; Shan J. Tightly Bound Excitons in Monolayer WSe_2_. Phys. Rev. Lett. 2014, 113, 026803 10.1103/PhysRevLett.113.026803. 25062219

[ref104] Ugeda M. M.; Bradley A. J.; Shi S.-F.; da Jornada F. H.; Zhang Y.; Qiu D. Y.; Ruan W.; Mo S.-K.; Hussain Z.; Shen Z.-X.; et al. Giant bandgap renormalization and excitonic effects in a monolayer transition metal dichalcogenide semiconductor. Nat. Mater. 2014, 13, 1091–1095. 10.1038/nmat4061. 25173579

[ref105] Dana J.; Haggag O. S.; Dehnel J.; Mor M.; Lifshitz E.; Ruhman S. Testing the fate of nascent holes in CdSe nanocrystals with sub-10 fs pump-probe spectroscopy. Nanoscale 2021, 13, 1982–1987. 10.1039/D0NR07651A. 33443522

[ref106] Sercel P. C.; Efros A. L. Band-Edge Exciton in CdSe and Other II–VI and III–V Compound Semiconductor Nanocrystals–Revisited. Nano Lett. 2018, 18, 4061–4068. 10.1021/acs.nanolett.8b01980. 29927610

[ref107] Becker M. A.; Vaxenburg R.; Nedelcu G.; Sercel P. C.; Shabaev A.; Mehl M. J.; Michopoulos J. G.; Lambrakos S. G.; Bernstein N.; Lyons J. L.; et al. Bright triplet excitons in caesium lead halide perovskites. Nature 2018, 553, 189–193. 10.1038/nature25147. 29323292

[ref108] Shornikova E. V.; Biadala L.; Yakovlev D. R.; Sapega V. F.; Kusrayev Y. G.; Mitioglu A. A.; Ballottin M. V.; Christianen P. C. M.; Belykh V. V.; Kochiev M. V.; et al. Addressing the exciton fine structure in colloidal nanocrystals: the case of CdSe nanoplatelets. Nanoscale 2018, 10, 646–656. 10.1039/C7NR07206F. 29239445

[ref109] Protesescu L.; Yakunin S.; Bodnarchuk M. I.; Krieg F.; Caputo R.; Hendon C. H.; Yang R. X.; Walsh A.; Kovalenko M. V. Nanocrystals of Cesium Lead Halide Perovskites (CsPbX3, X = Cl, Br, and I): Novel Optoelectronic Materials Showing Bright Emission with Wide Color Gamut. Nano Lett. 2015, 15, 3692–3696. 10.1021/nl5048779. 25633588 PMC4462997

[ref110] Sercel P. C.; Lyons J. L.; Wickramaratne D.; Vaxenburg R.; Bernstein N.; Efros A. L. Exciton fine structure in perovskite nanocrystals. Nano Lett. 2019, 19, 4068–4077. 10.1021/acs.nanolett.9b01467. 31088061

[ref111] Wu K.; Liang G.; Shang Q.; Ren Y.; Kong D.; Lian T. Ultrafast Interfacial Electron and Hole Transfer from CsPbBr3 Perovskite Quantum Dots. J. Am. Chem. Soc. 2015, 137, 12792–12795. 10.1021/jacs.5b08520. 26414242

[ref112] Le Thomas N.; Herz E.; Schöps O.; Woggon U.; Artemyev M. V. Exciton Fine Structure in Single CdSe Nanorods. Phys. Rev. Lett. 2005, 94, 016803 10.1103/PhysRevLett.94.016803. 15698113

[ref113] Planelles J.; Rajadell F.; Climente J. I. Hole Band Mixing in CdS and CdSe Quantum Dots and Quantum Rods. J. Phys. Chem. C 2010, 114, 8337–8342. 10.1021/jp102086q.

[ref114] Biadala L.; Liu F.; Tessier M. D.; Yakovlev D. R.; Dubertret B.; Bayer M. Recombination Dynamics of Band Edge Excitons in Quasi-Two-Dimensional CdSe Nanoplatelets. Nano Lett. 2014, 14, 1134–1139. 10.1021/nl403311n. 24559161

[ref115] Achtstein A. W.; Scott R.; Kickhöfel S.; Jagsch S. T.; Christodoulou S.; Bertrand G. H. V.; Prudnikau A. V.; Antanovich A.; Artemyev M.; Moreels I.; et al. $p$-State Luminescence in CdSe Nanoplatelets: Role of Lateral Confinement and a Longitudinal Optical Phonon Bottleneck. Phys. Rev. Lett. 2016, 116, 116802 10.1103/PhysRevLett.116.116802. 27035317

[ref116] Hill H. M.; Rigosi A. F.; Roquelet C.; Chernikov A.; Berkelbach T. C.; Reichman D. R.; Hybertsen M. S.; Brus L. E.; Heinz T. F. Observation of Excitonic Rydberg States in Monolayer MoS2 and WS2 by Photoluminescence Excitation Spectroscopy. Nano Lett. 2015, 15, 2992–2997. 10.1021/nl504868p. 25816155

[ref117] ’t Hooft G. W.; van der Poel W. A. J. A.; Molenkamp L. W.; Foxon C. T. Giant oscillator strength of free excitons in GaAs. Phys. Rev. B 1987, 35, 8281 10.1103/PhysRevB.35.8281. 9941174

[ref118] Feldmann J.; Peter G.; Göbel E. O.; Dawson P.; Moore K.; Foxon C.; Elliott R. J. Linewidth dependence of radiative exciton lifetimes in quantum wells. Phys. Rev. Lett. 1987, 59, 2337–2340. 10.1103/PhysRevLett.59.2337. 10035517

[ref119] Naeem A.; Masia F.; Christodoulou S.; Moreels I.; Borri P.; Langbein W. Giant exciton oscillator strength and radiatively limited dephasing in two-dimensional platelets. Phys. Rev. B 2015, 91, 121302 10.1103/PhysRevB.91.121302.

[ref120] Klimov V. I. Spectral and dynamical properties of multiexcitons in semiconductor nanocrystals. Annu. Rev. Phys. Chem. 2007, 58, 635–673. 10.1146/annurev.physchem.58.032806.104537. 17163837

[ref121] Zhu H.; Yang Y.; Wu K.; Lian T. Charge Transfer Dynamics from Photoexcited Semiconductor Quantum Dots. Annu. Rev. Phys. Chem. 2016, 67, 259–281. 10.1146/annurev-physchem-040215-112128. 27215815

[ref122] Sercel P. C.; Efros A. L. Band-Edge Exciton in CdSe and Other II-VI and III-V Compound Semiconductor Nanocrystals - Revisited. Nano Lett. 2018, 18, 4061–4068. 10.1021/acs.nanolett.8b01980. 29927610

[ref123] Klimov V. I.; McBranch D. W. Femtosecond 1P-to-1S electron relaxation in strongly confined semiconductor nanocrystals. Phys. Rev. Lett. 1998, 80, 4028–4031. 10.1103/PhysRevLett.80.4028.

[ref124] Morgan D. P.; Kelley D. F. What Does the Transient Absorption Spectrum of CdSe Quantum Dots Measure?. J. Phys. Chem. C 2020, 124, 8448–8455. 10.1021/acs.jpcc.0c02566.

[ref125] Labrador T.; Dukovic G. Simultaneous Determination of Spectral Signatures and Decay Kinetics of Excited State Species in Semiconductor Nanocrystals Probed by Transient Absorption Spectroscopy. J. Phys. Chem. C 2020, 124, 8439–8447. 10.1021/acs.jpcc.0c01701.

[ref126] Wang L.; Chen Z.; Liang G.; Li Y.; Lai R.; Ding T.; Wu K. Observation of a phonon bottleneck in copper-doped colloidal quantum dots. Nat. Commun. 2019, 10, 4532 10.1038/s41467-019-12558-y. 31586066 PMC6778069

[ref127] Nozik A. J. Quantum dot solar cells. Physica E: Low-dimensional Systems and Nanostructures 2002, 14, 115–120. 10.1016/S1386-9477(02)00374-0.

[ref128] Gao Y.; Peng X. Photogenerated Excitons in Plain Core CdSe Nanocrystals with Unity Radiative Decay in Single Channel: the Effects of Surface and Ligands. J. Am. Chem. Soc. 2015, 137, 4230–4235. 10.1021/jacs.5b01314. 25785631

[ref129] Qin H.; Niu Y.; Meng R.; Lin X.; Lai R.; Fang W.; Peng X. Single-dot spectroscopy of zinc-blende CdSe/CdS core/shell nanocrystals: nonblinking and correlation with ensemble measurements. J. Am. Chem. Soc. 2014, 136, 179–187. 10.1021/ja4078528. 24345247

[ref130] Wu K.; Lim J.; Klimov V. I. Superposition Principle in Auger Recombination of Charged and Neutral Multicarrier States in Semiconductor Quantum Dots. ACS Nano 2017, 11, 8437–8447. 10.1021/acsnano.7b04079. 28723072

[ref131] Wu K.; Park Y. S.; Lim J.; Klimov V. I. Towards zero-threshold optical gain using charged semiconductor quantum dots. Nat. Nanotechnol 2017, 12, 1140–1147. 10.1038/nnano.2017.189. 29035399

[ref132] Klimov V. V.; Mikhailovsky A. A.; McBranch D. W.; Leatherdale C. A.; Bawendi M. G. Quantization of multiparticle auger rates in semiconductor quantum dots. Science 2000, 287, 1011–1013. 10.1126/science.287.5455.1011. 10669406

[ref133] Zhu H.; Song N.; Rodríguez-Córdoba W.; Lian T. Wave Function Engineering for Efficient Extraction of up to Nineteen Electrons from One CdSe/CdS Quasi-Type II Quantum Dot. J. Am. Chem. Soc. 2012, 134, 4250–4257. 10.1021/ja210312s. 22329340

[ref134] Zhu H.; Yang Y.; Hyeon-Deuk K.; Califano M.; Song N.; Wang Y.; Zhang W.; Prezhdo O. V.; Lian T. Auger-assisted electron transfer from photoexcited semiconductor quantum dots. Nano Lett. 2014, 14, 1263–1269. 10.1021/nl4041687. 24359156

[ref135] Yang Y.; Rodriguez-Cordoba W.; Lian T. Ultrafast Charge Separation and Recombination Dynamics in Lead Sulfide Quantum Dot-Methylene Blue Complexes Probed by Electron and Hole lntraband Transitions. J. Am. Chem. Soc. 2011, 133, 9246–9249. 10.1021/ja2033348. 21615168

[ref136] Yang Y.; Rodríguez-Córdoba W.; Lian T. Multiple Exciton Generation and Dissociation in PbS Quantum Dot-Electron Acceptor Complexes. Nano Lett. 2012, 12, 4235–4241. 10.1021/nl301847r. 22757981

[ref137] Yang Y.; Rodríguez-Córdoba W.; Xiang X.; Lian T. Strong Electronic Coupling and Ultrafast Electron Transfer between PbS Quantum Dots and TiO2 Nanocrystalline Films. Nano Lett. 2012, 12, 303–309. 10.1021/nl2035783. 22182013

[ref138] Kang I.; Wise F. W. Electronic structure and optical properties of PbS and PbSe quantum dots. Journal of the Optical Society of America B 1997, 14, 1632–1646. 10.1364/JOSAB.14.001632.

[ref139] Wise F. W. Lead salt quantum dots: the limit of strong quantum confinement. Acc. Chem. Res. 2000, 33, 773–780. 10.1021/ar970220q. 11087314

[ref140] Shang Q.; Kaledin A. L.; Li Q.; Lian T. Size Dependent Charge Separation and Recombination in CsPbI3 Perovskite Quantum Dots. J. Chem. Phys. 2019, 151, 074705 10.1063/1.5109894. 31438693

[ref141] Wu K.; Liang G.; Shang Q.; Ren Y.; Kong D.; Lian T. Ultrafast Interfacial Electron and Hole Transfer from CsPbBr3 Perovskite Quantum Dots. J. Am. Chem. Soc. 2015, 137, 12792–12795. 10.1021/jacs.5b08520. 26414242

[ref142] Luo X.; Liang G.; Han Y.; Li Y.; Ding T.; He S.; Liu X.; Wu K. Triplet Energy Transfer from Perovskite Nanocrystals Mediated by Electron Transfer. J. Am. Chem. Soc. 2020, 142, 11270–11278. 10.1021/jacs.0c04583. 32479073

[ref143] Huang J.; Huang Z.; Jin S.; Lian T. Exciton Dissociation in CdSe Quantum Dots by Hole Transfer to Phenothiazine. J. Phys. Chem. C 2008, 112, 19734–19738. 10.1021/jp808291u.

[ref144] Klimov V. I.; McBranch D. W.; Leatherdale C. A.; Bawendi M. G. Electron and hole relaxation pathways in semiconductor quantum dots. Phys. Rev. B 1999, 60, 13740–13749. 10.1103/PhysRevB.60.13740.

[ref145] Dana J.; Haggag O.; Mor M.; Dehnel J.; Lifshitz E.; Ruhman S. Testing the fate of nascent holes in CdSe Nanocrystals with sub 10 fs pump-probe spectroscopy. Nanoscale 2021, 13, 1982–1987. 10.1039/D0NR07651A. 33443522

[ref146] Boulesbaa A.; Issac A.; Stockwell D.; Huang Z.; Huang J.; Guo J.; Lian T. Ultrafast charge separation at CdS quantum dot/rhodamine B molecule interface. J. Am. Chem. Soc. 2007, 129, 15132–15133. 10.1021/ja0773406. 18001027

[ref147] Zhu H.; Song N.; Lian T. Wave Function Engineering for Ultrafast Charge Separation and Slow Charge Recombination in Type II Core/Shell Quantum Dots. J. Am. Chem. Soc. 2011, 133, 8762–8771. 10.1021/ja202752s. 21534569

[ref148] Morris-Cohen A. J.; Frederick M. T.; Cass L. C.; Weiss E. A. Simultaneous determination of the adsorption constant and the photoinduced electron transfer rate for a CdS quantum dot-viologen complex. J. Am. Chem. Soc. 2011, 133, 10146–10154. 10.1021/ja2010237. 21618976

[ref149] Huang J.; Stockwell D.; Huang Z.; Mohler D. L.; Lian T. Photoinduced ultrafast electron transfer from CdSe quantum dots to Re-bipyridyl complexes. J. Am. Chem. Soc. 2008, 130, 5632–5633. 10.1021/ja8003683. 18393497

[ref150] Wu K.; Zhu H.; Liu Z.; Rodriguez-Cordoba W.; Lian T. Ultrafast Charge Separation and Long-Lived Charge Separated State in Photocatalytic CdS-Pt Nanorod Heterostructures. J. Am. Chem. Soc. 2012, 134, 10337–10340. 10.1021/ja303306u. 22655858

[ref151] Zhu H.; Lian T. Enhanced Multiple Exciton Dissociation from CdSe Quantum Rods: The Effect of Nanocrystal Shape. J. Am. Chem. Soc. 2012, 134, 11289–11297. 10.1021/ja304724u. 22702343

[ref152] Wu K.; Du Y.; Tang H.; Chen Z.; Lian T. Efficient Extraction of Trapped Holes from Colloidal CdS Nanorods. J. Am. Chem. Soc. 2015, 137, 10224–10230. 10.1021/jacs.5b04564. 26221916

[ref153] Wu K.; Chen J.; McBride J. R.; Lian T. CHARGE TRANSFER. Efficient hot-electron transfer by a plasmon-induced interfacial charge-transfer transition. Science 2015, 349, 632–635. 10.1126/science.aac5443. 26250682

[ref154] Wu K.; Li Q.; Du Y.; Chen Z.; Lian T. Ultrafast exciton quenching by energy and electron transfer in colloidal CdSe nanosheet-Pt heterostructures. Chemical Science 2015, 6, 1049–1054. 10.1039/C4SC02994A. 29560193 PMC5811111

[ref155] Li Q.; Zhao F.; Qu C.; Shang Q.; Xu Z.; Yu L.; McBride J. R.; Lian T. Two-Dimensional Morphology Enhances Light-Driven H2 Generation Efficiency in CdS Nanoplatelet-Pt Heterostructures. J. Am. Chem. Soc. 2018, 140, 11726–11734. 10.1021/jacs.8b06100. 30145886

[ref156] Li Q.; He S.; Lian T. How Exciton and Single Carriers Block the Excitonic Transition in Two-Dimensional Cadmium Chalcogenide Nanoplatelets. Nano Lett. 2020, 20, 6162–6169. 10.1021/acs.nanolett.0c02461. 32697589

[ref157] Wu K.; Song N.; Liu Z.; Zhu H.; Rodriguez-Cordoba W.; Lian T. Interfacial Charge Separation and Recombination in InP and Quasi-Type II InP/CdS Core/Shell Quantum Dot-Molecular Acceptor Complexes. J. Phys. Chem. A 2013, 117, 7561–7570. 10.1021/jp402425w. 23639000

[ref158] Yang W.; Yang Y.; Kaledin A. L.; He S.; Jin T.; McBride J. R.; Lian T. Surface passivation extends single and biexciton lifetimes of InP quantum dots. Chemical Science 2020, 11, 5779–5789. 10.1039/D0SC01039A. 32832054 PMC7416692

[ref159] Wu K.; Liu Z.; Zhu H.; Lian T. Exciton Annihilation and Dissociation Dynamics in Group II-V Cd3P2 Quantum Dots. J. Phys. Chem. A 2013, 117, 6362–6372. 10.1021/jp402511m. 23611312

[ref160] Knowles K. E.; McArthur E. A.; Weiss E. A. A multi-timescale map of radiative and nonradiative decay pathways for excitons in CdSe quantum dots. ACS Nano 2011, 5, 2026–2035. 10.1021/nn2002689. 21361353

[ref161] Klimov V. I.; Schwarz C. J.; McBranch D. W.; Leatherdale C. A.; Bawendi M. G. Ultrafast dynamics of inter- and intraband transitions in semiconductor nanocrystals: Implications for quantum-dot lasers. Phys. Rev. B 1999, 60, R2177–R2180. 10.1103/PhysRevB.60.R2177.

[ref162] Grimaldi G.; Geuchies J. J.; Van Der Stam W.; Du Fossé I.; Brynjarsson B.; Kirkwood N.; Kinge S.; Siebbeles L. D. A.; Houtepen A. J. Spectroscopic Evidence for the Contribution of Holes to the Bleach of Cd-Chalcogenide Quantum Dots. Nano Lett. 2019, 19, 3002–3010. 10.1021/acs.nanolett.9b00164. 30938530 PMC6509645

[ref163] He S.; Li Q.; Jin T.; Lian T. Contributions of Exciton Fine Structure and Hole Trapping on the Hole State Filling Effect in the Transient Absorption Spectra of CdSe Quantum Dots. J. Chem. Phys. 2022, 156, 054704 10.1063/5.0081192. 35135264

[ref164] Li Q.; He S.; Lian T. How Exciton and Single Carriers Block the Excitonic Transition in Two-Dimensional Cadmium Chalcogenide Nanoplatelets. Nano Lett. 2020, 20, 6162–6169. 10.1021/acs.nanolett.0c02461. 32697589

[ref165] Taheri M. M.; Elbert K. C.; Yang S.; Diroll B. T.; Park J.; Murray C. B.; Baxter J. B. Distinguishing Electron and Hole Dynamics in Functionalized CdSe/CdS Core/Shell Quantum Dots Using Complementary Ultrafast Spectroscopies and Kinetic Modeling. J. Phys. Chem. C 2021, 125, 31–41. 10.1021/acs.jpcc.0c07037.

[ref166] Huang Z.; Wang S.; Ren Y.; Wang Y. Observable Hole-State Kinetics and Its Implications for Optical Gain in Hole-Engineered Quantum Dots. ACS Photonics 2023, 10, 639–646. 10.1021/acsphotonics.2c01648.

[ref167] Brosseau P. J.; Geuchies J. J.; Jasrasaria D.; Houtepen A. J.; Rabani E.; Kambhampati P. Ultrafast hole relaxation dynamics in quantum dots revealed by two-dimensional electronic spectroscopy. Commun. Phys. 2023, 6, 48 10.1038/s42005-023-01169-1.

[ref168] Zhu H.; Lian T. Wavefunction engineering in quantum confined semiconductor nanoheterostructures for efficient charge separation and solar energy conversion. Energy & Environ. Sci. 2012, 5, 9406–9418. 10.1039/c2ee22679k.

[ref169] Reiss P.; Protière M.; Li L. Core/Shell Semiconductor Nanocrystals. Small 2009, 5, 154–168. 10.1002/smll.200800841. 19153991

[ref170] Talapin D. V.; Koeppe R.; Götzinger S.; Kornowski A.; Lupton J. M.; Rogach A. L.; Benson O.; Feldmann J.; Weller H. Highly Emissive Colloidal CdSe/CdS Heterostructures of Mixed Dimensionality. Nano Lett. 2003, 3, 1677–1681. 10.1021/nl034815s.

[ref171] Steiner D.; Dorfs D.; Banin U.; Della Sala F.; Manna L.; Millo O. Determination of Band Offsets in Heterostructured Colloidal Nanorods Using Scanning Tunneling Spectroscopy. Nano Lett. 2008, 8, 2954–2958. 10.1021/nl801848x. 18690751

[ref172] She C.; Demortière A.; Shevchenko E. V.; Pelton M. Using Shape to Control Photoluminescence from CdSe/CdS Core/Shell Nanorods. J. Phys. Chem. Lett. 2011, 2, 1469–1475. 10.1021/jz200510f.

[ref173] Lupo M. G.; Della Sala F.; Carbone L.; Zavelani-Rossi M.; Fiore A.; Lüer L.; Polli D.; Cingolani R.; Manna L.; Lanzani G. Ultrafast Electron–Hole Dynamics in Core/Shell CdSe/CdS Dot/Rod Nanocrystals. Nano Lett. 2008, 8, 4582–4587. 10.1021/nl8028366. 19367887

[ref174] Smith E. R.; Luther J. M.; Johnson J. C. Ultrafast Electronic Delocalization in CdSe/CdS Quantum Rod Heterostructures. Nano Lett. 2011, 11, 4923–4931. 10.1021/nl202869z. 22011256

[ref175] Rainò G.; Stöferle T.; Moreels I.; Gomes R.; Kamal J. S.; Hens Z.; Mahrt R. F. Probing the Wave Function Delocalization in CdSe/CdS Dot-in-Rod Nanocrystals by Time- and Temperature-Resolved Spectroscopy. ACS Nano 2011, 5, 4031–4036. 10.1021/nn2005969. 21504193

[ref176] Sitt A.; Sala F. D.; Menagen G.; Banin U. Multiexciton Engineering in Seeded Core/Shell Nanorods: Transfer from Type-I to Quasi-type-II Regimes. Nano Lett. 2009, 9, 3470–3476. 10.1021/nl901679q. 19655723

[ref177] Wu K.; Hill L. J.; Chen J.; McBride J. R.; Pavlopolous N. G.; Richey N. E.; Pyun J.; Lian T. Universal Length Dependence of Rod-to-Seed Exciton Localization Efficiency in Type I and Quasi-Type II CdSe@CdS Nanorods. ACS Nano 2015, 9, 4591–4599. 10.1021/acsnano.5b01245. 25803834

[ref178] Christodoulou S.; Rajadell F.; Casu A.; Vaccaro G.; Grim J. Q.; Genovese A.; Manna L.; Climente J. I.; Meinardi F.; Raino G.; et al. Band structure engineering via piezoelectric fields in strained anisotropic CdSe/CdS nanocrystals. Nat. Commun. 2015, 6, 7905 10.1038/ncomms8905. 26219691 PMC4532876

[ref179] García-Santamaría F.; Chen Y.; Vela J.; Schaller R. D.; Hollingsworth J. A.; Klimov V. I. Suppressed Auger Recombination in “Giant” Nanocrystals Boosts Optical Gain Performance. Nano Lett. 2009, 9, 3482–3488. 10.1021/nl901681d. 19505082 PMC2897714

[ref180] Sitt A.; Hadar I.; Banin U. Band-gap engineering, optoelectronic properties and applications of colloidal heterostructured semiconductor nanorods. Nano Today 2013, 8, 494–513. 10.1016/j.nantod.2013.08.002.

[ref181] Talapin D. V.; Nelson J. H.; Shevchenko E. V.; Aloni S.; Sadtler B.; Alivisatos A. P. Seeded Growth of Highly Luminescent CdSe/CdS Nanoheterostructures with Rod and Tetrapod Morphologies. Nano Lett. 2007, 7, 2951–2959. 10.1021/nl072003g. 17845068

[ref182] Carbone L.; Nobile C.; De Giorgi M.; Sala F. D.; Morello G.; Pompa P.; Hytch M.; Snoeck E.; Fiore A.; Franchini I. R.; et al. Synthesis and Micrometer-Scale Assembly of Colloidal CdSe/CdS Nanorods Prepared by a Seeded Growth Approach. Nano Lett. 2007, 7, 2942–2950. 10.1021/nl0717661. 17845067

[ref183] Halpert J. E.; Porter V. J.; Zimmer J. P.; Bawendi M. G. Synthesis of CdSe/CdTe Nanobarbells. J. Am. Chem. Soc. 2006, 128, 12590–12591. 10.1021/ja0616534. 17002320

[ref184] Kirsanova M.; Nemchinov A.; Hewa-Kasakarage N. N.; Schmall N.; Zamkov M. Synthesis of ZnSe/CdS/ZnSe Nanobarbells Showing Photoinduced Charge Separation. Chem. Mater. 2009, 21, 4305–4309. 10.1021/cm901615n.

[ref185] Li H.; Brescia R.; Krahne R.; Bertoni G.; Alcocer M. J. P.; D’Andrea C.; Scotognella F.; Tassone F.; Zanella M.; De Giorgi M.; et al. Blue-UV-Emitting ZnSe(Dot)/ZnS(Rod) Core/Shell Nanocrystals Prepared from CdSe/CdS Nanocrystals by Sequential Cation Exchange. ACS Nano 2012, 6, 1637–1647. 10.1021/nn204601n. 22283644

[ref186] Dorfs D.; Salant A.; Popov I.; Banin U. ZnSe Quantum Dots Within CdS Nanorods: A Seeded-Growth Type-II System. Small 2008, 4, 1319–1323. 10.1002/smll.200800084. 18680096

[ref187] Müller J.; Lupton J. M.; Rogach A. L.; Feldmann J.; Talapin D. V.; Weller H. Monitoring Surface Charge Movement in Single Elongated Semiconductor Nanocrystals. Phys. Rev. Lett. 2004, 93, 167402 10.1103/PhysRevLett.93.167402. 15525031

[ref188] Müller J.; Lupton J. M.; Lagoudakis P. G.; Schindler F.; Koeppe R.; Rogach A. L.; Feldmann J.; Talapin D. V.; Weller H. Wave Function Engineering in Elongated Semiconductor Nanocrystals with Heterogeneous Carrier Confinement. Nano Lett. 2005, 5, 2044–2049. 10.1021/nl051596x. 16218735

[ref189] Müller J.; Lupton J. M.; Rogach A. L.; Feldmann J.; Talapin D. V.; Weller H. Monitoring Surface Charge Migration in the Spectral Dynamics of Single CdSe/CdS Nanodot/Nanorod Heterostructures. Phys. Rev. B 2005, 72, 205339 10.1103/PhysRevB.72.205339.

[ref190] Kraus R. M.; Lagoudakis P. G.; Rogach A. L.; Talapin D. V.; Weller H.; Lupton J. M.; Feldmann J. Room-Temperature Exciton Storage in Elongated Semiconductor Nanocrystals. Phys. Rev. Lett. 2007, 98, 017401 10.1103/PhysRevLett.98.017401. 17358504

[ref191] Mauser C.; Limmer T.; Da Como E.; Becker K.; Rogach A. L.; Feldmann J.; Talapin D. V. Anisotropic Optical Emission of Single CdSe/CdS Tetrapod Heterostructures: Evidence for a Wavefunction Symmetry Breaking. Phys. Rev. B 2008, 77, 153303 10.1103/PhysRevB.77.153303.

[ref192] Morello G.; Della Sala F.; Carbone L.; Manna L.; Maruccio G.; Cingolani R.; De Giorgi M. Intrinsic Optical Nonlinearity in Colloidal Seeded Grown CdSe/CdS Nanostructures: Photoinduced Screening of The Internal Electric Field. Phys. Rev. B 2008, 78, 195313 10.1103/PhysRevB.78.195313.

[ref193] Luo Y.; Wang L.-W. Electronic Structures of the CdSe/CdS Core–Shell Nanorods. ACS Nano 2010, 4, 91–98. 10.1021/nn9010279. 20043692

[ref194] Saba M.; Minniberger S.; Quochi F.; Roither J.; Marceddu M.; Gocalinska A.; Kovalenko M. V.; Talapin D. V.; Heiss W.; Mura A.; et al. Exciton-Exciton Interaction and Optical Gain in Colloidal CdSe/CdS Dot/Rod Nanocrystals. Adv. Mater. 2009, 21, 4942 10.1002/adma.200901482. 25376736

[ref195] Borys N. J.; Walter M. J.; Huang J.; Talapin D. V.; Lupton J. M. The Role of Particle Morphology in Interfacial Energy Transfer in CdSe/CdS Heterostructure Nanocrystals. Science 2010, 330, 1371–1374. 10.1126/science.1198070. 21127250

[ref196] Lupo M. G.; Zavelani-Rossi M.; Fiore A.; Polli D.; Carbone L.; Cingolani R.; Manna L.; Lanzani G. Evidence of electron wave function delocalization in CdSe/CdS asymmetric nanocrystals. Superlattices Microstruct. 2010, 47, 170–173. 10.1016/j.spmi.2009.09.006.

[ref197] Lutich A. A.; Mauser C.; Da Como E.; Huang J.; Vaneski A.; Talapin D. V.; Rogach A. L.; Feldmann J. Multiexcitonic Dual Emission in CdSe/CdS Tetrapods and Nanorods. Nano Lett. 2010, 10, 4646–4650. 10.1021/nl1028057. 20964399

[ref198] Mauser C.; Da Como E.; Baldauf J.; Rogach A. L.; Huang J.; Talapin D. V.; Feldmann J. Spatio-temporal Dynamics of Coupled Electrons and Holes in Nanosize CdSe-CdS Semiconductor Tetrapods. Phys. Rev. B 2010, 82, 081306 10.1103/PhysRevB.82.081306.

[ref199] Morello G.; Della Sala F.; Carbone L.; Manna L.; Cingolani R.; De Giorgi M. Evidence for an Internal field in CdSe/CdS Nanorods by Time Resolved and Single Rod Experiments. Superlattices Microstruct. 2010, 47, 174–177. 10.1016/j.spmi.2009.07.030.

[ref200] Zavelani-Rossi M.; Lupo M. G.; Tassone F.; Manna L.; Lanzani G. Suppression of Biexciton Auger Recombination in CdSe/CdS Dot/Rods: Role of the Electronic Structure in the Carrier Dynamics. Nano Lett. 2010, 10, 3142–3150. 10.1021/nl101930z. 20698629

[ref201] Krahne R.; Morello G.; Figuerola A.; George C.; Deka S.; Manna L. Physical Properties of Elongated Inorganic Nanoparticles. Phys. Rep. 2011, 501, 75–221. 10.1016/j.physrep.2011.01.001.

[ref202] Xing G.; Chakrabortty S.; Ngiam S. W.; Chan Y.; Sum T. C. Three-Photon Absorption in Seeded CdSe/CdS Nanorod Heterostructures. J. Phys. Chem. C 2011, 115, 17711–17716. 10.1021/jp205238q.

[ref203] Rainò G.; Stöferle T.; Moreels I.; Gomes R.; Hens Z.; Mahrt R. F. Controlling the Exciton Fine Structure Splitting in CdSe/CdS Dot-in-Rod Nanojunctions. ACS Nano 2012, 6, 1979–1987. 10.1021/nn204447e. 22364241

[ref204] Chakrabortty S.; Xing G.; Xu Y.; Ngiam S. W.; Mishra N.; Sum T. C.; Chan Y. Engineering Fluorescence in Au-Tipped, CdSe-Seeded CdS Nanoheterostructures. Small 2011, 7, 2847–2852. 10.1002/smll.201100976. 21990190

[ref205] Tang M. L.; Grauer D. C.; Lassalle-Kaiser B.; Yachandra V. K.; Amirav L.; Long J. R.; Yano J.; Alivisatos A. P. Structural and Electronic Study of an Amorphous MoS3 Hydrogen-Generation Catalyst on a Quantum-Controlled Photosensitizer. Angew. Chem., Int. Ed. 2011, 50, 10203–10207. 10.1002/anie.201104412. 21956994

[ref206] Zhu H.; Song N.; Lv H.; Hill C. L.; Lian T. Near Unity Quantum Yield of Light-Driven Redox Mediator Reduction and Efficient H2 Generation Using Colloidal Nanorod Heterostructures. J. Am. Chem. Soc. 2012, 134, 11701–11708. 10.1021/ja303698e. 22721499

[ref207] Wu K.; Rodríguez-Córdoba W. E.; Liu Z.; Zhu H.; Lian T. Beyond Band Alignment: Hole Localization Driven Formation of Three Spatially Separated Long-Lived Exciton States in CdSe/CdS Nanorods. ACS Nano 2013, 7, 7173–7185. 10.1021/nn402597p. 23829512

[ref208] Klimov V. I. Optical Nonlinearities and Ultrafast Carrier Dynamics in Semiconductor Nanocrystals. J. Phys. Chem. B 2000, 104, 6112–6123. 10.1021/jp9944132.

[ref209] Pedetti S.; Ithurria S.; Heuclin H.; Patriarche G.; Dubertret B. Type-II CdSe/CdTe Core/Crown Semiconductor Nanoplatelets. J. Am. Chem. Soc. 2014, 136, 16430–16438. 10.1021/ja509307m. 25338215

[ref210] Tessier M. D.; Spinicelli P.; Dupont D.; Patriarche G.; Ithurria S.; Dubertret B. Efficient Exciton Concentrators Built from Colloidal Core/Crown CdSe/CdS Semiconductor Nanoplatelets. Nano Lett. 2014, 14, 207–213. 10.1021/nl403746p. 24328730

[ref211] Wu K.; Li Q.; Jia Y.; McBride J. R.; Xie Z. -x.; Lian T. Efficient and Ultrafast Formation of Long-Lived Charge-Transfer Exciton State in Atomically Thin Cadmium Selenide/Cadmium Telluride Type-II Heteronanosheets. ACS Nano 2015, 9, 961–968. 10.1021/nn506796m. 25548944

[ref212] Li Q.; Zhou B.; McBride J. R.; Lian T. Efficient Diffusive Transport of Hot and Cold Excitons in Colloidal Type II CdSe/CdTe Core/Crown Nanoplatelet Heterostructures. ACS Energy Letters 2017, 2, 174–181. 10.1021/acsenergylett.6b00634.

[ref213] Li Q.; Xu Z.; McBride J. R.; Lian T. Low Threshold Multiexciton Optical Gain in Colloidal CdSe/CdTe Core/Crown Type-II Nanoplatelet Heterostructures. ACS Nano 2017, 11, 2545–2553. 10.1021/acsnano.6b08674. 28157330

[ref214] Guzelturk B.; Kelestemur Y.; Olutas M.; Li Q.; Lian T.; Demir H. V. High-Efficiency Optical Gain in Type-II Semiconductor Nanocrystals of Alloyed Colloidal Quantum Wells. J. Phys. Chem. Lett. 2017, 8, 5317–5324. 10.1021/acs.jpclett.7b02367. 29022715

[ref215] Kelestemur Y.; Olutas M.; Delikanli S.; Guzelturk B.; Akgul M. Z.; Demir H. V. Type-II Colloidal Quantum Wells: CdSe/CdTe Core/Crown Heteronanoplatelets. J. Phys. Chem. C 2015, 119, 2177–2185. 10.1021/jp510466k.

[ref216] Antanovich A. V.; Prudnikau A. V.; Melnikau D.; Rakovich Y. P.; Chuvilin A.; Woggon U.; Achtstein A. W.; Artemyev M. V. Colloidal synthesis and optical properties of type-II CdSe-CdTe and inverted CdTe-CdSe core-wing heteronanoplatelets. Nanoscale 2015, 7, 8084–8092. 10.1039/C4NR07134D. 25873332

[ref217] Liu B.; Delikanli S.; Gao Y.; Dede D.; Gungor K.; Demir H. V. Nanocrystal light-emitting diodes based on type II nanoplatelets. Nano Energy 2018, 47, 115–122. 10.1016/j.nanoen.2018.02.004.

[ref218] Pandya R.; Chen R.; Cheminal A.; Dufour M.; Richter J. M.; Thomas T. H.; Ahmed S.; Sadhanala A.; Booker E. P.; Divitini G.; et al. Exciton-Phonon Interactions Govern Charge-Transfer-State Dynamics in CdSe/CdTe Two-Dimensional Colloidal Heterostructures. J. Am. Chem. Soc. 2018, 140, 14097–14111. 10.1021/jacs.8b05842. 30293427

[ref219] Cassette E.; Pedetti S.; Mahler B.; Ithurria S.; Dubertret B.; Scholes G. Ultrafast exciton dynamics in 2D in-plane hetero-nanostructures: delocalization and charge transfer. Phys. Chem. Chem. Phys. 2017, 19, 8373–8379. 10.1039/C6CP08689F. 28280802

[ref220] Delikanli S.; Guzelturk B.; Hernández-Martínez P. L.; Erdem T.; Kelestemur Y.; Olutas M.; Akgul M. Z.; Demir H. V. Continuously Tunable Emission in Inverted Type-I CdS/CdSe Core/Crown Semiconductor Nanoplatelets. Adv. Funct. Mater. 2015, 25, 4282–4289. 10.1002/adfm.201500403.

[ref221] Li Q.; Wu K.; Chen J.; Chen Z.; McBride J. R.; Lian T. Size-Independent Exciton Localization Efficiency in Colloidal CdSe/CdS Core/Crown Nanosheet Type-I Heterostructures. ACS Nano 2016, 10, 3843–3851. 10.1021/acsnano.6b00787. 26872065

[ref222] Guzelturk B.; Kelestemur Y.; Olutas M.; Delikanli S.; Demir H. V. Amplified Spontaneous Emission and Lasing in Colloidal Nanoplatelets. ACS Nano 2014, 8, 6599–6605. 10.1021/nn5022296. 24882737

[ref223] Naskar S.; Lübkemann F.; Hamid S.; Freytag A.; Wolf A.; Koch J.; Ivanova I.; Pfnür H.; Dorfs D.; Bahnemann D. W.; et al. Synthesis of Ternary and Quaternary Au and Pt Decorated CdSe/CdS Heteronanoplatelets with Controllable Morphology. Adv. Funct. Mater. 2017, 27, 1604685 10.1002/adfm.201604685.

[ref224] Yang Y.; Wu K.; Shabaev A.; Efros A. L.; Lian T.; Beard M. C. Direct Observation of Photoexcited Hole Localization in CdSe Nanorods. ACS Energy Letters 2016, 1, 76–81. 10.1021/acsenergylett.6b00036.

[ref225] Ding T.; Liang G.; Wang J.; Wu K. Carrier-doping as a tool to probe the electronic structure and multi-carrier recombination dynamics in heterostructured colloidal nanocrystals. Chemical Science 2018, 9, 7253–7260. 10.1039/C8SC01926F. 30288246 PMC6148752

[ref226] Christodoulou S.; Rajadell F.; Casu A.; Vaccaro G.; Grim J. Q.; Genovese A.; Manna L.; Climente J. I.; Meinardi F.; Rainò G.; et al. Band structure engineering via piezoelectric fields in strained anisotropic CdSe/CdS nanocrystals. Nat. Commun. 2015, 6, 7905 10.1038/ncomms8905. 26219691 PMC4532876

[ref227] Smith A. M.; Mohs A. M.; Nie S. Tuning the optical and electronic properties of colloidal nanocrystals by lattice strain. Nat. Nanotechnol. 2009, 4, 56–63. 10.1038/nnano.2008.360. 19119284 PMC2711767

[ref228] McDaniel H.; Pelton M.; Oh N.; Shim M. Effects of Lattice Strain and Band Offset on Electron Transfer Rates in Type-II Nanorod Heterostructures. J. Phys. Chem. Lett. 2012, 3, 1094–1098. 10.1021/jz300275f. 26288042

[ref229] Yang S.; Prendergast D.; Neaton J. B. Strain-Induced Band Gap Modification in Coherent Core/Shell Nanostructures. Nano Lett. 2010, 10, 3156–3162. 10.1021/nl101999p. 20698631

[ref230] McDaniel H.; Zuo J.-M.; Shim M. Anisotropic Strain-Induced Curvature in Type-II CdSe/CdTe Nanorod Heterostructures. J. Am. Chem. Soc. 2010, 132, 3286–3288. 10.1021/ja910233a. 20163144

[ref231] Chuang C.-H.; Doane T. L.; Lo S. S.; Scholes G. D.; Burda C. Measuring Electron and Hole Transfer in Core/Shell Nanoheterostructures. ACS Nano 2011, 5, 6016–6024. 10.1021/nn201788f. 21671650

[ref232] Lo S. S.; Mirkovic T.; Chuang C.-H.; Burda C.; Scholes G. D. Emergent Properties Resulting from Type-II Band Alignment in Semiconductor Nanoheterostructures. Adv. Mater. 2011, 23, 180–197. 10.1002/adma.201002290. 21069886

[ref233] Jin S.; Zhang J.; Schaller R. D.; Rajh T.; Wiederrecht G. P. Ultrafast Charge Separation from Highly Reductive ZnTe/CdSe Type II Quantum Dots. J. Phys. Chem. Lett. 2012, 3, 2052–2058. 10.1021/jz3008886.

[ref234] Zhu H. M.; Song N. H.; Lian T. Q. Wave Function Engineering for Ultrafast Charge Separation and Slow Charge Recombination in Type II Core/Shell Quantum Dots. J. Am. Chem. Soc. 2011, 133, 8762–8771. 10.1021/ja202752s. 21534569

[ref235] Dooley C. J.; Dimitrov S. D.; Fiebig T. Ultrafast Electron Transfer Dynamics in CdSe/CdTe Donor–Acceptor Nanorods. J. Phys. Chem. C 2008, 112, 12074–12076. 10.1021/jp804040r.

[ref236] Hewa-Kasakarage N. N.; El-Khoury P. Z.; Tarnovsky A. N.; Kirsanova M.; Nemitz I.; Nemchinov A.; Zamkov M. Ultrafast Carrier Dynamics in Type II ZnSe/CdS/ZnSe Nanobarbells. ACS Nano 2010, 4, 1837–1844. 10.1021/nn100229x. 20337435

[ref237] Smith A. M.; Nie S. Semiconductor Nanocrystals: Structure, Properties, and Band Gap Engineering. Acc. Chem. Res. 2010, 43, 190–200. 10.1021/ar9001069. 19827808 PMC2858563

[ref238] Warman J. M.; De Haas M. P.; Van Hovell tot Westerflier S. W. F. M.; Binsma J. J. M.; Kolar Z. I. Electronic processes in semiconductor materials studied by nanosecond time-resolved microwave conductivity. 1. Cadmium sulfide macroscopic crystal. J. Phys. Chem. 1989, 93, 5895–5899. 10.1021/j100352a048.

[ref239] Islam M. N.; Woods J. The Effect of Crystal Inhomogeneity on the Threshold Field for Current Saturation in Photoconducting CdS. J. Phys. D: Appl. Phys. 1970, 3, 1297 10.1088/0022-3727/3/8/420.

[ref240] Spear W. E.; Mort J. Electron and Hole Transport in CdS Crystals. Proc. Phys. Soc. 1963, 81, 130 10.1088/0370-1328/81/1/319.

[ref241] Mort J.; Spear W. E. Hole Drift Mobility and Lifetime in CdS Crystals. Phys. Rev. Lett. 1962, 8, 314–315. 10.1103/PhysRevLett.8.314.

[ref242] Islam M. N.; Woods J. Acoustoelectric Interaction and the Drift Mobility of Holes in CdS. Solid State Commun. 1969, 7, 1457–1461. 10.1016/0038-1098(69)90021-0.

[ref243] Yang Y.; Wu K.; Chen Z.; Jeong B.-S.; Lian T. Competition of branch-to-core exciton localization and interfacial electron transfer in CdSe tetrapods. Chem. Phys. 2016, 471, 32–38. 10.1016/j.chemphys.2015.08.011.

[ref244] Singh S.; Tomar R.; ten Brinck S.; De Roo J.; Geiregat P.; Martins J. C.; Infante I.; Hens Z. Colloidal CdSe Nanoplatelets, A Model for Surface Chemistry/Optoelectronic Property Relations in Semiconductor Nanocrystals. J. Am. Chem. Soc. 2018, 140, 13292–13300. 10.1021/jacs.8b07566. 30253644

[ref245] Li Q.; Lian T. Exciton Spatial Coherence and Optical Gain in Colloidal Two-Dimensional Cadmium Chalcogenide Nanoplatelets. Acc. Chem. Res. 2019, 52, 2684–2693. 10.1021/acs.accounts.9b00252. 31433164

[ref246] Kunneman L. T.; Tessier M. D.; Heuclin H.; Dubertret B.; Aulin Y. V.; Grozema F. C.; Schins J. M.; Siebbeles L. D. A. Bimolecular Auger Recombination of Electron-Hole Pairs in Two-Dimensional CdSe and CdSe/CdZnS Core/Shell Nanoplatelets. J. Phys. Chem. Lett. 2013, 4, 3574–3578. 10.1021/jz401970p.

[ref247] Liu Y.-H.; Wayman V. L.; Gibbons P. C.; Loomis R. A.; Buhro W. E. Origin of High Photoluminescence Efficiencies in CdSe Quantum Belts. Nano Lett. 2010, 10, 352–357. 10.1021/nl903740p. 20014799

[ref248] Ma X.; Diroll B. T.; Cho W.; Fedin I.; Schaller R. D.; Talapin D. V.; Gray S. K.; Wiederrecht G. P.; Gosztola D. J. Size-Dependent Biexciton Quantum Yields and Carrier Dynamics of Quasi-Two-Dimensional Core/Shell Nanoplatelets. ACS Nano 2017, 11, 9119–9127. 10.1021/acsnano.7b03943. 28787569

[ref249] Marcus R. A.; Sutin N. Electron transfers in chemistry and biology. Biochimica et Biophysica Acta (BBA) - Reviews on Bioenergetics 1985, 811, 265–322. 10.1016/0304-4173(85)90014-X.

[ref250] Ellis J. L.; Hickstein D. D.; Schnitzenbaumer K. J.; Wilker M. B.; Palm B. B.; Jimenez J. L.; Dukovic G.; Kapteyn H. C.; Murnane M. M.; Xiong W. Solvents Effects on Charge Transfer from Quantum Dots. J. Am. Chem. Soc. 2015, 137, 3759–3762. 10.1021/jacs.5b00463. 25751367

[ref251] Kaledin A. L.; Lian T.; Hill C. L.; Musaev D. G. A Hybrid Quantum Mechanical Approach: Intimate Details of Electron Transfer between Type-I CdSe/ZnS Quantum Dots and an Anthraquinone Molecule. journal of physical chemistry. B 2015, 119, 7651–7658. 10.1021/jp511935z. 25604315

[ref252] Marcus R. A. ON THEORY OF ELECTRON-TRANSFER REACTIONS 0.6. UNIFIED TREATMENT FOR HOMOGENEOUS AND ELECTRODE REACTIONS. J. Chem. Phys. 1965, 43, 679–701. 10.1063/1.1696792.

[ref253] Tvrdy K.; Frantsuzov P. A.; Kamat P. V. Photoinduced electron transfer from semiconductor quantum dots to metal oxide nanoparticles. Proc. Natl. Acad. Sci. U. S. A. 2011, 108, 29–34. 10.1073/pnas.1011972107. 21149685 PMC3017152

[ref254] Cánovas E.; Moll P.; Jensen S. A.; Gao Y.; Houtepen A. J.; Siebbeles L. D. A.; Kinge S.; Bonn M. Size-Dependent Electron Transfer from PbSe Quantum Dots to SnO2Monitored by Picosecond Terahertz Spectroscopy. Nano Lett. 2011, 11, 5234–5239. 10.1021/nl202550v. 22040524

[ref255] Klimka̅ns A.; Larsson S. Reorganization energies in benzene, naphthalene, and anthracene. Chem. Phys. 1994, 189, 25–31. 10.1016/0301-0104(94)80004-9.

[ref256] Amashukeli X.; Winkler J. R.; Gray H. B.; Gruhn N. E.; Lichtenberger D. L. Electron-Transfer Reorganization Energies of Isolated Organic Molecules†. J. Phys. Chem. A 2002, 106, 7593–7598. 10.1021/jp014148w.

[ref257] Marcus R.; Sutin N. Electron transfers in chemistry and biology. Biochim. Biophys. Acta 1985, 811, 265–322. 10.1016/0304-4173(85)90014-X.

[ref258] Cui S.-C.; Tachikawa T.; Fujitsuka M.; Majima T. Solvent-Polarity Dependence of Electron-Transfer Kinetics in a CdSe/ZnS Quantum Dot–Pyromellitimide Conjugate. J. Phys. Chem. C 2010, 114, 1217–1225. 10.1021/jp909579j.

[ref259] Hyun B. R.; Bartnik A. C.; Lee J. K.; Imoto H.; Sun L.; Choi J. J.; Chujo Y.; Hanrath T.; Ober C. K.; Wise F. W. Role of solvent dielectric properties on charge transfer from PbS nanocrystals to molecules. Nano Lett. 2010, 10, 318–323. 10.1021/nl903623n. 19968265

[ref260] Hyun B. R.; Bartnik A. C.; Sun L.; Hanrath T.; Wise F. W. Control of electron transfer from lead-salt nanocrystals to TiO(2). Nano Lett. 2011, 11, 2126–2132. 10.1021/nl200718w. 21506588

[ref261] Newton M. D. Quantum chemical probes of electron-transfer kinetics: the nature of donor-acceptor interactions. Chem. Rev. 1991, 91, 767–792. 10.1021/cr00005a007.

[ref262] Benniston A. C.; Harriman A. Charge on the move: how electron-transfer dynamics depend on molecular conformation. Chem. Soc. Rev. 2006, 35, 169–179. 10.1039/B503169A. 16444298

[ref263] Zhu H.; Song N.; Lian T. Controlling Charge Separation and Recombination Rates in CdSe/ZnS Type I Core–Shell Quantum Dots by Shell Thicknesses. J. Am. Chem. Soc. 2010, 132, 15038–15045. 10.1021/ja106710m. 20925344

[ref264] Ding T. X.; Olshansky J. H.; Leone S. R.; Alivisatos A. P. Efficiency of Hole Transfer from Photoexcited Quantum Dots to Covalently Linked Molecular Species. J. Am. Chem. Soc. 2015, 137, 2021–2029. 10.1021/ja512278a. 25591013

[ref265] Dworak L.; Matylitsky V. V.; Breus V. V.; Braun M.; Basché T.; Wachtveitl J. Ultrafast Charge Separation at the CdSe/CdS Core/Shell Quantum Dot/Methylviologen Interface: Implications for Nanocrystal Solar Cells. J. Phys. Chem. C 2011, 115, 3949–3955. 10.1021/jp111574w.

[ref266] Xu Z.; Hine C. R.; Maye M. M.; Meng Q.; Cotlet M. Shell thickness dependent photoinduced hole transfer in hybrid conjugated polymer/quantum dot nanocomposites: from ensemble to single hybrid level. ACS Nano 2012, 6, 4984–4992. 10.1021/nn300525b. 22686521

[ref267] Jiang Z.-J.; Kelley D. F. Effects of Inhomogeneous Shell Thickness in the Charge Transfer Dynamics of ZnTe/CdSe Nanocrystals. J. Phys. Chem. C 2012, 116, 12958–12968. 10.1021/jp303307r.

[ref268] Wang H.; McNellis E. R.; Kinge S.; Bonn M.; Canovas E. Tuning electron transfer rates through molecular bridges in quantum dot sensitized oxides. Nano Lett. 2013, 13, 5311–5315. 10.1021/nl402820v. 24093529

[ref269] Tagliazucchi M.; Tice D. B.; Sweeney C. M.; Morris-Cohen A. J.; Weiss E. A. Ligand-Controlled Rates of Photoinduced Electron Transfer in Hybrid CdSe Nanocrystal/Poly(viologen) Films. ACS Nano 2011, 5, 9907–9917. 10.1021/nn203683s. 22067227

[ref270] Morris-Cohen A. J.; Peterson M. D.; Frederick M. T.; Kamm J. M.; Weiss E. A. Evidence for a Through-Space Pathway for Electron Transfer from Quantum Dots to Carboxylate-Functionalized Viologens. J. Phys. Chem. Lett. 2012, 3, 2840–2844. 10.1021/jz301318m.

[ref271] Xu Z.; Cotlet M. Quantum dot-bridge-fullerene heterodimers with controlled photoinduced electron transfer. Angew. Chem. 2011, 50, 6079–6083. 10.1002/anie.201007270. 21560207

[ref272] Pu Y.-C.; Ma H.; Sajben N.; Xia G.; Zhang J.; Li Y.; Zhang J. Z. Dependence of Interfacial Charge Transfer on Bifunctional Aromatic Molecular Linkers in CdSe Quantum Dot Sensitized TiO2 Photoelectrodes. ACS Applied Energy Materials 2018, 1, 2907–2917. 10.1021/acsaem.8b00563.

[ref273] Dibbell R. S.; Watson D. F. Distance-Dependent Electron Transfer in Tethered Assemblies of CdS Quantum Dots and TiO_2_ Nanoparticles. J. Phys. Chem. C 2009, 113, 3139–3149. 10.1021/jp809269m.

[ref274] Xu B.; Tao N. J. Measurement of Single-Molecule Resistance by Repeated Formation of Molecular Junctions. Science 2003, 301, 1221–1223. 10.1126/science.1087481. 12947193

[ref275] Dibbell R. S.; Youker D. G.; Watson D. F. Excited-State Electron Transfer from CdS Quantum Dots to TiO2 Nanoparticles via Molecular Linkers with Phenylene Bridges. J. Phys. Chem. C 2009, 113, 18643–18651. 10.1021/jp9079469.

[ref276] Morris-Cohen A. J.; Aruda K. O.; Rasmussen A. M.; Canzi G.; Seideman T.; Kubiak C. P.; Weiss E. A. Controlling the rate of electron transfer between a quantum dot and a tri-ruthenium molecular cluster by tuning the chemistry of the interface. Phys. Chem. Chem. Phys. 2012, 14, 13794–13801. 10.1039/c2cp40827a. 22588225

[ref277] Hansen T.; Žídek K.; Zheng K.; Abdellah M.; Chábera P.; Persson P.; Pullerits T. Orbital Topology Controlling Charge Injection in Quantum-Dot-Sensitized Solar Cells. J. Phys. Chem. Lett. 2014, 5, 1157–1162. 10.1021/jz5001193. 26274464

[ref278] Kamisaka H.; Kilina S. V.; Yamashita K.; Prezhdo O. V. Ab Initio Study of Temperature and Pressure Dependence of Energy and Phonon-Induced Dephasing of Electronic Excitations in CdSe and PbSe Quantum Dots†. J. Phys. Chem. C 2008, 112, 7800–7808. 10.1021/jp710435q.

[ref279] Fu H.; Wang L.-W.; Zunger A. Applicability of the $\mathbf{k}\ensuremath{\cdot}\mathbf{p}$ method to the electronic structure of quantum dots. Phys. Rev. B 1998, 57, 9971–9987. 10.1103/PhysRevB.57.9971.

[ref280] Inamdar S. N.; Ingole P. P.; Haram S. K. Determination of Band Structure Parameters and the Quasi-Particle Gap of CdSe Quantum Dots by Cyclic Voltammetry. ChemPhysChem 2008, 9, 2574–2579. 10.1002/cphc.200800482. 18956405

[ref281] Hyun B.-R.; Zhong Y.-W.; Bartnik A. C.; Sun L.; Abruña H. D.; Wise F. W.; Goodreau J. D.; Matthews J. R.; Leslie T. M.; Borrelli N. F. Electron Injection from Colloidal PbS Quantum Dots into Titanium Dioxide Nanoparticles. ACS Nano 2008, 2, 2206–2212. 10.1021/nn800336b. 19206384

[ref282] Haram S. K.; Kshirsagar A.; Gujarathi Y. D.; Ingole P. P.; Nene O. A.; Markad G. B.; Nanavati S. P. Quantum Confinement in CdTe Quantum Dots: Investigation through Cyclic Voltammetry Supported by Density Functional Theory (DFT). J. Phys. Chem. C 2011, 115, 6243–6249. 10.1021/jp111463f.

[ref283] Ogawa S.; Hu K.; Fan F.-R. F.; Bard A. J. Photoelectrochemistry of Films of Quantum Size Lead Sulfide Particles Incorporated in Self-Assembled Monolayers on Gold. J. Phys. Chem. B 1997, 101, 5707–5711. 10.1021/jp970737j.

[ref284] Haram S. K.; Quinn B. M.; Bard A. J. Electrochemistry of CdS Nanoparticles: A Correlation between Optical and Electrochemical Band Gaps. J. Am. Chem. Soc. 2001, 123, 8860–8861. 10.1021/ja0158206. 11535097

[ref285] Jasieniak J.; Califano M.; Watkins S. E. Size-Dependent Valence and Conduction Band-Edge Energies of Semiconductor Nanocrystals. ACS Nano 2011, 5, 5888–5902. 10.1021/nn201681s. 21662980

[ref286] Miller J. R.; Calcaterra L. T.; Closs G. L. Intramolecular long-distance electron transfer in radical anions. The effects of free energy and solvent on the reaction rates. J. Am. Chem. Soc. 1984, 106, 3047–3049. 10.1021/ja00322a058.

[ref287] Huang J.; Stockwell D.; Huang Z.; Mohler D. L.; Lian T. Photoinduced Ultrafast Electron Transfer from CdSe Quantum Dots to Re-bipyridyl Complexes. J. Am. Chem. Soc. 2008, 130, 5632–5633. 10.1021/ja8003683. 18393497

[ref288] Gocalińska A.; Saba M.; Quochi F.; Marceddu M.; Szendrei K.; Gao J.; Loi M. A.; Yarema M.; Seyrkammer R.; Heiss W.; et al. Size-Dependent Electron Transfer from Colloidal PbS Nanocrystals to Fullerene. J. Phys. Chem. Lett. 2010, 1, 1149–1154. 10.1021/jz100116t.

[ref289] Scholz F.; Dworak L.; Matylitsky V. V.; Wachtveitl J. Ultrafast electron transfer from photoexcited CdSe quantum dots to methylviologen. ChemPhysChem 2011, 12, 2255–2259. 10.1002/cphc.201100120. 21726036

[ref290] El-Ballouli A. O.; Alarousu E.; Bernardi M.; Aly S. M.; Lagrow A. P.; Bakr O. M.; Mohammed O. F. Quantum confinement-tunable ultrafast charge transfer at the PbS quantum dot and phenyl-C(6)(1)-butyric acid methyl ester interface. J. Am. Chem. Soc. 2014, 136, 6952–6959. 10.1021/ja413254g. 24521255

[ref291] Olshansky J. H.; Ding T. X.; Lee Y. V.; Leone S. R.; Alivisatos A. P. Hole Transfer from Photoexcited Quantum Dots: The Relationship between Driving Force and Rate. J. Am. Chem. Soc. 2015, 137, 15567–15575. 10.1021/jacs.5b10856. 26597761

[ref292] Olshansky J. H.; Balan A. D.; Ding T. X.; Fu X.; Lee Y. V.; Alivisatos A. P. Temperature-Dependent Hole Transfer from Photoexcited Quantum Dots to Molecular Species: Evidence for Trap-Mediated Transfer. ACS Nano 2017, 11, 8346–8355. 10.1021/acsnano.7b03580. 28759718

[ref293] Žídek K.; Zheng K.; Ponseca C. S.; Messing M. E.; Wallenberg L. R.; Chábera P.; Abdellah M.; Sundström V.; Pullerits T. Electron Transfer in Quantum-Dot-Sensitized ZnO Nanowires: Ultrafast Time-Resolved Absorption and Terahertz Study. J. Am. Chem. Soc. 2012, 134, 12110–12117. 10.1021/ja3029679. 22730926

[ref294] Zheng K.; Žídek K.; Abdellah M.; Zhang W.; Chábera P.; Lenngren N.; Yartsev A.; Pullerits T. Ultrafast Charge Transfer from CdSe Quantum Dots to p-Type NiO: Hole Injection vs Hole Trapping. J. Phys. Chem. C 2014, 118, 18462–18471. 10.1021/jp506963q.

[ref295] Wang J.; Ding T.; Gao K.; Wang L.; Zhou P.; Wu K. Marcus inverted region of charge transfer from low-dimensional semiconductor materials. Nat. Commun. 2021, 12, 6333 10.1038/s41467-021-26705-x. 34732730 PMC8566515

[ref296] Tisdale W. A.; Zhu X.-Y. Artificial atoms on semiconductor surfaces. Proc. Natl. Acad. Sci. U. S. A. 2011, 108, 965–970. 10.1073/pnas.1006665107. 21097704 PMC3024701

[ref297] Sakata T.; Hashimoto K.; Hiramoto M. New aspects of electron transfer on semiconductor surface: dye-sensitization system. J. Phys. Chem. 1990, 94, 3040–3045. 10.1021/j100370a056.

[ref298] She C. X.; Anderson N. A.; Guo J. C.; Liu F.; Goh W. H.; Chen D. T.; Mohler D. L.; Tian Z. Q.; Hupp J. T.; Lian T. Q. pH-dependent electron transfer from re-bipyridyl complexes to metal oxide nanocrystalline thin films. J. Phys. Chem. B 2005, 109, 19345–19355. 10.1021/jp053948u. 16853498

[ref299] Asbury J. B.; Hao E.; Wang Y. Q.; Ghosh H. N.; Lian T. Q. Ultrafast electron transfer dynamics from molecular adsorbates to semiconductor nanocrystalline thin films. J. Phys. Chem. B 2001, 105, 4545–4557. 10.1021/jp003485m.

[ref300] Rabouw F. T.; Kamp M.; van Dijk-Moes R. J. A.; Gamelin D. R.; Koenderink A. F.; Meijerink A.; Vanmaekelbergh D. Delayed Exciton Emission and Its Relation to Blinking in CdSe Quantum Dots. Nano Lett. 2015, 15, 7718–7725. 10.1021/acs.nanolett.5b03818. 26496661

[ref301] Krause M. M.; Kambhampati P. Linking surface chemistry to optical properties of semiconductor nanocrystals. Phys. Chem. Chem. Phys. 2015, 17, 18882–18894. 10.1039/C5CP02173A. 26130059

[ref302] Wise F. W. Lead Salt Quantum Dots: the Limit of Strong Quantum Confinement. Acc. Chem. Res. 2000, 33, 773–780. 10.1021/ar970220q. 11087314

[ref303] Tisdale W. A.; Williams K. J.; Timp B. A.; Norris D. J.; Aydil E. S.; Zhu X.-Y. Hot-Electron Transfer from Semiconductor Nanocrystals. Science 2010, 328, 1543–1547. 10.1126/science.1185509. 20558714

[ref304] Anderson P. W. Localized Magnetic States in Metals. Phys. Rev. 1961, 124, 41–53. 10.1103/PhysRev.124.41.

[ref305] Newns D. M. Self-Consistent Model of Hydrogen Chemisorption. Phys. Rev. 1969, 178, 1123–1135. 10.1103/PhysRev.178.1123.

[ref306] Long R.; Prezhdo O. V. Ab initio nonadiabatic molecular dynamics of the ultrafast electron injection from a PbSe quantum dot into the TiO2 surface. J. Am. Chem. Soc. 2011, 133, 19240–19249. 10.1021/ja2085806. 22007727

[ref307] Wang H. I.; Infante I.; Brinck S. t.; Cánovas E.; Bonn M. Efficient Hot Electron Transfer in Quantum Dot-Sensitized Mesoporous Oxides at Room Temperature. Nano Lett. 2018, 18, 5111–5115. 10.1021/acs.nanolett.8b01981. 30039708

[ref308] Cooney R. R.; Sewall S. L.; Dias E. A.; Sagar D. M.; Anderson K. E. H.; Kambhampati P. Unified picture of electron and hole relaxation pathways in semiconductor quantum dots. Phys. Rev. B 2007, 75, 245311 10.1103/PhysRevB.75.245311.

[ref309] Kambhampati P. Hot Exciton Relaxation Dynamics in Semiconductor Quantum Dots: Radiationless Transitions on the Nanoscale. J. Phys. Chem. C 2011, 115, 22089–22109. 10.1021/jp2058673.

[ref310] Grimaldi G.; Crisp R. W.; ten Brinck S.; Zapata F.; van Ouwendorp M.; Renaud N.; Kirkwood N.; Evers W. H.; Kinge S.; Infante I.; et al. Hot-electron transfer in quantum-dot heterojunction films. Nat. Commun. 2018, 9, 2310 10.1038/s41467-018-04623-9. 29899361 PMC5998019

[ref311] Li M.; Bhaumik S.; Goh T. W.; Kumar M. S.; Yantara N.; Grätzel M.; Mhaisalkar S.; Mathews N.; Sum T. C. Slow cooling and highly efficient extraction of hot carriers in colloidal perovskite nanocrystals. Nat. Commun. 2017, 8, 14350 10.1038/ncomms14350. 28176882 PMC5309769

[ref312] Sarkar S.; Ravi V. K.; Banerjee S.; Yettapu G. R.; Markad G. B.; Nag A.; Mandal P. Terahertz Spectroscopic Probe of Hot Electron and Hole Transfer from Colloidal CsPbBr3 Perovskite Nanocrystals. Nano Lett. 2017, 17, 5402–5407. 10.1021/acs.nanolett.7b02003. 28831807

[ref313] Diroll B. T.; Fedin I.; Darancet P.; Talapin D. V.; Schaller R. D. Surface-Area-Dependent Electron Transfer Between Isoenergetic 2D Quantum Wells and a Molecular Acceptor. J. Am. Chem. Soc. 2016, 138, 11109–11112. 10.1021/jacs.6b06572. 27518932

[ref314] Okuhata T.; Tamai N. Face-Dependent Electron Transfer in CdSe Nanoplatelet–Methyl Viologen Complexes. J. Phys. Chem. C 2016, 120, 17052–17059. 10.1021/acs.jpcc.6b04765.

[ref315] Franceschetti A.; An J. M.; Zunger A. Impact ionization can explain carrier multiplication in PbSe quantum dots. Nano Lett. 2006, 6, 2191–2195. 10.1021/nl0612401. 17034081

[ref316] Shabaev A.; Efros A. L.; Nozik A. J. Multiexciton generation by a single photon in nanocrystals. Nano Lett. 2006, 6, 2856–2863. 10.1021/nl062059v. 17163719

[ref317] Schaller R. D.; Agranovich V. M.; Klimov V. I. High-efficiency carrier multiplication through direct photogeneration of multi-excitons via virtual single-exciton states. Nat. Phys. 2005, 1, 189–194. 10.1038/nphys151.

[ref318] Prezhdo O. V. Multiple excitons and the electron–phonon bottleneck in semiconductor quantum dots: An ab initio perspective. Chem. Phys. Lett. 2008, 460, 1–9. 10.1016/j.cplett.2008.03.099.

[ref319] Dutta N. K.; Nelson R. J. The case for Auger recombination in In1–xGaxAsyP1–y. J. Appl. Phys. 1982, 53, 74–92. 10.1063/1.329942.

[ref320] Sun J.; Yu W.; Usman A.; Isimjan T. T.; Dgobbo S.; Alarousu E.; Takanabe K.; Mohammed O. F. Generation of Multiple Excitons in Ag2S Quantum Dots: Single High-Energy versus Multiple-Photon Excitation. J. Phys. Chem. Lett. 2014, 5, 659–665. 10.1021/jz5000512. 26270833

[ref321] Zhu H. M.; Lian T. Q. Enhanced Multiple Exciton Dissociation from CdSe Quantum Rods: The Effect of Nanocrystal Shape. J. Am. Chem. Soc. 2012, 134, 11289–11297. 10.1021/ja304724u. 22702343

[ref322] Zhu H. M.; Song N. H.; Rodriguez-Cordoba W.; Lian T. Q. Wave Function Engineering for Efficient Extraction of up to Nineteen Electrons from One CdSe/CdS Quasi-Type II Quantum Dot. J. Am. Chem. Soc. 2012, 134, 4250–4257. 10.1021/ja210312s. 22329340

[ref323] Wang F.; Dukovic G.; Knoesel E.; Brus L. E.; Heinz T. F. Observation of rapid Auger recombination in optically excited semiconducting carbon nanotubes. Phys. Rev. B 2004, 70, 241403 10.1103/PhysRevB.70.241403.

[ref324] Htoon H.; Hollingsworth J. A.; Dickerson R.; Klimov V. I. Effect of Zero- to One-Dimensional Transformation on Multiparticle Auger Recombination in Semiconductor Quantum Rods. Phys. Rev. Lett. 2003, 91, 227401 10.1103/PhysRevLett.91.227401. 14683270

[ref325] Huang J.; Huang Z. Q.; Yang Y.; Zhu H. M.; Lian T. Q. Multiple Exciton Dissociation in CdSe Quantum Dots by Ultrafast Electron Transfer to Adsorbed Methylene Blue. J. Am. Chem. Soc. 2010, 132, 4858–4864. 10.1021/ja100106z. 20218563

[ref326] Ueda A.; Tayagaki T.; Kanemitsu Y. Dynamics of Quantized Auger Recombination in CdSe Nanocrystals Studied by Femtosecond Intraband Pump-Probe Spectroscopy. J. Phys. Soc. Jpn. 2009, 78, 083706 10.1143/JPSJ.78.083706.

[ref327] Padilha L. A.; Stewart J. T.; Sandberg R. L.; Bae W. K.; Koh W.-K.; Pietryga J. M.; Klimov V. I. Aspect Ratio Dependence of Auger Recombination and Carrier Multiplication in PbSe Nanorods. Nano Lett. 2013, 13, 1092–1099. 10.1021/nl304426y. 23360573

[ref328] Robel I.; Gresback R.; Kortshagen U.; Schaller R. D.; Klimov V. I. Universal Size-Dependent Trend in Auger Recombination in Direct-Gap and Indirect-Gap Semiconductor Nanocrystals. Phys. Rev. Lett. 2009, 102, 177404 10.1103/PhysRevLett.102.177404. 19518831

[ref329] Li Q.; Lian T. Area- and Thickness-Dependent Biexciton Auger Recombination in Colloidal CdSe Nanoplatelets: Breaking the “Universal Volume Scaling Law. Nano Lett. 2017, 17, 3152–3158. 10.1021/acs.nanolett.7b00587. 28418671

[ref330] Padilha L. A.; Stewart J. T.; Sandberg R. L.; Bae W. K.; Koh W. K.; Pietryga J. M.; Klimov V. I. Carrier Multiplication in Semiconductor Nanocrystals: Influence of Size, Shape, and Composition. Acc. Chem. Res. 2013, 46, 1261–1269. 10.1021/ar300228x. 23530867

[ref331] Schaller R. D.; Klimov V. I. High efficiency carrier multiplication in PbSe nanocrystals: Implications for solar energy conversion. Phys. Rev. Lett. 2004, 92, 186601 10.1103/PhysRevLett.92.186601. 15169518

[ref332] Schaller R. D.; Pietryga J. M.; Klimov V. I. Carrier multiplication in InAs nanocrystal quantum dots with an onset defined by the energy conservation limit. Nano Lett. 2007, 7, 3469–3476. 10.1021/nl072046x. 17967043

[ref333] Barzykin A. V.; Tachiya M. Stochastic models of charge carrier dynamics in semiconducting nanosystems. J. Phys-Condens Mat 2007, 19, 065105 10.1088/0953-8984/19/6/065105.

[ref334] Pal S.; Casanova D.; Prezhdo O. V. Effect of Aspect Ratio on Multiparticle Auger Recombination in Single-Walled Carbon Nanotubes: Time Domain Atomistic Simulation. Nano Lett. 2018, 18, 58–63. 10.1021/acs.nanolett.7b03150. 29190106

[ref335] Aerts M.; Spoor F. C. M.; Grozema F. C.; Houtepen A. J.; Schins J. M.; Siebbeles L. D. A. Cooling and Auger Recombination of Charges in PbSe Nanorods: Crossover from Cubic to Bimolecular Decay. Nano Lett. 2013, 13, 4380–4386. 10.1021/nl402223q. 23968451

[ref336] Wang F.; Wu Y.; Hybertsen M. S.; Heinz T. F. Auger recombination of excitons in one-dimensional systems. Phys. Rev. B 2006, 73, 245424 10.1103/PhysRevB.73.245424.

[ref337] Li Q.; Yang Y.; Que W.; Lian T. Size- and Morphology-Dependent Auger Recombination in CsPbBr3 Perovskite Two-Dimensional Nanoplatelets and One-Dimensional Nanorods. Nano Lett. 2019, 19, 5620–5627. 10.1021/acs.nanolett.9b02145. 31244208

[ref338] Dyakonov M. I.; Kachorovskii V. Y. Nonthreshold Auger recombination in quantum wells. Phys. Rev. B 1994, 49, 17130–17138. 10.1103/PhysRevB.49.17130. 10010891

[ref339] Grim J. Q.; Christodoulou S.; Di Stasio F.; Krahne R.; Cingolani R.; Manna L.; Moreels I. Continuous-wave biexciton lasing at room temperature using solution-processed quantum wells. Nat. Nanotechnol. 2014, 9, 891–895. 10.1038/nnano.2014.213. 25282045

[ref340] She C. X.; Fedin I.; Dolzhnikov D. S.; Demortiere A.; Schaller R. D.; Pelton M.; Talapin D. V. Low-Threshold Stimulated Emission Using Colloidal Quantum Wells. Nano Lett. 2014, 14, 2772–2777. 10.1021/nl500775p. 24773282

[ref341] Pelton M.; Andrews J. J.; Fedin I.; Talapin D. V.; Leng H.; O’Leary S. K. Nonmonotonic Dependence of Auger Recombination Rate on Shell Thickness for CdSe/CdS Core/Shell Nanoplatelets. Nano Lett. 2017, 17, 6900–6906. 10.1021/acs.nanolett.7b03294. 28994296

[ref342] Vaxenburg R.; Rodina A.; Shabaev A.; Lifshitz E.; Efros A. L. Nonradiative Auger Recombination in Semiconductor Nanocrystals. Nano Lett. 2015, 15, 2092–2098. 10.1021/nl504987h. 25693512

[ref343] Vaxenburg R.; Lifshitz E.; Efros A. Suppression of Auger-stimulated efficiency droop in nitride-based light emitting diodes. Appl. Phys. Lett. 2013, 102, 031120 10.1063/1.4789364.

[ref344] Climente J. I.; Movilla J. L.; Planelles J. Auger Recombination Suppression in Nanocrystals with Asymmetric Electron-Hole Confinement. Small 2012, 8, 754–759. 10.1002/smll.201101740. 22223514

[ref345] Cragg G. E.; Efros A. L. Suppression of Auger Processes in Confined Structures. Nano Lett. 2010, 10, 313–317. 10.1021/nl903592h. 20017564

[ref346] Chepic D. I.; Efros A. L.; Ekimov A. I.; Ivanov M. G.; Kharchenko V. A.; Kudriavtsev I. A.; Yazeva T. V. Auger Ionization of Semiconductor Quantum Drops in a Glass Matrix. J. Lumin. 1990, 47, 113–127. 10.1016/0022-2313(90)90007-X.

[ref347] Efros A. L.; Nesbitt D. J. Origin and control of blinking in quantum dots. Nat. Nano 2016, 11, 661–671. 10.1038/nnano.2016.140. 27485584

[ref348] Bae W. K.; Padilha L. A.; Park Y.-S.; McDaniel H.; Robel I.; Pietryga J. M.; Klimov V. I. Controlled Alloying of the Core–Shell Interface in CdSe/CdS Quantum Dots for Suppression of Auger Recombination. ACS Nano 2013, 7, 3411–3419. 10.1021/nn4002825. 23521208

[ref349] Wang X.; Ren X.; Kahen K.; Hahn M. A.; Rajeswaran M.; Maccagnano-Zacher S.; Silcox J.; Cragg G. E.; Efros A. L.; Krauss T. D. Non-blinking semiconductor nanocrystals. Nature 2009, 459, 686–689. 10.1038/nature08072. 19430463

[ref350] Lim J.; Park Y.-S.; Klimov V. I. Optical gain in colloidal quantum dots achieved with direct-current electrical pumping. Nat. Mater. 2018, 17, 42–49. 10.1038/nmat5011. 29180770

[ref351] Park Y.-S.; Lim J.; Makarov N. S.; Klimov V. I. Effect of Interfacial Alloying versus “Volume Scaling” on Auger Recombination in Compositionally Graded Semiconductor Quantum Dots. Nano Lett. 2017, 17, 5607–5613. 10.1021/acs.nanolett.7b02438. 28776995

[ref352] Ellingson R. J.; Beard M. C.; Johnson J. C.; Yu P. R.; Micic O. I.; Nozik A. J.; Shabaev A.; Efros A. L. Highly efficient multiple exciton generation in colloidal PbSe and PbS quantum dots. Nano Lett. 2005, 5, 865–871. 10.1021/nl0502672. 15884885

[ref353] Nootz G.; Padilha L. A.; Levina L.; Sukhovatkin V.; Webster S.; Brzozowski L.; Sargent E. H.; Hagan D. J.; Van Stryland E. W. Size dependence of carrier dynamics and carrier multiplication in PbS quantum dots. Phys. Rev. B 2011, 83, 155302 10.1103/PhysRevB.83.155302.

[ref354] Ji M. B.; Park S.; Connor S. T.; Mokari T.; Cui Y.; Gaffney K. J. Efficient Multiple Exciton Generation Observed in Colloidal PbSe Quantum Dots with Temporally and Spectrally Resolved Intraband Excitation. Nano Lett. 2009, 9, 1217–1222. 10.1021/nl900103f. 19226125

[ref355] Murphy J. E.; Beard M. C.; Norman A. G.; Ahrenkiel S. P.; Johnson J. C.; Yu P.; Mićić O. I.; Ellingson R. J.; Nozik A. J. PbTe Colloidal Nanocrystals: Synthesis, Characterization, and Multiple Exciton Generation. J. Am. Chem. Soc. 2006, 128, 3241–3247. 10.1021/ja0574973. 16522105

[ref356] Pijpers J. J. H.; Hendry E.; Milder M. T. W.; Fanciulli R.; Savolainen J.; Herek J. L.; Vanmaekelbergh D.; Ruhman S.; Mocatta D.; Oron D.; et al. Carrier Multiplication and Its Reduction by Photodoping in Colloidal InAs Quantum Dots. J. Phys. Chem. C 2007, 111, 4146–4152. 10.1021/jp066709v.

[ref357] Beard M. C.; Knutsen K. P.; Yu P.; Luther J. M.; Song Q.; Metzger W. K.; Ellingson R. J.; Nozik A. J. Multiple Exciton Generation in Colloidal Silicon Nanocrystals. Nano Lett. 2007, 7, 2506–2512. 10.1021/nl071486l. 17645368

[ref358] Stubbs S. K.; Hardman S. J. O.; Graham D. M.; Spencer B. F.; Flavell W. R.; Glarvey P.; Masala O.; Pickett N. L.; Binks D. J. Efficient carrier multiplication in InP nanoparticles. Phys. Rev. B 2010, 81, 081303 10.1103/PhysRevB.81.081303.

[ref359] Schaller R. D.; Petruska M. A.; Klimov V. I. Effect of electronic structure on carrier multiplication efficiency: Comparative study of PbSe and CdSe nanocrystals. Appl. Phys. Lett. 2005, 87, 253102–253103. 10.1063/1.2142092.

[ref360] Schaller R. D.; Sykora M.; Jeong S.; Klimov V. I. High-efficiency carrier multiplication and ultrafast charge separation in semiconductor nanocrystals studied via time-resolved photoluminescence. J. Phys. Chem. B 2006, 110, 25332–25338. 10.1021/jp065282p. 17165979

[ref361] Kobayashi Y.; Udagawa T.; Tamai N. Carrier Multiplication in CdTe Quantum Dots by Single-photon Timing Spectroscopy. Chem. Lett. 2009, 38, 830–831. 10.1246/cl.2009.830.

[ref362] Gachet D.; Avidan A.; Pinkas I.; Oron D. An Upper Bound to Carrier Multiplication Efficiency in Type II Colloidal Quantum Dots. Nano Lett. 2010, 10, 164–170. 10.1021/nl903172f. 19911830

[ref363] Trinh M. T.; Polak L.; Schins J. M.; Houtepen A. J.; Vaxenburg R.; Maikov G. I.; Grinbom G.; Midgett A. G.; Luther J. M.; Beard M. C.; et al. Anomalous Independence of Multiple Exciton Generation on Different Group IV–VI Quantum Dot Architectures. Nano Lett. 2011, 11, 1623–1629. 10.1021/nl200014g. 21348493

[ref364] Stolle C. J.; Harvey T. B.; Pernik D. R.; Hibbert J. I.; Du J.; Rhee D. J.; Akhavan V. A.; Schaller R. D.; Korgel B. A. Multiexciton Solar Cells of CuInSe2 Nanocrystals. J. Phys. Chem. Lett. 2014, 5, 304–309. 10.1021/jz402596v. 26270704

[ref365] Wang S.; Khafizov M.; Tu X.; Zheng M.; Krauss T. D. Multiple Exciton Generation in Single-Walled Carbon Nanotubes. Nano Lett. 2010, 10, 2381–2386. 10.1021/nl100343j. 20507082

[ref366] Nair G.; Bawendi M. G. Carrier multiplication yields of CdSe and CdTe nanocrystals by transient photoluminescence spectroscopy. Phys. Rev. B 2007, 76, 081304 10.1103/PhysRevB.76.081304.

[ref367] Ben-Lulu M.; Mocatta D.; Bonn M.; Banin U.; Ruhman S. On the Absence of Detectable Carrier Multiplication in a Transient Absorption Study of InAs/CdSe/ZnSe Core/Shell1/Shell2 Quantum Dots. Nano Lett. 2008, 8, 1207–1211. 10.1021/nl080199u. 18341299

[ref368] Pijpers J. J. H.; Ulbricht R.; Tielrooij K. J.; Osherov A.; Golan Y.; Delerue C.; Allan G.; Bonn M. Assessment of carrier-multiplication efficiency in bulk PbSe and PbS. Nat. Phys. 2009, 5, 811–814. 10.1038/nphys1393.

[ref369] Beard M. C.; Midgett A. G.; Hanna M. C.; Luther J. M.; Hughes B. K.; Nozik A. J. Comparing Multiple Exciton Generation in Quantum Dots To Impact Ionization in Bulk Semiconductors: Implications for Enhancement of Solar Energy Conversion. Nano Lett. 2010, 10, 3019–3027. 10.1021/nl101490z. 20698615

[ref370] Nair G.; Geyer S. M.; Chang L.-Y.; Bawendi M. G. Carrier multiplication yields in PbS and PbSe nanocrystals measured by transient photoluminescence. Phys. Rev. B 2008, 78, 125325 10.1103/PhysRevB.78.125325.

[ref371] Trinh M. T.; Houtepen A. J.; Schins J. M.; Hanrath T.; Piris J.; Knulst W.; Goossens A. P. L. M.; Siebbeles L. D. A. In spite of recent doubts carrier multiplication does occur in PbSe nanocrystals. Nano Lett. 2008, 8, 1713–1718. 10.1021/nl0807225. 18489170

[ref372] Tyagi P.; Kambhampati P. False multiple exciton recombination and multiple exciton generation signals in semiconductor quantum dots arise from surface charge trapping. J. Chem. Phys. 2011, 134, 094706–094710. 10.1063/1.3561063. 21384996

[ref373] McGuire J. A.; Sykora M.; Joo J.; Pietryga J. M.; Klimov V. I. Apparent Versus True Carrier Multiplication Yields in Semiconductor Nanocrystals. Nano Lett. 2010, 10, 2049–2057. 10.1021/nl100177c. 20459066

[ref374] Gdor I.; Sachs H.; Roitblat A.; Strasfeld D. B.; Bawendi M. G.; Ruhman S. Exploring Exciton Relaxation and Multiexciton Generation in PbSe Nanocrystals Using Hyperspectral Near-IR Probing. ACS Nano 2012, 6, 3269–3277. 10.1021/nn300184n. 22390473

[ref375] Kang I.; Wise F. W. Electronic structure and optical properties of PbS and PbSe quantum dots. J. Opt Soc. Am. B 1997, 14, 1632–1646. 10.1364/JOSAB.14.001632.

[ref376] Cunningham P. D.; Boercker J. E.; Foos E. E.; Lumb M. P.; Smith A. R.; Tischler J. G.; Melinger J. S. Enhanced Multiple Exciton Generation in Quasi-One-Dimensional Semiconductors. Nano Lett. 2011, 11, 3476–3481. 10.1021/nl202014a. 21766838

[ref377] Cirloganu C. M.; Padilha L. A.; Lin Q.; Makarov N. S.; Velizhanin K. A.; Luo H.; Robel I.; Pietryga J. M.; Klimov V. I. Enhanced carrier multiplication in engineered quasi-type-II quantum dots. Nat. Commun. 2014, 5, 4148 10.1038/ncomms5148. 24938462 PMC4083434

[ref378] Kubie L.; King L. A.; Kern M. E.; Murphy J. R.; Kattel S.; Yang Q.; Stecher J. T.; Rice W. D.; Parkinson B. A. Synthesis and Characterization of Ultrathin Silver Sulfide Nanoplatelets. ACS Nano 2017, 11, 8471–8477. 10.1021/acsnano.7b04280. 28752997

[ref379] Aerts M.; Bielewicz T.; Klinke C.; Grozema F. C.; Houtepen A. J.; Schins J. M.; Siebbeles L. D. A. Highly efficient carrier multiplication in PbS nanosheets. Nat. Commun. 2014, 5, 3789 10.1038/ncomms4789. 24781188 PMC4015322

[ref380] de Weerd C.; Gomez L.; Capretti A.; Lebrun D. M.; Matsubara E.; Lin J.; Ashida M.; Spoor F. C. M.; Siebbeles L. D. A.; Houtepen A. J.; et al. Efficient carrier multiplication in CsPbI3 perovskite nanocrystals. Nat. Commun. 2018, 9, 4199 10.1038/s41467-018-06721-0. 30305623 PMC6180104

[ref381] Li M.; Begum R.; Fu J.; Xu Q.; Koh T. M.; Veldhuis S. A.; Grätzel M.; Mathews N.; Mhaisalkar S.; Sum T. C. Low threshold and efficient multiple exciton generation in halide perovskite nanocrystals. Nat. Commun. 2018, 9, 4197 10.1038/s41467-018-06596-1. 30305633 PMC6180109

[ref382] Manzi A.; Tong Y.; Feucht J.; Yao E.-P.; Polavarapu L.; Urban A. S.; Feldmann J. Resonantly enhanced multiple exciton generation through below-band-gap multi-photon absorption in perovskite nanocrystals. Nat. Commun. 2018, 9, 1518 10.1038/s41467-018-03965-8. 29666394 PMC5904181

[ref383] Cong M.; Yang B.; Chen J.; Hong F.; Yang S.; Deng W.; Han K. Carrier Multiplication and Hot-Carrier Cooling Dynamics in Quantum-Confined CsPbI3 Perovskite Nanocrystals. J. Phys. Chem. Lett. 2020, 11, 1921–1926. 10.1021/acs.jpclett.0c00188. 32079404

[ref384] Timmerman D.; Matsubara E.; Gomez L.; Ashida M.; Gregorkiewicz T.; Fujiwara Y. Direct Visualization and Determination of the Multiple Exciton Generation Rate. ACS Omega 2020, 5, 21506–21512. 10.1021/acsomega.0c02067. 32905445 PMC7469370

[ref385] Chen Y.; Yin J.; Wei Q.; Wang C.; Wang X.; Ren H.; Yu S. F.; Bakr O. M.; Mohammed O. F.; Li M. Multiple exciton generation in tin–lead halide perovskite nanocrystals for photocurrent quantum efficiency enhancement. Nat. Photonics 2022, 16, 485–490. 10.1038/s41566-022-01006-x.

[ref386] Wang S.; Feng S.; Li R.; Jin J.; Wu J.; Zheng W.; Xia Z.; Chen X.; Ling Q.; Lin Z. Multiexciton Generation from a 2D Organic–Inorganic Hybrid Perovskite with Nearly 200% Quantum Yield of Red Phosphorescence. Adv. Mater. 2023, 35, 2211992 10.1002/adma.202211992. 36807946

[ref387] Pattantyus-Abraham A. G.; Kramer I. J.; Barkhouse A. R.; Wang X. H.; Konstantatos G.; Debnath R.; Levina L.; Raabe I.; Nazeeruddin M. K.; Gratzel M.; et al. Depleted-Heterojunction Colloidal Quantum Dot Solar Cells. ACS Nano 2010, 4, 3374–3380. 10.1021/nn100335g. 20496882

[ref388] Luther J. M.; Law M.; Beard M. C.; Song Q.; Reese M. O.; Ellingson R. J.; Nozik A. J. Schottky Solar Cells Based on Colloidal Nanocrystal Films. Nano Lett. 2008, 8, 3488–3492. 10.1021/nl802476m. 18729414

[ref389] Chang L.-Y.; Lunt R. R.; Brown P. R.; Bulović V.; Bawendi M. G. Low-Temperature Solution-Processed Solar Cells Based on PbS Colloidal Quantum Dot/CdS Heterojunctions. Nano Lett. 2013, 13, 994–999. 10.1021/nl3041417. 23406331

[ref390] Sukhovatkin V.; Hinds S.; Brzozowski L.; Sargent E. H. Colloidal Quantum-Dot Photodetectors Exploiting Multiexciton Generation. Science 2009, 324, 1542–1544. 10.1126/science.1173812. 19541992

[ref391] Luther J. M.; Beard M. C.; Song Q.; Law M.; Ellingson R. J.; Nozik A. J. Multiple Exciton Generation in Films of Electronically Coupled PbSe Quantum Dots. Nano Lett. 2007, 7, 1779–1784. 10.1021/nl0708617. 17530913

[ref392] Beard M. C.; Midgett A. G.; Law M.; Semonin O. E.; Ellingson R. J.; Nozik A. J. Variations in the Quantum Efficiency of Multiple Exciton Generation for a Series of Chemically Treated PbSe Nanocrystal Films. Nano Lett. 2009, 9, 836–845. 10.1021/nl803600v. 19170560

[ref393] Sandeep C. S. S.; Cate S. t.; Schins J. M.; Savenije T. J.; Liu Y.; Law M.; Kinge S.; Houtepen A. J.; Siebbeles L. D. A. High charge-carrier mobility enables exploitation of carrier multiplication in quantum-dot films. Nat. Commun. 2013, 4, 2360 10.1038/ncomms3360. 23974282 PMC3759061

[ref394] Kulkarni A.; Evers W. H.; Tomić S.; Beard M. C.; Vanmaekelbergh D.; Siebbeles L. D. A. Efficient Steplike Carrier Multiplication in Percolative Networks of Epitaxially Connected PbSe Nanocrystals. ACS Nano 2018, 12, 378–384. 10.1021/acsnano.7b06511. 29241008 PMC6150730

[ref395] Hilczer M.; Tachiya M. Stochastic Approach to Charge Separation in Multiexcited Quantum Dots. J. Phys. Chem. C 2009, 113, 18451–18454. 10.1021/jp907969d.

[ref396] Matylitsky V. V.; Dworak L.; Breus V. V.; Basche T.; Wachtveitl J. Ultrafast Charge Separation in Multiexcited CdSe Quantum Dots Mediated by Adsorbed Electron Acceptors. J. Am. Chem. Soc. 2009, 131, 2424 10.1021/ja808084y. 19191491

[ref397] Liu Y.; Cullen D. A.; Lian T. Slow Auger Recombination of Trapped Excitons Enables Efficient Multiple Electron Transfer in CdS–Pt Nanorod Heterostructures. J. Am. Chem. Soc. 2021, 143, 20264–20273. 10.1021/jacs.1c09125. 34797980

[ref398] Sambur J. B.; Novet T.; Parkinson B. A. Multiple Exciton Collection in a Sensitized Photovoltaic System. Science 2010, 330, 63–66. 10.1126/science.1191462. 20929804

[ref399] Gao J.; Fidler A. F.; Klimov V. I. Carrier multiplication detected through transient photocurrent in device-grade films of lead selenide quantum dots. Nat. Commun. 2015, 6, 8185 10.1038/ncomms9185. 26345390 PMC4569798

[ref400] Kodaimati M. S.; McClelland K. P.; He C.; Lian S.; Jiang Y.; Zhang Z.; Weiss E. A. Viewpoint: Challenges in Colloidal Photocatalysis and Some Strategies for Addressing Them. Inorg. Chem. 2018, 57, 3659–3670. 10.1021/acs.inorgchem.7b03182. 29561594

[ref401] Weiss E. A. Designing the Surfaces of Semiconductor Quantum Dots for Colloidal Photocatalysis. ACS Energy Letters 2017, 2, 1005–1013. 10.1021/acsenergylett.7b00061.

[ref402] Moroz P.; Boddy A.; Zamkov M. Challenges and Prospects of Photocatalytic Applications Utilizing Semiconductor Nanocrystals. Frontiers in Chemistry 2018, 6, 353 10.3389/fchem.2018.00353. 30159309 PMC6103974

[ref403] Zhao J.; Holmes M. A.; Osterloh F. E. Quantum Confinement Controls Photocatalysis: A Free Energy Analysis for Photocatalytic Proton Reduction at CdSe Nanocrystals. ACS Nano 2013, 7, 4316–4325. 10.1021/nn400826h. 23590186

[ref404] Holmes M. A.; Townsend T. K.; Osterloh F. E. Quantum confinement controlled photocatalytic water splitting by suspended CdSe nanocrystals. Chem. Commun. 2012, 48, 371–373. 10.1039/C1CC16082F. 22083249

[ref405] Dempsey J. L.; Brunschwig B. S.; Winkler J. R.; Gray H. B. Hydrogen Evolution Catalyzed by Cobaloximes. Acc. Chem. Res. 2009, 42, 1995–2004. 10.1021/ar900253e. 19928840

[ref406] Fihri A.; Artero V.; Razavet M.; Baffert C.; Leibl W.; Fontecave M. Cobaloxime-Based Photocatalytic Devices for Hydrogen Production. Angew. Chem. 2008, 120, 574–577. 10.1002/ange.200702953. 18095368

[ref407] Razavet M.; Artero V.; Fontecave M. Proton Electroreduction Catalyzed by Cobaloximes: Functional Models for Hydrogenases. Inorg. Chem. 2005, 44, 4786–4795. 10.1021/ic050167z. 15962987

[ref408] Du P.; Schneider J.; Luo G.; Brennessel W. W.; Eisenberg R. Visible Light-Driven Hydrogen Production from Aqueous Protons Catalyzed by Molecular Cobaloxime Catalysts. Inorg. Chem. 2009, 48, 4952–4962. 10.1021/ic900389z. 19397296

[ref409] Huang J.; Mulfort K. L.; Du P.; Chen L. X. Photodriven Charge Separation Dynamics in CdSe/ZnS Core/Shell Quantum Dot/Cobaloxime Hybrid for Efficient Hydrogen Production. J. Am. Chem. Soc. 2012, 134, 16472–16475. 10.1021/ja3062584. 22989083

[ref410] Gimbert-Suriñach C.; Albero J.; Stoll T.; Fortage J.; Collomb M.-N.; Deronzier A.; Palomares E.; Llobet A. Efficient and Limiting Reactions in Aqueous Light-Induced Hydrogen Evolution Systems using Molecular Catalysts and Quantum Dots. J. Am. Chem. Soc. 2014, 136, 7655–7661. 10.1021/ja501489h. 24799030

[ref411] Gross M. A.; Reynal A.; Durrant J. R.; Reisner E. Versatile Photocatalytic Systems for H2 Generation in Water Based on an Efficient DuBois-Type Nickel Catalyst. J. Am. Chem. Soc. 2014, 136, 356–366. 10.1021/ja410592d. 24320740 PMC3901378

[ref412] Martindale B. C. M.; Hutton G. A. M.; Caputo C. A.; Reisner E. Solar Hydrogen Production Using Carbon Quantum Dots and a Molecular Nickel Catalyst. J. Am. Chem. Soc. 2015, 137, 6018–6025. 10.1021/jacs.5b01650. 25864839

[ref413] Han Z.; Qiu F.; Eisenberg R.; Holland P. L.; Krauss T. D. Robust Photogeneration of H2 in Water Using Semiconductor Nanocrystals and a Nickel Catalyst. Science 2012, 338, 1321–1324. 10.1126/science.1227775. 23138979

[ref414] Dias E. A.; Saari J. I.; Tyagi P.; Kambhampati P. Improving Optical Gain Performance in Semiconductor Quantum Dots via Coupled Quantum Shells. J. Phys. Chem. C 2012, 116, 5407–5413. 10.1021/jp211325x.

[ref415] Cihan A. F.; Kelestemur Y.; Guzelturk B.; Yerli O.; Kurum U.; Yaglioglu H. G.; Elmali A.; Demir H. V. Attractive versus Repulsive Excitonic Interactions of Colloidal Quantum Dots Control Blue- to Red-Shifting (and Non-shifting) Amplified Spontaneous Emission. J. Phys. Chem. Lett. 2013, 4, 4146–4152. 10.1021/jz402211m.

[ref416] Li Z.-J.; Li X.-B.; Wang J.-J.; Yu S.; Li C.-B.; Tung C.-H.; Wu L.-Z. A robust ″artificial catalyst″ in situ formed from CdTe QDs and inorganic cobalt salts for photocatalytic hydrogen evolution. Energ Environ. Sci. 2013, 6, 465–469. 10.1039/C2EE23898E.

[ref417] Brown K. A.; Dayal S.; Ai X.; Rumbles G.; King P. W. Controlled Assembly of Hydrogenase-CdTe Nanocrystal Hybrids for Solar Hydrogen Production. J. Am. Chem. Soc. 2010, 132, 9672–9680. 10.1021/ja101031r. 20583755

[ref418] Brown K. A.; Wilker M. B.; Boehm M.; Dukovic G.; King P. W. Characterization of Photochemical Processes for H2 Production by CdS Nanorod–[FeFe] Hydrogenase Complexes. J. Am. Chem. Soc. 2012, 134, 5627–5636. 10.1021/ja2116348. 22352762

[ref419] Wilker M. B.; Shinopoulos K. E.; Brown K. A.; Mulder D. W.; King P. W.; Dukovic G. Electron Transfer Kinetics in CdS Nanorod–[FeFe]-Hydrogenase Complexes and Implications for Photochemical H2 Generation. J. Am. Chem. Soc. 2014, 136, 4316–4324. 10.1021/ja413001p. 24564271

[ref420] Nann T.; Ibrahim S. K.; Woi P.-M.; Xu S.; Ziegler J.; Pickett C. J. Water Splitting by Visible Light: A Nanophotocathode for Hydrogen Production. Angew. Chem., Int. Ed. 2010, 49, 1574–1577. 10.1002/anie.200906262. 20140925

[ref421] Wang F.; Wang W.-G.; Wang X.-J.; Wang H.-Y.; Tung C.-H.; Wu L.-Z. A Highly Efficient Photocatalytic System for Hydrogen Production by a Robust Hydrogenase Mimic in an Aqueous Solution. Angew. Chem., Int. Ed. 2011, 50, 3193–3197. 10.1002/anie.201006352. 21365722

[ref422] Jian J.-X.; Liu Q.; Li Z.-J.; Wang F.; Li X.-B.; Li C.-B.; Liu B.; Meng Q.-Y.; Chen B.; Feng K.; et al. Chitosan confinement enhances hydrogen photogeneration from a mimic of the diiron subsite of [FeFe]-hydrogenase. Nat. Commun. 2013, 4, 2695 10.1038/ncomms3695. 24158139

[ref423] Li C.-B.; Li Z.-J.; Yu S.; Wang G.-X.; Wang F.; Meng Q.-Y.; Chen B.; Feng K.; Tung C.-H.; Wu L.-Z. Interface-directed assembly of a simple precursor of [FeFe]-H2ase mimics on CdSe QDs for photosynthetic hydrogen evolution in water. Energ Environ. Sci. 2013, 6, 2597–2602. 10.1039/c3ee40992a.

[ref424] Wu K.; Chen Z.; Lv H.; Zhu H.; Hill C. L.; Lian T. Hole Removal Rate Limits Photo-driven H2 Generation Efficiency in CdS-Pt and CdSe/CdS-Pt Semiconductor Nanorod-metal tip Heterostructures. J. Am. Chem. Soc. 2014, 136, 7708–7716. 10.1021/ja5023893. 24798693

[ref425] Wolff C. M.; Frischmann P. D.; Schulze M.; Bohn B. J.; Wein R.; Livadas P.; Carlson M. T.; Jäckel F.; Feldmann J.; Würthner F.; et al. All-in-one visible-light-driven water splitting by combining nanoparticulate and molecular co-catalysts on CdS nanorods. Nature Energy 2018, 3, 862–869. 10.1038/s41560-018-0229-6.

[ref426] Lian S.; Kodaimati M. S.; Dolzhnikov D. S.; Calzada R.; Weiss E. A. Powering a CO2 Reduction Catalyst with Visible Light through Multiple Sub-picosecond Electron Transfers from a Quantum Dot. J. Am. Chem. Soc. 2017, 139, 8931–8938. 10.1021/jacs.7b03134. 28608682

[ref427] Brown K. A.; Harris D. F.; Wilker M. B.; Rasmussen A.; Khadka N.; Hamby H.; Keable S.; Dukovic G.; Peters J. W.; Seefeldt L. C.; et al. Light-driven dinitrogen reduction catalyzed by a CdS:nitrogenase MoFe protein biohybrid. Science 2016, 352, 448–450. 10.1126/science.aaf2091. 27102481

[ref428] Kalisman P.; Nakibli Y.; Amirav L. Perfect Photon-to-Hydrogen Conversion Efficiency. Nano Lett. 2016, 16, 1776–1781. 10.1021/acs.nanolett.5b04813. 26788824

[ref429] Yan H.; Yang J.; Ma G.; Wu G.; Zong X.; Lei Z.; Shi J.; Li C. Visible-light-driven hydrogen production with extremely high quantum efficiency on Pt–PdS/CdS photocatalyst. J. Catal. 2009, 266, 165–168. 10.1016/j.jcat.2009.06.024.

[ref430] Oshikiri T.; Ueno K.; Misawa H. Plasmon-Induced Ammonia Synthesis through Nitrogen Photofixation with Visible Light Irradiation. Angew. Chem., Int. Ed. 2014, 53, 9802–9805. 10.1002/anie.201404748. 25045027

[ref431] Chen X.; Li N.; Kong Z.; Ong W.-J.; Zhao X. Photocatalytic fixation of nitrogen to ammonia: state-of-the-art advancements and future prospects. Materials Horizons 2018, 5, 9–27. 10.1039/C7MH00557A.

[ref432] Zhu D.; Zhang L.; Ruther R. E.; Hamers R. J. Photo-illuminated diamond as a solid-state source of solvated electrons in water for nitrogen reduction. Nat. Mater. 2013, 12, 836–841. 10.1038/nmat3696. 23812128

[ref433] Zhang N.; Jalil A.; Wu D.; Chen S.; Liu Y.; Gao C.; Ye W.; Qi Z.; Ju H.; Wang C.; et al. Refining Defect States in W18O49 by Mo Doping: A Strategy for Tuning N2 Activation towards Solar-Driven Nitrogen Fixation. J. Am. Chem. Soc. 2018, 140, 9434–9443. 10.1021/jacs.8b02076. 29975522

[ref434] Jiang Z.-J.; Kelley D. F. Hot and Relaxed Electron Transfer from the CdSe Core and Core/Shell Nanorods. J. Phys. Chem. C 2011, 115, 4594–4602. 10.1021/jp112424z.

[ref435] Du P.; Schneider J.; Jarosz P.; Eisenberg R. Photocatalytic Generation of Hydrogen from Water Using a Platinum(II) Terpyridyl Acetylide Chromophore. J. Am. Chem. Soc. 2006, 128, 7726–7727. 10.1021/ja0610683. 16771472

[ref436] Kiwi J.; Gratzel M. Hydrogen evolution from water induced by visible light mediated by redox catalysis. Nature 1979, 281, 657–658. 10.1038/281657a0.

[ref437] Zhu H.; Chen Z.; Wu K.; Lian T. Wavelength Dependent Efficient Photoreduction of Redox Mediators Using Type II ZnSe/CdS Nanorod Heterostructures. Chemical Science 2014, 5, 3905–3914. 10.1039/C4SC01549E.

[ref438] Bridewell V. L.; Alam R.; Karwacki C. J.; Kamat P. V. CdSe/CdS Nanorod Photocatalysts: Tuning the Interfacial Charge Transfer Process through Shell Length. Chem. Mater. 2015, 27, 5064–5071. 10.1021/acs.chemmater.5b01689.

[ref439] Zhao F.; Li Q.; Han K.; Lian T. Mechanism of Efficient Viologen Radical Generation by Ultrafast Electron Transfer from CdS Quantum Dots. J. Phys. Chem. C 2018, 122, 17136–17142. 10.1021/acs.jpcc.8b06551.

[ref440] Chica B.; Wu C.-H.; Liu Y.; Adams M. W. W.; Lian T.; Dyer R. B. Balancing electron transfer rate and driving force for efficient photocatalytic hydrogen production in CdSe/CdS nanorod-[NiFe] hydrogenase assemblies. Energy & Environ. Sci. 2017, 10, 2245–2255. 10.1039/C7EE01738C.

[ref441] Mokari T.; Rothenberg E.; Popov I.; Costi R.; Banin U. Selective Growth of Metal Tips onto Semiconductor Quantum Rods and Tetrapods. Science 2004, 304, 1787–1790. 10.1126/science.1097830. 15205530

[ref442] Costi R.; Saunders A. E.; Elmalem E.; Salant A.; Banin U. Visible Light-Induced Charge Retention and Photocatalysis with Hybrid CdSe–Au Nanodumbbells. Nano Lett. 2008, 8, 637–641. 10.1021/nl0730514. 18197720

[ref443] Wu K.; Chen J.; McBride J. R.; Lian T. Efficient hot-electron transfer by a plasmon-induced interfacial charge-transfer transition. Science 2015, 349, 632–635. 10.1126/science.aac5443. 26250682

[ref444] Habas S. E.; Yang P.; Mokari T. Selective Growth of Metal and Binary Metal Tips on CdS Nanorods. J. Am. Chem. Soc. 2008, 130, 3294–3295. 10.1021/ja800104w. 18302389

[ref445] Chen X.; Shen S.; Guo L.; Mao S. S. Semiconductor-based photocatalytic hydrogen generation. Chem. Rev. 2010, 110, 6503–6570. 10.1021/cr1001645. 21062099

[ref446] Dukovic G.; Merkle M. G.; Nelson J. H.; Hughes S. M.; Alivisatos A. P. Photodeposition of Pt on Colloidal CdS and CdSe/CdS Semiconductor Nanostructures. Adv. Mater. 2008, 20, 4306–4311. 10.1002/adma.200800384.

[ref447] Li Q.; Zhao F.; Qu C.; Shang Q.; Xu Z.; Yu L.; McBride J. R.; Lian T. Two-Dimensional Morphology Enhances Light-Driven H2 Generation Efficiency in CdS Nanoplatelet-Pt Heterostructures. J. Am. Chem. Soc. 2018, 140, 11726–11734. 10.1021/jacs.8b06100. 30145886

[ref448] Zhukovskyi M.; Tongying P.; Yashan H.; Wang Y.; Kuno M. Efficient Photocatalytic Hydrogen Generation from Ni Nanoparticle Decorated CdS Nanosheets. ACS Catal. 2015, 5, 6615–6623. 10.1021/acscatal.5b01812.

[ref449] Li Q.; Lian T. Exciton dissociation dynamics and light-driven H2 generation in colloidal 2D cadmium chalcogenide nanoplatelet heterostructures. Nano Res. 2018, 11, 3031–3049. 10.1007/s12274-018-2024-x.

[ref450] Wu K.; Zhu H.; Liu Z.; Rodríguez-Córdoba W.; Lian T. Ultrafast Charge Separation and Long-Lived Charge Separated State in Photocatalytic CdS–Pt Nanorod Heterostructures. J. Am. Chem. Soc. 2012, 134, 10337–10340. 10.1021/ja303306u. 22655858

[ref451] Khon E.; Lambright K.; Khnayzer R. S.; Moroz P.; Perera D.; Butaeva E.; Lambright S.; Castellano F. N.; Zamkov M. Improving the Catalytic Activity of Semiconductor Nanocrystals through Selective Domain Etching. Nano Lett. 2013, 13, 2016–2023. 10.1021/nl400715n. 23541120

[ref452] Acharya K. P.; Khnayzer R. S.; O’Connor T.; Diederich G.; Kirsanova M.; Klinkova A.; Roth D.; Kinder E.; Imboden M.; Zamkov M. The Role of Hole Localization in Sacrificial Hydrogen Production by Semiconductor–Metal Heterostructured Nanocrystals. Nano Lett. 2011, 11, 2919–2926. 10.1021/nl201388c. 21615085

[ref453] Berr M.; Vaneski A.; Susha A. S.; Rodriguez-Fernandez J.; Doblinger M.; Jackel F.; Rogach A. L.; Feldmann J. Colloidal CdS Nanorods Decorated with Subnanometer Sized Pt Clusters for Photocatalytic Hydrogen Generation. Appl. Phys. Lett. 2010, 97, 093108 10.1063/1.3480613.

[ref454] Amirav L.; Oba F.; Aloni S.; Alivisatos A. P. Modular Synthesis of a Dual Metal–Dual Semiconductor Nano-Heterostructure. Angew. Chem., Int. Ed. 2015, 54, 7007–7011. 10.1002/anie.201411461. 25924726

[ref455] Huang L.; Wang X.; Yang J.; Liu G.; Han J.; Li C. Dual Cocatalysts Loaded Type I CdS/ZnS Core/Shell Nanocrystals as Effective and Stable Photocatalysts for H2 Evolution. J. Phys. Chem. C 2013, 117, 11584–11591. 10.1021/jp400010z.

[ref456] Li X.-B.; Gao Y.-J.; Wang Y.; Zhan F.; Zhang X.-Y.; Kong Q.-Y.; Zhao N.-J.; Guo Q.; Wu H.-L.; Li Z.-J.; et al. Self-Assembled Framework Enhances Electronic Communication of Ultrasmall-Sized Nanoparticles for Exceptional Solar Hydrogen Evolution. J. Am. Chem. Soc. 2017, 139, 4789–4796. 10.1021/jacs.6b12976. 28281343

[ref457] Arias-Rotondo D. M.; McCusker J. K. The photophysics of photoredox catalysis: a roadmap for catalyst design. Chem. Soc. Rev. 2016, 45, 5803–5820. 10.1039/C6CS00526H. 27711624

[ref458] Young R. M.; Jensen S. C.; Edme K.; Wu Y.; Krzyaniak M. D.; Vermeulen N. A.; Dale E. J.; Stoddart J. F.; Weiss E. A.; Wasielewski M. R.; et al. Ultrafast Two-Electron Transfer in a CdS Quantum Dot–Extended-Viologen Cyclophane Complex. J. Am. Chem. Soc. 2016, 138, 6163–6170. 10.1021/jacs.5b13386. 27111529

[ref459] Liu C.; Qiu F.; Peterson J. J.; Krauss T. D. Aqueous Photogeneration of H2 with CdSe Nanocrystals and Nickel Catalysts: Electron Transfer Dynamics. J. Phys. Chem. B 2015, 119, 7349–7357. 10.1021/jp510935w. 25523941

[ref460] Burke R.; Cogan N. M. B.; Oi A.; Krauss T. D. Recovery of Active and Efficient Photocatalytic H2 Production for CdSe Quantum Dots. J. Phys. Chem. C 2018, 122, 14099–14106. 10.1021/acs.jpcc.8b01237.

[ref461] Huang J.; Tang Y.; Mulfort K. L.; Zhang X. The direct observation of charge separation dynamics in CdSe quantum dots/cobaloxime hybrids. Phys. Chem. Chem. Phys. 2016, 18, 4300–4303. 10.1039/C5CP07611K. 26805707

[ref462] Johnson R. C.; Li J.; Hupp J. T.; Schatz G. C. Hyper-Rayleigh scattering studies of silver, copper, and platinum nanoparticle suspensions. Chem. Phys. Lett. 2002, 356, 534–540. 10.1016/S0009-2614(02)00407-4.

[ref463] Cline R. P.; Utterback J. K.; Strong S. E.; Dukovic G.; Eaves J. D. On the Nature of Trapped-Hole States in CdS Nanocrystals and the Mechanism of Their Diffusion. J. Phys. Chem. Lett. 2018, 9, 3532–3537. 10.1021/acs.jpclett.8b01148. 29856225

[ref464] O’Connor T.; Panov M. S.; Mereshchenko A.; Tarnovsky A. N.; Lorek R.; Perera D.; Diederich G.; Lambright S.; Moroz P.; Zamkov M. The Effect of the Charge-Separating Interface on Exciton Dynamics in Photocatalytic Colloidal Heteronanocrystals. ACS Nano 2012, 6, 8156–8165. 10.1021/nn302810y. 22881284

[ref465] Conca E.; Aresti M.; Saba M.; Casula M. F.; Quochi F.; Mula G.; Loche D.; Kim M. R.; Manna L.; Corrias A.; et al. Charge separation in Pt-decorated CdSe@CdS octapod nanocrystals. Nanoscale 2014, 6, 2238–2243. 10.1039/C3NR05567A. 24424255

[ref466] Ithurria S.; Bousquet G.; Dubertret B. Continuous Transition from 3D to 1D Confinement Observed during the Formation of CdSe Nanoplatelets. J. Am. Chem. Soc. 2011, 133, 3070–3077. 10.1021/ja110046d. 21323349

[ref467] Joo J.; Son J. S.; Kwon S. G.; Yu J. H.; Hyeon T. Low-Temperature Solution-Phase Synthesis of Quantum Well Structured CdSe Nanoribbons. J. Am. Chem. Soc. 2006, 128, 5632–5633. 10.1021/ja0601686. 16637619

[ref468] Son J. S.; Wen X.-D.; Joo J.; Chae J.; Baek S. -i.; Park K.; Kim J. H.; An K.; Yu J. H.; Kwon S. G.; et al. Large-Scale Soft Colloidal Template Synthesis of 1.4 nm Thick CdSe Nanosheets. Angew. Chem., Int. Ed. 2009, 48, 6861–6864. 10.1002/anie.200902791. 19688802

[ref469] Son J. S.; Yu J. H.; Kwon S. G.; Lee J.; Joo J.; Hyeon T. Colloidal Synthesis of Ultrathin Two-Dimensional Semiconductor Nanocrystals. Adv. Mater. 2011, 23, 3214–3219. 10.1002/adma.201101334. 21894625

[ref470] Liu Y.; Yang W.; Chen Q.; Cullen D. A.; Xie Z.; Lian T. Pt Particle Size Affects Both the Charge Separation and Water Reduction Efficiencies of CdS–Pt Nanorod Photocatalysts for Light Driven H2 Generation. J. Am. Chem. Soc. 2022, 144, 2705–2715. 10.1021/jacs.1c11745. 35089025

[ref471] Liu Y.; Yang W.; Chen Q.; Xie Z.; Lian T. Nanorod length-dependent photodriven H2 production in 1D CdS–Pt heterostructures. J. Chem. Phys. 2023, 159, 104706 10.1063/5.0157927. 37698197

[ref472] Berr M. J.; Wagner P.; Fischbach S.; Vaneski A.; Schneider J.; Susha A. S.; Rogach A. L.; Jackel F.; Feldmann J. Hole scavenger redox potentials determine quantum efficiency and stability of Pt-decorated CdS nanorods for photocatalytic hydrogen generation. Appl. Phys. Lett. 2012, 100, 223903 10.1063/1.4723575.

[ref473] Simon T.; Bouchonville N.; Berr M. J.; Vaneski A.; Adrović A.; Volbers D.; Wyrwich R.; Döblinger M.; Susha A. S.; Rogach A. L.; et al. Redox shuttle mechanism enhances photocatalytic H2 generation on Ni-decorated CdS nanorods. Nat. Mater. 2014, 13, 1013–1018. 10.1038/nmat4049. 25087066

[ref474] Mooney J.; Krause M. M.; Saari J. I.; Kambhampati P. A microscopic picture of surface charge trapping in semiconductor nanocrystals. J. Chem. Phys. 2013, 138, 204705 10.1063/1.4807054. 23742498

[ref475] Mooney J.; Krause M. M.; Saari J. I.; Kambhampati P. Challenge to the deep-trap model of the surface in semiconductor nanocrystals. Phys. Rev. B 2013, 87, 081201 10.1103/PhysRevB.87.081201.

[ref476] Li X.-B.; Liu B.; Wen M.; Gao Y.-J.; Wu H.-L.; Huang M.-Y.; Li Z.-J.; Chen B.; Tung C.-H.; Wu L.-Z. Hole-Accepting-Ligand-Modified CdSe QDs for Dramatic Enhancement of Photocatalytic and Photoelectrochemical Hydrogen Evolution by Solar Energy. Adv. Sci. 2016, 3, 1500282 10.1002/advs.201500282. PMC5063123 27774400

[ref477] Costantino F.; Kamat P. V. Do Sacrificial Donors Donate H2 in Photocatalysis?. ACS Energy Letters 2022, 7, 242–246. 10.1021/acsenergylett.1c02487.

[ref478] Schneider J.; Bahnemann D. W. Undesired Role of Sacrificial Reagents in Photocatalysis. J. Phys. Chem. Lett. 2013, 4, 3479–3483. 10.1021/jz4018199.

[ref479] Hainer A. S.; Hodgins J. S.; Sandre V.; Vallieres M.; Lanterna A. E.; Scaiano J. C. Photocatalytic Hydrogen Generation Using Metal-Decorated TiO2: Sacrificial Donors vs True Water Splitting. ACS Energy Letters 2018, 3, 542–545. 10.1021/acsenergylett.8b00152.

[ref480] Helm M. L.; Stewart M. P.; Bullock R. M.; DuBois M. R.; DuBois D. L. A synthetic nickel electrocatalyst with a turnover frequency above 100,000 s–1 for H2 production. Science 2011, 333, 863–866. 10.1126/science.1205864. 21836012

[ref481] Wilson A. D.; Newell R. H.; McNevin M. J.; Muckerman J. T.; Rakowski DuBois M.; DuBois D. L. Hydrogen Oxidation and Production Using Nickel-Based Molecular Catalysts with Positioned Proton Relays. J. Am. Chem. Soc. 2006, 128, 358–366. 10.1021/ja056442y. 16390166

[ref482] Nakibli Y.; Mazal Y.; Dubi Y.; Wächtler M.; Amirav L. Size Matters: Cocatalyst Size Effect on Charge Transfer and Photocatalytic Activity. Nano Lett. 2018, 18, 357–364. 10.1021/acs.nanolett.7b04210. 29236508

[ref483] Nakibli Y.; Kalisman P.; Amirav L. Less Is More: The Case of Metal Cocatalysts. J. Phys. Chem. Lett. 2015, 6, 2265–2268. 10.1021/acs.jpclett.5b00872. 26266602

[ref484] Murdoch M.; Waterhouse G. I. N.; Nadeem M. A.; Metson J. B.; Keane M. A.; Howe R. F.; Llorca J.; Idriss H. The effect of gold loading and particle size on photocatalytic hydrogen production from ethanol over Au/TiO2 nanoparticles. Nat. Chem. 2011, 3, 489–492. 10.1038/nchem.1048. 21602866

[ref485] Ben-Shahar Y.; Scotognella F.; Kriegel I.; Moretti L.; Cerullo G.; Rabani E.; Banin U. Optimal metal domain size for photocatalysis with hybrid semiconductor-metal nanorods. Nat. Commun. 2016, 7, 10413 10.1038/ncomms10413. 26783194 PMC4735686

[ref486] Schweinberger F. F.; Berr M. J.; Döblinger M.; Wolff C.; Sanwald K. E.; Crampton A. S.; Ridge C. J.; Jäckel F.; Feldmann J.; Tschurl M.; et al. Cluster Size Effects in the Photocatalytic Hydrogen Evolution Reaction. J. Am. Chem. Soc. 2013, 135, 13262–13265. 10.1021/ja406070q. 23961721

[ref487] Berr M. J.; Schweinberger F. F.; Döblinger M.; Sanwald K. E.; Wolff C.; Breimeier J.; Crampton A. S.; Ridge C. J.; Tschurl M.; Heiz U.; et al. Size-Selected Subnanometer Cluster Catalysts on Semiconductor Nanocrystal Films for Atomic Scale Insight into Photocatalysis. Nano Lett. 2012, 12, 5903–5906. 10.1021/nl3033069. 23043642

[ref488] Amirav L.; Alivisatos A. P. Luminescence Studies of Individual Quantum Dot Photocatalysts. J. Am. Chem. Soc. 2013, 135, 13049–13053. 10.1021/ja404918z. 23895591

[ref489] Bang J. U.; Lee S. J.; Jang J. S.; Choi W.; Song H. Geometric Effect of Single or Double Metal-Tipped CdSe Nanorods on Photocatalytic H2 Generation. J. Phys. Chem. Lett. 2012, 3, 3781–3785. 10.1021/jz301732n. 26291111

[ref490] Simon T.; Carlson M. T.; Stolarczyk J. K.; Feldmann J. Electron Transfer Rate vs Recombination Losses in Photocatalytic H2 Generation on Pt-Decorated CdS Nanorods. ACS Energy Letters 2016, 1, 1137–1142. 10.1021/acsenergylett.6b00468.

[ref491] Nocera D. G. Artificial Leaf. Accounts Chem. Res. 2012, 45, 767–776. 10.1021/ar2003013. 22475039

[ref492] Lewis N. S.; Nocera D. G. Powering the planet: Chemical challenges in solar energy utilization. Proc. Natl. Acad. Sci. U. S. A. 2006, 103, 15729–15735. 10.1073/pnas.0603395103. 17043226 PMC1635072

[ref493] Meyer T. J. Chemical approaches to artificial photosynthesis. Acc. Chem. Res. 1989, 22, 163–170. 10.1021/ar00161a001.

[ref494] Alstrum-Acevedo J. H.; Brennaman M. K.; Meyer T. J. Chemical Approaches to Artificial Photosynthesis. 2. Inorg. Chem. 2005, 44, 6802–6827. 10.1021/ic050904r. 16180838

[ref495] Pirnot M. T.; Rankic D. A.; Martin D. B. C.; MacMillan D. W. C. Photoredox Activation for the Direct β-Arylation of Ketones and Aldehydes. Science 2013, 339, 1593–1596. 10.1126/science.1232993. 23539600 PMC3723331

[ref496] Bernt C. M.; Burks P. T.; DeMartino A. W.; Pierri A. E.; Levy E. S.; Zigler D. F.; Ford P. C. Photocatalytic Carbon Disulfide Production via Charge Transfer Quenching of Quantum Dots. J. Am. Chem. Soc. 2014, 136, 2192–2195. 10.1021/ja4083599. 24151929

[ref497] Li X.-B.; Li Z.-J.; Gao Y.-J.; Meng Q.-Y.; Yu S.; Weiss R. G.; Tung C.-H.; Wu L.-Z. Mechanistic Insights into the Interface-Directed Transformation of Thiols into Disulfides and Molecular Hydrogen by Visible-Light Irradiation of Quantum Dots. Angew. Chem., Int. Ed. 2014, 53, 2085–2089. 10.1002/anie.201310249. 24470069

[ref498] Zhao L.-M.; Meng Q.-Y.; Fan X.-B.; Ye C.; Li X.-B.; Chen B.; Ramamurthy V.; Tung C.-H.; Wu L.-Z. Photocatalysis with Quantum Dots and Visible Light: Selective and Efficient Oxidation of Alcohols to Carbonyl Compounds through a Radical Relay Process in Water. Angew. Chem., Int. Ed. 2017, 56, 3020–3024. 10.1002/anie.201700243. 28177559

[ref499] Caputo J. A.; Frenette L. C.; Zhao N.; Sowers K. L.; Krauss T. D.; Weix D. J. General and Efficient C–C Bond Forming Photoredox Catalysis with Semiconductor Quantum Dots. J. Am. Chem. Soc. 2017, 139, 4250–4253. 10.1021/jacs.6b13379. 28282120

[ref500] Zhang Z.; Edme K.; Lian S.; Weiss E. A. Enhancing the Rate of Quantum-Dot-Photocatalyzed Carbon–Carbon Coupling by Tuning the Composition of the Dot’s Ligand Shell. J. Am. Chem. Soc. 2017, 139, 4246–4249. 10.1021/jacs.6b13220. 28290682

[ref501] Wakerley D. W.; Kuehnel M. F.; Orchard K. L.; Ly K. H.; Rosser T. E.; Reisner E. Solar-driven reforming of lignocellulose to H2 with a CdS/CdOx photocatalyst. Nat. Energy 2017, 2, 17021 10.1038/nenergy.2017.21.

[ref502] Kuehnel M. F.; Wakerley D. W.; Orchard K. L.; Reisner E. Photocatalytic Formic Acid Conversion on CdS Nanocrystals with Controllable Selectivity for H2 or CO. Angew. Chem., Int. Ed. 2015, 54, 9627–9631. 10.1002/anie.201502773. PMC4552973 26201752

[ref503] Pal A.; Ghosh I.; Sapra S.; König B. Quantum Dots in Visible-Light Photoredox Catalysis: Reductive Dehalogenations and C–H Arylation Reactions Using Aryl Bromides. Chem. Mater. 2017, 29, 5225–5231. 10.1021/acs.chemmater.7b01109.

[ref504] Xi Z.-W.; Yang L.; Wang D.-Y.; Pu C.-D.; Shen Y.-M.; Wu C.-D.; Peng X.-G. Visible-Light Photocatalytic Synthesis of Amines from Imines via Transfer Hydrogenation Using Quantum Dots as Catalysts. Journal of Organic Chemistry 2018, 83, 11886–11895. 10.1021/acs.joc.8b01651. 30168324

[ref505] Warrier M.; Lo M. K. F.; Monbouquette H.; Garcia-Garibay M. A. Photocatalytic reduction of aromatic azides to amines using CdS and CdSe nanoparticles. Photochem. Photobiol. Sci. 2004, 3, 859–863. 10.1039/b408152h. 15346187

[ref506] Jensen S. C.; Bettis Homan S.; Weiss E. A. Photocatalytic Conversion of Nitrobenzene to Aniline through Sequential Proton-Coupled One-Electron Transfers from a Cadmium Sulfide Quantum Dot. J. Am. Chem. Soc. 2016, 138, 1591–1600. 10.1021/jacs.5b11353. 26784531

[ref507] Simlandy A. K.; Bhattacharyya B.; Pandey A.; Mukherjee S. Picosecond Electron Transfer from Quantum Dots Enables a General and Efficient Aerobic Oxidation of Boronic Acids. ACS Catal. 2018, 8, 5206–5211. 10.1021/acscatal.8b01078.

[ref508] Huang Y.; Zhu Y.; Egap E. Semiconductor Quantum Dots as Photocatalysts for Controlled Light-Mediated Radical Polymerization. ACS Macro Lett. 2018, 7, 184–189. 10.1021/acsmacrolett.7b00968. 35610890

[ref509] Garakyaraghi S.; Mongin C.; Granger D. B.; Anthony J. E.; Castellano F. N. Delayed Molecular Triplet Generation from Energized Lead Sulfide Quantum Dots. J. Phys. Chem. Lett. 2017, 8, 1458–1463. 10.1021/acs.jpclett.7b00546. 28300410

[ref510] Luo X.; Han Y.; Chen Z.; Li Y.; Liang G.; Liu X.; Ding T.; Nie C.; Wang M.; Castellano F. N.; et al. Mechanisms of triplet energy transfer across the inorganic nanocrystal/organic molecule interface. Nat. Commun. 2020, 11, 28 10.1038/s41467-019-13951-3. 31911606 PMC6946700

[ref511] Luo X.; Liang G.; Han Y.; Li Y.; Ding T.; He S.; Liu X.; Wu K. Triplet Energy Transfer from Perovskite Nanocrystals Mediated by Electron Transfer. J. Am. Chem. Soc. 2020, 142, 11270–11278. 10.1021/jacs.0c04583. 32479073

[ref512] Wang J.; Ding T.; Nie C.; Wang M.; Zhou P.; Wu K. Spin-Controlled Charge-Recombination Pathways across the Inorganic/Organic Interface. J. Am. Chem. Soc. 2020, 142, 4723–4731. 10.1021/jacs.9b12724. 32070096

[ref513] Zhao G.; Chen Z.; Xiong K.; Liang G.; Zhang J.; Wu K. Triplet energy migration pathways from PbS quantum dots to surface-anchored polyacenes controlled by charge transfer. Nanoscale 2021, 13, 1303–1310. 10.1039/D0NR07837A. 33409530

[ref514] Huang Z.; Xu Z.; Mahboub M.; Liang Z.; Jaimes P.; Xia P.; Graham K. R.; Tang M. L.; Lian T. Enhanced Near-Infrared-to-Visible Upconversion by Synthetic Control of PbS Nanocrystal Triplet Photosensitizers. J. Am. Chem. Soc. 2019, 141, 9769–9772. 10.1021/jacs.9b03385. 31180212

[ref515] Jin T.; Uhlikova N.; Xu Z.; Zhu Y.; Huang Y.; Egap E.; Lian T. Enhanced triplet state generation through radical pair intermediates in BODIPY-quantum dot complexes. J. Chem. Phys. 2019, 151, 241101 10.1063/1.5136045. 31893904

[ref516] Xu Z.; Jin T.; Huang Y.; Mulla K.; Evangelista F. A.; Egap E.; Lian T. Direct triplet sensitization of oligothiophene by quantum dots. Chemical Science 2019, 10, 6120–6124. 10.1039/C9SC01648A. 31360418 PMC6585591

[ref517] Huang Z.; Xu Z.; Huang T.; Gray V.; Moth-Poulsen K.; Lian T.; Tang M. L. Evolution from Tunneling to Hopping Mediated Triplet Energy Transfer from Quantum Dots to Molecules. J. Am. Chem. Soc. 2020, 142, 17581–17588. 10.1021/jacs.0c07727. 32969652

[ref518] Jin T.; Lian T. Trap state mediated triplet energy transfer from CdSe quantum dots to molecular acceptors. J. Chem. Phys. 2020, 153, 074703 10.1063/5.0022061. 32828113

[ref519] Jin T.; Uhlikova N.; Xu Z.; Zhu Y.; Huang Y.; Egap E.; Lian T. Competition of Dexter, Förster, and charge transfer pathways for quantum dot sensitized triplet generation. J. Chem. Phys. 2020, 152, 214702 10.1063/5.0009833. 32505156

[ref520] Xu Z.; Huang Z.; Li C.; Huang T.; Evangelista F. A.; Tang M. L.; Lian T. Tuning the Quantum Dot (QD)/Mediator Interface for Optimal Efficiency of QD-Sensitized Near-Infrared-to-Visible Photon Upconversion Systems. ACS Appl. Mater. Interfaces 2020, 12, 36558–36567. 10.1021/acsami.0c10269. 32677433

[ref521] Xu Z.; Huang Z.; Jin T.; Lian T.; Tang M. L. Mechanistic Understanding and Rational Design of Quantum Dot/Mediator Interfaces for Efficient Photon Upconversion. Acc. Chem. Res. 2021, 54, 70–80. 10.1021/acs.accounts.0c00526. 33141563

[ref522] Jin T.; He S.; Zhu Y.; Egap E.; Lian T. Bright State Sensitized Triplet Energy Transfer from Quantum Dot to Molecular Acceptor Revealed by Temperature Dependent Energy Transfer Dynamics. Nano Lett. 2022, 22, 3897–3903. 10.1021/acs.nanolett.2c00017. 35561343

[ref523] Miyashita T.; Jaimes P.; Lian T.; Tang M. L.; Xu Z. Quantifying the Ligand-Induced Triplet Energy Transfer Barrier in a Quantum Dot-Based Upconversion System. J. Phys. Chem. Lett. 2022, 13, 3002–3007. 10.1021/acs.jpclett.2c00514. 35347991

[ref524] Jiang Y.; Wang C.; Rogers C. R.; Kodaimati M. S.; Weiss E. A. Regio- and diastereoselective intermolecular [2 + 2] cycloadditions photocatalysed by quantum dots. Nat. Chem. 2019, 11, 1034–1040. 10.1038/s41557-019-0344-4. 31654049 PMC6820707

[ref525] Jiang Y.; Yang M.; Wu Y.; López-Arteaga R.; Rogers C. R.; Weiss E. A. Chemo- and stereoselective intermolecular [2 + 2] photocycloaddition of conjugated dienes using colloidal nanocrystal photocatalysts. Chem. Catalysis 2021, 1, 106–116. 10.1016/j.checat.2021.02.001. 34337591 PMC8323757

[ref526] Jiang Y.; López-Arteaga R.; Weiss E. A. Quantum Dots Photocatalyze Intermolecular [2 + 2] Cycloadditions of Aromatic Alkenes Adsorbed to their Surfaces via van der Waals Interactions. J. Am. Chem. Soc. 2022, 144, 3782–3786. 10.1021/jacs.2c00833. 35230100

[ref527] Liu M.; Xia P.; Zhao G.; Nie C.; Gao K.; He S.; Wang L.; Wu K. Energy-Transfer Photocatalysis Using Lead Halide Perovskite Nanocrystals: Sensitizing Molecular Isomerization and Cycloaddition. Angew. Chem., Int. Ed. 2022, 61, e202208241 10.1002/anie.202208241. 35796033

[ref528] Nie C.; Lin X.; Zhao G.; Wu K. Low-Toxicity ZnSe/ZnS Quantum Dots as Potent Photoreductants and Triplet Sensitizers for Organic Transformations. Angew. Chem., Int. Ed. 2022, 61, e202213065 10.1002/anie.202213065. 36250269

[ref529] Kodaimati M. S.; McClelland K. P.; He C.; Lian S.; Jiang Y.; Zhang Z.; Weiss E. A. Viewpoint: Challenges in Colloidal Photocatalysis and Some Strategies for Addressing Them. Inorg. Chem. 2018, 57, 3659–3670. 10.1021/acs.inorgchem.7b03182. 29561594

[ref530] Lin Y.; Guo J.; San Martin J.; Han C.; Martinez R.; Yan Y. Photoredox Organic Synthesis Employing Heterogeneous Photocatalysts with Emphasis on Halide Perovskite. Chem.-Eur. J. 2020, 26, 13118–13136. 10.1002/chem.202002145. 32533611

[ref531] Yuan Y.; Jin N.; Saghy P.; Dube L.; Zhu H.; Chen O. Quantum Dot Photocatalysts for Organic Transformations. J. Phys. Chem. Lett. 2021, 12, 7180–7193. 10.1021/acs.jpclett.1c01717. 34309389

[ref532] Wu H.-L.; Qi M.-Y.; Tang Z.-R.; Xu Y.-J. Semiconductor quantum dots: a versatile platform for photoredox organic transformation. J. Mater. Chem. A 2023, 11, 3262–3280. 10.1039/D2TA09423A.

[ref533] Ye C.; Zhang D.-S.; Chen B.; Tung C.-H.; Wu L.-Z. Quantum dots: Another choice to sensitize organic transformations. Chemical Physics Reviews 2023, 4, 011304 10.1063/5.0126893.

[ref534] Liang W.; Nie C.; Du J.; Han Y.; Zhao G.; Yang F.; Liang G.; Wu K. Near-infrared photon upconversion and solar synthesis using lead-free nanocrystals. Nat. Photonics 2023, 17, 346–353. 10.1038/s41566-023-01156-6.

